# Supplementary material for: The role of novel biomarkers in the early diagnosis of pancreatic cancer: A systematic review and meta-analysis
Source: PLoS One. 2025 May 23;20(5):e0322720. doi: 10.1371/journal.pone.0322720 (PMC12101772; doi:10.1371/journal.pone.0322720)
Supplement: S1 file — S1 Fig. Sensitivity with CA19–9 + Protein Biomarker (Either-Positive).S2 Fig. Sensitivity with CA19–9 + Protein Biomarker (Predictive/ Machine Learning). S1 Table. Detailed search strategy for the systematic review.S2 Table. QUADAS-2 review items.S3 Table. QUADAS-2 review results.S4 Table. Characteristics and diagnostic outcomes of studies using protein or ctDNA combined with CA19–9 biomarkers.S5 Table. Characteristics and diagnostic outcomes of studies in healthy control populations.S6 Table. Diagnostic accuracy of protein biomarkers and miRNA biomarkers using specific detection methods for early pancreatic cancer.S7 Table. Results of leave-one-out analysis. (PDF) [file pone.0322720.s001.pdf]

Supporting information		
**Supplementary Fig 1**	Sensitivity of Protein Biomarkers Combined with CA19-9 Using the Either-Positive Principle	Page 2
**Supplementary Fig 2**	Sensitivity of Protein Biomarkers Combined with CA19-9 Using Predictive Models or Machine Learning	Page 2
**Supplementary Table 1**	Detailed Search Strategy for the Systematic Review	Page 3
**Supplementary Table 2**	QUADAS-2 Review Items	Page 5
**Supplementary Table 3**	QUADAS-2 Review Results	Page 6
**Supplementary Table 4**	Characteristics and Diagnostic outcomes of Studies Using Protein or ctDNA Combined with CA19-9 Biomarkers	Page 8
**Supplementary Table 5**	Characteristics and Diagnostic outcomes of Studies in Healthy Control Populations	Page 9
**Supplementary Table 6**	Diagnostic Accuracy of Protein Biomarkers and miRNAs Using Specific Detection Methods for Early Pancreatic Cancer	Page 11
**Supplementary Table 7**	Results of leave-one-out analysis	Page 12

Supplementary Fig 1. Sensitivity of Protein Biomarkers Combined with CA19-9 Using the Either-Positive Principle

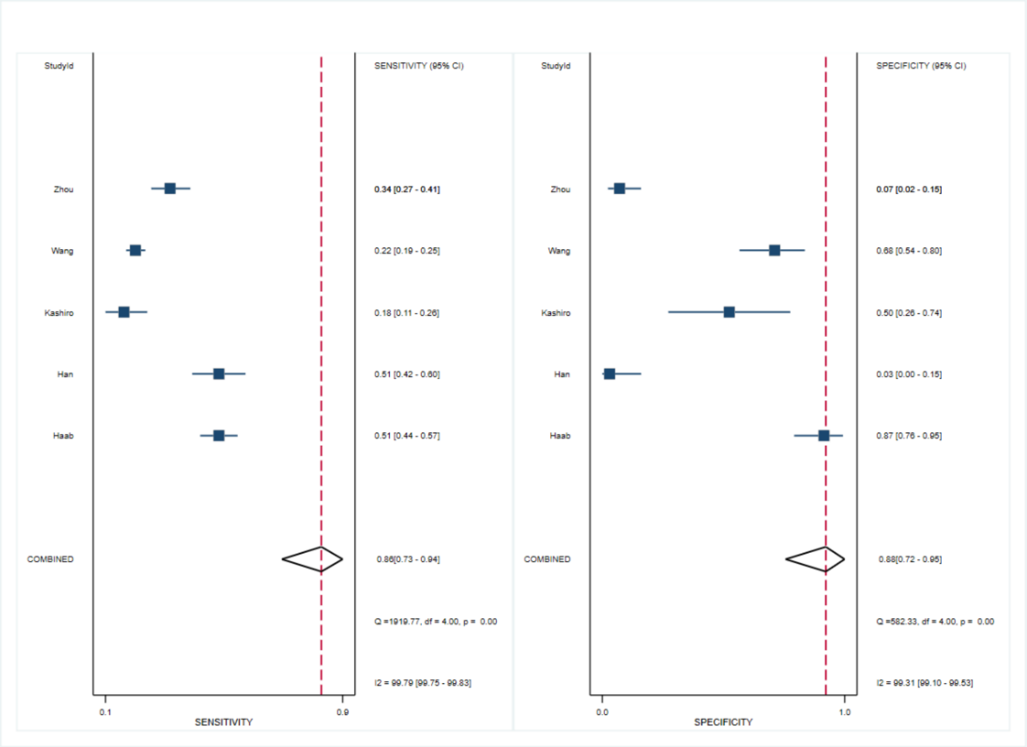

Supplementary Fig 2. Sensitivity of Protein Biomarkers Combined with CA19-9 Using Predictive Models or Machine Learning

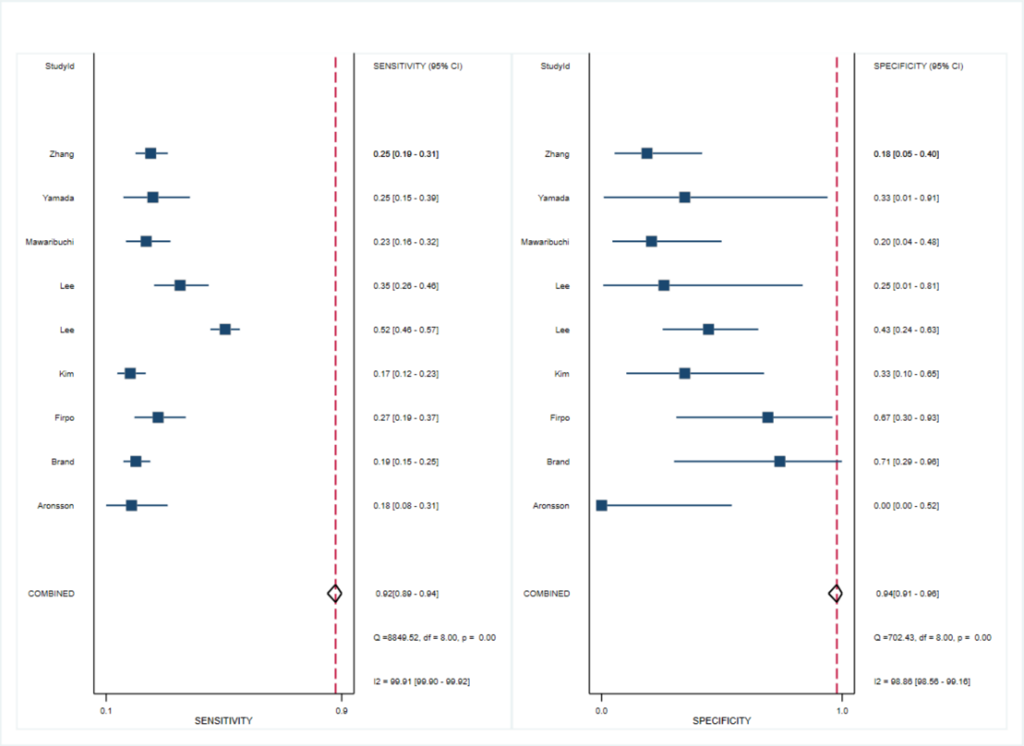

Supplementary Table 1. Detailed Search Strategy for the Systematic Review

electronic databases	keywords
**Pubmed**	#1 "Pancreatic Neoplasms"[Mesh] OR "pancreatic cancer" OR "pancreatic carcinoma" OR "pancreatic tumor"<br>#2 "Early Detection of Cancer"[Mesh] OR "early diagnosis" OR "early detection" OR "screening"<br>#3 "Biomarkers, Tumor"[Mesh] OR "biomarker" OR "biomarkers" OR "circulating tumor DNA" OR "ctDNA" OR "microRNA" OR "miRNA" OR "protein marker" OR "genetic marker"<br>#4 "Sensitivity and Specificity"[Mesh] OR "diagnostic accuracy" OR "sensitivity" OR "specificity" OR "positive predictive value" OR "negative predictive value"<br>#5 #1 AND #2 AND #3 AND #4<br>#6 Filters: Publication date from 2014/01/01 to 2024/05/31; Humans; English
**Embase**	#1 'pancreas tumor'/exp OR 'pancreatic cancer' OR 'pancreatic carcinoma' OR 'pancreatic tumor'<br>#2 'early cancer detection'/exp OR 'early diagnosis' OR 'early detection' OR 'screening'<br>#3 'tumor biomarker'/exp OR 'biomarker' OR 'biomarkers' OR 'circulating tumor DNA' OR 'ctDNA' OR 'microRNA' OR 'miRNA' OR 'protein marker' OR 'genetic marker'<br>#4 'diagnostic accuracy'/exp OR 'sensitivity' OR 'specificity' OR 'positive predictive value' OR 'negative predictive value'<br>#5 #1 AND #2 AND #3 AND #4<br>#6 Filters: Publication year from 2014 to 2024; Human; English
**Cochrane Central Register**	#1 [mh "Pancreatic Neoplasms"] OR "pancreatic cancer" OR "pancreatic carcinoma" OR "pancreatic tumor"<br>#2 [mh "Early Detection of Cancer"] OR "early diagnosis" OR "early detection" OR "screening"<br>#3 [mh "Biomarkers, Tumor"] OR "biomarker" OR "biomarkers" OR "circulating tumor DNA" OR "ctDNA" OR "microRNA" OR "miRNA" OR "protein marker" OR "genetic marker"<br>#4 [mh "Sensitivity and Specificity"] OR "diagnostic accuracy" OR "sensitivity" OR "specificity" OR "positive predictive value" OR "negative predictive value"<br>#5 #1 AND #2 AND #3 AND #4<br>#6 Filters: Publication date from Jan 2014 to May 2024; Humans; English

**Web Of Science**	#1 TS=("pancreatic cancer" OR "pancreatic carcinoma" OR "pancreatic tumor")<br>#2 TS=("early diagnosis" OR "early detection" OR "screening")<br>#3 TS=("biomarker" OR "biomarkers" OR "circulating tumor DNA" OR "ctDNA" OR "microRNA" OR "miRNA" OR "protein marker" OR "genetic marker")<br>#4 TS=("diagnostic accuracy" OR "sensitivity" OR "specificity" OR "positive predictive value" OR "negative predictive value")<br>#5 #1 AND #2 AND #3 AND #4<br>#6 Filters: Timespan=2014-2024; Document Types=Article; Languages=English

Supplementary Table 2. QUADAS-2 Review Items

**Domain**	**Patient Selection**	**Index Test**	**Reference Standard**	**Flow and Timing**
**Questions (yes/no/unclear)**	Was the patient sample consecutive or randomly selected?	Were the results of the index test interpreted without knowledge of the reference standard results?	Did the reference standard correctly classify the target disease?	Was the time interval between the index test and reference standard appropriate?
				Did all patients receive the reference standard?
	Was a case-control design avoided?	Was the threshold for the index test pre-specified?	Were the reference standard results interpreted without knowledge of the index test results?	Did all patients receive the same reference standard?
	Were inappropriate exclusions avoided?			Were all patients included in the analysis?
**Risk of bias(High/Low/Unclear)**				
**Applicability Concerns**				

Supplementary Table 3. QUADAS-2 Review Results

author	year	Risk of Bias				Applicability Concerns		
		Patient Selection	Index Test	Reference Standard	Flow and Timing	Patient Selection	Index Test	Reference Standard
**Fujimoto**	2021	High	Low	Unclear	Low	Low	Low	Low
**Majumder**	2021	High	Low	Low	Low	Low	Low	Low
**Wu**	2022	High	Low	Low	Low	Low	Low	Low
**Bauden**	2015	High	Low	Low	Low	Low	Low	Low
**Henriksen**	2016	Low	Low	Low	Low	Low	Low	Low
**Eissa**	2019	High	Low	Low	Low	Low	Low	Low
**Cohen**	2017	Low	Low	Low	Low	Low	Low	Low
**Debernardi**	2015	High	Low	Low	Low	Low	Low	Low
**Masterson**	2023	Low	Low	Low	Low	Low	Low	Low
**Yu**	2020	Unclear	Low	Low	Low	Low	Low	Low
**Nakamura**	2022	Low	Low	Low	Low	Low	Low	Low
**Huang**	2024	Low	Low	Low	Low	Low	Low	Low
**Schultz**	2014	Unclear	Low	Low	Low	Low	Low	Low
**Ganepola**	2014	High	Low	Low	Low	Low	Low	Low
**Wolrab**	2021	Low	Low	Low	Low	Low	Low	Low
**Fukutake**	2015	Unclear	Low	Low	Low	Low	Low	Low
**Xie**	2015	High	Low	Low	Low	Low	Low	Low
**Zhang**	2014	High	Low	Low	Low	Low	Low	Low
**Mayerle**	2017	Unclear	Low	Low	Low	Low	Low	Low
**Hirata**	2017	Unclear	Low	Low	Low	Low	Low	Low
**Yamada**	2019	High	Low	Low	Low	Low	Low	Low
**Wang**	2014	High	Low	Low	Low	Low	Low	Low
**Matsunaga**	2017	Unclear	Low	Low	Low	Low	Low	Low
**Zhou**	2018	High	Low	Low	Low	Low	Low	Low
**Sato**	2020	Low	Low	Low	Low	Low	Low	Low
**Mawaribuchi**	2023	High	Low	Low	Low	Low	Low	Low
**Nam**	2022	Unclear	Low	Low	Low	Low	Low	Low
**Han**	2015	High	Low	Low	Low	Low	Low	Low
**Kashiro**	2024	Low	Low	Low	Low	Low	Low	Low
**Yu**	2021	High	Low	Low	Low	Low	Low	Low
**Mohamed**	2015	High	Low	Low	Low	Low	Low	Low
**Radon**	2015	High	Low	Low	Low	Low	Low	Low
**Li**	2023	Low	Low	Low	Low	Low	Low	Low
**Lee. M**	2021	Unclear	Low	Low	Low	Low	Low	Low
**Brand**	2022	Low	Low	Low	Low	Low	Low	Low
**Aronsson**	2018	High	Low	Low	Low	Low	Low	Low
**Wen**	2024	High	Low	Low	Low	Low	Low	Low
**Haab**	2024	High	Low	Low	Low	Low	Low	Low

**Kim**	2020	Unclear	Low	Low	Low	Low	Low	Low
**Lee. D**	2021	Unclear	Low	Low	Unclear	Low	Low	Low
**Firpo**	2023	Unclear	Low	Low	Low	Low	Low	Low
**Zhang**	2013	Unclear	Low	Low	Low	Low	Low	Low

Supplementary Table 4. Characteristics and Diagnostic outcomes of Studies Using Protein or ctDNA Combined with CA19-9 Biomarkers

author	year	tp	fp	fn	tn	sample size	biomarker name
**Yamada**	2019	14	2	1	41	58	anti-3'-sialyllactose antibodies+CA19-9
**Wang**	2014	134	18	38	482	672	Macrophage inhibitory cytokine 1+CA19-9
**Haab**	2024	122	7	48	118	295	CA199STRA+MUC16STRA+CA19-9
**Kim**	2020	38	8	4	183	233	ApoA1、CA125、CA19-9、CEA、ApoA2 and TTR
**Mawaribuchi**	2023	26	12	3	87	128	rBC2LCN-reactive SERPINA3+CA19-9
**Brand**	2022	51	2	5	214	272	IMMray PanCan-d test+CA19-9
**Aronsson**	2018	9	5	0	42	56	IL-17E, B7-1, DR6+CA19-9
**Lee**	2023	180	16	12	167	375	LRG1, TTR, CA 19-9
**Han**	2015	61	33	1	59	154	Serum dickkopf-1+CA19-9
**Firpo**	2023	26	3	6	69	104	31 analytes Serum Biomarker Panel
**Kashiro**	2024	21	9	9	97	136	apolipoprotein−A2 isoforms+CA19-9
**Zhang**	2013	54	18	4	165	241	CA19-9, ALB, CRP and IL-8
**Lee**	2021	34	3	1	62	100	Complement Factor B (CFB)+CA19-9
**Zhou**	2018	67	68	5	131	271	serum glypican-1+CA19-9
**Cohen**	2017	132	1	89	181	403	CA19-9+KRAS ctDNA
**Majumder**	2021	41	3	9	47	100	Thirteen MDMs+CA19-9
**Wu**	2022	57	4	12	33	106	PDACatch+CA19-9
**Bauden**	2015	23	3	2	31	49	5-methylcytosine, H2AZ, H2A1.1, H3K4Me2+CA19-9
**Fujimoto**	2021	7	6	2	86	101	Methylated RUNX3+CA19-9

Supplementary Table 5. Characteristics and Diagnostic outcomes of Studies in Healthy Control Populations

author	year	tp	fp	fn	tn	sample size	biomarker name
**Zhou**	2018	49	48	23	115	235	serum glypican-1
**Yamada**	2019	9	8	6	35	58	anti-3'-sialyllactose antibodies
**Wang**	2014	112	18	60	482	672	Macrophage inhibitory cytokine 1
**Matsunaga**	2017	22	14	4	47	87	S100P in Duodenal Fluid
**Mawaribuchi**	2023	26	18	3	81	128	rBC2LCN-reactive SERPINA3
**Nam**	2022	106	13	5	186	310	Asprosin
**Kashiro**	2024	15	5	15	101	136	apolipoprotein-A2 isoforms
**Yu**	2021	20	1	17	35	73	selected protein panels
**Mohamed**	2015	19	11	2	9	41	MIC-1
**Radon**	2015	12	6	3	20	41	LYVE-1, REG1A, and TFF1 in Urine
**Li**	2023	36	25	31	145	237	A panel of three TAAbs (anti-HEXB, anti-TXLNA, and anti-SLAMF6)
**Brand**	2022	49	2	7	214	272	IMMray PanCan-d test
**Aronsson**	2018	7	9	2	38	56	IL-17E, B7-1, DR6
**Wen**	2024	43	18	8	70	139	N-glycan biosignatures
**Majumder**	2021	34	3	16	47	100	Thirteen MDMs
**Wu**	2022	49	4	20	33	106	PDACatch
**Bauden**	2015	10	3	15	21	49	5-methylcytosine, H2AZ, H2A1.1, H3K4Me2, H2AK119Ub
**Henriksen**	2016	29	21	11	103	164	BMP3 RASSF1A、BNC1、MESTv2、TFPI2、APC、SFRP1、SFRP2
**Eissa**	2019	36	8	1	87	132	promoter DNA methylation of the genes ADAMTS1 and BNC1
**Cohen**	2017	66	1	155	181	403	KRAS ctDNA
**Debernardi**	2015	5	1	1	25	32	urinary miRNA miR-143 + miR-30e
**Masterson**	2023	34	1	1	60	96	microRNA-10b+microRNA-let7a
**Nakamura**	2022	40	3	10	30	83	13 markers (5 cf- and 8 exo-miRNAs)
**Huang**	2024	97	15	6	183	301	miRNA Panel（hsa-miR-132-3p, hsa-miR-30c-5p, hsa-miR-24-3p, hsa-miR-23a-3p）
**Schultz**	2014	56	58	14	264	392	Index2
**Zhang**	2014	13	30	2	175	220	serum unsaturated fatty acids（Panel a: C16:1 Palmitoleic acid , C18:3 linolenic acid, C18:2 linoleic acid, C20:4 arachidonic acid and C22:6 docosahexaenoic acid）
**Mayerle**	2017	36	15	4	65	120	blood-derived metabolite biomarkers

**Hirata**	2017	38	6	16	52	112	metabolites(histidine, xylitol)
**Fukutake**	2015	54	1554	30	6218	7856	six plasma free amino acids: serine, asparagine, isoleucine, alanine, histidine, and tryptophan
**Wolrab**	2021	38	1	1	38	78	serum lipids(sphingomyelins (SM), ceramides (Cer), phosphatidylcholines (PC), lysophosphatidylcholines (LPC))

Supplementary Table 6. Diagnostic Accuracy of Protein Biomarkers and miRNAs Using Specific Detection Methods for Early Pancreatic Cancer

author	year	tp	fp	fn	tn	sample size	biomarker name
**Wang**	2014	112	18	60	482	672	Macrophage inhibitory cytokine 1
**Matsunaga**	2017	22	14	4	47	87	S100P in Duodenal Fluid
**Zhou**	2018	49	58	23	141	271	serum glypican-1
**Sato**	2020	11	395	6	3766	4178	Apolipoprotein A2 isoforms (apoA2-i)
**Mawaribuchi**	2023	26	18	3	81	128	rBC2LCN-reactive SERPINA3
**Nam**	2022	106	13	5	186	310	Asprosin
**Han**	2015	53	19	9	73	154	Serum dickkopf-1
**Kashiro**	2024	15	5	15	101	136	apolipoprotein-A2 isoforms
**Yu**	2021	20	1	17	35	73	selected protein panels
**Mohamed**	2015	19	11	2	9	41	MIC-1
**Radon**	2015	12	6	3	20	41	LYVE-1, REG1A, and TFF1 in Urine
**Li**	2023	36	25	31	145	237	A panel of three TAAbs (anti-HEXB, anti-TXLNA, and anti-SLAMF6)
**Lee**	2021	33	3	2	62	100	Complement Factor B (CFB)
**Yamada**	2019	9	8	6	35	58	anti-3'-sialyllactose antibodies
**Debernardi**	2015	5	1	1	25	32	urinary miRNA miR-143 + miR-30e
**Yu**	2020	33	6	9	85	133	serum miRNA-25
**Nakamura**	2022	40	3	10	30	83	13 markers (5 cf- and 8 exo-miRNAs)
**Huang**	2024	98	81	5	271	455	miRNA Panel（hsa-miR-132-3p, hsa-miR-30c-5p, hsa-miR-24-3p, hsa-miR-23a-3p）
**Ganepola**	2014	10	2	1	20	33	miR-642b-3p, miR-885-5p, miR-22-3p
**Schultz**	2014	56	68	14	279	417	Index2

Supplementary Table 7. Results of leave-one-out analysis

Study	year	Sensitivity (%)	I^2 (%)	Specificity (%)	I^2 (%)
**ctDNA Studies**		65	94.33	94	58.08
**without Bauden**	2015	69	95.47	94	88.66
**without Cohen**	2017	72	80.5	90	43.42
**without Eissa**	2019	58	92.25	94	86.97
**without Fujimoto**	2021	67	95.32	94	87.87
**without Henriksen**	2016	65	94.87	95	76.52
**without Majumder**	2021	65	94.89	94	87
**without Wu**	2022	65	94.54	94	87.01
**miRNA Studies**		88	77.36	91	91.27
**without Debernardi**	2015	88	79.26	90	90.94
**without Ganepola**	2014	88	80.65	91	92.36
**without Huang**	2024	85	59.28	93	91.62
**without Masterson**	2023	85	62.78	88	77.81
**without Nakamura**	2022	90	81.95	91	93.81
**without Schultz**	2014	89	79.77	92	91.84
**without Yu**	2020	90	81.09	90	91.97
**Metabolite Studies**		84	81.17	85	98.05
**without Fukutake**	2015	86	75.53	87	74.48
**without Hirata**	2017	87	84.55	86	98.54
**without Mayerle**	2017	83	82.37	86	98.73
**without Wolrab**	2021	79	70.49	84	95.66
**without Xie**	2015	84	83.63	86	98.71
**without Xie**	2015	86	84.15	86	98.98
**without Zhang**	2014	83	83.09	83	97.95
**Protein Studies**		79	83.61	88	92.76
**without Aronsson**	2018	79	84.71	88	93.15
**without Brand**	2022	78	83.38	86	92.55
**without Han**	2015	78	83.64	89	93
**without Kashiro**	2024	80	83.22	87	93.06
**without Lee**	2021	78	82.95	87	93.16
**without Li**	2023	80	82.01	88	93.16
**without Matsunaga**	2017	78	84.34	89	92.99
**without Mawaribuchi**	2023	78	83.88	88	93.1
**without Mohamed**	2015	78	84.13	89	91.87
**without Nam**	2022	77	77.28	88	93.07
**without Radon**	2015	79	84.62	89	93.12
**without Sato**	2020	80	84.51	88	92.53

**without Wang**	2014	80	84.35	87	92.41
**without Wen**	2024	78	83.98	89	93.02
**without Yamada**	2019	80	84.57	88	93.17
**without Yu**	2021	80	83.5	87	93.13
**without Zhou**	2018	80	84.3	89	90.35
