# Supplementary material for: The role of novel biomarkers in the early diagnosis of pancreatic cancer: A systematic review and meta-analysis
Source: PLoS One. 2025 May 23;20(5):e0322720. doi: 10.1371/journal.pone.0322720 (PMC12101772; doi:10.1371/journal.pone.0322720)
Supplement: S2 file — (PDF) [file pone.0322720.s002.pdf]

	Author	Year	Title	Journal	Reason
1		2017	Immunotherapy Bridge 2016 and Melanoma Bridge 2016: Meeting Abstracts	Journal of Translational Medicine	Records excluded after screening
2		2017	New Biomarker Identified for PDAC	Cancer discovery	Records excluded after screening
3	Aalami, A. H., H. Abdeahad, M. Mesgari and A. Sahebkar	2021	MicroRNA-223 in gastrointestinal cancers: A systematic review and diagnostic meta-analysis	European Journal of Clinical Investigation	Records excluded after screening
4	Abdallah, R., V. Taly, S. Zhao, D. Pietrasz, J. B. Bachet, D. Basile, L. Mas, A. Zaanan, P. Laurent-Puig and J. Taieb	2020	Plasma circulating tumor DNA in pancreatic adenocarcinoma for screening, diagnosis, prognosis, treatment and follow-up: A systematic review	Cancer Treatment Reviews	Records excluded after screening
5	Abe, N., K. Matsuo, T. Kumasaka, K. Naka, S. Hashimoto, T. Takemura, M. Fujiwara, Y. Ito, R. Nakata, T. Hashimoto, M. Makuuchi, Y. Soejima and M. Sawabe	2016	Systematic cytological evaluation and immunocytochemistry of minichromosome maintenance protein 2 and p53 significantly improve cytological diagnosis of pancreaticobiliary adenocarcinoma	Journal of medical and dental sciences	Records excluded after screening
6	Abe, T., C. Koi, S. Kohi, K. B. Song, K. Tamura, A. Macgregor-Das, N. Kitaoka, M. Chuidian, M. Ford, M. Dbouk, M. Borges, J. He, R. Burkhart, C. L. Wolfgang, A. P. Klein, J. R. Eshleman, R. H. Hruban, M. I. Canto and M. Goggins	2020	Gene Variants That Affect Levels of Circulating Tumor Markers Increase Identification of Patients With Pancreatic Cancer	Clinical Gastroenterology and Hepatology	Records excluded after screening
7	Acedo, P., K. Fraser, D. Osei, A. Garcia-Sampedro, A. Ney, M. Zaccaria, B. Marin and S. P. Pereira	2023	Profiling immuno-inflammatory and serum biomarkers for the early detection of pancreatic cancer	Pancreatology	Records excluded after screening
8	Adam, M. G., G. Beyer, N. Christiansen, B. Kamlage, C. Pilarsky, M. Distler, T. Fahlbusch, A. Chromik, F. Klein, M. Bahra, W. Uhl, R. Grü tzmann, U. M. Mahajan, F. U. Weiss, J. Mayerle and M. M. Lerch	2021	Identification and validation of a multivariable prediction model based on blood plasma and serum metabolomics for the distinction of chronic pancreatitis subjects from non-pancreas disease control subjects	Gut	Records excluded after screening

9	Adams, D. L., R. C. Bergan, S. S. Martin, S. Chumsri, M. Charpentier, R. G. Lapidus, R. K. Alpaugh, M. Cristofanilli, S. Tsai, C. M. Tang and M. J. Edelman	2015	Correlation of cancer-associated macrophage-like cells with systemic therapy and pathological stage in numerous malignancies	Journal of Clinical Oncology	Records excluded after screening
10	Aderinto, N., M. O. Abdulbasit, D. Olatunji and M. Edun	2023	Unveiling the potential of galectin-3 as a diagnostic biomarker for pancreatic cancer: A review	Annals of Medicine and Surgery	Records excluded after screening
11	Ahmad, E., A. Ali, Nimisha, A. Kumar Sharma, Apurva, A. Kumar, G. M. Dar, R. Sumayya Abdul Sattar, R. Verma, B. Mahajan and S. Singh Saluja	2022	Molecular markers in cancer	Clinica Chimica Acta	Records excluded after screening
12	Ahmad, S., M. R. Naber, R. H. Giles, G. D. Valk and R. S. van Leeuwaarde	2021	Diagnostic and management strategies for pNETs in Von Hippel-Lindau: a systematic review	Endocr Relat Cancer	Records excluded after screening
13	Ahmadipour, M., A. Bhattacharya, M. Sarafbidabad, E. Syuhada Sazali, S. Krishna Ghoshal, M. Satgunam, R. Singh, M. Rezaei Ardani, N. Missaoui, H. Kahri, U. Pal and A. Ling Pang	2024	CA19-9 and CEA biosensors in pancreatic cancer	Clinica Chimica Acta	Records excluded after screening
14	Ahmadpour, F., M. S. Mousavi Maleki, S. Mahmoodi and H. R. Rasouli	2020	Circulating HSPs Levels and Risk of Human Gastrointestinal Related Cancers: A Systematic Review and Meta-analysis	International Journal of Peptide Research and Therapeutics	Records excluded after screening
15	Ahn, J., K. Jung, J. Lee, B. Kim, J. C. Lee, J. Kim and J. H. Hwang	2023	1640P Response monitoring with ctDNA in metastatic pancreatic cancer	Annals of Oncology	Records excluded after screening
16	Ahn, J., K. Jung, J. Park, J. H. Jung, J. C. Lee, J. Kim and J. H. Hwang	2022	RESPONSE MONITORING WITH CTDNA IN METASTATIC PANCREATIC CANCER	United European Gastroenterology Journal	Records excluded after screening
17	Ahuja, N.	2024	The epigenetic-based liquid biopsy odyssey of bedside pancreatic cancer diagnostics	Cancer Research	Records excluded after screening
18	Ajgaonkar, R., B. Lee, A. Valimukhametova, S. Nguyen, R. Gonzalez-Rodriguez, J. Coffer, G. R. Akkaraju and A. Naumov	2022	Detection of Pancreatic Cancer miRNA with Biocompatible Nitrogen-Doped Graphene Quantum Dots	Materials	Records excluded after screening

19	Akl, E. A., L. A. Kahale, M. B. Hakoum, C. F. Matar, F. Sperati, M. Barba, V. E. D. Yosuico, I. Terrenato, A. Synnot and H. Schünemann	2017	Parenteral anticoagulation in ambulatory patients with cancer	Cochrane Database of Systematic Reviews	Records excluded after screening
20	Alauddin, M. M. and L. De Palatis	2015	Current and future trends in early detection of pancreatic cancer: Molecular targets and PET probes	Current Medicinal Chemistry	Records excluded after screening
21	Ali, S., M. Coory, P. Donovan, R. Na, N. Pandeya, S. A. Pearson, K. Spilsbury, K. Tuesley, S. J. Jordan and R. E. Neale	2024	Predicting the risk of pancreatic cancer in women with new-onset diabetes mellitus	Journal of Gastroenterology and Hepatology (Australia)	Records excluded after screening
22	Alizadeh Savareh, B., H. Asadzadeh Aghdaie, A. Behmanesh, A. Bashiri, A. Sadeghi, M. Zali and R. Shams	2020	A machine learning approach identified a diagnostic model for pancreatic cancer through using circulating microRNA signatures	Pancreatology	Records excluded after screening
23	Al-Shaheri, F. N., M. S. S. Alhamdani, A. S. Bauer, N. Giese, M. W. Büchler, T. Hackert and J. D. Hoheisel	2021	Blood biomarkers for differential diagnosis and early detection of pancreatic cancer	Cancer Treatment Reviews	Records excluded after screening
24	Amaravadi, M., A. S. Bauer, A. Hotz-Wagenblatt, S. K. Botla, M. Löchelt, M. Pawlita, M. W. Büchler, N. Giese and J. D. Hoheisel	2014	Identification of pathogenic virus sequences in pancreatic cancer	European Journal of Cancer	Records excluded after screening
25	Ameli Mojarad, M., M. Ameli Mojarad, B. Shojaee and E. Nazemalhosseini-Mojarad	2022	piRNA: A promising biomarker in early detection of gastrointestinal cancer	Pathol Res Pract	Records excluded after screening
26	Amor, R. E., A. Zinger, Y. Y. Broza, A. Schroeder and H. Haick	2022	Artificially Intelligent Nanoarray Detects Various Cancers by Liquid Biopsy of Volatile Markers	Advanced Healthcare Materials	Records excluded after screening
27	An, Y. R., L. Song, X. Y. Chen, C. M. Ni, K. Z. Mao, L. J. Zhu, Y. Y. Gu, Y. Q. Miao, B. Song and H. Y. Ma	2022	3D Biomimetic Hydrangea-Like BiOCl and PtNi Nanocube-Based Electrochemical Immunosensor for Quantitative Detection of CA19-9	Journal of the Electrochemical Society	Records excluded after screening
28	Anderson, B. W. and D. A. Ahlquist	2016	Molecular Detection of Gastrointestinal Neoplasia: Innovations in Early Detection and Screening	Gastroenterology Clinics of North America	Records excluded after screening

29	Ando, Y., M. Dbouk, T. Yoshida, H. Saba, E. Abou Diwan, K. Yoshida, A. Dbouk, A. L. Blackford, M. T. Lin, A. M. Lennon, R. A. Burkhart, J. He, L. Sokoll, J. R. Eshleman, M. I. Canto and M. Goggins	2024	Using Tumor Marker Gene Variants to Improve the Diagnostic Accuracy of DUPAN-2 and Carbohydrate Antigen 19-9 for Pancreatic Cancer	Journal of clinical oncology : official journal of the American Society of Clinical Oncology	Incomplete diagnostic performance data provided
30	Ansari, D., A. Gustafsson and R. Andersson	2015	Update on the management of pancreatic cancer: Surgery is not enough	World Journal of Gastroenterology	Records excluded after screening
31	Antonini, F., L. Fuccio, C. Fabbri, G. Macarri and L. Palazzo	2015	Management of serous cystic neoplasms of the pancreas	Expert Review of Gastroenterology and Hepatology	Records excluded after screening
32	Aoki, M., H. Shoji, A. Kashiro, K. Takeuchi, Y. Shimizu and K. Honda	2020	Prospects for comprehensive analyses of circulating tumor cells in tumor biology	Cancers	Records excluded after screening
33	Arasawa, T., T. Hiwasa, A. Kagaya, T. Maruyama, M. Uesato, M. Kano, S. Kobayashi, H. Takizawa, K. Iwase, F. Nomura, K. Matsushita and H. Matsubara	2023	Analysis of patients with colorectal cancer shows a specific increase in serum anti-ING1 autoantibody levels	Bmc Cancer	Records excluded after screening
34	Ardila-Molano, J., M. Vizcaíno and M. L. Serrano	2015	Circulating microRNAs as potential cancer biomarkers	Revista Colombiana de Cancerologia	Records excluded after screening
35	Armstrong, A., S. Mirbagheri, U. Barlass, M. R. Haque, D. Z. Gilbert, A. Naqib, J. Amin, A. Singh, H. V. Abdala-Valencia and F. Bishehsari	2021	MULTIPLEX PATIENT-BASED DRUG RESPONSE ASSAY IN PANCREATIC DUCTAL ADENOCARCINOMA	Gastroenterology	Records excluded after screening
36	Arnoletti, P., J. Wang, S. Litherland and X. Han	2018	Lipidomics analysis of alterations in portal vein plasma lipids in pancreatic cancer patients	HPB	Records excluded after screening
37	Aronsson, L., R. Andersson, M. Bauden, B. Andersson, T. Bygott and D. Ansari	2018	High-density and targeted glycoproteomic profiling of serum proteins in pancreatic cancer and intraductal papillary mucinous neoplasm	Scandinavian Journal of Gastroenterology	Studies included in review
38	Asada, T., S. Nakahata, Y. R. Fauzi, T. Ichikawa, K. Inoue, N. Shibata, Y. Fujii, N. Imamura, M. Hiyoshi, A. Nanashima and K. Morishita	2022	Integrin α6A (ITGA6A)-type Splice Variant in Extracellular Vesicles Has a Potential as a Novel Marker of the Early Recurrence of Pancreatic Cancer	Anticancer Research	Records excluded after screening

39	Asai, A., M. Konno, M. Ozaki, K. Kawamoto, R. Chijimatsu, N. Kondo, T. Hirotsu and H. Ishii	2021	Scent test using caenorhabditis elegans to screen for early-stage pancreatic cancer	Oncotarget	Records excluded after screening
40	Asai, Y., T. Itoi, M. Sugimoto, A. Sofuni, T. Tsuchiya, R. Tanaka, R. Tonozuka, M. Honjo, S. Mukai, M. Fujita, K. Yamamoto, Y. Matsunami, T. Kurosawa, Y. Nagakawa, M. Kaneko, S. Ota, S. Kawachi, M. Shimazu, T. Soga, M. Tomita and M. Sunamura	2018	Elevated polyamines in saliva of pancreatic cancer	Cancers	Non-diagnostic studies
41	Asar, M. and M. Soendergaard	2019	Phage Display Selection and Identification of Novel Pancreatic Cancer Targeting Peptides	FASEB Journal	Records excluded after screening
42	Ashkar, M. and T. B. Gardner	2014	Role of endoscopic ultrasound in pancreatic diseases: A systematic review	Minerva Gastroenterologica e Dietologica	Records excluded after screening
43	Azangou-Khyavy, M., M. Ghasemi, J. Khanali, M. Boroomand-Saboor, M. Jamalkhah, M. Soleimani and J. Kiani	2020	CRISPR/Cas: From Tumor Gene Editing to T Cell-Based Immunotherapy of Cancer	Frontiers in Immunology	Records excluded after screening
44	Bahado-Singh, R. O., O. Turkoglu, B. Aydas and S. Vishweswaraiah	2023	Precision oncology: Artificial intelligence, circulating cell-free DNA, and the minimally invasive detection of pancreatic cancer-A pilot study	Cancer Med	Records excluded after screening
45	Bailleux, C., L. Lacroix, E. Barranger and S. Delaloge	2020	Using methylation signatures on cell-free DNA for early cancer detection: a new era in liquid biopsy?	Annals of Oncology	Records excluded after screening
46	Baker, M. J., J. M. Cameron, A. Sala, G. Antoniou, J. J. A. Conn, R. G. McHardy and D. S. Palmer	2023	1216P A spectroscopic liquid biopsy for the earlier detection of multiple cancer types	Annals of Oncology	Records excluded after screening
47	Balasenthil, S., Y. Huang, S. Liu, T. Marsh, J. Chen, S. A. Stass, D. Ku Kuruga, R. Brand, N. Chen, M. L. Frazier, J. J. Lee, S. Srivastava, S. Sen and A. M. Killary	2019	Migration signature biomarker panel improves gold standard CA 19-9 performance for detection of early stage pancreatic cancer in the blood	Pancreas	Records excluded after screening

48	Balasenthil, S., Y. Huang, S. Liu, T. Marsh, J. Chen, S. A. Stass, D. KuKuruga, R. Brand, N. Chen, M. L. Frazier, J. Jack Lee, S. Srivastava, S. Sen and A. McNeill Killary	2017	A Plasma Biomarker Panel to Identify Surgically Resectable Early-Stage Pancreatic Cancer	J Natl Cancer Inst	Records excluded after screening
49	Bam, R., I. Daryaei, L. Abou-Elkacem, J. G. Vilches-Moure, E. J. Meuillet, A. Lutz, E. R. Marinelli, E. C. Unger, S. S. Gambhir and R. Paulmurugan	2020	Toward the Clinical Development and Validation of a Thy1-Targeted Ultrasound Contrast Agent for the Early Detection of Pancreatic Ductal Adenocarcinoma	Investigative Radiology	Records excluded after screening
50	Banaei, N., A. Foley, J. M. Houghton, Y. Sun and B. Kim	2017	Multiplex detection of pancreatic cancer biomarkers using a SERS-based immunoassay	Nanotechnology	Records excluded after screening
51	Banaei, N., J. Moshfegh and B. Kim	2021	Surface enhanced Raman spectroscopy-based immunoassay detection of tumor-derived extracellular vesicles to differentiate pancreatic cancers from chronic pancreatitis	Journal of Raman Spectroscopy	Records excluded after screening
52	Banaei, N., J. Moshfegh, A. Mohseni-Kabir, J. M. Houghton, Y. B. Sun and B. Kim	2019	Machine learning algorithms enhance the specificity of cancer biomarker detection using SERS-based immunoassays in microfluidic chips	Rsc Advances	Records excluded after screening
53	Bansal, S., S. Wang, Y. Li, S. Bansal, J. Smith, J. B. Tyburski, K. Unger and A. Cheema	2024	Plasma EV profiling facilitates low abundance biomarker discovery in pancreatic cancer	Cancer Research	Records excluded after screening
54	Bantis, L. E. and J. V. Tsimikas	2022	On optimal biomarker cutoffs accounting for misclassification costs in diagnostic trilemmas with applications to pancreatic cancer	Statistics in Medicine	Records excluded after screening
55	Bantis, L. E., Q. X. Yan, J. V. Tsimikas and Z. D. Feng	2017	Estimation of smooth ROC curves for biomarkers with limits of detection	Statistics in Medicine	Records excluded after screening
56	Bartsch, D. K., N. Gercke, K. Strauch, R. Wieboldt, E. Matthäi, V. Wagner, S. Rospleszcz, A. Schä fer, F. S. Franke, I. Mintziras, C. Bauer, T. Grote, J. Figiel, P. Di Fazio, A. Burchert, S. Reinartz, E. P. von Strandmann, G. Klö ppel and E. P. Slater	2018	The Combination of MiRNA-196b, LCN2, and TIMP1 is a Potential Set of Circulating Biomarkers for Screening Individuals at Risk for Familial Pancreatic Cancer	Journal of Clinical Medicine	Not early-stage

57	Bashi, A. C., E. A. Coker, K. C. Bulusu, P. Jaaks, C. Crafter, H. Lightfoot, M. Milo, K. McCarten, D. F. Jenkins, D. van der Meer, J. T. Lynch, S. Barthorpe, C. L. Andersen, S. T. Barry, A. Beck, J. Cidado, J. A. Gordon, C. Hall, J. Hall, I. Mali, T. Mironenko, K. Mongeon, J. Morris, L. Richardson, P. D. Smith, O. Tavana, C. Tolley, F. Thomas, B. S. Willis, W. Yang, M. J. O'connor, U. McDermott, S. E. Critchlow, L. Drew, S. E. Fawell, J. T. Mettetal and M. J. Garnett	2024	Large-scale Pan-cancer Cell Line Screening Identifies Actionable and Effective Drug Combinations	Cancer Discovery	Records excluded after screening
58	Batista, L., L. Ruiz, C. Ferrer, Y. Zabana, M. Aceituno, B. Arau, X. Andújar, M. Esteve and F. Fernández-Bañares	2019	Usefulness of fecal calprotectin as a biomarker of microscopic colitis in a cohort of patients with chronic watery diarrhoea of functional characteristics	Digestive and Liver Disease	Records excluded after screening
59	Bauden, M., D. Pamart, D. Ansari, M. Herzog, M. Eccleston, J. Micallef, B. Andersson and R. Andersson	2015	Circulating nucleosomes as epigenetic biomarkers in pancreatic cancer	Clinical Epigenetics	Studies included in review
60	Bazarbashi, S., W. P. Su, S. W. Wong, R. A. Singarachari, S. Rawal, M. I. Volkova and D. A. Bastos	2021	A Narrative Review of Implementing Precision Oncology in Metastatic Castration-Resistant Prostate Cancer in Emerging Countries	Oncology and Therapy	Records excluded after screening
61	Becker, A. E., Y. G. Hernandez, H. Frucht and A. L. Lucas	2014	Pancreatic ductal adenocarcinoma: risk factors, screening, and early detection	World J Gastroenterol	Records excluded after screening
62	Beeler, K., R. Bruderer, M. Tognetti, K. Sklodowski, S. Mueller, D. Kamber and L. Reiter	2021	Unbiased proteomic profiling leads to the discovery of a novel non-invasive blood-based protein panel with significant positive predictive value in pancreatic and colorectal cancers	Journal for ImmunoTherapy of Cancer	Records excluded after screening
63	Belotti, D., A. Resovi, G. Taraboletti, R. T. Lawlor, A. Scarpa and R. Giavazzi	2015	Circulating stroma-related molecules as potential biomarkers for pancreatic ductal adenocarcinoma	Cancer Research	Records excluded after screening
64	Benati, M., M. Montagnana, E. Danese, E. Paviati, S. Giudici, O. Ruzzenente, M. Franchi and G. Lippi	2018	The clinical significance of DJ-1 and HE4 in patients with endometrial cancer	Journal of Clinical Laboratory Analysis	Records excluded after screening

65	Bennett, R. L., D. C. Monagle, A. Sobh, C. Vakoc, K. S. Smalley, J. W. Harbour and J. D. Licht	2021	Identification of essential proliferation and trametinib resistance mechanisms in GNAQ-mutant uveal melanoma	Cancer Research	Records excluded after screening
66	Bergamaschi, A., D. Haan, M. Collins, G. Guler, M. Peters, L. Gigliotti, S. Chowdhury, W. Volkmuth and S. Levy	2023	Early detection of pancreatic cancer using 5-hydroxymethylation profiles in plasmaderived cell-free DNA	Journal of Clinical Oncology	Records excluded after screening
67	Bergamaschi, A., D. Haan, V. Friedl, G. Guler, M. Collins, T. Phillips, M. Antoine, M. Peters, S. Chowdhury, W. Volkmuth and S. Levy	2024	Comparison Between Avantect, a Blood-based Epigenomic Pancreatic Cancer Test, and the CA19-9 Biomarker	Pancreas	Records excluded after screening
68	Bergamaschi, A., D. Haan, J. Ku, Y. Ning, C. Ellison, G. Guler, T. Phillips, E. McCarthy, M. Antoine, A. Nguyen, A. Scott, P. Lloyd, A. Ashworth, K. Bethel and S. Levy	2020	Effect of detection of epigenomic changes in plasma-derived cellfree DNA on multicancer classification	Journal of Clinical Oncology	Records excluded after screening
69	Berghmans, E., K. Boonen, E. Maes, I. Mertens, P. Pauwels and G. Baggerman	2020	Implementation of Maldi mass spectrometry imaging in cancer proteomics research: Applications and challenges	Journal of Personalized Medicine	Records excluded after screening
70	Berry, W., E. Algar, B. Kumar, C. Desmond, M. Swan, B. J. Jenkins and D. Croagh	2017	Endoscopic ultrasound-guided fine-needle aspirate-derived preclinical pancreatic cancer models reveal panitumumab sensitivity in KRAS wild-type tumors	Int J Cancer	Records excluded after screening
71	Best, L. M. J., V. Rawji, S. P. Pereira, B. R. Davidson and K. S. Gurusamy	2017	Imaging modalities for characterising focal pancreatic lesions	Cochrane Database of Systematic Reviews	Records excluded after screening
72	Best, L. M. J., Y. Takwoingi, S. Siddique, A. Selladurai, A. Gandhi, B. Low, M. Yaghoobi and K. S. Gurusamy	2018	Non-invasive diagnostic tests for Helicobacter pylori infection	Cochrane Database of Systematic Reviews	Records excluded after screening
73	Bestari, M. B., I. R. Joewono and A. F. Syam	2024	A Quest for Survival: A Review of the Early Biomarkers of Pancreatic Cancer and the Most Effective Approaches at Present	Biomolecules	Records excluded after screening
74	Bhasin, M., O. Bucur, K. Ndebele, J. Plati, A. Bullock, X. Gu, E. Castan, R. Najarian, J. J. Yeh, C. Der, J. C. Haines, K. Ruping, R. Miksad, R. Khosravi-Far and T. A. Libermann	2015	Transcriptome meta-analysis identifies new 5-gene classifier for early detection of pancreatic cancer	Cancer Research	Records excluded after screening

75	Bhasin, M., A. Bullock, X. Gu, O. Bucur, R. Najarian, J. Cody Haines, K. Ruping, R. Miksad, T. Libermann and R. Khosravi-Far	2014	Early detection of pancreatic cancer using a new 5-gene classifier	Cancer Research	Records excluded after screening
76	Bhasin, M. K., K. Ndebele, O. Bucur, E. U. Yee, H. H. Otu, J. Plati, A. Bullock, X. Gu, E. Castan, P. Zhang, R. Najarian, M. S. Muraru, R. Miksad, R. Khosravi-Far and T. A. Libermann	2016	Meta-analysis of transcriptome data identifies a novel 5-gene pancreatic adenocarcinoma classifier	Oncotarget	Records excluded after screening
77	Bhutani, M., P. Koduru, G. Lanke, M. Bruno, A. Maitra and M. Giovannini	2015	The emerging role of endoscopic ultrasound-guided core biopsy for the evaluation of solid pancreatic masses	Minerva Gastroenterol Dietol	Records excluded after screening
78	Bhutani, M. S., P. Koduru, V. Joshi, P. Saxena, R. Suzuki, A. Irisawa and K. Yamao	2016	The role of endoscopic ultrasound in pancreatic cancer screening	Endoscopic Ultrasound	Records excluded after screening
79	Bian, F., L. Sun, L. Cai, Y. Wang, Y. Zhao, S. Wang and M. Zhou	2019	Molybdenum disulfide-integrated photonic barcodes for tumor markers screening	Biosensors and Bioelectronics	Records excluded after screening
80	Bijlsma, M. F., P. C. Strasser, M. C. Schneider and N. Thumfart	2024	A novel early cancer detection approach for pancreatic cancer	Journal of Clinical Oncology	Records excluded after screening
81	Bingham, M., I. Bhamra, R. Armer, B. Thompson, S. Woodcock, A. Thomason, C. Phillips, H. McKeever, J. Bradford, B. Chaffey, L. Little and G. Clack	2017	Identification of an RNF43 mutated gastric cancer patient population with potential sensitivity to porcupine inhibitor RXC004 and development of a complimentary ctDNA liquid biopsy assay for patient screening	Annals of Oncology	Records excluded after screening

82	Blume, J., G. Bundalian, J. Chan, C. Chao-Shern, J. Choi, R. Cuaresma, K. Dai, S. N. Golmaei, J. Heok Jang, M. Khadka, E. Khaledian, T. Khin, Y. Kodama, A. Kokate, J. Y. Lee, M. Liu, H. Malekpour, M. Mora, N. Mudaliar, P. Prasad, M. Ramaiah, S. Ramaswamy, P. Spiro, K. Swaminathan, D. Vitko, J. Yee, B. Young, S. Zhang, C. Belthangady, B. Wilcox, B. Koh and P. Ma	2023	A multi-omics classifier achieves high sensitivity and specificity for pancreatic ductal adenocarcinoma in a case-control study of 146 subjects	Cancer Research	Records excluded after screening
83	Blyuss, O., S. Duffy and T. Crnogorac-Jurcevic	2019	Development of a biomarker panel risk score, PancRISK, for early detection of pancreatic cancer	Pancreatology	Records excluded after screening
84	Blyuss, O., A. Zaikin, V. Cherepanova, D. Munblit, E. M. Kiseleva, O. M. Prytomanova, S. W. Duffy and T. Crnogorac-Jurcevic	2020	Development of PancRISK, a urine biomarker-based risk score for stratified screening of pancreatic cancer patients	British Journal of Cancer	Not early-stage
85	Boekhoff, H., L. Hendricks, E. Cairo, A. Bauer, M. Hambardzumyan, M. W. Büchler, S. Calderazzo, V. Weru, A. Kopp-Schneider, H. Brenner, T. Hackert, O. Strobel, T. Waterboer, N. Giese and J. D. Hoheisel	2022	Profiling of circulating autoreactive antibodies in some 1200 patients with pancreatic ductal adenocarcinoma and progressive precursor lesions	Cancer Research	Records excluded after screening
86	Boulaiz, H., M. C. Ramos, C. Griñán-Lisón, M. E. García-Rubiño, F. Vicente and J. A. Marchal	2017	What's new in the diagnosis of pancreatic cancer: a patent review (2011-present)	Expert Opin Ther Pat	Records excluded after screening
87	Braig, Z. V.	2022	Personalized medicine: From diagnostic to adaptive	Biomedical Journal	Records excluded after screening
88	Brand, R. E., B. M. Nolen, H. J. Zeh, P. J. Allen, M. A. Eloubeidi, M. Goldberg, E. Elton, J. P. Arnoletti, J. D. Christein, S. M. Vickers and et al.	2011	Serum biomarker panels for the detection of pancreatic cancer	Clinical cancer research	Records excluded after screening

89	Brand, R. E., J. Persson, S. O. Bratlie, D. C. Chung, B. W. Katona, A. Carrato, M. Castillo, J. Earl, A. Kokkola, A. L. Lucas, A. J. Moser, C. Decicco, L. D. Mellby and T. C. King	2022	Detection of Early-Stage Pancreatic Ductal Adenocarcinoma from Blood Samples: Results of a Multiplex Biomarker Signature Validation Study	Clinical and Translational Gastroenterology	Studies included in review
90	Brezgyte, G., V. Shah, D. Jach and T. Crnogorac-jurcevic	2021	Non-invasive biomarkers for earlier detection of pancreatic cancer—a comprehensive review	Cancers	Records excluded after screening
91	Brito-Rocha, T., V. Constâncio, R. Henrique and C. Jeró nimo	2023	Shifting the Cancer Screening Paradigm: The Rising Potential of Blood-Based Multi-Cancer Early Detection Tests	Cells	Records excluded after screening
92	Brunet, L. R., J. Crooks, A. MacDonald, S. LaBrie and D. Eisinger	2017	The immunomodulatory effects of cancer therapy on IFN-gamma responses in the periphery	Journal for ImmunoTherapy of Cancer	Records excluded after screening
93	Bryce, C. and M. Bucaj	2024	Pancreatic Cancer: Rapid Evidence Review	American Family Physician	Records excluded after screening
94	Brychta, N., T. Krahn and O. Von Ahsen	2016	Detection of KRAS mutations in circulating tumor DNA by digital PCR in early stages of pancreatic cancer	Clinical Chemistry	Records excluded after screening
95	Bunganič, B., L. š ovičková, M. Tatarkovič, L. Kocourková, š. Suchá nek, P. Frič, V. Setnička and M. Zavoral	2015	Molecular spectroscopy of blood plasma - Towards the diagnostics of pancreatic cancer?	Gastroenterologi e a Hepatologie	Records excluded after screening
96	Bunger, S., S. Freitag-Wolf, T. Gemoll, M. Kelly, A. Chacko, P. Lowry, S. Fitzgerald, H. Bruch, U. Roblick, T. Keck and J. Habermann	2015	A multiplex serum biochip allows screening for pancreatic cancer at early tumor stages	Clinical Chemistry and Laboratory Medicine	Records excluded after screening
97	Burki, T. K.	2018	Protein-metabolite panel for early-stage pancreatic cancer	Lancet Oncol	Records excluded after screening
98	Buscail, E., C. Caumont, C. Alix-Panabières, J. P. Merlio, A. Bedel, F. Moreau-Gaudry, V. Vendrely, C. Laurent, S. Dabernat and L. Chiche	2018	Comparative analysis of circulating tumour cells detection and circulating tumor DNA in liquid biopsy for the diagnosis of early stage pancreatic adenocarcinoma	HPB	Records excluded after screening
99	Calabrese, F., P. Carrera, S. Benedetti, S. Merella, G. Pipitone, C. Diresta, M. G. Patricelli, A. Russo Raucci, R. Rosati, M. Reni, M. Ronzoni, P. A. Testoni, M. Ferrari and G. M. Cavestro	2017	An interdisciplinary model in identification of GI syndromic cancers using NGS	Digestive and Liver Disease	Records excluded after screening

100	Camblin, A. J., E. A. Pace, S. Adams, M. D. Curley, V. Rimkunas, L. Nie, G. Tan, T. Bloom, S. Iadevaia, J. Baum, C. Minx, A. Czibere, C. U. Louis, D. C. Drummond, U. B. Nielsen, B. Schoeberl, J. M. Pipas, R. M. Straubinger, V. Askoxylakis and A. A. Lugovskoy	2018	Dual Inhibition of IGF-1R and ErbB3 Enhances the Activity of Gemcitabine and Nab-Paclitaxel in Preclinical Models of Pancreatic Cancer	Clin Cancer Res	Records excluded after screening
101	Cao, F., A. Wei, X. Hu, Y. He, J. Zhang, L. Xia, K. Tu, J. Yuan, Z. Guo, H. Liu, D. Xie and A. Li	2020	Integrated epigenetic biomarkers in circulating cell-free DNA as a robust classifier for pancreatic cancer	Clinical Epigenetics	Records excluded after screening
102	Cao, J., C. Xia, T. Cui, H. Guo, H. Li, Y. Ren and S. Wang	2015	Correlations between serum trypsinogen-2 and pancreatic cancer	Hepatogastroenterology	Records excluded after screening
103	Cao, L., C. Huang, D. Cui Zhou, Y. Hu, T. M. Lih, S. R. Savage, K. Krug, D. J. Clark, M. Schnaubelt, L. Chen, F. da Veiga Leprevost, R. V. Eguez, W. Yang, J. Pan, B. Wen, Y. Dou, W. Jiang, Y. Liao, Z. Shi, N. V. Terekhanova, S. Cao, R. J. Lu, Y. Li, R. Liu, H. Zhu, P. Ronning, Y. Wu, M. A. Wyczalkowski, H. Easwaran, L. Danilova, A. S. Mer, S. Yoo, J. M. Wang, W. Liu, B. Haibe-Kains, M. Thiagarajan, S. D. Jewell, G. Hostetter, C. J. Newton, Q. K. Li, M. H. Roehrl, D. Fenyö, P. Wang, A. I. Nesvizhskii, D. R. Mani, G. S. Omenn, E. S. Boja, M. Mesri, A. I. Robles, H. Rodriguez, O. F. Bathe, D. W. Chan, R. H. Hruban, L.	2021	Proteogenomic characterization of pancreatic ductal adenocarcinoma	Cell	Records excluded after screening
104	Cao, Y., R. Zhao, K. Guo, S. Ren, Y. Zhang, Z. Lu, L. Tian, T. Li, X. Chen and Z. Wang	2022	Potential Metabolite Biomarkers for Early Detection of Stage-I Pancreatic Ductal Adenocarcinoma	Frontiers in Oncology	Small sample size

105	Cappello, F., M. Logozzi, C. Campanella, C. C. Bavisotto, A. Marcilla, F. Properzi and S. Fais	2017	Exosome levels in human body fluids: A tumor marker by themselves?	European Journal of Pharmaceutical Sciences	Records excluded after screening
106	Caputo, C., M. Falco, A. Grimaldi, A. Lombardi, C. C. Miceli, M. Cocule, M. Montella, L. Pompella, G. Tirino, S. Campione, C. Tammaro, A. Cossu, G. Fenu Pintori, M. Maioli, D. Coradduzza, G. Savarese, A. Fico, A. Ottaiano, G. Conzo, M. S. Tathode, F. Ciardiello, M. Caraglia, F. De Vita and G. Misso	2024	Identification of Tissue miRNA Signatures for Pancreatic Ductal Adenocarcinoma	Cancers	Not early-stage
107	Caputo, D. and G. Caracciolo	2020	Nanoparticle-enabled blood tests for early detection of pancreatic ductal adenocarcinoma	Cancer Letters	Records excluded after screening
108	Caputo, D., M. Cartillone, C. Cascone, D. Pozzi, L. Digiacomo, S. Palchetti, G. Caracciolo and R. Coppola	2018	Improving the accuracy of pancreatic cancer clinical staging by exploitation of nanoparticle-blood interactions: A pilot study	Pancreatology	Records excluded after screening
109	Caputo, D., A. Coppola, E. Quagliarini, R. Di Santo, A. L. Capriotti, R. Cammarata, A. Laganà, M. Papi, L. Digiacomo, R. Coppola, D. Pozzi and G. Caracciolo	2022	Multiplexed Detection of Pancreatic Cancer by Combining a Nanoparticle-Enabled Blood Test and Plasma Levels of Acute-Phase Proteins	Cancers	Records excluded after screening
110	Caputo, D., L. Digiacomo, C. Cascone, D. Pozzi, S. Palchetti, R. Di Santo, E. Quagliarini, R. Coppola, M. Mahmoudi and G. Caracciolo	2021	Synergistic analysis of protein corona and haemoglobin levels detects pancreatic cancer	Cancers	Records excluded after screening
111	Caputo, D., D. Pozzi, T. Farolfi, R. Passa, R. Coppola and G. Caracciolo	2021	Nanotechnology and pancreatic cancer management: State of the art and further perspectives	World Journal of Gastrointestinal Oncology	Records excluded after screening
112	Caputo, D., E. Quagliarini, A. Coppola, V. La Vaccara, B. Marmiroli, B. Sartori, G. Caracciolo and D. Pozzi	2023	Inflammatory biomarkers and nanotechnology: new insights in pancreatic cancer early detection	International Journal of Surgery	Not early-stage

113	Caputo, D. D., C. Cascone, D. Pozzi, L. Digiacomo, S. Palchetti, G. Caracciolo and R. Coppola	2017	Exploitation of nanoparticle-blood interaction for biomarker discovery in pancreatic cancer	Journal of the American College of Surgeons	Records excluded after screening
114	Carnevale, I., S. Coppola, D. Deng, N. Funel, T. Schmidt, G. Kazemier, E. Zaura and E. Giovannetti	2018	Development of a fluorescence in situ hybridization (FISH) method for detection of intra-tumour bacteria involved in pancreatic cancer chemoresistance	ESMO Open	Records excluded after screening
115	Carotenuto, P., F. Amato, A. Lampis, C. Rae, S. Hedayat, M. C. Previdi, D. Zito, M. Raj, V. Guzzardo, F. Sclafani, A. Lanese, C. Parisi, C. Vicentini, I. Said-Huntingford, J. C. Hahne, A. Hallsworth, V. Kirkin, K. Young, R. Begum, A. Wotherspoon, K. Kouvelakis, S. X. Azevedo, V. Michalarea, R. Upstill-Goddard, S. Rao, D. Watkins, N. Starling, A. Sadanandam, D. K. Chang, A. V. Biankin, N. B. Jamieson, A. Scarpa, D. Cunningham, I. Chau, P. Workman, M. Fassan, N. Valeri and C. Braconi	2021	Modulation of pancreatic cancer cell sensitivity to FOLFIRINOX through microRNA-mediated regulation of DNA damage	Nature Communications	Records excluded after screening
116	Carotenuto, P., D. Zito, M. C. Previdi, M. Raj, M. Fassan, A. Lampis, F. Scalafani, A. Lanese, I. Said-Huntingford, J. C. Hahne, K. Young, R. Begum, Z. Ethiar, A. Wotherspoon, N. Starling, A. Sadanandam, D. Cunningham, I. Chau, P. Workman, R. Chopra, N. Valeri and C. Braconi	2018	MIR1307 mediates pancreatic cancer resistance to FOLFIRINOX chemotherapy by affecting response to DNA damage	Cancer Research	Records excluded after screening
117	Carr, R. A., M. T. Yip-Schneider, S. Dolejs, B. A. Hancock, H. Wu, M. Radovich and C. M. Schmidt	2017	Pancreatic Cyst Fluid Vascular Endothelial Growth Factor A and Carcinoembryonic Antigen: A Highly Accurate Test for the Diagnosis of Serous Cystic Neoplasm	Journal of the American College of Surgeons	Non-diagnostic studies

118	Carr, R. A., M. T. Yip-Schneider, R. E. Simpson, S. Dolejs, J. G. Schneider, H. Wu, E. P. Ceppa, W. Park and C. M. Schmidt	2018	Pancreatic cyst fluid glucose: rapid, inexpensive, and accurate diagnosis of mucinous pancreatic cysts	Surgery (United States)	Not early-stage
119	Carter, J. V., H. L. Roberts, J. Pan, J. D. Rice, J. F. Burton, N. J. Galbraith, M. R. Eichenberger, J. Jorden, P. Deveaux, R. Farmer, A. Williford, Z. Kanaan, S. N. Rai and S. Galandiuk	2016	A highly predictive model for diagnosis of colorectal neoplasms using plasma MicroRNA: Improving specificity and sensitivity	Annals of Surgery	Non-diagnostic studies
120	Cayssials, V., G. Buckland, M. Crous-Bou, C. Bonet, E. Weiderpass, G. Skie, D. Aune, A. Heath, T. H. Nøst, G. Masala, C. Agnoli, M. S. De Magistris, B. Bueno-de-Mesquita, J. Derksen, I. Huybrechts, P. Ferrari, O. Franklin, S. Bodén, M. Schulze, J. M. Huerta, A. Barricarte, C. Sacerdote, P. Amiano, R. Tumino, E. Molina-Montes, A. Tjønneland, C. Kyrø, G. Severi, M. C. Boutron-Ruault, V. Rebours, V. Katzke, A. Agudo and P. Jakszyn	2022	Inflammatory potential of diet and pancreatic cancer risk in the EPIC study	European Journal of Nutrition	Records excluded after screening
121	Chambers, A. G., A. J. Percy, R. Simon and C. H. Borchers	2014	MRM for the verification of cancer biomarker proteins: Recent applications to human plasma and serum	Expert Review of Proteomics	Records excluded after screening
122	Champanhac, C., I. T. Teng, S. Cansiz, L. Zhang, X. Wu, Z. Zhoa, T. Fu and W. Tan	2015	Development of a panel of DNA Aptamers with High Affinity for Pancreatic Ductal Adenocarcinoma	Scientific reports	Records excluded after screening
123	Chan, A., I. Prassas, A. Dimitromanolakis, R. E. Brand, S. Serra, E. P. Diamandis and I. M. Blasutig	2014	Validation of biomarkers that complement CA19.9 in detecting early pancreatic cancer	Clin Cancer Res	Studies excluded after qualitative synthesis
124	Chang, J. C. and M. Kundranda	2017	Novel diagnostic and predictive biomarkers in pancreatic adenocarcinoma	International Journal of Molecular Sciences	Records excluded after screening

125	Chari, S. T., K. Kelly, M. A. Hollingsworth, S. P. Thayer, D. A. Ahlquist, D. K. Andersen, S. K. Batra, T. A. Brentnall, M. Canto, D. F. Cleeter, M. A. Firpo, S. S. Gambhir, V. L. Go, O. J. Hines, B. J. Kenner, D. S. Klimstra, M. M. Lerch, M. J. Levy, A. Maitra, S. J. Mulvihill, G. M. Petersen, A. D. Rhim, D. M. Simeone, S. Srivastava, M. Tanaka, A. I. Vinik and D. Wong	2015	Early detection of sporadic pancreatic cancer: summative review	Pancreas	Records excluded after screening
126	Chavez-Pineda, O. G., R. Rodriguez-Moncayo, D. F. Cedillo-Alcantar, P. E. Guevara-Pantoja, J. U. Amador-Hernandez and J. L. Garcia-Cordero	2022	Microfluidic systems for the analysis of blood-derived molecular biomarkers	Electrophoresis	Records excluded after screening
127	Chemi, F., S. Mohan, T. Guevara, A. Clipson, D. G. Rothwell and C. Dive	2021	Early Dissemination of Circulating Tumor Cells: Biological and Clinical Insights	Frontiers in Oncology	Records excluded after screening
128	Chen, C., C. Q. Wu, T. W. Chen, M. Y. Tang and X. M. Zhang	2015	Molecular Imaging with MRI: Potential Application in Pancreatic Cancer	Biomed Res Int	Records excluded after screening
129	Chen, F., Y. Guo and L. Wang	2015	The Emerging Genetic Basis and Its Clinical Implication in Pancreatic Cancer	Gastrointestinal Tumors	Records excluded after screening
130	Chen, G., Y. Liu, D. Su, J. Qiu, J. Long, F. Zhao, J. Tao, G. Yang, H. Huang, J. Xiao, T. Zhang and Y. Zhao	2023	Genomic analysis and filtration of novel prognostic biomarkers based on metabolic and immune subtypes in pancreatic cancer	Cellular Oncology	Records excluded after screening
131	Chen, H., J. Zhang, X. Sun, Y. Wang and Y. Qian	2022	Mitophagy-mediated molecular subtypes depict the hallmarks of the tumour metabolism and guide precision chemotherapy in pancreatic adenocarcinoma	Frontiers in Cell and Developmental Biology	Records excluded after screening
132	Chen, J., H. Li, W. Xu and X. Guo	2021	Evaluation of serum ATX and LPA as potential diagnostic biomarkers in patients with pancreatic cancer	BMC Gastroenterology	Incomplete diagnostic performance data provided
133	Chen, J., H. Wang, L. Zhou, Z. Liu and X. Tan	2022	A combination of circulating tumor cells and CA199 improves the diagnosis of pancreatic cancer	Journal of Clinical Laboratory Analysis	No sample information provided
134	Chen, J., X. X. Zhu, H. Xu, H. Z. Fang and J. Q. Zhao	2016	Expression and prognostic significance of unique ULBPs in pancreatic cancer	OncoTargets and Therapy	Records excluded after screening

135	Chen, J. W. and J. Dhahbi	2022	Identification of four serum miRNAs as potential markers to screen for thirteen cancer types	PLoS ONE	Records excluded after screening
136	Chen, K., Q. Wang, M. Kornmann, X. Tian and Y. Yang	2021	The Role of Exosomes in Pancreatic Cancer From Bench to Clinical Application: An Updated Review	Frontiers in Oncology	Records excluded after screening
137	Chen, Q., D. R. Cherry, V. Nalawade, E. M. Qiao, A. Kumar, A. M. Lowy, D. R. Simpson and J. D. Murphy	2021	Clinical Data Prediction Model to Identify Patients With Early-Stage Pancreatic Cancer	Jco Clinical Cancer Informatics	Records excluded after screening
138	Chen, Q., D. Yu, Y. Zhao, J. Qiu, Y. Xie and M. Tao	2019	Screening and identification of hub genes in pancreatic cancer by integrated bioinformatics analysis	Journal of Cellular Biochemistry	Records excluded after screening
139	Chen, W., Y. Zhou, V. Asadpour, R. Parker, E. Lustigova, E. Puttock and B. U. Wu	2021	Quantitative Imaging Features on Pre-diagnostic Computed Tomography Can Accurately Predict Pancreatic Cancer	Pancreas	Records excluded after screening
140	Chen, W. C. Y., B. Boursi, R. Mamtani and Y. X. Yang	2019	Total Serum Cholesterol and Pancreatic Cancer: A Nested Case-Control Study	Cancer Epidemiology Biomarkers & Prevention	Not early-stage
141	Chen, W. S., Y. C. Zhou, V. Asadpour, R. A. Parker, E. J. Puttock, E. Lustigova and B. U. Wu	2023	Quantitative Radiomic Features From Computed Tomography Can Predict Pancreatic Cancer up to 36 Months Before Diagnosis	Clinical and Translational Gastroenterology	No specific biomarkers provided
142	Chen, X., X. Liao, B. Zheng, F. Wang, F. Chen, Z. Deng, H. Jiang and S. Qin	2023	Differential Plasma Proteins Identified via iTRAQ-Based Analysis Serve as Diagnostic Markers of Pancreatic Ductal Adenocarcinoma	Disease Markers	Records excluded after screening
143	Chen, Y., W. He, M. Chen, L. Zhao, J. Shao, S. Lin, B. Li, H. Xu, R. Li, J. Feng, N. Liang, Y. Meng, X. Cheng, W. Zhang, H. Yu and R. Xu	2023	MiR-92a expression profiling of exfoliated colonocytes isolated from feces for colorectal cancer screening	American Journal of Translational Research	Records excluded after screening
144	Chen, Y., Z. Ye, M. Ma, J. Yang, R. Liu, Y. Zhang, P. Ma and D. Song	2024	Electrochemiluminescence biosensor for specific detection of pancreatic ductal carcinoma through dual targeting of MUC1 and miRNA-196a	Biosensors and Bioelectronics	Records excluded after screening
145	Chen, Y. Z., D. Liu, Y. X. Zhao, H. T. Wang, Y. Gao and Y. Chen	2014	Diagnostic performance of serum macrophage inhibitory cytokine-1 in pancreatic cancer: A meta-analysis and meta-regression analysis	DNA and Cell Biology	Records excluded after screening
146	Chew, C., S. L. Chan, E. S. H. Cheow, A. W. C. Kow, K. Madhavan, S. G. Iyer, C. E. Chee and G. K. Bonney	2021	Improving accessibility to pancreatic cancer with circulating tumour cell technologies for targeted molecular therapeutics	HPB	Records excluded after screening
147	Chew, C., E. Cheow, S. L. Chan, A. W. C. Kow, S. Iyer, K. Madhavan, E. Chow, C. E. Chee, L. Ho and G. K. Bonney	2022	A Precision Clinical-Omics Approach in Predicting Therapeutic Response to Pancreatic Cancer	HPB	Records excluded after screening

148	Chhoda, A., A. Sharma, B. Sailo, H. Tang, N. Ruzgar, W. Y. Tan, L. Ying, R. Khatri, A. Narayanan, S. Mane, B. De Kumar, L. D. Wood, C. Iacobuzio-Donahue, C. L. Wolfgang, J. W. Kunstman, R. R. Salem, J. J. Farrell and N. Ahuja	2023	Utility of promoter hypermethylation in malignant risk stratification of intraductal papillary mucinous neoplasms	Clinical Epigenetics	Records excluded after screening
149	Chi, H., H. Chen, R. Wang, J. Zhang, L. Jiang, S. Zhang, C. Jiang, J. Huang, X. Quan, Y. Liu, Q. Zhang and G. Yang	2023	Proposing new early detection indicators for pancreatic cancer: Combining machine learning and neural networks for serum miRNA-based diagnostic model	Frontiers in Oncology	Records excluded after screening
150	Choe, J. W., H. J. Kim, J. S. Kim, J. Cha, M. K. Joo, B. J. Lee, J. J. Park and Y. T. Bak	2018	Usefulness of CA 19-9 for pancreatic cancer screening in patients with new-onset diabetes	Hepatobiliary Pancreat Dis Int	Records excluded after screening
151	Choe, J. W., J. S. Kim, H. J. Kim, S. Y. Hwang, M. K. Joo, B. J. Lee, J. H. Kim, J. E. Yeon, J. J. Park, K. S. Byun and Y. T. Bak	2016	Value of Early Check-Up of Carbohydrate Antigen 19-9 Levels for Pancreatic Cancer Screening in Asymptomatic New-Onset Diabetic Patients	Pancreas	Records excluded after screening
152	Choi, H., S. E. Ju, K. S. Kang, M. H. Seo, J. M. Kim, E. Miyoshi, M. K. Yeo and S. Y. Park	2023	Terminal fucosylation of haptoglobin in cancer-derived exosomes during cholangiocarcinoma progression	Frontiers in Oncology	Records excluded after screening
153	Choi, M., M. Park, S. H. Lee, M. J. Lee, Y. K. Paik, S. I. Jang, D. K. Lee, S. G. Lee and C. M. Kang	2023	Development of a metabolite calculator for diagnosis of pancreatic cancer	Cancer Medicine	Records excluded after screening
154	Choi, M. H., E. Mejlænder-Larsen, S. Manueldas, K. El Jellas, S. J. Steine, K. Tjensvoll, H. A. Sætran, S. Knappskog, D. Hoem, O. Nordgard, R. Hovland and A. Molven	2018	Mutation analysis by deep sequencing of pancreatic juice in patients with pancreatic ductal adenocarcinoma	Pancreatology	Small sample size
155	Choi, Y. J., W. Yoon, Y. Han, H. Kim, W. Kwon, A. Lee, J. Y. Jang and T. Park	2021	Pancreatic Cancer Risk Prediction Model Using a Multi-biomarker Panel	HPB	Records excluded after screening

156	Choi, Y. J., W. Yoon, A. Lee, Y. Han, Y. Byun, J. S. Kang, H. Kim, W. Kwon, Y. A. Suh, Y. Kim, S. Lee, J. Namkung, S. Han, Y. Choi, J. S. Heo, J. O. Park, J. K. Park, S. C. Kim, C. M. Kang, W. J. Lee, T. Park and J. Y. Jang	2021	Diagnostic model for pancreatic cancer using a multi-biomarker panel	Annals of Surgical Treatment and Research	Not early-stage
157	Chung, K. H., J. C. Lee, J. Lee, I. K. Cho, J. Kim, W. Jang, B. Chul Yoo and J. H. Hwang	2017	Serum Fibrinogen as a Diagnostic and Prognostic Biomarker for Pancreatic Ductal Adenocarcinoma	Gut and Liver	Records excluded after screening
158	Chung, K. H., J. C. Lee, J. Lee, I. K. Cho, J. Kim, W. Jang, B. C. Yoo and J. H. Hwang	2020	Serum fibrinogen as a diagnostic and prognostic biomarker for pancreatic ductal adenocarcinoma	Pancreatology	Not early-stage
159	Chung, S., P. Vail, A. K. Witkiewicz and E. S. Knudsen	2019	Coordinately Targeting Cell-Cycle Checkpoint Functions in Integrated Models of Pancreatic Cancer	Clin Cancer Res	Records excluded after screening
160	Cohen, J. D., B. Diergaarde, N. Papadopoulos, K. W. Kinzler and R. E. Schoen	2020	Tumor DNA as a cancer biomarker through the lens of colorectal neoplasia A C	Cancer Epidemiology Biomarkers and Prevention	Records excluded after screening
161	Cohen, J. D., A. A. Javed, C. Thoburn, F. Wong, J. Tie, P. Gibbs, C. M. Schmidt, M. T. Yip-Schneider, P. J. Allen, M. Schattner, R. E. Brand, A. D. Singhi, G. M. Petersen, S. M. Hong, S. C. Kim, M. Falconi, C. Doglioni, M. J. Weiss, N. Ahuja, J. He, M. A. Makary, A. Maitra, S. M. Hanash, M. Dal Molin, Y. Wang, L. Li, J. Ptak, L. Dobbyn, J. Schaefer, N. Silliman, M. Popoli, M. G. Goggins, R. H. Hruban, C. L. Wolfgang, A. P. Klein, C. Tomasetti, N. Papadopoulos, K. W. Kinzler, B. Vogelstein and A. M. Lennon	2017	Combined circulating tumor DNA and protein biomarker-based liquid biopsy for the earlier detection of pancreatic cancers	Proceedings of the National Academy of Sciences of the United States of America	Studies included in review

162	Cohen, J. D., L. Li, Y. Wang, C. Thoburn, B. Afsari, L. Danilova, C. Douville, A. A. Javed, F. Wong, A. Mattox, R. H. Hruban, C. L. Wolfgang, M. G. Goggins, M. Dal Molin, T. L. Wang, R. Roden, A. P. Klein, J. Ptak, L. Dobbyn, J. Schaefer, N. Silliman, M. Popoli, J. T. Vogelstein, J. D. Browne, R. E. Schoen, R. E. Brand, J. Tie, P. Gibbs, H. L. Wong, A. S. Mansfield, J. Jen, S. M. Hanash, M. Falconi, P. J. Allen, S. Zhou, C. Bettegowda, L. A. Diaz, Jr., C. Tomasetti, K. W. Kinzler, B. Vogelstein, A. M. Lennon and N. Papadopoulos	2018	Detection and localization of surgically resectable cancers with a multi-analyte blood test	Science	Records excluded after screening
163	Coker, E. A., P. Jaaks, D. J. Vis, N. Aben, S. Barthorpe, D. Van Der Meer, H. Lightfoot, L. Wessels and M. Garnett	2019	A high-throughput screen to identify effective and translationally-relevant drug combinations for breast, colorectal, and pancreatic cancers	Molecular Cancer Therapeutics	Records excluded after screening
164	Colbert, L. E., A. V. Petrova, S. B. Fisher, B. G. Pantazides, M. Z. Madden, C. W. Hardy, M. D. Warren, Y. Pan, G. P. Nagaraju, E. A. Liu, B. Saka, W. A. Hall, J. W. Shelton, K. Gandhi, R. Pauly, J. Kowalski, D. A. Kooby, B. F. El-Rayes, C. A. Staley, 3rd, N. V. Adsay, W. J. Curran, Jr., J. C. Landry, S. K. Maithel and D. S. Yu	2014	CHD7 expression predicts survival outcomes in patients with resected pancreatic cancer	Cancer Res	Records excluded after screening
165	Connor, A. A. and S. Gallinger	2017	Next generation sequencing of pancreatic ductal adenocarcinoma: right or wrong?	Expert Review of Gastroenterology and Hepatology	Records excluded after screening
166	Conwell, D. L. and D. C. Whitcomb	2016	Advances in pancreatic biomarker measures: A novel approach to an obscure organ	Clinical and Translational Gastroenterology	Records excluded after screening

167	Coolen-Maturi, T.	2017	Predictive inference for best linear combination of biomarkers subject to limits of detection	Stat Med	Records excluded after screening
168	Coronel, E. and I. Waxman	2016	State-of-the-art endoscopic procedures for pancreatic cancer	Future Oncology	Records excluded after screening
169	Cruz-Monserrate, Z., K. Gumpper, V. Pita, P. A. Hart, C. Forsmark, D. C. Whitcomb, D. Yadav, R. T. Waldron, S. Pandol, H. Steen, V. Anani, N. Kanwar, S. S. Vege, S. Appana, L. Li, J. Serrano, J. A. S. Rinaudo, M. Topazian and D. L. Conwell	2021	Biomarkers of Chronic Pancreatitis: A systematic literature review	Pancreatology	Records excluded after screening
170	Cui, J., X. Wang and S. Chen	2022	Ho2O3-TiO2 Nanobelts Electrode for Highly Selective and Sensitive Detection of Cancer miRNAs	Biosensors	Records excluded after screening
171	Cui, M., Y. Hu, Z. Zhang, T. Chen, M. Dai, Q. Xu, J. Guo, T. Zhang, Q. Liao, J. Yu and Y. Zhao	2023	Cyst fluid glycoproteins accurately distinguishing malignancies of pancreatic cystic neoplasm	Signal Transduction and Targeted Therapy	Records excluded after screening
172	D'Amora, P., I. D. C. G. Silva, S. S. Evans, A. J. Nagourney, K. A. Kirby, B. Herrmann, D. Cavalheiro, F. R. Francisco, P. J. Bernard and R. A. Nagourney	2024	Diagnostic and Prognostic Performance of Metabolic Signatures in Pancreatic Ductal Adenocarcinoma: The Clinical Application of Quantitative NextGen Mass Spectrometry	Metabolites	Non-diagnostic studies
173	Daemen, A., D. Peterson, N. Sahu, R. McCord, X. Du, B. Liu, K. Kowanetz, R. Hong, J. Moffat, M. Gao, A. Boudreau, R. Mroue, L. Corson, T. O'Brien, J. Qing, D. Sampath, M. Merchant, R. Yauch, G. Manning, J. Settleman, G. Hatzivassiliou and M. Evangelista	2015	Metabolite profiling stratifies pancreatic ductal adenocarcinomas into subtypes with distinct sensitivities to metabolic inhibitors	Proc Natl Acad Sci U S A	Records excluded after screening
174	Dai, M., S. Chen, X. Teng, K. Chen and W. Cheng	2022	KRAS as a Key Oncogene in the Clinical Precision Diagnosis and Treatment of Pancreatic Cancer	Journal of Cancer	Records excluded after screening
175	Dai, X., W. Pang, Y. Zhou, W. Yao, L. Xia, C. Wang, X. Chen, K. Zen, C. Y. Zhang and Y. Yuan	2016	Altered profile of serum microRNAs in pancreatic cancer-associated new-onset diabetes mellitus	J Diabetes	Records excluded after screening
176	Dai, Y., K. Abbasi, M. DePietro, S. Butler and C. C. Liu	2018	Advanced fabrication of biosensor on detection of Glypican-1 using S-Acetylmercaptosuccinic anhydride (SAMSA) modification of antibody	Sci Rep	Records excluded after screening

177	Das, S. and D. Cardin	2020	Targeting DNA Damage Repair Pathways in Pancreatic Adenocarcinoma	Curr Treat Options Oncol	Records excluded after screening
178	Davalos, V. and M. Esteller	2023	Cancer epigenetics in clinical practice	CA Cancer Journal for Clinicians	Records excluded after screening
179	Davies, A., M. Hidalgo, J. Stebbing, D. Ciznadija, A. Katz and D. Sidransky	2016	Mouse clinical trials of pancreatic cancer: Integration of PDX models with genomics to improve patient outcomes to chemotherapeutics	Annals of Oncology	Records excluded after screening
180	Dbouk, M., T. Abe, C. Koi, Y. Ando, H. Saba, E. Abou Diwan, A. MacGregor-Das, A. L. Blackford, E. Mocci, K. Beierl, A. Dbouk, J. He, R. Burkhart, A. M. Lennon, L. Sokoll, M. I. Canto, J. R. Eshleman and M. Goggins	2023	Diagnostic Performance of a Tumor Marker Gene Test to Personalize Serum CA19-9 Reference Ranges	Clin Cancer Res	Incomplete diagnostic performance data provided
181	De Assis Cirino, M. L., F. S. L. Neto, V. B. Filiputti, P. C. Fadel, D. P. Da Cunha Tirapelli, R. Kemp, A. K. Sankarankutty and J. S. Dos Santos	2019	Combination of the anti-apoptotic and pro-apoptotic microRNAs expression and the anti-apoptotic protein XIAP with serum CA19-9 in the diagnosis of pancreatic adenocarcinoma	FASEB Journal	Records excluded after screening
182	de Figueiredo, A. M., J. C. Glória, Y. O. Chaves, W. L. L. Neves and L. A. M. Mariúba	2022	Diagnostic applications of microsphere-based flow cytometry: A review	Experimental Biology and Medicine	Records excluded after screening
183	De Maio, G., C. Rengucci, W. Zoli and D. Calistri	2014	Circulating and stool nucleic acid analysis for colorectal cancer diagnosis	World Journal of Gastroenterology	Records excluded after screening
184	Deben, C., L. F. Boullosa, F. R. Fortes, E. C. De La Hoz, M. Le Compte, S. Seghers, M. Peeters, S. Vanlanduit, A. Lin, K. K. Dijkstra, P. Van Schil, J. M. H. Hendriks, H. Prenen, G. Roeyen, F. Lardon and E. Smits	2024	Auranofin repurposing for lung and pancreatic cancer: low CA12 expression as a marker of sensitivity in patient-derived organoids, with potentiated efficacy by AKT inhibition	Journal of Experimental and Clinical Cancer Research	Records excluded after screening
185	Debernardi, S., O. Blyuss, D. Rycyk, K. Srivastava, C. Y. Jeon, H. Cai, Q. Cai, X. O. Shu and T. Crnogorac-Jurcevic	2023	Urine biomarkers enable pancreatic cancer detection up to 2 years before diagnosis	International Journal of Cancer	Not early-stage
186	Debernardi, S., D. Jach, G. Brezgyte, O. Blyuss and T. Crnogorac-Jurcevic	2020	A combination of urinary biomarker panel and PancRISK score for earlier detection of pancreatic cancer	Pancreatology	Records excluded after screening

187	Debernardi, S., N. J. Massat, T. P. Radon, A. Sangaralingam, A. Banissi, D. P. Ennis, T. Dowe, C. Chelala, S. P. Pereira, H. M. Kocher, B. D. Young, G. Bond-Smith, R. Hutchins and T. Crnogorac-Jurcevic	2015	Noninvasive urinary miRNA biomarkers for early detection of pancreatic adenocarcinoma	American Journal of Cancer Research	Studies included in review
188	Delgado, J. A., M. A. Ballesteros, M. M. Parera and J. M. Bauç a	2021	Pancreatic Cancer Insights: Optimization of the Diagnostic Capacity of Tumor Biomarkers	Lab Medicine	Records excluded after screening
189	Demyan, L., A. N. Habowski, D. Plenker, D. A. King, O. J. Standring, C. Tsang, L. St Surin, A. Rishi, J. M. Crawford, J. Boyd, S. A. Pasha, H. Patel, Z. Galluzzo, C. Metz, P. K. Gregersen, S. Fox, C. Valente, S. Abadali, S. Matadial-Ragoo, D. K. DePeralta, G. B. Deutsch, J. M. Herman, M. A. Talamini, D. A. Tuveson and M. J. Weiss	2022	Pancreatic Cancer Patient-derived Organoids Can Predict Response to Neoadjuvant Chemotherapy	Annals of Surgery	Records excluded after screening
190	Deng, T., Y. Yuan, C. Zhang, C. Zhang, W. Yao, C. Wang, R. Liu and Y. Ba	2016	Identification of Circulating miR-25 as a Potential Biomarker for Pancreatic Cancer Diagnosis	Cellular Physiology and Biochemistry	Not early-stage
191	Deng, Z., X. Li, Y. Shi, Y. Lu, W. Yao and J. Wang	2020	A Novel Autophagy-Related IncRNAs Signature for Prognostic Prediction and Clinical Value in Patients With Pancreatic Cancer	Frontiers in Cell and Developmental Biology	Records excluded after screening
192	Deutsch, O., Y. Haviv, G. Krief, N. Keshet, R. Westreich, S. M. Stemmer, B. Zaks, S. P. Navat, R. Yanko, O. Lahav, D. J. Aframian and A. Palmon	2020	Possible proteomic biomarkers for the detection of pancreatic cancer in oral fluids	Scientific Reports	Records excluded after screening
193	Dhani, H., J. P. Hinestrosa, J. Izaguirre-Carbonell, H. I. Balcer, R. Kurzrock and P. R. Billings	2023	Case Report: Early detection of pancreatic pre-cancer lesion in multimodal approach with exosome liquid biopsy	Frontiers in Oncology	Records excluded after screening
194	Dhasmana, A., S. Dhasmana, S. Khan, M. M. Yallapu, M. Jaggi and S. C. Chauhan	2023	CEACAM7 an early detection biomarker for pancreatic ductal adenocarcinoma	Cancer Research	Records excluded after screening

195	Dhasmana, A., S. Dhasmana, S. Kotnala, P. Laskar, S. Khan, S. Haque, M. Jaggi, M. M. Yallapu and S. C. Chauhan	2024	CEACAM7 expression contributes to early events of pancreatic cancer	Journal of Advanced Research	Records excluded after screening
196	Dhayat, S. A. and Z. Yang	2020	Impact of circulating tumor DNA in hepatocellular and pancreatic carcinomas	Journal of Cancer Research and Clinical Oncology	Records excluded after screening
197	Di Gangi, I. M., T. Mazza, A. Fontana, M. Copetti, C. Fusilli, A. Ippolito, F. Mattivi, A. Latiano, A. Andriulli, U. Vrhovsek and V. Pazienza	2016	Metabolomic profile in pancreatic cancer patients: a consensus-based approach to identify highly discriminating metabolites	Oncotarget	Non-diagnostic studies
198	Diamandis, E. P.	2014	Present and future of cancer biomarkers	Clinical Chemistry and Laboratory Medicine	Records excluded after screening
199	Diamandis, E. P. and M. Plebani	2016	Glypican-1 as a highly sensitive and specific pancreatic cancer biomarker	Clinical Chemistry and Laboratory Medicine	Records excluded after screening
200	Diaz, P. M., A. Leehans, P. Ravishankar and A. Daily	2023	Multiomic Approaches for Cancer Biomarker Discovery in Liquid Biopsies: Advances and Challenges	Biomarker Insights	Records excluded after screening
201	Digiacomo, L., E. Quagliarini, D. Pozzi, R. Coppola, G. Caracciolo and D. Caputo	2023	Stratifying Risk for Pancreatic Cancer by Multiplexed Blood Test	Cancers	Records excluded after screening
202	Dima, A. C., D. V. Balaban and A. Dima	2021	Diagnostic application of volatile organic compounds as potential biomarkers for detecting digestive neoplasia: A systematic review	Diagnostics	Records excluded after screening
203	Dimastromatteo, J., T. Brentnall and K. A. Kelly	2017	Imaging in pancreatic disease	Nat Rev Gastroenterol Hepatol	Records excluded after screening
204	Ding, Q., X. Kong, W. Zhong and W. Liu	2022	Fecal biomarkers: Non-invasive diagnosis of colorectal cancer	Frontiers in Oncology	Records excluded after screening
205	Dinges, S. S., A. Hohm, L. A. Vandergrift, J. Nowak, P. Habbel, I. A. Kaltashov and L. L. Cheng	2019	Cancer metabolomic markers in urine: evidence, techniques and recommendations	Nature Reviews Urology	Records excluded after screening
206	Dittmar, R. L., S. Liu, M. C. Tai, K. Rajapakshe, Y. Huang, G. Longton, C. DeCapite, M. W. Hurd, P. L. Paris, K. S. Kirkwood, C. Coarfa, A. Maitra, R. E. Brand, A. M. Killary and S. Sen	2021	Plasma miRNA biomarkers in limited volume samples for detection of early-stage pancreatic cancer	Cancer Prevention Research	Not early-stage

207	Dolphus, C., P. Basile, F. Blanchard, D. Sefrioui, M. Paresy, N. Sarafan-Vasseur, P. Michel and J. C. Sabourin	2015	Circulating tumor cells and circulating tumoral DNA in diagnosis of pancreatic tumor	Laboratory Investigation	Records excluded after screening
208	Dong, Z., X. Chen, Z. Cheng, Y. Luo, M. He, T. Chen, Z. Zhang, X. Qian and W. Chen	2022	Differential diagnosis of pancreatic cystic neoplasms through a radiomics-assisted system	Frontiers in Oncology	No specific biomarkers provided
209	Douville, C., J. D. Cohen, J. Ptak, M. Popoli, J. Schaefer, N. Silliman, L. Dobbyn, R. E. Schoen, J. Tie, P. Gibbs, M. Goggins, C. L. Wolfgang, T. L. Wang, I. M. Shih, R. Karchin, A. M. Lennon, R. H. Hruban, C. Tomasetti, C. Bettegowda, K. W. Kinzler, N. Papadopoulos and B. Vogelstein	2020	Assessing aneuploidy with repetitive element sequencing	Proceedings of the National Academy of Sciences of the United States of America	Records excluded after screening
210	Douville, C., K. Lahouel, A. Kuo, H. Grant, B. E. Avigdor, S. D. Curtis, M. Summers, J. D. Cohen, Y. Wang, A. Mattox, J. Dudley, L. Dobbyn, M. Popoli, J. Ptak, N. Nehme, N. Silliman, C. Blair, K. Romans, C. Thoburn, J. Gizzi, R. E. Schoen, J. Tie, P. Gibbs, L. T. Ho-Pham, B. N. H. Tran, T. S. Tran, T. V. Nguyen, M. Goggins, C. L. Wolfgang, T. L. Wang, I. M. Shih, A. M. Lennon, R. H. Hruban, C. Bettegowda, K. W. Kinzler, N. Papadopoulos, B. Vogelstein and C. Tomasetti	2024	Machine learning to detect the SINEs of cancer	Science Translational Medicine	Non-diagnostic studies
211	Douville, C., S. Springer, I. Kinde, J. D. Cohen, R. H. Hruban, A. M. Lennon, N. Papadopoulos, K. W. Kinzler, B. Vogelstein and R. Karchin	2018	Detection of aneuploidy in patients with cancer through amplification of long interspersed nucleotide elements (LINEs)	Proceedings of the National Academy of Sciences of the United States of America	Non-diagnostic studies

212	Drăgan, A. and A. Ş. Drăgan	2024	Novel Insights in Venous Thromboembolism Risk Assessment Methods in Ambulatory Cancer Patients: From the Guidelines to Clinical Practice	Cancers	Records excluded after screening
213	Draus, T., D. Ansari and R. Andersson	2023	Model-based screening for pancreatic cancer in Sweden	Scandinavian Journal of Gastroenterology	Records excluded after screening
214	Du, W., Z. B. Cao, Y. Wang, F. F. Zhou, W. Pang, X. Chen, Y. Tian and Y. C. Liang	2015	Specific Biomarkers: Detection of Cancer Biomarkers Through High-Throughput Transcriptomics Data	Cognitive Computation	Records excluded after screening
215	Duan, B., X. Hu, M. Fan, X. Xiong, L. Han, Z. Wang, D. Tong, L. Liu, X. Wang, W. Li, J. Yang and C. Huang	2019	RNA-Binding Motif Protein 6 is a Candidate Serum Biomarker for Pancreatic Cancer	Proteomics - Clinical Applications	Not early-stage
216	Duarte-Medrano, G., I. Lopez-Méndez, M. Á. Ramírez-Luna, F. Valdovinos-Andraca, R. Cruz-Martínez, I. Medina-Vera, C. Pérez-Monter and F. I. Téllez-Ávila	2019	Analysis of circulating blood and tissue biopsy PDX1 and MSX2 gene expression in patients with pancreatic cancer: A case-control experimental study	Medicine	Records excluded after screening
217	Dubey, A. K., I. Kaur, R. Madaan, S. Raheja, R. Bala, M. Garg, S. Kumar, V. Lather, V. Mittal, D. Pandita, R. Gundamaraju, R. K. Singla and R. Sharma	2024	Unlocking the potential of oncology biomarkers: advancements in clinical theranostics	Drug Metabolism and Personalized Therapy	Records excluded after screening
218	Dugalic, V. D., D. M. Knezevic, V. N. Obradovic, M. G. Gojnic-Dugalic, S. V. Matic, A. R. Pavlovic-Markovic, P. D. Dugalic and S. M. Knezevic	2014	Drain amylase value as an early predictor of pancreatic fistula after cephalic duodenopancreatectomy	World J Gastroenterol	Records excluded after screening
219	Dumitrescu, R. G.	2018	Early Epigenetic Markers for Precision Medicine. Cancer Epigenetics for Precision Medicine: Methods and Protocols. R. G. Dumitrescu and M. Verma.		Records excluded after screening
220	Dumstrei, K., H. Chen and H. Brenner	2016	A systematic review of serum autoantibodies as biomarkers for pancreatic cancer detection	Oncotarget	Records excluded after screening
221	Durin, L., A. Pradines, C. Basset, B. Ulrich, L. Keller, V. Dongay, G. Favre, J. Mazieres and N. Guibert	2020	Liquid biopsy of non-plasma body fluids in non-small cell lung cancer: Look closer to the tumor!	Cells	Records excluded after screening

222	Dutta, P., S. C. Pando, M. Mascaro, E. Riquelme, M. Zoltan, N. M. Zacharias, S. T. Gammon, D. Piwnica-Worms, M. D. Pagel, S. Sen, A. Maitra, S. Shams, F. McAllister and P. K. Bhattacharya	2020	Early Detection of Pancreatic Intraepithelial Neoplasias (PanINs) in Transgenic Mouse Model by Hyperpolarized ¹³C Metabolic Magnetic Resonance Spectroscopy	International Journal of Molecular Sciences	Records excluded after screening
223	Dutta, S. K., M. Girotra, M. Singla, A. Dutta, F. Otis Stephen, P. P. Nair and N. B. Merchant	2012	Serum HSP70: a novel biomarker for early detection of pancreatic cancer	Pancreas	Records excluded after screening
224	Dutton, K. and M. Soden	2017	Malignancy screening in autoimmune myositis among Australian rheumatologists	Internal Medicine Journal	Records excluded after screening
225	eBioMedicine	2022	Emerging biomarkers for early diagnosis of pancreatic cancer	eBioMedicine	Records excluded after screening
226	Eckhoff, A., E. Kanu, A. Fletcher, M. Bao, D. P. Nussbaum, D. G. Blazer, M. E. Lidsky, S. Zani, K. Shah, V. Aushev, N. S. Krinshpun, N. A. Jurdi, N. M. Liu, N. P. J. Allen and G. Herbert	2023	Personalized Circulating Tumor DNA and Survival in Patients with Resectable Pancreatic Cancer	Annals of Surgical Oncology	Records excluded after screening
227	Effraimidis, G., U. Knigge, M. Rossing, P. Oturai, Å. K. Rasmussen and U. Feldt-Rasmussen	2022	Multiple endocrine neoplasia type 1 (MEN-1) and neuroendocrine neoplasms (NENs)	Seminars in Cancer Biology	Records excluded after screening
228	Einoch Amor, R., A. Zinger, Y. Y. Broza, A. Schroeder and H. Haick	2022	Artificially Intelligent Nanoarray Detects Various Cancers by Liquid Biopsy of Volatile Markers	Adv Healthc Mater	Records excluded after screening
229	Eissa, M. A. L., L. Lerner, E. Abdelfatah, N. Shankar, J. K. Canner, N. M. Hasan, V. Yaghoobi, B. Huang, Z. Kerner, F. Takaesu, C. Wolfgang, R. Kwak, M. Ruiz, M. Tam, T. R. Pisanic, C. A. Iacobuzio-Donahue, R. H. Hruban, J. He, T. H. Wang, L. D. Wood, A. Sharma and N. Ahuja	2019	Promoter methylation of ADAMTS1 and BNC1 as potential biomarkers for early detection of pancreatic cancer in blood	Clinical Epigenetics	Studies included in review
230	Elfaituri, M. K. and A. Khaled	2023	83P Evaluation of serum macrophage inhibitory cytokine 1 as a diagnostic biomarker for pancreatic cancer: A systematic review and diagnostic accuracy meta-analysis	ESMO Open	Records excluded after screening

231	El-Jurdi, N. H. and M. W. Saif	2014	Pancreatic cancer: new hopes for early detection and a future screening tool?	JOP : Journal of the pancreas	Records excluded after screening
232	Endo, Y., M. Fujimoto, N. Ito, Y. Takahashi, M. Kitago, M. Gotoh, N. Hiraoka, T. Yoshida, Y. Kitagawa, Y. Kanai and E. Arai	2021	Clinicopathological impacts of DNA methylation alterations on pancreatic ductal adenocarcinoma: prediction of early recurrence based on genome-wide DNA methylation profiling	J Cancer Res Clin Oncol	Not early-stage
233	Engels, M. M., C. K. Berger, S. A. Hoogenboom, D. Sarwal, D. C. Klatte, J. De La Fuente, S. Gandhi, W. R. Taylor, P. H. Foote, K. Doering, A. Delgado, K. N. Burger, D. Mahoney, B. K. A. Dayyeh, A. Bofill-Garcia, B. brahmbhatt, V. Chandrasekhara, F. C. Gleeson, V. Gomez, V. Kumbhari, R. Law, M. J. Levy, F. Lukens, M. Raimondo, E. Rajan, A. C. Storm, E. J. Vargas, M. B. Wallace, J. B. Kisiel and S. Majumder	2023	PANCREATIC CANCER DETECTION USING METHYLATED DNA MARKERS IN PANCREATIC JUICE: A PROSPECTIVE MULTICENTER VALIDATION STUDY	Gastroenterology	Records excluded after screening
234	Esposito, I., B. Konukiewitz, A. M. Schlitter and G. Klö ppel	2014	Pathology of pancreatic ductal adenocarcinoma: Facts, challenges and future developments	World Journal of Gastroenterology	Records excluded after screening
235	Ettrich, T. J., A. W. Berger, A. Reinacher-Schick, H. Algül, A. Kö nig, E. Gallmeier, K. Wille, S. Daum, M. Geissler, A. Tannapfel, W. Uhl and T. Seufferlein	2018	A composite liquid biomarker for non-invasive diagnosis of resectable pancreatic ductal adenocarcinoma	United European Gastroenterology Journal	Records excluded after screening
236	Ettrich, T. J., A. W. Berger, D. Schwerdel, A. C. Reinacher-Schick, W. Uhl, H. Alguel, H. Friess, A. Koenig, M. Ghadimi, E. Gallmeier, D. K. Bartsch, M. Geissler, L. Staib, A. Tannapfel, A. Kleger and T. Seufferlein	2019	A blood-based assay for diagnosis of early-stage pancreatic cancer	Journal of Clinical Oncology	Records excluded after screening
237	Euctr, D. E.	2012	Afatinib as Cancer therapy for Exocrine Pancreatic Tumours	https://trialsearch.who.int/Trial2.aspx?TrialID=EUCTR2011-004063-77-DE	Records excluded after screening

238	Fahrmann, J. F., T. Marsh, E. Irajizad, N. Patel, E. Murage, J. Vykoukal, J. B. Dennison, K. A. Do, E. Ostrin, M. R. Spitz, S. Lam, S. Shete, R. Meza, M. C. Tammemägi, Z. Feng and S. M. Hanash	2022	Blood-Based Biomarker Panel for Personalized Lung Cancer Risk Assessment	J Clin Oncol	Records excluded after screening
239	Fahrmann, J. F., C. M. Schmidt, X. Mao, E. Irajizad, M. Loftus, J. Zhang, N. Patel, J. Vykoukal, J. B. Dennison, J. P. Long, K. A. Do, J. Zhang, J. A. Chabot, M. D. Kluger, F. Kastrinos, L. Brais, A. Babic, K. Jajoo, L. S. Lee, T. E. Clancy, K. Ng, A. Bullock, J. Genkinger, M. T. Yip-Schneider, A. Maitra, B. M. Wolpin and S. Hanash	2021	Lead-Time Trajectory of CA19-9 as an Anchor Marker for Pancreatic Cancer Early Detection	Gastroenterology	No sample information provided
240	Faleiro, I. and P. Castelo-Branco	2018	Epigenetic alterations of the PI3K/Akt signaling pathway as potential pancreatic cancer biomarkers	Annals of Medicine	Records excluded after screening
241	Faleiro, I., V. P. Roberto, S. D. Canli, N. A. Fraunhoffer, J. Iovanna, A. O. Gure, W. Link and P. Castelo-Branco	2021	DNA Methylation of PI3K/AKT Pathway-Related Genes Predicts Outcome in Patients with Pancreatic Cancer: A Comprehensive Bioinformatics-Based Study	Cancers	Records excluded after screening
242	Fan, C., H. Qu, X. Wang, N. Sobhani, L. Wang, S. Liu, W. Xiong, Z. Zeng and Y. Li	2021	Cancer/testis antigens: from serology to mRNA cancer vaccine	Seminars in Cancer Biology	Records excluded after screening
243	Fang, J. M., J. Li and J. Shi	2022	An update on the diagnosis of gastroenteropancreatic neuroendocrine neoplasms	World Journal of Gastroenterology	Records excluded after screening
244	Farina, A., J. M. Dumonceau, P. Antinori, I. Annessi-Ramseyer, J. L. Frossard, D. F. Hochstrasser, M. Delhaye and P. Lescuyer	2014	Bile carcinoembryonic cell adhesion molecule 6 (CEAM6) as a biomarker of malignant biliary stenoses	Biochimica Et Biophysica Acta-Proteins and Proteomics	Records excluded after screening
245	Farooq, M., E. Leevan, J. Ahmed, B. Ko, S. Shin, A. De Souza and N. Takebe	2024	Blood-based multi-cancer detection: A state-of-the-art update	Current Problems in Cancer	Records excluded after screening

246	Felix, K., K. Honda, K. Nagashima, A. Kashiro, K. Takeuchi, T. Kobayashi, S. Hinterkopf, M. M. Gaida, H. Dang, N. Brindl, J. Kaiser, M. W. Büchler and O. Strobel	2022	Noninvasive risk stratification of intraductal papillary mucinous neoplasia with malignant potential by serum apolipoprotein-A2-isoforms	International Journal of Cancer	Not early-stage
247	Feng, Z., M. E. Hom, T. E. Bearrood, Z. C. Rosenthal, D. Fernández, A. E. Ondrus, Y. Gu, A. K. McCormick, M. G. Tomaske, C. R. Marshall, T. Kline, C. H. Chen, D. Mochly-Rosen, C. J. Kuo and J. K. Chen	2022	Targeting colorectal cancer with small-molecule inhibitors of ALDH1B1	Nature Chemical Biology	Records excluded after screening
248	Feng, Z., K. Li, K. Qin, J. Liang, M. Shi, Y. Ma, S. Zhao, H. Liang, D. Han, B. Shen, C. Peng, H. Chen and L. Jiang	2022	The LINC00623/NAT10 signaling axis promotes pancreatic cancer progression by remodeling ac4C modification of mRNA	Journal of Hematology and Oncology	Records excluded after screening
249	Fiala, C. and E. P. Diamandis	2020	Can a Broad Molecular Screen Based on Circulating Tumor DNA Aid in Early Cancer Detection?	Journal of Applied Laboratory Medicine	Records excluded after screening
250	Filiputti, V. B., M. L. A. Cirino, F. S. L. Neto, P. C. Novais, L. P. Turra, M. Tazima, F. M. Peria, D. P. C. Tirapelli, R. Kemp, J. S. dos Santos and A. K. Sankarankutty	2020	Combination of microRNA-21 expression with the serum marker CA19- 9 increases the accuracy of diagnosis of pancreatic adenocarcinoma	Genetics and Molecular Research	Records excluded after screening
251	Firpo, M. A., K. M. Boucher, J. Bleicher, G. D. Khanderao, A. Rosati, K. E. Poruk, S. Kamal, L. Marzullo, M. De Marco, A. Falco, A. Genovese, J. M. Adler, V. De Laurenzi, D. G. Adler, K. E. Affolter, I. Garrido-Laguna, C. L. Scaife, M. C. Turco and S. J. Mulvihill	2022	A multi-analyte serum biomarker panel for early detection of pancreatic adenocarcinoma.		Records excluded after screening

252	Firpo, M. A., K. M. Boucher, J. Bleicher, G. D. Khanderao, A. Rosati, K. E. Poruk, S. Kamal, L. Marzullo, M. De Marco, A. Falco, A. Genovese, J. M. Adler, V. De Laurenzi, D. G. Adler, K. E. Affolter, I. Garrido-Laguna, C. L. Scaife, M. C. Turco and S. J. Mulvihill	2023	Multianalyte Serum Biomarker Panel for Early Detection of Pancreatic Adenocarcinoma	JCO Clin Cancer Inform	Studies included in review
253	Firpo, M. A., K. M. Boucher and S. J. Mulvihill	2014	Prospects for developing an accurate diagnostic biomarker panel for low prevalence cancers	Theoretical Biology and Medical Modelling	Records excluded after screening
254	Flick, K. F., M. T. Yip-Schneider, C. M. Sublette, R. E. Simpson, C. L. Colgate, H. Wu, M. Soufi, J. M. Dewitt, A. L. Mosley, E. P. Ceppa, J. Zhang and C. M. Schmidt	2020	A Quantitative Global Proteomics Approach Identifies Candidate Urinary Biomarkers That Correlate with Intraductal Papillary Mucinous Neoplasm Dysplasia	Pancreas	Records excluded after screening
255	Flory, A. and H. Wilson-Robles	2024	Noninvasive Blood-Based Cancer Detection in Veterinary Medicine	Veterinary Clinics of North America - Small Animal Practice	Records excluded after screening
256	Forones, N. M.	2018	Tumor markers in gastrointestinal cancer	Tumor Biology	Records excluded after screening
257	Forouzandeh, N., S. Srinivasan, K. Jurdi, J. Q. Wang, N. Tofteland, W. Yang, W. Salyers and J. Lu	2021	Digital PCR-based cell-free DNA KRas mutations analysis and clinical implication in pancreatic cancer	Journal of Clinical Oncology	Records excluded after screening
258	Fottner, C., S. Sollfrank, M. Ghiasi, A. Adenaeuer, T. Musholt, A. Schad, M. Miederer, S. Schadmand-Fischer, M. M. Weber, K. J. Lackner and H. Rossmann	2022	Second MAFA Variant Causing a Phosphorylation Defect in the Transactivation Domain and Familial Insulinomatosis	Cancers	Records excluded after screening
259	Franklin, O., D. Ö hlund, C. Lundin, M. Öman, P. Naredi, W. Wang and M. Sund	2015	Combining conventional and stroma-derived tumour markers in pancreatic ductal adenocarcinoma	Cancer Biomarkers	Small sample size
260	Franses, J. W., O. Basar, A. Kadayifci, O. Yuksel, M. Choz, A. S. Kulkarni, E. Tai, K. D. Vo, K. S. Arora, N. Desai, J. A. Licausi, M. Toner, S. Maheswaran, D. A. Haber, D. P. Ryan, W. R. Brugge and D. T. Ting	2018	Improved Detection of Circulating Epithelial Cells in Patients with Intraductal Papillary Mucinous Neoplasms	Oncologist	Records excluded after screening

261	Friedecký, D. and A. Kvasnička	2023	MASS SPECTROMETRY BASED OMICS IN CANCER DIAGNOSIS	Clinical Chemistry and Laboratory Medicine	Records excluded after screening
262	Fu, L., W. Jin, J. Zhang, L. Zhu, J. Lu, Y. Zhen, L. Zhang, L. Ouyang, B. Liu and H. Yu	2022	Repurposing non-oncology small-molecule drugs to improve cancer therapy: Current situation and future directions	Acta Pharmaceutica Sinica B	Records excluded after screening
263	Fu, Z. C., F. Qian, X. H. Yang, H. L. Jiang, Y. Chen and S. H. Liu	2014	Circulating miR-222 in plasma and its potential diagnostic and prognostic value in gastric cancer	Medical Oncology	Records excluded after screening
264	Fujimoto, Y., Y. Suehiro, S. Kaino, S. Suenaga, T. Tsuyama, H. Matsui, S. Higaki, I. Fujii, C. Suzuki, T. Hoshida, T. Matsumoto, T. Takami, H. Nagano, I. Sakaida and T. Yamasaki	2021	Combination of CA19-9 and Blood Free-Circulating Methylated RUNX3 May Be Useful to Diagnose Stage I Pancreatic Cancer	Oncology	Studies included in review
265	Fukushige, S. and A. Horii	2014	Road to early detection of pancreatic cancer: Attempts to utilize epigenetic biomarkers	Cancer Lett	Records excluded after screening
266	Fukutake, N., M. Ueno, N. Hiraoka, K. Shimada, K. Shiraishi, N. Saruki, T. Ito, M. Yamakado, N. Ono, A. Imaizumi, S. Kikuchi, H. Yamamoto and K. Katayama	2015	A Novel Multivariate Index for Pancreatic Cancer Detection Based On the Plasma Free Amino Acid Profile	PLoS One	Studies included in review
267	Fuller, R. N., A. Morcos, D. Escalera and N. R. Wall	2024	Unlocking the potential of miRNA sequencing for early detection and monitoring of pancreatic cancer progression and recurrence	Cancer Research	Records excluded after screening
268	Funamizu, N., M. Honjo, K. Tamura, K. Sakamoto, K. Ogawa and Y. Takada	2023	microRNAs Associated with Gemcitabine Resistance via EMT, TME, and Drug Metabolism in Pancreatic Cancer	Cancers	Records excluded after screening
269	Furini, S. and C. Falciani	2021	Expression and Role of Heparan Sulfated Proteoglycans in Pancreatic Cancer	Frontiers in Oncology	Records excluded after screening
270	Gablo, N. A., V. Prochazka, Z. Kala, O. Slaby and I. Kiss	2019	Cell-free microRNAs as non-invasive diagnostic and prognostic biomarkers in pancreatic cancer	Current Genomics	Records excluded after screening
271	Galeano-Garces, C., K. J. Smith, N. Heller, M. Ahmadi, J. Hong, J. Parthasarathy and J. M. Drake	2023	CTC-derived organoids from liver and pancreatic cancer patients for personalized therapy	Cancer Research	Records excluded after screening
272	Gall, T. M., H. Wasan and L. R. Jiao	2015	Pancreatic cancer: current understanding of molecular and genetic aetiologies	Postgrad Med J	Records excluded after screening
273	Gall, T. M. H., S. Belete, E. Khanderia, A. E. Frampton and L. R. Jiao	2019	Circulating Tumor Cells and Cell-Free DNA in Pancreatic Ductal Adenocarcinoma	American Journal of Pathology	Records excluded after screening
274	Gallamini, A., C. Zwarthoed and A. Borra	2014	Positron emission tomography (PET) in oncology	Cancers	Records excluded after screening

275	Ganepola, G. A. P., J. R. Rutledge, P. Suman, A. Yiengpruksawan and D. H. Chang	2014	Novel blood-based microRNA biomarker panel for early diagnosis of pancreatic cancer	World Journal of Gastrointestinal Oncology	Studies included in review
276	Gao, F. Y., F. L. Jiao, C. S. Xia, Y. Zhao, W. T. Ying, Y. P. Xie, X. Y. Guan, M. Tao, Y. J. Zhang, W. J. Qin and X. H. Qian	2019	A novel strategy for facile serum exosome isolation based on specific interactions between phospholipid bilayers and TiO₂	Chemical Science	Records excluded after screening
277	Gao, G., C. Liu, S. Jain, D. Li, H. Wang, Y. Zhao and J. Liu	2019	Potential use of aptamers for diagnosis and treatment of pancreatic cancer	Journal of Drug Targeting	Records excluded after screening
278	Gao, H. J., Z. X. Zheng, Y. S. Mao, W. Wang, Y. Y. Qiao, L. P. Zhou, F. Liu, H. Z. He and X. H. Zhao	2014	Identification of tumor antigens that elicit a humoral immune response in the sera of Chinese esophageal squamous cell carcinoma patients by modified serological proteome analysis	Cancer Letters	Records excluded after screening
279	Gao, Y., M. Wang, X. Guo, J. Hu, T. Chen, J. Lacy, J. W. Kuntsman, C. H. Cha, M. D. Bellin, M. E. Robert, G. V. Desir and F. S. Gorelick	2020	Renalase may play a role in the development of pancreatic cancer and in the survival of pancreatic cancer patients	Pancreas	Records excluded after screening
280	García Vence, M., M. D. P. Chantada-Vázquez, S. Vázquez-Estévez, J. Manuel Cameselle-Teijeiro, S. B. Bravo and C. Núñez	2020	Potential clinical applications of the personalized, disease-specific protein corona on nanoparticles	Clinica Chimica Acta	Records excluded after screening
281	García-Muñoz, R. A., V. Morales and A. Toledano	2014	Cancer diagnosis by breath analysis: What is the future?	Bioanalysis	Records excluded after screening
282	Garcia-Romero, N., S. Esteban-Rubio, G. Rackov, J. Carrión-Navarro, C. Belda-Iniesta and A. Ayuso-Sacido	2018	Extracellular vesicles compartment in liquid biopsies: Clinical application	Molecular Aspects of Medicine	Records excluded after screening
283	Gay, C. M., P. Tong, R. J. Cardnell, T. Sen, X. Su, J. Ma, R. O. Bara, F. M. Johnson, C. Wakefield, J. V. Heymach, J. Wang and L. A. Byers	2019	Differential sensitivity analysis for resistant malignancies (DISARM) identifies common candidate therapies across platinum-resistant cancers	Clinical Cancer Research	Records excluded after screening
284	Gayral, M., S. Jo, N. Hanoun, A. Vignolle-Vidoni, H. Lulka, Y. Delpu, A. Meulle, M. Dufresne, M. Humeau, M. Chalret du Rieu, B. Bournet, J. Sèlves, R. Guimbaud, N. Carrère, L. Buscail, J. Torrisani and P. Cordelier	2014	MicroRNAs as emerging biomarkers and therapeutic targets for pancreatic cancer	World J Gastroenterol	Records excluded after screening

285	Ge, L., B. Pan, F. Song, J. Ma, D. Zeraatkar, J. Zhou and J. Tian	2017	Comparing the diagnostic accuracy of five common tumour biomarkers and CA19-9 for pancreatic cancer: A protocol for a network meta-analysis of diagnostic test accuracy	BMJ Open	Records excluded after screening
286	Ge, W., Y. Wang, M. Quan, T. Mao, E. Y. Bischof, H. Xu, X. Zhang, S. Li, M. Yue, J. Ma, H. Yang, L. Wang, Z. Yu, L. Wang and J. Cui	2024	Activation of the PI3K/AKT signaling pathway by ARNTL2 enhances cellular glycolysis and sensitizes pancreatic adenocarcinoma to erlotinib	Molecular Cancer	Non-diagnostic studies
287	Genco, E., F. Modena, L. Sarcina, K. Björkström, C. Brunetti, M. Caironi, M. Caputo, V. M. Demartis, C. Di Franco, G. Frusconi, L. Haeberle, P. Larizza, M. T. Mancini, R. Österbacka, W. Reeves, G. Scamarcio, C. Scandurra, M. Wheeler, E. Cantatore, I. Esposito, E. Macchia, F. Torricelli, F. A. Viola and L. Torsi	2023	A Single-Molecule Bioelectronic Portable Array for Early Diagnosis of Pancreatic Cancer Precursors	Advanced Materials	Records excluded after screening
288	Ghatak, S., S. Nishiwada, E. Jun, F. Sonohara, Y. Kodera, S. C. Kim and A. Goel	2020	DEVELOPMENT OF A NOVEL CIRCULAR RNA-BASED, NONINVASIVE LIQUID BIOPSY ASSAY FOR THE EARLY DETECTION OF PANCREATIC DUCTAL ADENOCARCINOMA	Gastroenterology	Records excluded after screening
289	Ghiorzo, P.	2014	Genetic predisposition to pancreatic cancer	World J Gastroenterol	Records excluded after screening
290	Ghosh, R., R. Ahmed, H. Ahmed and B. P. Chatterjee	2022	Phosphorylated Proteins from Serum: A Promising Potential Diagnostic Biomarker of Cancer	International Journal of Molecular Sciences	Records excluded after screening
291	Ghweil, A. A., H. A. Osman, M. H. Hassan, A. M. M. Sabry, R. E. Mahdy, A. R. H. Ahmed, A. Okasha, A. Khodeary and H. H. Ameen	2020	Validity of serum amyloid A and HMGB1 as biomarkers for early diagnosis of gastric cancer	Cancer Management and Research	Records excluded after screening
292	Giannis, D., D. Moris and A. S. Barbas	2021	Diagnostic, predictive and prognostic molecular biomarkers in pancreatic cancer: An overview for clinicians	Cancers	Records excluded after screening
293	Giannone, F., N. Slovic, P. Pessaux, C. Schuster, T. F. Baumert and J. Lupberger	2023	Inflammation-related prognostic markers in resected hepatocellular carcinoma	Frontiers in Oncology	Records excluded after screening

294	Gilhotra, R., S. Htut, C. McGowan, M. Gururatsakul, G. Peter-Kini and P. Boyd	2020	The importance of a multidisciplinary team in assessing Liver Imaging Reporting and Data System indeterminate lesions: Wait and watch or biopsy?	Journal of Gastroenterology and Hepatology (Australia)	Records excluded after screening
295	Gillies, R. J. and M. B. Schabath	2020	Radiomics Improves Cancer Screening and Early Detection	Cancer Epidemiol Biomarkers Prev	Records excluded after screening
296	Giovannetti, E., D. Massihnia, A. Avan, N. Funel, M. Maftouh, A. Van Krieken, C. Granchi, R. Raktoe, U. Boggi, B. Aicher, F. Minutolo, A. Russo, L. Leon and G. Peters	2017	Phospho-Akt: A potential resistance marker to chemotherapy and therapy-target to restore sensitivity in pancreatic cancer	European Journal of Cancer	Records excluded after screening
297	Glatz, J., P. B. Garcia-Allende, V. Becker, M. Koch, A. Meining and V. Ntziachristos	2014	Near-infrared fluorescence cholangiopancreatoscopy: Initial clinical feasibility results	Gastrointestinal Endoscopy	Records excluded after screening
298	Goel, S.	2021	PanCan diagnosed: The early diagnosis and personalized treatment of pancreatic cancer	Asia-Pacific Journal of Clinical Oncology	Records excluded after screening
299	Gómez-Peñaloza, C., M. L. Serrano-Aré valo, L. F. Villegas-González, L. Flores-Hernández, L. S. Lino-Silva, E. B. Ruiz-García and J. Diaz-Ch ávez	2019	Addition of analysis of KRAS mutation or immunohistochemistry with MUC1 and carcinoembryonic antigen improves the diagnostic performance of fine needle aspiration cytology for the diagnosis of pancreatic carcinoma	Cytopathology	Records excluded after screening
300	Gong, D., J. A. Guo, C. Shiau, A. D. Jambhale, S. Wang, S. Ginebaugh, P. Z. Yu, K. S. Kapner, S. Chugh, L. Abbassi, D. Zhao, W. W. Wu, P. Chen, H. Singh, W. L. Hwang and A. J. Aguirre	2024	Molecular stratification of therapeutic targets in pancreatic cancer	Cancer Research	Records excluded after screening
301	Gong, J., Q. Zhang, Q. Peng and D. Shi	2024	Identification of Chronic Pancreatitis Associated microRNAs and Genes for the Diagnosis of Pancreatic Cancer	American Surgeon	Non-diagnostic studies
302	Gong, Y., L. Song, L. Ou, Y. Y. Lu, X. Huang and Q. Zeng	2023	Diagnostic and Prognostic Performance of MicroRNA-25, Carbohydrate Antigen 19-9, Carcinoembryonic Antigen, and Carbohydrate Antigen 125 in Pancreatic Ductal Adenocarcinoma	Iranian Journal of Medical Sciences	Small sample size

303	González-Boja, I., A. Viúdez, S. Goñi, E. Santamaria, E. Carrasco-García, J. Pérez-Sanz, I. Hernández-García, P. Sala-Elarre, V. Arrazubi, E. Oyaga-Iriarte, R. Zárate, S. Arévalo, O. Sayar, R. Vera and J. Fernández-Irigoyen	2019	Omics approaches in pancreatic adenocarcinoma	Cancers	Records excluded after screening
304	González-Moreno, J., Á. Gragera-Martínez, A. Rodríguez, C. Borrachero-Garro, S. García-Garrido, C. Barceló, A. Manovel-Sánchez, M. A. Ribot-Sansó, L. Ibargüen-González, R. Gomila, F. Muñoz-Beamud, I. Losada-López and E. Cisneros-Barroso	2024	Biomarkers of axonal damage to favor early diagnosis in variant transthyretin amyloidosis (A-ATTRv)	Scientific reports	Records excluded after screening
305	Goonesekere, N. C. W., X. Wang, L. Ludwig and C. Guda	2014	A meta analysis of pancreatic microarray datasets yields new targets as cancer genes and biomarkers	PLoS ONE	Records excluded after screening
306	Gordon, B. L., B. M. Finnerty, A. Aronova and T. J. Fahey	2015	Genomic medicine for cancer diagnosis	Journal of Surgical Oncology	Records excluded after screening
307	Goyal, H., S. A. A. Sherazi, R. Mann, Z. Gandhi, A. Perisetti, M. Aziz, S. Chandan, J. Kopel, B. Tharian, N. Sharma and N. Thosani	2021	Scope of artificial intelligence in gastrointestinal oncology	Cancers	Records excluded after screening
308	Grady, W. M., M. Yu and S. D. Markowitz	2021	Epigenetic Alterations in the Gastrointestinal Tract: Current and Emerging Use for Biomarkers of Cancer	Gastroenterology	Records excluded after screening
309	Grande, A. J., V. Silva, L. Sawaris Neto, J. P. Teixeira Basmage, M. S. Peccin and M. Maddocks	2021	Exercise for cancer cachexia in adults	Cochrane Database of Systematic Reviews	Records excluded after screening
310	Green, M. F., J. L. Bell, C. B. Hubbard, S. J. McCall, M. S. McKinney, J. E. Riedel, C. S. Menendez, J. L. Abbruzzese, J. H. Strickler and M. B. Datto	2021	Implementation of a molecular tumor registry to support the adoption of precision oncology within an Academic Medical Center: The Duke University Experience	JCO Precision Oncology	Records excluded after screening

311	Gress, T. M., L. Lausser, L. R. Schirra, L. Ortmüller, R. Diels, B. Kong, C. W. Michalski, T. Hackert, O. Strobel, N. A. Giese, M. Schenk, R. T. Lawlor, A. Scarpa, H. A. Kestler and M. Buchholz	2017	Combined microRNA and mRNA microfluidic TaqMan array cards for the diagnosis of malignancy of multiple types of pancreaticobiliary tumors in fine-needle aspiration material	Oncotarget	Non-diagnostic studies
312	Grinkevitch, V., M. Wappett, N. Crawford, S. Price, A. Lees, C. McCann, K. McAllister, J. Prehn, J. Young, J. Bateson, L. Gallagher, M. Michaut, V. Iyer, A. Chatzipli, S. Barthorpe, D. Ciznadija, I. Sloma, A. Wesa, D. A. Tice, L. Wessels, M. Garnett, D. B. Longley, U. McDermott and S. S. McDade	2022	Functional Genomic Identification of Predictors of Sensitivity and Mechanisms of Resistance to Multivalent Second-Generation TRAIL-R2 Agonists	Molecular Cancer Therapeutics	Records excluded after screening
313	Gu, M., J. Sun, S. Zhang, J. Chen, G. Wang, S. Ju and X. Wang	2021	A novel methylation signature predicts inferior outcome of patients with PDAC	Aging	Not early-stage
314	Gu, W. and Z. Tong	2020	Clinical Application of Metabolomics in Pancreatic Diseases: A Mini-Review	Lab Medicine	Records excluded after screening
315	Gu, X. and T. Minko	2024	Targeted Nanoparticle-Based Diagnostic and Treatment Options for Pancreatic Cancer	Cancers	Records excluded after screening
316	Gu, Y., Q. Hua, Z. Li, X. Zhang, C. Lou, Y. Zhang, W. Wang, P. Cai and J. Zhao	2023	Diagnostic value of combining preoperative inflammatory markers ratios with CA199 for patients with early-stage pancreatic cancer	BMC cancer	Not early-stage
317	Gu, Y. L., C. Lan, H. Pei, S. N. Yang, Y. F. Liu and L. L. Xiao	2015	Applicative Value of Serum CA19-9, CEA, CA125 and CA242 in Diagnosis and Prognosis for Patients with Pancreatic Cancer Treated by Concurrent Chemoradiotherapy	Asian Pacific journal of cancer prevention : APJCP	No specific biomarkers provided
318	Guerrero, P. E., A. Duran, M. R. Ortiz, E. Castro, A. Garcia-Velasco, E. Llop and R. Peracaula	2021	Microfibril associated protein 4 (MFAP4) is a carrier of the tumor associated carbohydrate sialyl-Lewis x (sLex) in pancreatic adenocarcinoma	Journal of Proteomics	Records excluded after screening
319	Gui, J. C., W. L. Yan and X. D. Liu	2014	CA19-9 and CA242 as tumor markers for the diagnosis of pancreatic cancer: a meta-analysis	Clin Exp Med	Records excluded after screening
320	Guo, S., M. Su, C. Ma, X. Shi, M. Xu, S. Gao, H. Wang, Y. Pan, Q. He, Z. Su and G. Jin	2022	Aberrant circulating tumour DNA methylations as biomarkers for early detection of pancreatic ductal adenocarcinoma: a retrospective study	The Lancet Oncology	Records excluded after screening

321	Guo, S., M. Su, X. Shi, S. Gao, H. Wang, Y. Pan, C. Gong, Q. He, R. Liu and G. Jin	2022	Development of PCR assays to detect signature circulating tumor DNA methylation markers and KRas mutations for pancreatic ductal adenocarcinoma (PDAC)	Journal of Clinical Oncology	Records excluded after screening
322	Guo, Y., J. Ren, X. Li, X. Liu, N. Liu, Y. Wang and Z. Li	2017	Simultaneous quantification of serum Multi-Phospholipids as potential biomarkers for differentiating different pathophysiological states of lung, stomach, intestine, and pancreas	Journal of Cancer	Records excluded after screening
323	Gupta, M., R. Iyer and C. Fountzilas	2019	Poly(ADP-ribose) polymerase inhibitors in pancreatic cancer: A new treatment paradigms and future implications	Cancers	Records excluded after screening
324	Haab, B., L. Qian, B. Staal, M. Jain, J. Fahrmann, C. Worthington, D. Prosser, L. Velokokhatnaya, C. Lopez, R. Tang, M. Hurd, G. Natarajan, S. Kumar, L. Smith, S. Hanash, S. Batra, A. Maitra, A. Lokshin, Y. Huang and R. E. Brand	2024	Combinations of previously reported biomarkersachieve improved sensitivity and specificity of detection of early stage pancreatic ductal adenocarcinoma	Cancer Research	Studies included in review
325	Habartová, L., B. Bunganic, M. Tatarkovic, M. Zavoral, J. Vondrousová, K. Syslová and V. Setnicka	2018	Chiroptical spectroscopy and metabolomics for blood-based sensing of pancreatic cancer	Chirality	Records excluded after screening
326	Habartova, L., J. Vondrousova, K. Syslova, B. Bunganic, S. Suchanek, M. Zavoral and V. Setnicka	2019	Metabolomic profiling of pancreatic cancer	United European Gastroenterology Journal	Records excluded after screening
327	Hagen, M. E., J. Douissard, F. Ris and C. Toso	2021	Liquid biopsy & surgery: What's ahead?	American Journal of Surgery	Records excluded after screening
328	Hagihara, A., Y. Murakami, A. Tamori, K. Kimura, R. Amano, K. Hirakawa and N. Kawada	2016	Pancreatic cancer biomarkers among exosome-fractionated circulating miRNAs	Journal of Clinical Oncology	Records excluded after screening
329	Hajeri, P. B., N. S. Sharma and M. Yamamoto	2020	Oncolytic adenoviruses: Strategies for improved targeting and specificity	Cancers	Records excluded after screening
330	Han, H. S. and K. W. Lee	2024	Liquid Biopsy: An Emerging Diagnostic, Prognostic, and Predictive Tool in Gastric Cancer	Journal of Gastric Cancer	Records excluded after screening
331	Han, S., G. Jin, L. Wang, M. Li, C. He, X. Guo and Q. Zhu	2014	The role of PAM4 in the management of pancreatic cancer: Diagnosis, radioimmunodetection, and radioimmunotherapy	Journal of Immunology Research	Records excluded after screening

332	Han, S. X., X. Zhou, X. Sui, C. C. He, M. J. Cai, J. L. Ma, Y. Y. Zhang, C. Y. Zhou, C. X. Ma, A. Varela-Ramirez and Q. Zhu	2015	Serum dickkopf-1 is a novel serological biomarker for the diagnosis and prognosis of pancreatic cancer	Oncotarget	Studies included in review
333	Hanada, K., A. Shimizu, K. Kurihara, M. Ikeda, T. Yamamoto, Y. Okuda and S. Tazuma	2022	Endoscopic approach in the diagnosis of high-grade pancreatic intraepithelial neoplasia	Digestive Endoscopy	Records excluded after screening
334	Hanna-Sawires, R. G., J. H. Schiphuis, M. Wuhrer, H. F. A. Vasen, M. E. van Leerdam, B. A. Bonsing, W. E. Mesker, Y. E. M. van der Burgt and R. Tollenaar	2021	Clinical Perspective on Proteomic and Glycomic Biomarkers for Diagnosis, Prognosis, and Prediction of Pancreatic Cancer	Int J Mol Sci	Records excluded after screening
335	Hao, Y., F. Zhang, Y. Ma, Y. Luo, Y. Zhang, N. Yang, M. Liu, H. Liu and J. Li	2023	Potential biomarkers for the early detection of bone metastases	Frontiers in Oncology	Records excluded after screening
336	Hasan, S., R. Jacob, U. Manne and R. Paluri	2019	Advances in pancreatic cancer biomarkers	Oncology Reviews	Records excluded after screening
337	Hashimoto, K., M. Inada, Y. Yamamoto and T. Ochiya	2021	Preliminary evaluation of miR-1307-3p in human serum for detection of 13 types of solid cancer using microRNA chip	Heliyon	Records excluded after screening
338	Hata, T., M. Mizuma, T. Kusakabe, H. Amano, T. Furukawa, T. Iwao and M. Unno	2023	Simultaneous and sequential combination of genetic and epigenetic biomarkers for the presence of high-grade dysplasia in patients with pancreatic cyst: Discovery in cyst fluid and test in pancreatic juice	Pancreatology	Records excluded after screening
339	Hayashi, H., N. Uemura, K. Matsumura, L. Zhao, H. Sato, Y. Shiraishi, Y. I. Yamashita and H. Baba	2021	Recent advances in artificial intelligence for pancreatic ductal adenocarcinoma	World J Gastroenterol	Records excluded after screening
340	He, J., J. Long, C. Zhai, J. Xu, K. Bao, W. Su, L. Jiang, G. Shen and X. Ding	2024	Codetection of Proteins and RNAs on Extracellular Vesicles for Pancreatic Cancer Early Diagnosis	Anal Chem	Non-diagnostic studies
341	He, R. Q., P. R. Wu, X. L. Xue, X. Yang, H. W. Liang, X. H. Qiu, L. H. Yang, Z. G. Peng and G. Chen	2018	Downregulated miR-23b-3p expression acts as a predictor of hepatocellular carcinoma progression: A study based on public data and RT-qPCR verification	International Journal of Molecular Medicine	Records excluded after screening
342	He, S., F. Zeng, H. Yin, P. Wang, Y. Bai, Q. Song, J. Chu, Z. Huang, Y. Liu, H. Liu, Q. Chen, L. Liu, J. Zhou, H. Hu, X. Li, T. Li, G. Wang, J. Cai, Y. Jiao and H. Zhao	2023	Molecular diagnosis of pancreatobiliary tract cancer by detecting mutations and methylation changes in bile samples	eClinicalMedicine	Not early-stage

343	He, X., J. Zhong, S. Wang, Y. Zhou, L. Wang, Y. Zhang and Y. Yuan	2017	Serum metabolomics differentiating pancreatic cancer from new-onset diabetes	Oncotarget	Records excluded after screening
344	He, X. Y. and Y. Z. Yuan	2014	Advances in pancreatic cancer research: moving towards early detection	World J Gastroenterol	Records excluded after screening
345	Henriksen, S. D., P. H. Madsen, A. C. Larsen, M. B. Johansen, A. M. Drewes, I. S. Pedersen, H. Krarup and O. Thorlacius-Ussing	2016	Cell-free DNA promoter hypermethylation in plasma as a diagnostic marker for pancreatic adenocarcinoma	Clinical Epigenetics	Studies included in review
346	Hernandez, S., A. L. Lourenco, E. Calabrese, T. York, A. Glencer, S. Behr, Z. J. Wang, E. Koay, C. Craik and K. Kirkwood	2020	Pancreatic Cyst Risk Stratification for Early Detection of Pancreatic Cancer Using Quantitative Radiomics and Activity-Based Biomarkers	Journal of Clinical and Translational Science	Records excluded after screening
347	Herreros-Villanueva, M. and L. Bujanda	2016	Glypican-1 in exosomes as biomarker for early detection of pancreatic cancer	Annals of Translational Medicine	Records excluded after screening
348	Herreros-Villanueva, M. and L. Bujanda	2016	Non-invasive biomarkers in pancreatic cancer diagnosis: What we need versus what we have	Annals of Translational Medicine	Records excluded after screening
349	Heymann, J. J. and M. T. Siddiqui	2020	Ancillary Techniques in Cytologic Specimens Obtained from Solid Lesions of the Pancreas: A Review	Acta Cytologica	Records excluded after screening
350	Hinestrosa, J. P., H. Dhani, G. Schroeder, J. M. Lewis, H. I. Balcer, R. Kurzrock, D. Keith, R. Sears and P. Billings	2023	Pancreatic ductal adenocarcinoma (PDAC) early detection	Cancer Research	Records excluded after screening
351	Hinestrosa, J. P., J. M. Lewis, H. I. Balcer, R. Kurzrock, S. Lippman and R. Krishnan	2022	Blood-based extracellular vesicle biomarker test for detection of early-stage pancreatic cancer	Cancer Research	Records excluded after screening
352	Hiramoto, H., T. Muramatsu, D. Ichikawa, E. Otsuji and J. Inazawa	2018	miR-509-5p and miR-1243 inhibit epithelial-mesenchymal transition in pancreatic cancer	Cancer Science	Records excluded after screening
353	Hiramoto, H., T. Muramatsu, D. Ichikawa, K. Tanimoto, S. Komatsu, S. Yasukawa, E. Otsuji and J. Inazawa	2017	Exploring epithelial-mesenchymal transition: Suppressive mirnas using combination of cell-based reporter system and miRNA library	Journal of the American College of Surgeons	Records excluded after screening
354	Hiramoto, H., T. Muramatsu, D. Ichikawa, K. Tanimoto, S. Yasukawa, E. Otsuji and J. Inazawa	2017	miR-509-5p and miR-1243 increase the sensitivity to gemcitabine by inhibiting epithelial-mesenchymal transition in pancreatic cancer	Scientific reports	Records excluded after screening

355	Hirata, Y., T. Kobayashi, S. Nishiumi, K. Yamanaka, T. Nakagawa, S. Fujigaki, T. Iemoto, M. Kobayashi, T. Okusaka, S. Nakamori, M. Shimahara, T. Ueno, A. Tsuchida, N. Sata, T. Ioka, Y. Yasunami, T. Kosuge, T. Kaneda, T. Kato, K. Yagihara, S. Fujita, T. Yamada, K. Honda, T. Azuma and M. Yoshida	2017	Identification of highly sensitive biomarkers that can aid the early detection of pancreatic cancer using GC/MS/MS-based targeted metabolomics	Clinica Chimica Acta	Studies included in review
356	Hocker, J. R., R. G. Postier, M. Li, M. R. Lerner, S. A. Lightfoot, M. D. Peyton, S. J. Deb, C. M. Baker, T. L. Williams, R. J. Hanas, D. E. Stowell, T. J. Lander, D. J. Brackett and J. S. Hanas	2015	Discriminating patients with early-stage pancreatic cancer or chronic pancreatitis using serum electrospray mass profiling	Cancer Letters	No specific biomarkers provided
357	Hofving, T., Y. Arvidsson, B. Almobarak, L. Inge, R. Pfragner, M. Persson, G. Stenman, E. Kristiansson, V. Johanson and O. Nilsson	2018	The neuroendocrine phenotype, genomic profile and therapeutic sensitivity of GEPNET cell lines	Endocr Relat Cancer	Records excluded after screening
358	Hogendorf, P., A. Durczynski, A. Skulimowski, A. Kumor, G. Poznanska and J. Strzelczyk	2018	Growth differentiation factor (GDF-15) concentration combined with Ca125 levels in serum is superior to commonly used cancer biomarkers in differentiation of pancreatic mass	Cancer Biomarkers	Records excluded after screening
359	Holm, M., M. Saraswat, S. Joenväär ä, A. Ristimäki, C. Haglund and R. Renkonen	2018	Colorectal cancer patients with different C reactive protein levels and 5-year survival times can be differentiated with quantitative serum proteomics	Plos One	Records excluded after screening
360	Honda, K.	2022	Risk stratification of pancreatic cancer by a blood test for apolipoprotein A2-isoforms	Cancer Biomarkers	Non-diagnostic studies
361	Honda, K., F. Canzian, T. Kobayashi, Y. Sato, A. Kashiro, K. Takeuchi, Y. Nomura, H. Konishi, V. Katzke, S. Srivastava and R. Kaaks	2022	Early detection of pancreatic cancer and stratification of high-risk individuals using apolipoprotein A2-isoforms	Pancreatology	Records excluded after screening

362	Honda, K., A. H. Kashiro, M. Kobayashi, K. Takeuchi, Y. Shizume, T. Oh, C. Morizane, S. Hijioka and S. Nara	2024	Early detection and risk stratification of pancreatic cancer using apolipoprotein A2-isoforms as a blood biomarker	Cancer Research	Records excluded after screening
363	Honda, K., V. Katzke, A. Hüsing, S. Okaya, H. Shoji, K. Onidani, F. Canzian and R. Kaaks	2018	Carbohydrate antigen 19-9 and apolipoprotein A2 isoform as early detection biomarkers for pancreatic cancer: A prospective evaluation by the EPIC study	Annals of Oncology	Records excluded after screening
364	Honda, K., V. Katzke, A. Hüsing, M. Yoshida, S. Kikuchi, F. Canzian and R. Kaaks	2019	Apolipoprotein A2 isoform and CA19-9 as biomarkers for early detection of pancreatic cancer - A prospective evaluation by the EPIC study	Pancreatology	Records excluded after screening
365	Honda, K., V. A. Katzke, A. Hüsing, S. Okaya, H. Shoji, K. Onidani, A. Olsen, A. Tjonneland, K. Overvad, E. Weiderpass, P. Vineis, D. Muller, K. Tsilidis, D. Palli, V. Pala, R. Tumino, A. Naccarati, S. Panico, K. Aleksandrova, H. Boeing, H. B. Bueno-de-Mesquita, P. H. Peeters, A. Trichopoulou, P. Lagiou, K. T. Khaw, N. Wareham, R. C. Travis, S. Merino, E. J. Duell, M. Rodríguez-Barranco, M. D. Chirlaque, A. Barricarte, V. Rebours, M. C. Boutron-Ruault, F. R. Mancini, P. Brennan, G. Scelo, J. Manjer, M. Sund, D. Öhlund, F. Canzian and R. Kaaks	2019	CA19-9 and apolipoprotein-A2 isoforms as detection markers for pancreatic cancer: a prospective evaluation	International Journal of Cancer	No sample information provided
366	Honda, K., T. Kobayashi, Y. Sato, S. Nishiumi, S. Okaya, K. Takeuchi, K. Nagashima and M. Yoshida	2019	Stratification of pancreatic cancer risk in the general population using an apolipoprotein A2 isoform blood test	Pancreas	Records excluded after screening
367	Honda, K. and S. Srivastava	2016	Potential usefulness of apolipoprotein A2 isoforms for screening and risk stratification of pancreatic cancer	Biomarkers in Medicine	Records excluded after screening
368	Hong, L., L. Xu, L. Jin, K. Xu, W. Tang, Y. Zhu, X. Qiu and J. Wang	2022	Exosomal circular RNA hsa_circ_0006220, and hsa_circ_0001666 as biomarkers in the diagnosis of pancreatic cancer	Journal of Clinical Laboratory Analysis	Records excluded after screening

369	Horala, A., A. Swiatly, J. Matysiak, P. Banach, E. Nowak-Markwitz and Z. J. Kokot	2017	Diagnostic Value of Serum Angiogenesis Markers in Ovarian Cancer Using Multiplex Immunoassay	International Journal of Molecular Sciences	Records excluded after screening
370	Hossan, M. S., E. S. Lin, E. Riedl, A. Stram, E. Mehlhaff, L. Koeppel, J. Warner, I. Uko, L. M. Gettle, S. Lubner, S. M. McGregor, W. Zhang, W. Murphy and J. D. Kratz	2023	Spatial Alignment of Organoids Tracking Subclonal Chemotherapy Resistance in Pancreatic and Ampullary Cancer	Bioengineering-Basel	Non-diagnostic studies
371	Hou, J., X. T. Li and K. P. Xie	2021	Coupled liquid biopsy and bioinformatics for pancreatic cancer early detection and precision prognostication	Molecular Cancer	Records excluded after screening
372	Houvast, R. D., M. van Duijvenvoorde, J. X. Chua, M. Vankemmelbeke, L. G. Durrant, A. Inderson, J. E. van Hooft, J. S. D. Mieog, B. A. Bonsing, C. F. M. Sier, A. S. L. P. Crobach, A. L. Vahrmeijer and P. J. K. Kuppen	2023	Prediction of Biomarker Expression on Primary Pancreatic Ductal Adenocarcinoma Tissues Using Fine-Needle Biopsies: Paving the Way for a Patient-Tailored Molecular Imaging Approach	Molecular Diagnosis and Therapy	Records excluded after screening
373	Hsu, C. C. and Y. Wu	2022	Recent advances in nanotechnology-enabled biosensors for detection of exosomes as new cancer liquid biopsy	Experimental Biology and Medicine	Records excluded after screening
374	Hsu, H. J., C. P. Tung, C. M. Yu, C. Y. Chen, H. S. Chen, Y. C. Huang, P. H. Tsai, S. I. Lin, H. P. Peng, Y. K. Chiu, Y. L. Tsou, W. Y. Kuo, J. W. Jian, F. H. Hung, C. Y. Hsieh, M. Hsiao, S. S. H. Chuang, C. N. Shen, Y. A. Wang and A. S. Yang	2021	Eradicating mesothelin-positive human gastric and pancreatic tumors in xenograft models with optimized anti-mesothelin antibody-drug conjugates from synthetic antibody libraries	Scientific reports	Records excluded after screening
375	Hsu, S. K., M. Jadhao, W. T. Liao, W. T. Chang, I. L. Lin and C. C. Chiu	2023	The Role of Exosomes in Pancreatic Ductal Adenocarcinoma Progression and Their Potential as Biomarkers	Cancers	Records excluded after screening

376	Hsu, T. K., T. Y. Liu, B. Gould, C. Decapite, A. Zureikat, A. Paniccia, E. Ariazi, M. Bertin, R. Bourgon, K. Coil, H. Donnella, A. Drake, J. M. Granka, P. Kaur, M. C. Louie, S. Mahajan, A. Pasupathy, O. Shapira, P. Ulz, C. Yang, C. J. Lin and R. Brand	2021	Plasma-based detection of pancreatic cancer: A multiomics approach	Cancer Research	Records excluded after screening
377	Hu, H., Q. Zhang, C. Huang, Y. Shen, X. Chen, X. Shi and W. Tang	2014	Diagnostic value of S100P for pancreatic cancer: a meta-analysis	Tumour Biol	Records excluded after screening
378	Hu, Y., D. Jones, A. K. Esnakula, S. G. Krishna and W. Chen	2024	Molecular Pathology of Pancreatic Cystic Lesions with a Focus on Malignant Progression	Cancers	Records excluded after screening
379	Hu, Y., Y. Zhu, W. Nie, J. Shi, X. Wei, C. Tang and W. Zhang	2023	Thioredoxin reductase as a novel biomarker for the diagnosis and efficacy prediction of gastrointestinal malignancy: a large-scale, retrospective study	International Journal of Clinical Oncology	Records excluded after screening
380	Hu, Z., J. Wu, S. Tan, L. Yang, J. Huang, C. Mo, H. Chen, X. Ruan and X. Qin	2022	Diagnostic value of long noncoding RNA LINC01485 in patients with colorectal cancer	Clin Biochem	Records excluded after screening
381	Hu, Z. I., J. C. Bendell, A. Bullock, N. K. LoConte, H. Hatoum, P. Ritch, H. Hool, J. W. Leach, J. Sanchez, D. P. S. Sohal and et al.	2019	A randomized phase II trial of nab-paclitaxel and gemcitabine with tarextumab or placebo in patients with untreated metastatic pancreatic cancer	Cancer medicine	Non-diagnostic studies
382	Hu, Z. J., J. R. Wu, S. L. Tan, L. J. Yang, J. H. Huang, C. J. Mo, H. P. Chen, X. L. Ruan and X. Qin	2022	Diagnostic value of long noncoding RNA LINC01485 in patients with colorectal cancer	Clinical Biochemistry	Records excluded after screening
383	Hua, Y., H. Chen, L. Wang, F. Wang, P. Wang, Z. Ning, Y. Li, L. Liu, Z. Chen and Z. Meng	2017	Low serum miR-373 predicts poor prognosis in patients with pancreatic cancer	Cancer Biomark	Not early-stage
384	Huang, B., Y. Liu, L. Hu and Z. Li	2024	Diagnostic biomarkers for chronic pancreatitis: research progress	Academic Journal of Naval Medical University	Records excluded after screening
385	Huang, J., G. Gao, Y. Ge, J. Liu, H. Cui, R. Zheng, J. Wang, S. Wang, V. L. Go, S. Hu, Y. Liu, M. Yang, Y. Sun, D. Shang, Y. Tian, Z. Zhang, Z. Xiang, H. Wang, J. Guo and G. G. Xiao	2024	Development of a Serum-Based MicroRNA Signature for Early Detection of Pancreatic Cancer: A Multicenter Cohort Study	Digestive Diseases and Sciences	Studies included in review

386	Huang, J., J. Liu, K. Chen-Xiao, X. Zhang, W. N. Paul Lee, V. L. W. Go and G. G. Xiao	2016	Advance in microRNA as a potential biomarker for early detection of pancreatic cancer	Biomarker Research	Records excluded after screening
387	Huang, J., A. C. Soupir, B. D. Schlick, M. Teng, I. H. Sahin, J. B. Permuth, E. M. Siegel, B. J. Manley, B. Pellini and L. Wang	2021	Cancer detection and classification by CpG island hypermethylation signatures in plasma cell-free DNA	Cancers	Records excluded after screening
388	Huang, L. Y.	2017	Abdominal pain, abnormal ca199 and new onset diabetes: The main clue to diagnosis of pancreatic cancer	Clinical Gastroenterology and Hepatology	Records excluded after screening
389	Huang, P., W. Gao, C. Fu and R. Tian	2023	Functional and Clinical Proteomic Exploration of Pancreatic Cancer	Molecular and Cellular Proteomics	Records excluded after screening
390	Huang, S. F., Z. L. Yang, D. Q. Li, Z. Y. Liu, C. W. Wang, X. Y. Miao, Q. Zou and Y. Yuan	2016	Jagged1 and DLL4 expressions in benign and malignant pancreatic lesions and their clinicopathological significance	Hepatobiliary Pancreat Dis Int	Non-diagnostic studies
391	Huang, W., L. Xue, H. Xu, Z. Kong, J. Xu, H. Zhao and Y. Nie	2021	Diagnostic value of neuronal pentraxin II methylation in patients with pancreatic cancer: Meta-analysis	International Journal of Clinical Practice	Records excluded after screening
392	Huang, X. D., F. J. Xiao, Y. T. Guo, Y. Sun, Y. K. Zhang and X. J. Shi	2022	Protein tyrosine phosphatase 1 protects human pancreatic cancer from erastin-induced ferroptosis	Asian Journal of Surgery	Records excluded after screening
393	Huang, Y., F. Chen, L. Zhang, Q. Lv, J. Yan and W. Cui	2021	MALDI-TOF-MS Analysis in the Discovery and Identification of the Serum Peptide Pattern of Pancreatic Ductal Adenocarcinoma	Lab Medicine	Not early-stage
394	Huang, Y., W. Zhang, Q. Li, Z. Wang and X. Yang	2023	Identification of m6A/m5C/m1A-associated LncRNAs for prognostic assessment and immunotherapy in pancreatic cancer	Scientific reports	Records excluded after screening
395	Huang, Z., W. Chen, Y. Du, Q. Guo, Y. Mao, X. Zhou and D. Hua	2019	Serum miR-16 as a potential biomarker for human cancer diagnosis: results from a large-scale population	Journal of Cancer Research and Clinical Oncology	Records excluded after screening
396	Huang, Z., Z. Jiang, C. Zhao, W. Han, L. Lin, A. Liu, S. Weng and X. Lin	2017	Simple and effective label-free electrochemical immunoassay for carbohydrate antigen 19-9 based on polythionine-Au composites as enhanced sensing signals for detecting different clinical samples	International Journal of Nanomedicine	Records excluded after screening
397	Huanga, Y. and Z. Fengb	2024	ASSESSING SCREENING EFFICACY IN THE PRESENCE OF CANCER OVERDIAGNOSIS	Annals of Applied Statistics	Records excluded after screening
398	Huerta, M., S. Roselló, L. Sabater, A. Ferrer, N. Tarazona, D. Roda, V. Gambardella, C. Alfaro-Cervelló, M. Garcés-Albir, A. Cervantes and M. Ibarrola-Villava	2021	Circulating tumor dna detection by digital-droplet pcr in pancreatic ductal adenocarcinoma: A systematic review	Cancers	Records excluded after screening

399	Huffman, K. E., L. S. Li, R. Carstens, H. Park, L. Girard, K. Avila, S. Wei, R. Kollipara, B. Timmons, J. Sudderth, N. Bendris, J. Kim, P. Villalobos, J. Fujimoto, S. Schmid, R. J. Deberardinis, I. Wistuba, J. Heymach, R. Kittler, E. A. Akbay, B. Posner, Y. Wang, S. Lam, S. A. Kliewer, D. J. Mangelsdorf and J. D. Minna	2023	Glucocorticoid mediated inhibition of LKB1 mutant non-small cell lung cancers	Frontiers in Oncology	Records excluded after screening
400	Humeau, M., A. Vignolle-Vidoni, F. Sicard, F. Martins, B. Bournet, L. Buscail, J. Torrisani and P. Cordelier	2015	Salivary MicroRNA in Pancreatic Cancer Patients	Plos One	Small sample size
401	Husi, H., M. Fernandes, R. J. Skipworth, J. Miller, A. D. Cronshaw, K. C. H. Fearon and J. A. Ross	2019	Identification of diagnostic upper gastrointestinal cancer tissue type specific urinary biomarkers	Biomedical Reports	Records excluded after screening
402	Husi, H., A. MacDonald, R. J. E. Skipworth, J. Miller, A. Cronshaw, C. Greig, K. C. H. Fearon and J. A. Ross	2018	Urinary diagnostic proteomic markers for dynapenia in cancer patients	Biomedical Reports	Records excluded after screening
403	Husi, H., R. J. E. Skipworth, A. Cronshaw, N. A. Stephens, H. Wackerhage, C. Greig, K. C. H. Fearon and J. A. Ross	2015	Programmed cell death 6 interacting protein (PDCD6IP) and Rabenosyn-5 (ZFYVE20) are potential urinary biomarkers for upper gastrointestinal cancer	Proteomics Clinical Applications	Records excluded after screening
404	Hussein, N. A., Z. A. Kholy, M. M. Anwar, M. A. Ahmad and S. M. Ahmad	2017	Plasma miR-22-3p, miR-642b-3p and miR-885-5p as diagnostic biomarkers for pancreatic cancer	J Cancer Res Clin Oncol	Not early-stage
405	Hussung, S., M. Follo, R. F. U. Klar, S. Michalczyk, K. Fritsch, F. Nollmann, J. Hipp, J. Duyster, F. Scherer, N. von Bubnoff, M. Boerries, U. Wittel and R. M. Fritsch	2020	Development and Clinical Validation of Discriminatory Multitarget Digital Droplet PCR Assays for the Detection of Hot Spot KRAS and NRAS Mutations in Cell-Free DNA	J Mol Diagn	Records excluded after screening
406	Hussung, S., K. Kö hler, N. Köhne, S. Michalczyk, J. Mastroianni, K. Fritsch, R. Zeiser, M. Boerries, U. Wittel and R. Fritsch	2017	Pancreatic cancer 3D organoids: A co-clinical platform for translational research and ex vivo drug testing	Oncology Research and Treatment	Records excluded after screening

407	Huynh, K. Q., A. T. Le, T. T. Phan, T. T. Ho, S. P. Pho, H. T. Nguyen, B. T. Le, T. T. Nguyen and S. T. Nguyen	2023	The Diagnostic Power of Circulating miR-1246 in Screening Cancer: An Updated Meta-analysis	Oxidative Medicine and Cellular Longevity	Records excluded after screening
408	Iacob, R., D. Paul and I. Popescu	2023	Editorial: The emerging role of liquid biopsy in gastrointestinal, pancreatic and liver cancers	Frontiers in Medicine	Records excluded after screening
409	Ideno, N., Y. Mori, M. Nakamura and T. Ohtsuka	2020	Early detection of pancreatic cancer: Role of biomarkers in pancreatic fluid samples	Diagnostics	Records excluded after screening
410	Ikemoto, J., M. Serikawa, Y. Ishii, T. Tsuboi, R. Kawamura, K. Tsushima, Y. Saito, T. Sekito, S. Nakamura, T. Hirano, A. Fukiage, Y. Kiyoshita, S. Saeki, Y. Tamura, Y. Murakami and K. Arihiro	2019	Clinical features and pathologic diagnosis strategy of early diagnosis of pancreatic cancer	Pancreas	Records excluded after screening
411	Ikeura, T., Y. Hori, T. Mitsuyama, H. Miyoshi, M. Shimatani, K. Uchida, M. Takaoka, U. Ota, A. Kamiya, K. Takahashi, M. Ishizuka, M. Kaibori and K. Okazaki	2020	Effectiveness of photodynamic screening using 5-aminolevulinic acid for the diagnosis of pancreatic cancer	Anticancer Research	Not early-stage
412	Illés, D., E. Ivány, G. Holzinger, K. Kosár, M. G. Adam, B. Kamlage, G. Zsóri, M. Tajti, M. M. Svébis, V. Horváth, I. Oláh, K. Márta, S. Váncsa, N. Zádori, A. Szentesi, B. Czakó, P. Hegyi and L. Czakó	2020	New Onset of DiabetEs in aSsociation with pancreatic ductal adenocarcinoma (NODES Trial): Protocol of a prospective, multicentre observational trial	BMJ Open	Records excluded after screening
413	Illés, D., E. Ivány, G. Holzinger, K. Kosár, G. Zsóri, M. Tajti and L. Czakó	2019	Early diagnosis of pancreatic cancer with metabolite biomarkers in new-onset diabetes mellitus-New Onset of Diabetes in the Elderly Study (NODES)	Pancreatology	Records excluded after screening
414	Illés, D., V. Terzin, G. Holzinger, K. Kosár, R. Róka, G. Zsóri, G. Ábrahám and L. Czakó	2016	New-onset type 2 diabetes mellitus--A high-risk group suitable for the screening of pancreatic cancer?	Pancreatology	Records excluded after screening
415	Imamura, T., S. Komatsu, D. Ichikawa, T. Kawaguchi, M. Miyamae, W. Okajima, T. Ohashi, T. Arita, H. Konishi, A. Shiozaki, R. Morimura, H. Ikoma, K. Okamoto and E. Otsuji	2016	Liquid biopsy in patients with pancreatic cancer: Circulating tumor cells and cell-free nucleic acids	World Journal of Gastroenterology	Records excluded after screening

416	Irie, S., Y. Mise and A. Saiura	2022	The Early Detection of Circulating Tumor Cells Using Telomerase-Selective Replicating Adenovirus in Pancreatic Cancer Patient	HPB	Records excluded after screening
417	Ishige, F., I. Hoshino, Y. Iwatate, S. Chiba, H. Arimitsu, H. Yanagibashi, H. Nagase and W. Takayama	2020	MIR1246 in body fluids as a biomarker for pancreatic cancer	Scientific Reports	Not early-stage
418	Israilov, S., H. J. Cho and M. Krouss	2022	Things We Do for No Reason™: Tumor markers CA125, CA19-9, and CEA in the initial diagnosis of malignancy	Journal of Hospital Medicine	Records excluded after screening
419	Italiano, A., S. Cousin, C. Bellera, J. P. Guegan, J. P. Metges, A. Adenis, R. Bahleda, P. Cassier, C. Cantarel, M. Kind, J. Palussiere, L. Vanhersecke and A. Bessede	2024	Avelumab combined with regorafenib in solid tumors with tertiary lymphoid structures: A phase 2 REGOMUNE trial cohort	Cancer Research	Records excluded after screening
420	Ivanova, I. I.	2018	May glypican-3 be a novel biomarker and potential therapeutic target in hepatocellular cancer?	Journal of IMAB - Annual Proceeding (Scientific Papers)	Records excluded after screening
421	Ivry, S. L., G. M. Knudsen, F. Caiazza, J. M. Sharib, K. Jaradeh, M. Ravalin, A. J. O'Donoghue, K. S. Kirkwood and C. S. Craik	2019	The lysosomal aminopeptidase tripeptidyl peptidase 1 displays increased activity in malignant pancreatic cysts	Biological Chemistry	Records excluded after screening
422	Iwai, K., T. Nambu, O. Kurasawa, N. Uchiyama, R. Dairiki, Y. Yamamoto, S. Nishizawa, M. Zhang, Y. Ishii, H. Niu and A. Ohashi	2017	Potential predictive biomarkers of clinical responses for a novel CDC7-selective inhibitor TAK-931	Cancer Research	Records excluded after screening
423	Iwano, T., K. Yoshimura, G. Watanabe, R. Saito, S. Kiritani, H. Kawaida, T. Moriguchi, T. Murata, K. Ogata, D. Ichikawa, J. Arita, K. Hasegawa and T. Sen	2021	High-performance Collective Biomarker from Liquid Biopsy for Diagnosis of Pancreatic Cancer Based on Mass Spectrometry and Machine Learning	Journal of Cancer	Records excluded after screening
424	Iyengar, D., K. Tatiparti, N. S. Gavande, S. Sau and A. K. Iyer	2022	Nanomedicine for overcoming therapeutic and diagnostic challenges associated with pancreatic cancer	Drug Discovery Today	Records excluded after screening
425	Jabbar, K. S., L. Arike, C. S. Verbeke, R. Sadik and G. C. Hansson	2018	Highly Accurate Identification of Cystic Precursor Lesions of Pancreatic Cancer Through Targeted Mass Spectrometry: A Phase IIc Diagnostic Study	J Clin Oncol	Not early-stage

426	Jackson, E. R. K., O. P. Pointon, R. Bohmer and J. R. Burgess	2017	Utility of FDG-PET imaging for risk stratification of pancreatic neuroendocrine tumors in MEN1	Journal of Clinical Endocrinology and Metabolism	Records excluded after screening
427	Jacobson, R. A., E. Munding, D. M. Hayden, M. Levy, T. M. Kuzel, S. G. Pappas and A. Masood	2019	Evolving clinical utility of liquid biopsy in gastrointestinal cancers	Cancers	Records excluded after screening
428	Jafari, A., K. Karimabadi, A. Rahimi, G. Rostaminasab, M. Khazaei, L. Rezakhani and T. Ahmadi jouybari	2023	The Emerging Role of Exosomal miRNAs as Biomarkers for Early Cancer Detection: A Comprehensive Literature Review	Technology in Cancer Research and Treatment	Records excluded after screening
429	Jahan, R., K. Ganguly, L. M. Smith, P. Atri, J. Carmicheal, Y. Sheinin, S. Rachagani, G. Natarajan, R. E. Brand, M. A. Macha, P. M. Grandgenett, S. Kaur and S. K. Batra	2019	Trefoil factor(s) and CA19.9: A promising panel for early detection of pancreatic cancer	Ebiomedicine	No sample information provided
430	Jang, J. Y., D. Y. Oh, S. W. Kim, W. Lee, J. S. Heo, C. M. Kang, J. Namkung, Y. Choi, S. Han, Y. Kim and T. Park	2016	Novel biomarker panel for the early detection of pancreatic cancer and its clinical validation	HPB	Records excluded after screening
431	Jang, S. I., H. K. Lee, E. J. Chang, S. Kim, S. Y. Kim, I. Y. Hong, J. K. Kim, H. S. Lee, J. Yang, J. H. Cho and D. K. Lee	2023	Improved predictability of pancreatic ductal adenocarcinoma diagnosis using a blood immune cell biomarker panel developed from bulk mRNA sequencing and single-cell RNA-sequencing	Cancer Immunology, Immunotherapy	Not early-stage
432	Jaride, M. and S. N. M. Firouzabadi	2021	Patient-Derived Xenograft (PDX) Models: A Step Forward in Personalized Medicine	Frontiers in Biomedical Technologies	Records excluded after screening
433	Javed, S., T. A. Qureshi, S. Gaddam, A. Wachsman, L. Azab, V. Asadpour, W. Chen, B. Wu, Y. Xie, S. Pandol and D. Li	2022	Predicting pancreatic cancer using artificial intelligence analysis of pancreatic subregions using computed tomography images	Cancer Research	Records excluded after screening
434	Javed, S., T. A. Qureshi, S. Gaddam, L. Wang, L. Azab, A. M. Wachsman, W. Chen, V. Asadpour, C. Y. Jeon, B. Wu, Y. Xie, S. J. Pandol and D. Li	2022	Risk prediction of pancreatic cancer using AI analysis of pancreatic subregions in computed tomography images	Frontiers in Oncology	No specific biomarkers provided

435	Javidi, M. A., A. H. Ahmadi, B. Bakhshinejad, N. Nouraee, S. Babashah and M. Sadeghizadeh	2014	Cell-free microRNAs as cancer biomarkers: the odyssey of miRNAs through body fluids	Medical Oncology	Records excluded after screening
436	Jaworski, J. J., R. D. Morgan and S. Sivakumar	2020	Circulating cell-free tumour DNA for early detection of pancreatic cancer	Cancers	Records excluded after screening
437	Jayant, K., S. Agrawal and R. Agrawal	2016	miRNA a real kid for early recognition for PANcreatic adenocarcinoma (MARKER PAN study)	Journal for ImmunoTherapy of Cancer	Records excluded after screening
438	Jeffrey, G. P., L. G. Gordon, M. M. Hill and G. A. Ramm	2020	Liquid Biopsies for Hepatocellular Cancer and Their Potential in Clinical Practice	Hepatology	Records excluded after screening
439	Jelski, W. and B. Mroczko	2019	Biochemical diagnostics of pancreatic cancer - Present and future	Clinica Chimica Acta	Records excluded after screening
440	Jenkinson, C., J. Earl, P. Ghaneh, C. Halloran, A. Carrato, W. Greenhalf, J. Neoptolemos and E. Costello	2015	Biomarkers for early diagnosis of pancreatic cancer	Expert Review of Gastroenterology and Hepatology	Records excluded after screening
441	Jenkinson, C., V. L. Elliott, A. Evans, L. Oldfield, R. E. Jenkins, D. P. O'Brien, S. Apostolidou, A. Gentry-Maharaj, E. O. Fourkala, I. J. Jacobs, U. Menon, T. Cox, F. Campbell, S. P. Pereira, D. A. Tuveson, B. K. Park, W. Greenhalf, R. Sutton, J. F. Timms, J. P. Neoptolemos and E. Costello	2016	Decreased serum thrombospondin-1 levels in pancreatic cancer patients up to 24 months prior to clinical diagnosis: Association with diabetes mellitus	Clinical Cancer Research	Incomplete diagnostic performance data provided
442	Jeong, S., J. Park, D. Pathania, C. M. Castro, R. Weissleder and H. Lee	2016	Integrated Magneto-Electrochemical Sensor for Exosome Analysis	Acs Nano	Records excluded after screening
443	Jia, E., N. Ren, X. Shi, R. Zhang, H. Yu, F. Yu, S. Qin and J. Xue	2022	Extracellular vesicle biomarkers for pancreatic cancer diagnosis: a systematic review and meta-analysis	BMC Cancer	Records excluded after screening
444	Jia, K., S. Kundrot, M. Palchuk, J. Warnick, K. Haapala, I. Kaplan, M. Rinard and L. Appelbaum	2023	Developing and validating a pancreatic cancer risk model for the general population using multi-institutional electronic health records from a federated network.		Records excluded after screening
445	Jiang, F., X. Yang, X. He and M. Yang	2019	Circulating DNA, a Potentially Sensitive and Specific Diagnostic Tool for Future Medicine	Dose-Response	Records excluded after screening
446	Jiang, X., D. Hou, Z. Wei, S. Zheng, Y. Zhang and J. Li	2019	Extracellular and intracellular micrornas in pancreatic cancer: From early diagnosis to reducing chemoresistance	ExRNA	Records excluded after screening

447	Jiang, X., Y. Ma, T. Wang, H. Zhou, K. Wang, W. Shi, L. Qin, J. Guan, L. Li, B. Long, J. Wang, X. Guan, H. Ye, J. Yang, Z. Yu and Z. Jiao	2023	Targeting UBE2T Potentiates Gemcitabine Efficacy in Pancreatic Cancer by Regulating Pyrimidine Metabolism and Replication Stress	Gastroenterology	Records excluded after screening
448	Jiao, B., R. Gulati, H. A. Katki, P. E. Castle and R. Etzioni	2022	A Quantitative Framework to Study Potential Benefits and Harms of Multi-Cancer Early Detection Testing	Cancer Epidemiology Biomarkers and Prevention	Records excluded after screening
449	Jiao, Y., Z. Fu, Y. Li, W. Zhang and Y. Liu	2019	Aberrant FAM64A mRNA expression is an independent predictor of poor survival in pancreatic cancer	PLoS One	Records excluded after screening
450	Jin, J., J. Cheng, N. Wang, L. Huang, X. Zhou, L. Chen, H. Luo, T. Liu and J. Meng	2022	MIP3α As an Early Prognostic Predictor for Patients with B-Cell Malignancies Receiving CD19/CD22-Redirected CAR T-Cell Cocktail Therapy	Blood	Records excluded after screening
451	Jin, S., S. S. Tan and H. Li	2015	MicroRNA-18a as a promising biomarker for cancer detection: A meta-analysis	International Journal of Clinical and Experimental Medicine	Records excluded after screening
452	Jin, X. L., B. Xu and Y. L. Wu	2014	Detection of pancreatic cancer with normal carbohydrate antigen 19-9 using protein chip technology	World J Gastroenterol	Incomplete diagnostic performance data provided
453	Johansen, J. S., D. Calatayud, V. Albieri, N. A. Schultz, C. Dehlendorff, J. Werner, B. V. Jensen, P. Pfeiffer, S. E. Bojesen, N. Giese, K. R. Nielsen, S. E. Nielsen, M. Yilmaz, N. H. Holländer and K. K. Andersen	2016	The potential diagnostic value of serum microRNA signature in patients with pancreatic cancer	International Journal of Cancer	Non-diagnostic studies
454	Jordaens, S., K. Zwaenepoel, W. Tjalma, C. Deben, K. Beyers, V. Vankerckhoven, P. Pauwels and A. Vorsters	2023	Urine biomarkers in cancer detection: A systematic review of preanalytical parameters and applied methods	International Journal of Cancer	Records excluded after screening
455	Jugniot, N., R. Bam, E. J. Meuillet, E. C. Unger and R. Paulmurugan	2021	Current status of targeted microbubbles in diagnostic molecular imaging of pancreatic cancer	Bioengineering and Translational Medicine	Records excluded after screening

456	Julich-Haertel, H., S. K. Urban, M. Krawczyk, A. Willms, K. Jankowski, W. Patkowski, B. Kruk, M. Krasnodębski, J. Ligocka, R. Schwab, I. Richardsen, S. Schaaf, A. Klein, S. Gehlert, H. Sänger, M. Casper, J. M. Banales, D. Schuppan, P. Milkiewicz, F. Lammert, M. Krawczyk, V. Lukacs-Kornek and M. Kornek	2017	Cancer-associated circulating large extracellular vesicles in cholangiocarcinoma and hepatocellular carcinoma	Journal of Hepatology	Records excluded after screening
457	Jung, K., I. H. Jung, J. Park, J. C. Lee, J. Kim and J. H. Hwang	2020	Serial Circulating Tumor DNA Monitoring to Evaluate Treatment Response in Metastatic Pancreatic Cancer	Gut and Liver	Records excluded after screening
458	Kaczor-Urbanowicz, K. E., J. Cheng, J. C. King, A. Sedarat, S. J. Pandol, J. J. Farrell, D. T. W. Wong and Y. Kim	2020	Reviews on Current Liquid Biopsy for Detection and Management of Pancreatic Cancers	Pancreas	Records excluded after screening
459	Kaczor-Urbanowicz, K. E., C. Martin Carreras-Presas, K. Aro, M. Tu, F. Garcia-Godoy and D. T. W. Wong	2017	Saliva diagnostics – Current views and directions	Experimental Biology and Medicine	Records excluded after screening
460	Kaczor-Urbanowicz, K. E., F. Wei, S. L. Rao, J. Kim, H. Shin, J. Cheng, M. Tu, D. T. W. Wong and Y. Kim	2019	Clinical validity of saliva and novel technology for cancer detection	Biochimica et Biophysica Acta - Reviews on Cancer	Records excluded after screening
461	Kadayifci, A., M. Atar, J. L. Wang, D. G. Forcione, B. W. Casey, M. B. Pitman and W. R. Brugge	2017	Value of adding GNAS testing to pancreatic cyst fluid KRAS and carcinoembryonic antigen analysis for the diagnosis of intraductal papillary mucinous neoplasms	Digestive Endoscopy	Non-diagnostic studies
462	Kaiser, J.	2018	'Liquid biopsy' for cancer promises early detection: Combining DNA and protein markers brings researchers closer to a universal cancer screening test	Science	Records excluded after screening
463	Kakaei, N., G. Mohammadi and Z. Izadi	2023	Application of Imaging Techniques in the Diagnosis and Treatment of Pancreatic Cancer	Frontiers in Biomedical Technologies	Records excluded after screening
464	Kalra, J., K. Laderoute, D. Renouf, D. Shaeffer and M. Bally	2016	Developing circRNA signatures as a biomarker for the early diagnosis of pancreatic carcinoma	Cancer Research	Records excluded after screening

465	Kamal, M. A., I. Siddiqui, C. Belgiovine, M. Barbagallo, V. Paleari, D. Pistillo, C. Chiabrando, S. Schiarea, B. Bottazzi, R. Leone, R. Avigni, R. Migliore, P. Spaggiari, F. Gavazzi, G. Capretti, F. Marchesi, A. Mantovani, A. Zerbi and P. Allavena	2022	Oncogenic KRAS-Induced Protein Signature in the Tumor Secretome Identifies Laminin-C2 and Pentraxin-3 as Useful Biomarkers for the Early Diagnosis of Pancreatic Cancer	Cancers	Not early-stage
466	Kamyabi, N., V. Bernard and A. Maitra	2019	Liquid biopsies in pancreatic cancer	Expert Review of Anticancer Therapy	Records excluded after screening
467	Kandel, P., C. W. Wu, B. A. Dukek, P. H. Foote, W. R. Taylor, T. C. Yab, D. W. Mahoney, K. N. Burger, K. Doering, J. R. Arndt, J. B. Kisiel, M. B. Wallace, D. A. Ahlquist and S. Majumder	2019	ADDITION OF PRESERVATIVE BUFFER IMPROVES DNA YIELD FROM ENDOSCOPICALLY COLLECTED PANCREATIC JUICE AND FACILITATES DETECTION OF PANCREATIC DUCTAL ADENOCARCINOMA USING METHYLATED DNA MARKERS	Gastroenterology	Records excluded after screening
468	Kandimalla, R., J. Xu, A. Link, T. Matsuyama, K. Yamamura, I. Parker, H. Uetake, E. Hernandez-Illan, J. Lozano, E. Borazanci, S. Tsai, D. Evans, S. J. Meltzer, H. Baba, R. Brand, D. Von Hoff, F. Balaguer, W. Li and A. Goel	2020	EpiPanGI-Dx: A cell-free DNA methylation fingerprint for the early detection of gastrointestinal cancers	Cancer Research	Records excluded after screening
469	Kandimalla, R., J. Xu, A. Link, T. Matsuyama, K. Yamamura, M. I. Parker, H. Uetake, F. Balaguer, E. Borazanci, S. Tsai, D. Evans, S. J. Meltzer, H. Baba, R. Brand, D. Von Hoff, W. Li and A. Goe	2021	EpiPanGI Dx: A Cell-free DNA Methylation fingerprint for the early detection of Gastrointestinal cancers	Clinical Cancer Research	Non-diagnostic studies
470	Kane, L. E., G. S. Mellotte, E. Mylod, R. O'Brien, F. O'Connell, K. Nguyen, C. E. Buckley, J. Arlow, D. Mockler, A. D. Meade, B. M. Ryan and S. G. Maher	2021	Diagnostic accuracy of blood-based multi-omic biomarkers for pancreatic adenocarcinoma: A systematic review and meta-analysis	Cancer Research	Records excluded after screening

471	Kane, L. E., G. S. Mellotte, E. Mylod, R. M. O'Brien, F. O'Connell, C. E. Buckley, J. Arlow, K. Nguyen, D. Mockler, A. D. Meade, B. M. Ryan and S. G. Maher	2022	Diagnostic Accuracy of Blood-based Biomarkers for Pancreatic Cancer: A Systematic Review and Meta-analysis	Cancer Res Commun	Records excluded after screening
472	Kanesvaran, R., P. L. Chia, E. Chiong, M. L. K. Chua, N. T. Ngo, S. Ow, H. G. Sim, M. H. Tan, K. H. Tay, A. S. C. Wong, S. W. Wong and P. H. Tan	2023	An approach to genetic testing in patients with metastatic castration-resistant prostate cancer in Singapore	Annals of the Academy of Medicine Singapore	Records excluded after screening
473	Kang, J. S., S. Y. Hong, Y. Han, H. J. Sohn, M. Lee, Y. H. Kang, H. S. Kim, H. Kim, W. Kwon and J. Y. Jang	2021	Limits of serum carcinoembryonic antigen and carbohydrate antigen 19-9 as the diagnosis of gallbladder cancer	Annals of Surgical Treatment and Research	Records excluded after screening
474	Kang, M., W. Qin, M. Buya, X. Dong, W. Zheng, W. Lu, J. Chen, Q. Guo and Y. Wu	2016	VNN1, a potential biomarker for pancreatic cancer-associated new-onset diabetes, aggravates paraneoplastic islet dysfunction by increasing oxidative stress	Cancer Letters	Records excluded after screening
475	Kannan, S., P. S. S. Ali and A. Sheeza	2022	Short report - Lethal and aggressive pancreatic cancer: molecular pathogenesis, cellular heterogeneity, and biomarkers of pancreatic ductal adenocarcinoma	European Review for Medical and Pharmacological Sciences	Records excluded after screening
476	Kanwar, S. S., C. J. Dunlay, D. M. Simeone and S. Nagrath	2014	Simultaneous isolation and quantification of circulating exosomes for cancer biomarker discovery	Cancer Research	Records excluded after screening
477	Karar, M. E., N. El-Fishawy and M. Radad	2023	Automated classification of urine biomarkers to diagnose pancreatic cancer using 1-D convolutional neural networks	Journal of Biological Engineering	Not early-stage
478	Karimi, F., H. Karimi-Maleh, J. Rouhi, N. Zare, C. Karaman, M. Baghayeri, L. Fu, S. Rostamnia, E. N. Dragoi, A. Ayati and P. Krivoshapkin	2023	Revolutionizing cancer monitoring with carbon-based electrochemical biosensors	Environmental Research	Records excluded after screening

479	Kartal, E., T. S. B. Schmidt, E. Molina-Montes, S. Rodríguez-Perales, J. Wirbel, O. M. Maistrenko, W. A. Akanni, B. Alashkar Alhamwe, R. J. Alves, A. Carrato, H. P. Erasmus, L. Estudillo, F. Finkelmeier, A. Fullam, A. M. Glazek, P. Gómez-Rubio, R. Hercog, F. Jung, S. Kandels, S. Kersting, M. Langheinrich, M. Márquez, X. Molero, A. Orakov, T. Van Rossum, R. Torres-Ruiz, A. Telzerow, K. Zych, V. Benes, G. Zeller, J. Trebicka, F. X. Real, N. Malats and P. Bork	2022	A faecal microbiota signature with high specificity for pancreatic cancer	Gut	Not early-stage
480	Kashiro, A., M. Kobayashi, T. Oh, M. Miyamoto, J. Atsumi, K. Nagashima, K. Takeuchi, S. Nara, S. Hijioka, C. Morizane, S. Kikuchi, S. Kato, K. Kato, H. Ochiai, D. Obata, Y. Shizume, H. Konishi, Y. Nomura, K. Matsuyama, C. Xie, C. Wong, Y. Huang, G. Jung, S. Srivastava, H. Kutsumi and K. Honda	2024	Clinical development of a blood biomarker using apolipoprotein-A2 isoforms for early detection of pancreatic cancer	Journal of Gastroenterology	Studies included in review
481	Kashiro, A., M. Kobayashi, T. Oh, K. Nagashima, K. Takeuchi, S. Nara, S. Hijioka, C. Morizane, S. Kikuchi, S. Kato, K. Kato, H. Ochiai, D. Obata, H. Konishi, K. Matsuyama, H. Kutsumi and K. Honda	2023	Clinical development of a blood biomarker using apolipoprotein-A2 isoforms for early detection of pancreatic cancer	Pancreatology	Records excluded after screening
482	Kasi, P. M., L. Shen, P. Ramachandran, K. Moser, G. Xu, P. Buckley, D. Serie, C. Lebrilla, H. Xu and C. H. F. Chan	2020	Serum glycoproteomic-based liquid biopsy for the detection of pancreatic ductal adenocarcinoma	Journal of Clinical Oncology	Records excluded after screening
483	Kastan, J. P., O. Standring, P. Cifani, V. Gaeth, A. Jensen, Y. Park and D. Tuveson	2024	Secretome labeling identifies gelsolin as a potential pro-tumorigenic PDAC biomarker	Cancer Research	Records excluded after screening

484	Kasuga, A., T. Okamoto, S. Udagawa, C. Mori, T. Mie, T. Furukawa, Y. Yamada, T. Takeda, M. Matsuyama, T. Sasaki, M. Ozaka, A. Ueki and N. Sasahira	2022	Molecular Features and Clinical Management of Hereditary Pancreatic Cancer Syndromes and Familial Pancreatic Cancer	International Journal of Molecular Sciences	Records excluded after screening
485	Katchman, B. A., R. Alam, G. Wallstrom, J. LaBaer, M. A. Hollingsworth, D. W. Cramer and K. S. Anderson	2014	Identification of autoantibody biomarkers to wild-type and mutant p53 in pancreatic and ovarian cancer	Cancer Research	Records excluded after screening
486	Katchman, B. A., R. Barderas, R. Alam, D. Chowell, M. S. Field, L. J. Esserman, G. Wallstrom, J. LaBaer, D. W. Cramer, M. A. Hollingsworth and K. S. Anderson	2016	Proteomic mapping of p53 immunogenicity in pancreatic, ovarian, and breast cancers	Proteomics - Clinical Applications	Not early-stage
487	Katerov, S., A. Vaccaro, J. Hennek, J. Carlson, W. R. Taylor, D. Mahoney, J. B. Kisiel and H. T. Allawi	2021	Accurate multi-cancer detection using methylated DNA markers and proteins in plasma	Cancer Research	Records excluded after screening
488	Kawada, T.	2019	Biomarkers for screening of pancreatic cancer: A risk assessment	International Journal of Cancer	Records excluded after screening
489	Kenner, B. J., S. T. Chari, D. F. Cleeter and V. L. Go	2015	Early detection of sporadic pancreatic cancer: strategic map for innovation--a white paper	Pancreas	Records excluded after screening
490	Khakoo, S., A. Petrillo, M. Salati, A. Muhith, J. Evangelista, S. Seghezzi, F. Petrelli, G. Tomasello and M. Ghidini	2021	Neoadjuvant treatment for pancreatic adenocarcinoma: A false promise or an opportunity to improve outcome?	Cancers	Records excluded after screening
491	Khaledian, E., P. Prasad, J. Blume, G. Bundalian, C. Chao-Shern, J. Choi, R. Cuaresma, J. Deyarmin, J. Heok Jang, M. Khadka, T. Khin, Y. Kodama, A. Kokate, J. Y. Lee, M. Liu, N. Mudaliar, M. Ramaiah, S. Ramaswamy, P. Spiro, K. Swaminathan, P. Williams, M. Yang, J. Yee, B. Young, R. Zawada, S. Zhang, C. Belthangady, B. Wilcox and P. Ma	2022	High-dimensional, multi-omics analyses of proteins, metabolites, transcripts, and genes enable biomarker discovery in early- and late-stage pancreatic cancer	Cancer Research	Records excluded after screening
492	Khan, H., M. R. Shah, J. Barek and M. I. Malik	2023	Cancer biomarkers and their biosensors: A comprehensive review	TrAC - Trends in Analytical Chemistry	Records excluded after screening

493	Khan, I. A., S. Gupta, S. Rashid, R. Yadav, N. Singh, B. Garg, D. Gunjan, N. R. Das, P. Das and A. Saraya	2019	MicroRNA expression profiles to differentiate pancreatic cancer from normal pancreas and chronic pancreatitis	Journal of Gastroenterology and Hepatology	Records excluded after screening
494	Khan, I. A., S. Rashid, N. Singh, S. Rashid, V. Singh, D. Gunjan, P. Das, N. R. Dash, R. M. Pandey, S. S. Chauhan, S. Gupta and A. Saraya	2021	Panel of serum miRNAs as potential non-invasive biomarkers for pancreatic ductal adenocarcinoma	Sci Rep	Incomplete diagnostic performance data provided
495	Khan, M. Z. I., S. Y. Tam and H. K. W. Law	2022	Advances in High Throughput Proteomics Profiling in Establishing Potential Biomarkers for Gastrointestinal Cancer	Cells	Records excluded after screening
496	Khan, R., Q. Nai, P. Zhang, H. Luo, S. Sen, I. Sidhom, T. Mathew, M. Islam, S. Sen and A. Yousif	2016	Increased erythropoietin levels as a biomarker of pancreatic adenocarcinoma: A case report	Molecular and Clinical Oncology	Records excluded after screening
497	Khatri, I. and M. K. Bhasin	2020	A Transcriptomics-Based Meta-Analysis Combined With Machine Learning Identifies a Secretory Biomarker Panel for Diagnosis of Pancreatic Adenocarcinoma	Frontiers in Genetics	Records excluded after screening
498	Kianfard, Z. and S. A. Sabatinos	2016	Board Number: B481 Studying the genetic requirements of gemcitabine resistance in fission yeast	Molecular Biology of the Cell	Records excluded after screening
499	Kiczmer, P., A. P. Senkowska, B. Szydło, E. Swietochowska and Z. Ostrowska	2017	Assessing the merits of existing pancreatic cancer biomarkers	Nowotwory	Records excluded after screening
500	Kiio, L. K., J. O. Onyatta, P. M. Ndangili, F. Oloo, C. Santamaria, L. M. Montuenga and D. N. Mbui	2024	Ultrasensitive immunosensor for multiplex detection of cancer biomarkers carcinoembryonic antigen (CEA) and yamaguchi sarcoma viral oncogene homolog 1 (YES1) based on eco-friendly synthesized gold nanoparticles	Talanta	Records excluded after screening
501	Kim, A. K., J. P. Hamilton, S. Y. Lin, T. T. Chang, H. W. Hann, C. T. Hu, Y. Lou, Y. J. Lin, T. P. Gade, G. Park, H. Luu, T. J. Lee, J. Wang, D. Chen, M. G. Goggins, S. Jain, W. Song and Y. H. Su	2022	Urine DNA biomarkers for hepatocellular carcinoma screening	British Journal of Cancer	Records excluded after screening
502	Kim, H., F. Civitci, J. Wagner, P. Anur, M. Rames, X. Nan, T. Morgan and T. Ngo	2019	Liquid biopsy for early cancer detection	Cancer Research	Records excluded after screening
503	Kim, H., K. N. Kang, Y. S. Shin, Y. Byun, Y. Han, W. Kwon, C. W. Kim and J. Y. Jang	2020	Biomarker Panel for the Diagnosis of Pancreatic Ductal Adenocarcinoma	Cancers	Studies included in review

504	Kim, H. S., Y. Han, J. S. Kang, Y. H. Kang, M. Lee, H. J. Sohn, H. Kim, W. Kwon and J. Y. Jang	2022	Limited usefulness of serum carcinoembryonic antigen and carbohydrate antigen 19-9 as preoperative diagnostic biomarkers of extrahepatic bile duct cancer	Journal of Hepato-Biliary-Pancreatic Sciences	Records excluded after screening
505	Kim, J., W. R. Bamlet, A. L. Oberg, K. G. Chaffee, G. Donahue, X. J. Cao, S. Chari, B. A. Garcia, G. M. Petersen and K. S. Zaret	2017	Detection of early pancreatic ductal adenocarcinoma with thrombospondin-2 & CA19-9 blood markers	Science Translational Medicine	Not early-stage
506	Kim, J. S., J. W. Choe, H. J. Kim, M. K. Joo, J. J. Park and Y. T. Bak	2014	Serum CA 19-9 as screening test for pancreatic cancer in new onset diabetic patients	United European Gastroenterology Journal	Records excluded after screening
507	Kim, J. W., K. J. Lee, S. K. Cho and G. S. Lee	2018	Complementary Role of Serum DUPAN-2 for Screening of Carbohydrate Antigen 19-9 in Patients with Pancreatico-Biliary Malignancy	Gut and Liver	Records excluded after screening
508	Kim, M. W., H. Koh, J. Y. Kim, S. Lee, H. Lee, Y. Kim, H. K. Hwang and S. I. Kim	2021	Tumor-Specific miRNA Signatures in Combination with CA19-9 for Liquid Biopsy-Based Detection of PDAC	International Journal of Molecular Sciences	Small sample size
509	Kim, Y., U. B. Kang, S. Kim, H. B. Lee, J. Jung, H. K. Kim, H. G. Moon, W. Han and D. Y. Noh	2019	Mastocheck: Notable plasma protein biomarker for diagnosis of breast cancer in the real clinical practice by using multiple reaction monitoring-based mass spectrometry	Journal of Clinical Oncology	Records excluded after screening
510	Kim, Y., U. B. Kang, S. Kim, H. B. Lee, H. G. Moon, W. Han and D. Y. Noh	2019	A validation study of a multiple reaction monitoring-based proteomic assay to diagnose breast cancer	Journal of Breast Cancer	Records excluded after screening
511	Kim, Y., D. Kim, B. Cao, R. Carvajal and M. Kim	2020	PDXGEM: patient-derived tumor xenograft-based gene expression model for predicting clinical response to anticancer therapy in cancer patients	BMC bioinformatics	Records excluded after screening
512	Kim, Y. J., H. S. Lee, D. E. Jung, J. M. Kim and S. Y. Song	2017	The DNA aptamer binds stemness-enriched cancer cells in pancreatic cancer	Journal of molecular recognition : JMR	Records excluded after screening
513	Kim, Y. J., W. Y. Rho, S. M. Park and B. H. Jun	2024	Optical nanomaterial-based detection of biomarkers in liquid biopsy	Journal of Hematology and Oncology	Records excluded after screening
514	Kishikawa, T., M. Otsuka, M. Ohno, T. Yoshikawa, A. Takata and K. Koike	2015	Circulating RNAs as new biomarkers for detecting pancreatic cancer	World J Gastroenterol	Records excluded after screening
515	Kisiel, J. B.	2015	The expanding scope of early gastrointestinal tract cancer diagnosis: Process and success of a methylomics approach	Oncologist	Records excluded after screening
516	Kisiel, J. B., N. Papadopoulos, M. C. Liu, D. Crosby, S. Srivastava and E. T. Hawk	2022	Multicancer early detection test: Preclinical, translational, and clinical evidence–generation plan and provocative questions	Cancer	Records excluded after screening

517	Kitagawa, T., K. Taniuchi, M. Tsuboi, M. Sakaguchi, T. Kohsaki, T. Okabayashi and T. Saibara	2019	Circulating pancreatic cancer exosomal RNAs for detection of pancreatic cancer	Mol Oncol	Small sample size
518	Kitagawa, Y., K. Okumura, T. Watanabe, K. Tsukamoto, S. Kitano, R. Nankinzan, T. Suzuki, T. Hara, H. Soda, T. Denda, T. Yamaguchi and H. Nagase	2019	Enrichment technique to allow early detection and monitor emergence of <i>KRAS</i> mutation in response to treatment	Scientific Reports	Records excluded after screening
519	Kladi-Skandali, A., K. Michaelidou, A. Scorilas and K. Mavridis	2015	Long noncoding RNAs in digestive system malignancies: A novel class of cancer biomarkers and therapeutic targets?	Gastroenterology Research and Practice	Records excluded after screening
520	Kobayashi, M., A. Fujita, T. Ogawa, Y. Tanisaka, M. Mizuide, N. Kondo, Y. Imaizumi, T. Hirotsu and S. Ryozawa	2021	Caenorhabditis elegans as a Diagnostic Aid for Pancreatic Cancer	Pancreas	No specific biomarkers provided
521	Kobayashi, T., Y. Hirata, M. Yoshida, S. Nishiumi and K. Honda	2017	Validation of serum/plasma metabolomic biomarkers against pancreatic cancer by quantitative targeted GC/MS/MS	United European Gastroenterology Journal	Records excluded after screening
522	Kobayashi, T., Y. Sato, S. Nishiumi, Y. Kodama, K. Nagashima and K. Honda	2022	Prospective Study of Plasma Apolipoprotein A2-ATQ/AT to Screen for High-Risk Individuals of Pancreatic Cancer	Pancreatology	Records excluded after screening
523	Koh, E. Y., J. H. Jeong, H. S. Ahn, K. Kim and E. Jun	2024	Identifying of a novel diagnostic markers for pancreatic neuroendocrine tumors by proteomics with patient blood	Cancer Research	Records excluded after screening
524	Kokabi, M., M. N. Tahir, D. Singh and M. Javanmard	2023	Advancing Healthcare: Synergizing Biosensors and Machine Learning for Early Cancer Diagnosis	Biosensors	Records excluded after screening
525	Komatsu, S., D. Ichikawa, T. Imamura, J. Kiuchi, K. Nishibeppu, W. Okajima, M. Miyamae, H. Konishi, R. Morimura, A. Shiozaki, H. Ikoma, H. Taniguchi and E. Otsuji	2018	Future perspective of liquid biopsy using microRNAs in blood of patients with hepatobiliary and pancreatic neoplasms	Cancer Science	Records excluded after screening
526	Komatsu, S., D. Ichikawa, H. Takeshita, R. Morimura, S. Hirajima, M. Tsujiura, T. Kawaguchi, M. Miyamae, H. Nagata, H. Konishi, A. Shiozaki and E. Otsuji	2014	Circulating miR-18a: A sensitive cancer screening biomarker in human cancer	In Vivo	Records excluded after screening

527	Konno, N., R. Suzuki, T. Takagi, M. Sugimoto, H. Asama, Y. Sato, H. Irie, T. Hikichi and H. Ohira	2021	Clinical utility of a newly developed microfluidic device for detecting circulating tumor cells in the blood of patients with pancreatico-biliary malignancies	Journal of Hepato-Biliary-Pancreatic Sciences	Records excluded after screening
528	Koopmann, B. D., N. Dunnewind, I. Lansdorp-Vogelaar, M. J. Bruno, D. L. Cahen and I. M. De Kok	2021	A model based analysis on the maximum effect of pancreatic cyst surveillance	United European Gastroenterology Journal	Records excluded after screening
529	Korfiatis, P., G. Suman, N. G. Patnam, K. H. Trivedi, A. Karbhari, S. Mukherjee, C. Cook, J. R. Klug, A. Patra, H. Khasawneh, N. Rajamohan, J. G. Fletcher, M. J. Truty, S. Majumder, C. W. Bolan, K. Sandrasegaran, S. T. Chari and A. H. Goenka	2023	Automated Artificial Intelligence Model Trained on a Large Data Set Can Detect Pancreas Cancer on Diagnostic Computed Tomography Scans As Well As Visually Occult Preinvasive Cancer on Prediagnostic Computed Tomography Scans	Gastroenterology	No sample information provided
530	Kouhestani, D., A. Mittelstädt, P. David, M. Podolska, A. Benard, I. Swierzy, L. Roßdeutsch, M. Brunner, A. Jacobsen, R. Gruetzmann and G. Weber	2022	Combined Exosomal CD40 and CD25 as Potential Liquid Biopsy Markers for the Detection of Gastrointestinal Cancers	European Surgical Research	Records excluded after screening
531	Koukaki, T., I. Balgkouranidou, E. Biziota, A. Karayiannakis, H. Bolanaki, E. Karamitrousis, P. Zarogoulidis, S. Deftereos, C. Charalampidis, A. Ioannidis, D. Matthaios, K. Amarantidis and S. Kakolyris	2024	Prognostic significance of BRCA1 and BRCA2 methylation status in circulating cell-free DNA of Pancreatic Cancer patients	Journal of Cancer	Incomplete diagnostic performance data provided
532	Koustoulidou, S., J. C. Knight, V. Kersemans, E. O'Neill and B. Cornelissen	2016	Imaging p53 in Pancreatic Ductal Adenocarcinoma (PDAC)	European Journal of Nuclear Medicine and Molecular Imaging	Records excluded after screening
533	Kowada, A.	2020	Cost-effectiveness of Abdominal Ultrasound Versus Magnetic Resonance Imaging for Pancreatic Cancer Screening in Familial High-Risk Individuals in Japan	Pancreas	Records excluded after screening

534	Krasnoslobodtsev, A. V., M. P. Torres, S. Kaur, I. V. Vlassiouk, R. J. Lipert, M. Jain, S. K. Batra and Y. L. Lyubchenko	2015	Nano-immunoassay with improved performance for detection of cancer biomarkers	Nanomedicine-Nanotechnology Biology and Medicine	Records excluded after screening
535	Krishna, S., A. Abdelbaki, P. A. Hart and J. D. Machicado	2024	Endoscopic Ultrasound-Guided Needle-Based Confocal Endomicroscopy as a Diagnostic Imaging Biomarker for Intraductal Papillary Mucinous Neoplasms	Cancers	Records excluded after screening
536	Krishna, S., A. McClelland and T. H. Zeng	2023	Nano Sensing for Early Diagnosis of Pancreatic Cancer.		Records excluded after screening
537	Kriz, D., D. Ansari and R. Andersson	2020	Potential biomarkers for early detection of pancreatic ductal adenocarcinoma	Clinical and Translational Oncology	Records excluded after screening
538	Kruger, D., Y. Y. Yako, J. Devar, N. Lahoud and M. Smith	2019	Inflammatory cytokines and combined biomarker panels in pancreatic ductal adenocarcinoma: Enhancing diagnostic accuracy	PLoS ONE	Non-diagnostic studies
539	Krulikas, L. J., I. M. McDonald, B. Lee, D. O. Okumu, M. P. East, T. S. K. Gilbert, L. E. Herring, B. T. Golitz, C. I. Wells, A. D. Axtman, W. J. Zuercher, T. M. Willson, D. Kireev, J. J. Yeh, G. L. Johnson, A. T. Baines and L. M. Graves	2018	Application of Integrated Drug Screening/Kinome Analysis to Identify Inhibitors of Gemcitabine-Resistant Pancreatic Cancer Cell Growth	SLAS Discov	Records excluded after screening
540	Kuang, J., L. Wang, Y. Yin, W. Shen, C. Liu, H. K. Lee and S. Tang	2023	Spatial Confinement of Single-Drop System to Enhance Aggregation-Induced Emission for Detection of MicroRNAs	Anal Chem	Records excluded after screening
541	Kumar, V., A. K. Chaudhary, Y. Dong, H. A. Zhong, G. Mondal, F. Lin, V. Kumar and R. I. Mahato	2017	Design, Synthesis and Biological Evaluation of novel Hedgehog Inhibitors for treating Pancreatic Cancer	Scientific reports	Records excluded after screening
542	Kumar, V., S. Gupta, A. Chaurasia and M. Sachan	2021	Evaluation of Diagnostic Potential of Epigenetically Deregulated MiRNAs in Epithelial Ovarian Cancer	Frontiers in Oncology	Records excluded after screening
543	Kumbasar, U., Z. G. Dikmen, Y. Yilmaz, B. Ancin, E. Dikmen and R. Dogan	2017	HE4 as a diagnostic and follow-up biomarker in non- small cell lung cancer	Clinical Chemistry	Records excluded after screening
544	Kunovsky, L., P. Tesarikova, Z. Kala, R. Kroupa, P. Kysela, J. Dolina and J. Trna	2018	The Use of Biomarkers in Early Diagnostics of Pancreatic Cancer	Canadian Journal of Gastroenterology and Hepatology	Records excluded after screening
545	Kupfer, S. S., S. Gupta, J. N. Weitzel and J. Samadder	2020	AGA Clinical Practice Update on Colorectal and Pancreatic Cancer Risk and Screening in BRCA1 and BRCA2 Carriers: Commentary	Gastroenterology	Records excluded after screening
546	Kurniali, P. C., M. H. Storandt and Z. Jin	2023	Utilization of Circulating Tumor Cells in the Management of Solid Tumors	Journal of Personalized Medicine	Records excluded after screening

547	Kusakabe, M., M. Sato, Y. Nakamura, H. Mikami, J. Lin and H. Nagase	2021	Elemental analysis by Metallobalance provides a complementary support layer over existing blood biochemistry panel-based cancer risk assessment	PeerJ	Records excluded after screening
548	Kusumoto, H., K. Tashiro, S. Shimaoka, K. Tsukasa, Y. Baba, S. Furukawa, J. Furukawa, T. Niihara, T. Hirotsu and T. Uozumi	2020	Efficiency of gastrointestinal cancer detection by nematode-NOSE (N-NOSE)	In Vivo	Records excluded after screening
549	Kuttanchettiyar, K. G., V. K. V and M. M. Chisthi	2024	Cyfra 21.1: A Useful Tumour Marker in Pancreatic Ductal Adenocarcinoma: Cross-Sectional Study	Cancer Control	Records excluded after screening
550	Kuvendjiska, J., F. Mueller, P. Bronsert, S. Timme-Bronsert, S. Fichtner-Feigl and B. Kulemann	2023	Circulating Epithelial Cells in Patients with Intraductal Papillary Mucinous Neoplasm of the Pancreas	Life-Basel	Records excluded after screening
551	Laderoute, K., D. Renouf, D. Shaeffer, M. Bally, E. Guns and J. Kalra	2017	Characterisation of exosomes and exosomal circular RNA from pancreatic ductal adenocarcinoma carcinoma cell lines	Journal of Extracellular Vesicles	Records excluded after screening
552	Laeseke, P. F., R. Chen, R. B. Jeffrey, T. A. Brentnall and J. K. Willmann	2015	Combining in vitro diagnostics with in vivo imaging for earlier detection of pancreatic ductal adenocarcinoma: Challenges and solutions1	Radiology	Records excluded after screening
553	Lai, L., X. Chen, G. Tian, R. Liang, X. Chen, Y. Qin, K. Chen and X. Zhu	2022	Clinical Significance of Pim-1 in Human Cancers: A Meta-analysis of Association with Prognosis and Clinicopathological Characteristics	Cancer Control	Records excluded after screening
554	Lam, R. C. T., D. Johnson, G. Lam, M. L. Y. Li, J. W. L. Wong, W. K. J. Lam, K. C. A. Chan and B. Ma	2022	Clinical applications of circulating tumor-derived DNA in the management of gastrointestinal cancers – current evidence and future directions	Frontiers in Oncology	Records excluded after screening
555	Lane, J. S., D. Von Hoff, D. Cridebring and A. Goel	2020	Extracellular Vesicles in Diagnosis and Treatment of Pancreatic Cancer: Current State and Future Perspectives	Cancers	Records excluded after screening
556	Larsen, E. K., M. Amrutkar, M. Aasrum, P. Sántha, C. S. Verbeke and I. P. Gladhaug	2019	Establishment and characterization of human PDAC-derived primary cancer cell lines	Pancreatology	Records excluded after screening
557	Lawson, P., N. Nissan, R. Faermann, O. Halshtok, A. Shalmon, M. Gotleib, M. A. B. David and M. S. Levy	2019	Trends in imaging workup of the male breast: Experience from a single center	Israel Medical Association Journal	Records excluded after screening
558	Lee, B., J. Cohen, L. R. Lipton, J. Tie, A. A. Javed, L. Li, D. Goldstein, P. Cooray, A. Nagrial, M. E. Burge and et al.	2017	Potential role of circulating tumor DNA (ctDNA) in the early diagnosis and postoperative management of localised pancreatic cancer	Journal of clinical oncology	Records excluded after screening

559	Lee, C. H., K. B. Yoo, B. S. Choung, S. Y. Seo, S. H. Kim, S. W. Kim, S. T. Lee, I. H. Kim, D. G. Kim and S. O. Lee	2014	Combination analysis of tumor markers with a higher cut-off value can increase the diagnostic rate of malignancies in a health screening	Journal of Gastroenterology and Hepatology (Australia)	Records excluded after screening
560	Lee, D. H., W. Yoon, A. Lee, Y. Han, Y. Byun, J. S. Kang, H. Kim, W. Kwon, Y. A. Suh, Y. Choi, J. Namkung, S. Han, S. G. Yi, J. S. Heo, I. W. Han, J. O. Park, J. K. Park, S. C. Kim, E. Jun, C. M. Kang, W. J. Lee, H. K. Lee, H. Lee, S. Lee, S. Y. Jeong, K. E. Lee, W. Han, T. Park and J. Y. Jang	2023	Multi-biomarker panel prediction model for diagnosis of pancreatic cancer	Journal of Hepato-Biliary-Pancreatic Sciences	Studies included in review
561	Lee, H. J., S. Y. Kim, D. W. Kim, Y. S. Park, J. H. Hwang, S. K. Cho and J. Y. Cho	2022	Serum protein profiling of lung, pancreatic, and colorectal cancers reveals alcohol consumption-mediated disruptions in early-stage cancer detection	Heliyon	Records excluded after screening
562	Lee, H. J., Y. J. Lee, J. Y. Lee, K. S. Park and K. B. Cho	2017	Clinical Value of High Elevated Serum Levels of CA 19-9 and Role of Abdominal Ultrasonography in Health Check-up Populations	Gut and Liver	Records excluded after screening
563	Lee, J., K. Jung, J. Ahn, B. Kim, S. Yang, J. C. Lee, J. Kim and J. H. Hwang	2023	Serial Circulating Tumor DNA Monitoring as a Predictive Biomarker for Progression and Recurrence in Patients with Pancreatic Cancer	Gut and Liver	Records excluded after screening
564	Lee, J. C., H. S. Kim, E. J. Sim, H. Cho, Y. Yoon, K. Jung, J. H. Jung, B. Kim, J. Ahn, J. Kim, J. Kim and J. H. Hwang	2022	A Novel mRNA-Based Immunological Biomarker for Early Diagnosis of Resectable Pancreatic Cancer	Gut and Liver	Records excluded after screening
565	Lee, J. H., L. S. Cassani, P. Bhosale and W. A. Ross	2016	The endoscopist's role in the diagnosis and management of pancreatic cancer	Expert Review of Gastroenterology and Hepatology	Records excluded after screening
566	Lee, J. H., Y. H. Kim, K. H. Kim, J. Y. Cho, S. M. Woo, B. C. Yoo and S. C. Kim	2018	Profiling of Serum Metabolites Using MALDI-TOF and Triple-TOF Mass Spectrometry to Develop a Screen for Ovarian Cancer	Cancer Research and Treatment	Records excluded after screening
567	Lee, J. H., S. E. Yu, K. H. Kim, M. H. Yu, I. H. Jeong, J. Y. Cho, S. J. Park, W. J. Lee, S. S. Han, T. H. Kim, E. K. Hong, S. M. Woo and B. C. Yoo	2018	Individualized metabolic profiling stratifies pancreatic and biliary tract cancer: a useful tool for innovative screening programs and predictive strategies in healthcare	EPMA Journal	Incomplete diagnostic performance data provided
568	Lee, M. J., W. H. Han, J. Y. Chun, S. Y. Kim and J. H. Kim	2021	Presepsin in the rapid response system for cancer patients: A retrospective analysis	Journal of Clinical Medicine	Records excluded after screening

569	Lee, M. J., K. Na, S. K. Jeong, J. S. Lim, S. A. Kim, M. J. Lee, S. Y. Song, H. Kim, W. S. Hancock and Y. K. Paik	2014	Identification of Human Complement Factor B as a Novel Biomarker Candidate for Pancreatic Ductal Adenocarcinoma	Journal of Proteome Research	Not early-stage
570	Lee, M. J., K. Na, H. Shin, C. Y. Kim, J. Y. Cho, C. M. Kang, S. H. Kim, H. Kim, H. J. Choi, C. K. Lee, S. Bae, S. Son and Y. K. Paik	2021	Early Diagnostic Ability of Human Complement Factor B in Pancreatic Cancer Is Partly Linked to Its Potential Tumor-Promoting Role	J Proteome Res	Studies included in review
571	Lee, S. and J. Kim	2021	The development of a highly efficient diagnostic marker combined with PSA of prostate cancer (PCa)	Cancer Research	Records excluded after screening
572	Lee, S. H. and J. Park	2022	Comprehensive Multi-Omics Profiling Reveals Clinically Relevant Molecular Subtypes with Potential Therapeutic Opportunities for Resectable Pancreatic Cancer	HPB	Records excluded after screening
573	Lee, S. H. and J. Park	2022	Unsupervised clustering of multi-omics molecular layers reveals consensus molecular subtypes showing potential therapeutic opportunities for pancreatic cancer	Cancer Research	Records excluded after screening
574	Lee, S. H., J. Park, S. Lee and J. S. Lee	2022	Comprehensive multi-omics profiling uncover potential precision strategies for resectable pancreatic cancer	International Journal of Surgery	Records excluded after screening
575	Lee, S. H., J. Park, S. Lee and J. S. Lee	2023	Cancer-specific molecular subtypes of pancreatic cancer reveal clinically relevant molecular dependency	Cancer Research	Records excluded after screening
576	Lee, Y. S., H. Kim, H. W. Kim, J. C. Lee, K. H. Paik, J. Kang, J. Kim, Y. S. Yoon, H. S. Han, I. Sohn, J. Cho and J. H. Hwang	2015	High Expression of MicroRNA-196a Indicates Poor Prognosis in Resected Pancreatic Neuroendocrine Tumor	Medicine (Baltimore)	Records excluded after screening
577	Lei, X. F., S. Z. Jia, J. Ye, Y. L. Qiao, G. M. Zhao, X. H. Li and H. Chang	2017	Application values of detection of serum CA199, CA242 AND CA50 in the diagnosis of pancreatic cancer	Journal of Biological Regulators and Homeostatic Agents	Records excluded after screening
578	Lenaerts, L., S. Tuveri, T. Jatsenko, F. Amant and J. R. Vermeesch	2020	Detection of incipient tumours by screening of circulating plasma DNA: hype or hope?	Acta Clinica Belgica: International Journal of Clinical and Laboratory Medicine	Records excluded after screening
579	Lenggenhager, D., S. Bengs, R. Fritsch, S. Hussung, P. Busenhart, K. Endhardt, A. Töpfer, F. O. The, S. Bütikofer, C. Gubler, M. Scharl and B. Morell	2021	β6-Integrin Serves as a Potential Serum Marker for Diagnosis and Prognosis of Pancreatic Adenocarcinoma	Clinical and Translational Gastroenterology	Non-diagnostic studies

580	Lennon, A. M., A. H. Buchanan, I. Kinde, A. Warren, A. Honushefsky, A. T. Cohain, D. H. Ledbetter, F. Sanfilippo, K. Sheridan, D. Rosica, C. S. Adonizio, H. J. Hwang, K. Lahouel, J. D. Cohen, C. Douville, A. A. Pate, L. N. Hagmann, D. D. Rolston, N. Malani, S. Zhou, C. Bettegowda, D. L. Diehl, B. Urban, C. D. Still, L. Kann, J. I. Woods, Z. M. Salvati, J. Vadakara, R. Leeming, P. Bhattacharya, C. Walter, A. Parker, C. Lengauer, A. Klein, C. Tomasetti, E. K. Fishman, R. H. Hruban, K. W. Kinzler, B. Vogelstein and N. Papadopoulos	2020	Feasibility of blood testing combined with PET-CT to screen for cancer and guide intervention	Science	Records excluded after screening
581	Lennon, A. M. and M. I. Canto	2017	Pancreatic Cysts - Part 2: Should We Be Less Cyst Centric?	Pancreas	Records excluded after screening
582	Levink, I., D. Klatte, R. Hanna-Sawires, I. Ibrahim, Y. van der Burgt, K. Overbeek, B. Koopmann, D. Cahen, G. Fuhler, M. Wuhrer, B. Bonsing, R. Tollenaar, F. Vleggaar, H. Vasen, M. van Leerdam, M. Bruno and W. Mesker	2021	Detection of serum N-glycans based on changes over time for earlier detection of pancreatic cancer in high-risk individuals	Pancreatology	Records excluded after screening

583	Levink, I. J. M., S. C. Jaarsma, B. D. M. Koopmann, P. A. van Riet, K. A. Overbeek, J. Meziani, M. L. J. A. Sprij, R. Casadei, C. Ingaldi, M. Polkowski, M. M. L. Engels, L. A. van der Waaij, S. Carrara, E. Pando, M. Vornhülz, P. Honkoop, E. J. Schoon, J. Laukkarinen, J. F. Bergmann, G. Rossi, F. G. I. van Vilsteren, A. M. van Berkel, T. Tabone, M. P. Schwartz, A. C. I. T. L. Tan, J. E. van Hooft, R. Quispel, E. van Soest, L. Czacko, M. J. Bruno and D. L. Cahen	2023	The additive value of CA19.9 monitoring in a pancreatic cyst surveillance program	United European Gastroenterology Journal	Records excluded after screening
584	Levink, I. J. M., D. C. F. Klatte, R. G. Hanna-Sawires, G. C. M. Vreeker, I. S. Ibrahim, Y. E. M. van der Burgt, K. A. Overbeek, B. D. M. Koopmann, D. L. Cahen, G. M. Fuhler, M. Wuhrer, B. A. Bonsing, R. Tollenaar, F. P. Vleggaar, H. F. A. Vasen, M. E. van Leerdam, M. J. Bruno and W. E. Mesker	2022	Longitudinal changes of serum protein N-Glycan levels for earlier detection of pancreatic cancer in high-risk individuals	Pancreatology	Not early-stage
585	Levink, I. J. M., M. I. Srebniak, W. G. De Valk, M. M. van Veghel-Plandsoen, A. Wagner, D. L. Cahen, G. M. Fuhler and M. J. Bruno	2023	An 8q24 Gain in Pancreatic Juice Is a Candidate Biomarker for the Detection of Pancreatic Cancer	International Journal of Molecular Sciences	Records excluded after screening
586	Levy, A., T. Popovici and P. N. Bories	2017	Tumor markers in pancreatic cystic fluids for diagnosis of malignant cysts	International Journal of Biological Markers	Non-diagnostic studies
587	Lewandowski, S., L. Diao, A. Quigley, M. Crochiere and J. Pinkas	2024	EDB+FN is an attractive therapeutic target in oncology: Insights from protein expression analysis of solid tumors	Cancer Research	Records excluded after screening
588	Lewis, A. R., J. W. Valle and M. G. McNamara	2016	Pancreatic cancer: Are "liquid biopsies" ready for prime-time?	World Journal of Gastroenterology	Records excluded after screening
589	Lewis, J. M., A. D. Vyas, Y. Qiu, K. S. Messer, R. White and M. J. Heller	2018	Integrated Analysis of Exosomal Protein Biomarkers on Alternating Current Electrokinetic Chips Enables Rapid Detection of Pancreatic Cancer in Patient Blood	ACS nano	Small sample size

590	Lewis, J. S.	2015	New methods of PET imaging pancreas cancer	Cancer Research	Records excluded after screening
591	Lewis, J. S.	2019	Retooling a blood-based biomarker into a theranostic agent	Cancer Research	Records excluded after screening
592	Li, B., C. Liu, W. Pan and L. Zheng	2019	DNA assembly assisting magnetic fluorescence nanosensor based on aggregation-induced emission probe/graphene oxide for cancerous exosome analysis	Journal of Extracellular Vesicles	Records excluded after screening
593	Li, D. D., Z. Q. Fu, Q. Lin, Y. Zhou, Q. B. Zhou, Z. H. Li, L. P. Tan, R. F. Chen and Y. M. Liu	2015	Linc00675 is a novel marker of short survival and recurrence in patients with pancreatic ductal adenocarcinoma	World J Gastroenterol	Records excluded after screening
594	Li, F. X., H. Y. Zhao, T. F. Lin, Y. W. Jiang, D. Liu, C. Wei, Z. Y. Zhao, Z. Y. Yang, F. Sha, Z. R. Yang and J. L. Tang	2023	Regular Glucosamine Use May Have Different Roles in the Risk of Site-Specific Cancers: Findings from a Large Prospective Cohort	Cancer Epidemiology Biomarkers and Prevention	Records excluded after screening
595	Li, G. S., T. Huang and H. F. Zhou	2023	Gene S-phase kinase associated protein 2 is a novel prognostic marker in human neoplasms	BMC Medical Genomics	Records excluded after screening
596	Li, H., A. R. Warden, W. Su, J. He, X. Zhi, K. Wang, L. Zhu, G. Shen and X. Ding	2021	Highly sensitive and portable mRNA detection platform for early cancer detection	Journal of Nanobiotechnology	Records excluded after screening
597	Li, J., Y. Li, S. Chen, W. Duan, X. Kong, Y. Wang, L. Zhou, P. Li, C. Zhang, L. Du and C. Wang	2022	Highly Sensitive Exosome Detection for Early Diagnosis of Pancreatic Cancer Using Immunoassay Based on Hierarchical Surface-Enhanced Raman Scattering Substrate	Small Methods	Records excluded after screening
598	Li, J., Z. Ye, X. Hu, S. Hou and Q. Hang	2022	Prognostic, Diagnostic, and Clinicopathological Significance of Circular RNAs in Pancreatic Cancer: A Systematic Review and Meta-Analysis	Cancers	Records excluded after screening
599	Li, L., M. Liu, J. B. Lin, X. B. Hong, W. X. Chen, H. Guo, L. Y. Xu, Y. W. Xu, E. M. Li and Y. H. Peng	2017	Diagnostic Value of Autoantibodies against Ezrin in Esophageal Squamous Cell Carcinoma	Disease Markers	Records excluded after screening
600	Li, L. X., B. Zhang and R. Z. Gong	2020	Insights into the role of tumor abnormal protein in early diagnosis of cancer: A prospective cohort study	Medicine (Baltimore)	Records excluded after screening
601	Li, Q., S. H. Maier, P. Li, J. Peterhansl, C. Belka, J. Mayerle and U. M. Mahajan	2020	Aptamers: a novel targeted theranostic platform for pancreatic ductal adenocarcinoma	Radiation Oncology	Records excluded after screening
602	Li, Q., H. Wang, G. Zogopoulos, Q. Shao, J. L. Liu and Z. H. Gao	2016	Overexpression of Reg proteins in human pancreatic ductal adenocarcinoma and acinar-to-ductal metaplasia	Cancer Research	Records excluded after screening
603	Li, Q., G. Zogopoulos, X. Gui, Z. H. Gao and J. L. Liu	2014	Specific overexpression of reg proteins 1a and 1b in human pancreatic ductal adenocarcinoma	Endocrine Reviews	Records excluded after screening
604	Li, S., L. Wang, Q. Zhao, Z. Wang, S. Lu, Y. Kang, G. Jin and J. Tian	2020	Genome-Wide Analysis of Cell-Free DNA Methylation Profiling for the Early Diagnosis of Pancreatic Cancer	Frontiers in Genetics	Records excluded after screening

605	Li, S. L., J. Yang, X. F. Lei, J. N. Zhang, H. L. Yang, K. Li and C. Q. Xu	2016	Peptide-Conjugated Quantum Dots Act as the Target Marker for Human Pancreatic Carcinoma Cells	Cell Physiol Biochem	Records excluded after screening
606	Li, T., J. Xia, H. Yun, G. Sun, Y. Shen, P. Wang, J. Shi, K. Wang, H. Yang and H. Ye	2023	A novel autoantibody signatures for enhanced clinical diagnosis of pancreatic ductal adenocarcinoma	Cancer Cell International	Studies included in review
607	Li, T. D., R. Zhang, H. Chen, Z. P. Huang, X. Ye, H. Wang, A. M. Deng and J. L. Kong	2018	An ultrasensitive polydopamine bi-functionalized SERS immunoassay for exosome-based diagnosis and classification of pancreatic cancer	Chemical Science	Records excluded after screening
608	Li, W., M. Gonzalez-Gonzalez, L. Sanz-Criado, N. Garcia-Carbonero, A. Celdran, P. Villarejo-Campos, P. Minguez, R. Pazo-Cid, C. Garcia-Jimenez, A. Orta-Ruiz, J. Garcia-Foncillas and J. Martinez-Useros	2022	A Novel PiRNA Enhances CA19-9 Sensitivity for Pancreatic Cancer Identification by Liquid Biopsy	Journal of Clinical Medicine	No sample information provided
609	Li, X., P. Gao, Y. Wang and X. Wang	2018	Blood-derived microRNAs for pancreatic cancer diagnosis: A narrative review and meta-analysis	Frontiers in Physiology	Records excluded after screening
610	Li, X., X. Guo, H. Li, H. Lin and Y. Sun	2014	Serum carbohydrate antigen 242 expression exerts crucial function in the diagnosis of pancreatic cancer	Tumor Biology	Records excluded after screening
611	Li, X., Z. Li, H. Zhu and X. Yu	2020	Autophagy Regulatory Genes MET and RIPK2 Play a Prognostic Role in Pancreatic Ductal Adenocarcinoma: A Bioinformatic Analysis Based on GEO and TCGA	Biomed Res Int	Records excluded after screening
612	Li, X. Y., L. X. Su, W. X. Chen, H. Liu, L. Y. Zhang, Y. C. Shen, J. X. You, J. B. Wang, L. Zhang, D. Wang, M. Z. Wen, Z. Wang, Y. H. Shao, D. H. Chen and X. T. Yang	2022	Clinical Implications of Necroptosis Genes Expression for Cancer Immunity and Prognosis: A Pan-Cancer Analysis	Frontiers in Immunology	Records excluded after screening
613	Li, Y., S. Sui and A. Goel	2024	Extracellular vesicles associated microRNAs: Their biology and clinical significance as biomarkers in gastrointestinal cancers	Seminars in Cancer Biology	Records excluded after screening
614	Li, Y., L. Wu, W. Tao, D. Wu, F. Ma and N. Li	2020	Expression Atlas of FGF and FGFR Genes in Pancancer Uncovered Predictive Biomarkers for Clinical Trials of Selective FGFR Inhibitors	BioMed Research International	Records excluded after screening
615	Li, Z., C. Hsu, L. Hwang and Y. Lin	2015	Towards early pancreatic cancers detection by CA19-9 conjugated magnetic nanoparticles and active feedback magnetic resonance molecular imaging	Molecular Imaging and Biology	Records excluded after screening
616	Li, Z. Z., L. F. Shen, Y. Y. Li, P. Chen and L. Z. Chen	2016	Clinical utility of microRNA-378 as early diagnostic biomarker of human cancers: a meta-analysis of diagnostic test	Oncotarget	Records excluded after screening

617	Liang, B., L. Zhong, Q. He, S. Wang, Z. Pan, T. Wang and Y. Zhao	2015	Serum dickkopf-1 as a biomarker in screening gastrointestinal cancers: A systematic review and meta-analysis	OncoTargets and Therapy	Records excluded after screening
618	Liao, W. C.	2023	Early Detection of Pancreatic Cancer: Opportunities Provided by Cancer-induced Paraneoplastic Phenomena and Artificial Intelligence	Journal of Cancer Research and Practice	Records excluded after screening
619	Liberati, D., I. Marzinotto, C. Brigatti, E. Dugnani, V. Pasquale, M. Reni, G. Balzano, M. Falconi, L. Piemonti and V. Lampasona	2018	No evidence of pancreatic ductal adenocarcinoma specific autoantibodies to Ezrin in a liquid phase LIPS immunoassay	Cancer Biomark	Not early-stage
620	Lih, T. M., L. Cao, P. Minoo, G. S. Omenn, R. H. Hruban, D. W. Chan, O. F. Bathe and H. Zhang	2024	Detection of Pancreatic Ductal Adenocarcinoma-Associated Proteins in Serum	Mol Cell Proteomics	Non-diagnostic studies
621	Lin, M., M. Alnaggar, S. Liang, J. Chen, K. Xu, S. Dong, D. Du and L. Niu	2018	Circulating Tumor DNA as a Sensitive Marker in Patients Undergoing Irreversible Electroporation for Pancreatic Cancer	Cellular Physiology and Biochemistry	No specific biomarkers provided
622	Lin, M. S., W. C. Chen, J. X. Huang, H. J. Gao and H. H. Sheng	2014	Aberrant expression of microRNAs in serum may identify individuals with pancreatic cancer	International Journal of Clinical and Experimental Medicine	Records excluded after screening
623	Linh, V. T. N., M. Y. Lee, J. Mun, Y. Kim, H. Kim, I. W. Han, S. G. Park, S. Choi, D. H. Kim, J. Rho and H. S. Jung	2023	3D plasmonic coral nanoarchitecture paper for label-free human urine sensing and deep learning-assisted cancer screening	Biosensors and Bioelectronics	Records excluded after screening
624	Litman-Zawadzka, A., M. Łukaszewicz-Zając, M. Gryko, A. Kulczyńska-Przybik, B. Kędra and B. Mroczko	2020	Specific Receptors for the Chemokines CXCR2 and CXCR4 in Pancreatic Cancer	Int J Mol Sci	Records excluded after screening
625	Liu, C., T. Qin, Y. Huang, Y. Li, G. Chen and C. Sun	2020	Drug screening model meets cancer organoid technology	Translational Oncology	Records excluded after screening
626	Liu, F., F. Du and X. Chen	2014	Multiple tumor marker protein chip detection system in diagnosis of pancreatic cancer	World Journal of Surgical Oncology	Records excluded after screening
627	Liu, F., B. Liu, J. Qian, G. Wu, J. Li and Z. Ma	2017	miR-153 enhances the therapeutic effect of gemcitabine by targeting Snail in pancreatic cancer	Acta biochimica et biophysica Sinica	Records excluded after screening
628	Liu, F., H. Mao, S. Chai and H. Mao	2021	Meta-analysis of the diagnostic value of exosomal miR-21 as a biomarker for the prediction of cancer	Journal of Clinical Laboratory Analysis	Records excluded after screening
629	Liu, H., B. Sun, S. Wang, C. Liu, Y. Lu, D. Li and X. Liu	2017	Circulating tumor cells as a biomarker in pancreatic ductal adenocarcinoma	Cellular Physiology and Biochemistry	No sample information provided
630	Liu, H. and X. Wang	2018	Advances in research of peripheral blood mirnas in early diagnosis of tumors	Chinese Journal of Cancer Biotherapy	Records excluded after screening

631	Liu, H. J., Y. Y. Guo and D. J. Li	2017	Predicting novel salivary biomarkers for the detection of pancreatic cancer using biological feature-based classification	Pathology Research and Practice	Records excluded after screening
632	Liu, J. X., A. Li, L. Y. Zhou, X. F. Liu, Z. H. Wei, X. Z. Wang and H. Q. Ying	2018	Significance of combined preoperative serum Alb and dNLR for diagnosis of pancreatic cancer	Future Oncology	Not early-stage
633	Liu, L., H. Qin and M. Ye	2021	Recent advances in glycopeptide enrichment and mass spectrometry data interpretation approaches for glycoproteomics analyses	Se pu = Chinese journal of chromatography	Records excluded after screening
634	Liu, W., W. Liu, K. Lin, Y. Liu, H. Hu and L. Yang	2022	A clinical study of serum human epididymis protein 4 (HE4) in the diagnosis of pancreatic cancer	Indian Journal of Cancer	Not early-stage
635	Liu, X., S. Guo, C. Ma, Y. Li, X. Liu, Z. Zhang, L. Yu, M. Dai, S. Shen, H. M. Wu, Z. Su, Q. He, R. Liu, J. Gang and Z. Liang	2021	Circulating tumor DNAmethylation as markers for earlydetection of pancreatic ductaladenocarcinoma (PDAC)	Journal of Clinical Oncology	Records excluded after screening
636	Liu, X., Q. He, Z. Liang, H. Wu, Y. Li, Z. Zhang, L. Yu, M. Dai, S. Guo, G. Jin, S. Shen, Z. Su, C. Ma, Z. Xie and R. Liu	2020	118MO Circulating tumour DNA methylation are markers for early detection of pancreatic ductal adenocarcinoma (PDAC)	Annals of Oncology	Records excluded after screening
637	Liu, X., Q. He, Z. Su, S. Guo, Z. Liang and G. Jin	2020	Early detection of pancreatic ductal adenocarcinoma (PDAC) using methylation signatures in circulating tumour DNA	Annals of Oncology	Records excluded after screening
638	Liu, X., W. Zheng, W. Wang, H. Shen, L. Liu, W. Lou, X. Wang and P. Yang	2017	A new panel of pancreatic cancer biomarkers discovered using a mass spectrometry-based pipeline	British Journal of Cancer	Records excluded after screening
639	Liu, X. D., H. Wu, Y. Li, X. Liu, Z. Zhang, L. Yu, Z. Qin, Z. Su, R. Liu, Q. He, M. Dai and Z. Liang	2019	Early detection of pancreatic ductal adenocarcinoma using methylation signatures in circulating tumour DNA	Annals of Oncology	Records excluded after screening
640	Liu, X. H., W. M. Zheng, W. S. Wang, H. L. Shen, L. X. Liu, W. H. Lou, X. L. Wang and P. Y. Yang	2017	A new panel of pancreatic cancer biomarkers discovered using a mass spectrometry-based pipeline	British Journal of Cancer	Records excluded after screening
641	Liu, Y., W. Feng, W. Liu, X. Kong, L. Li, J. He, D. Wang, M. Zhang, G. Zhou, W. Xu, W. Chen, A. Gong and M. Xu	2019	Circulating lncRNA ABHD11-AS1 serves as a biomarker for early pancreatic cancer diagnosis	Journal of Cancer	Not early-stage
642	Liu, Y., H. Meng, S. Xu and X. Qi	2020	Galectins for diagnosis and prognostic assessment of human diseases: An overview of meta-analyses	Medical Science Monitor	Records excluded after screening
643	Liyanage, T., A. N. Masterson, H. H. Oyem, H. Kaimakliotis, H. Nguyen and R. Sardar	2019	Plasmoelectronic-Based Ultrasensitive Assay of Tumor Suppressor microRNAs Directly in Patient Plasma: Design of Highly Specific Early Cancer Diagnostic Technology	Anal Chem	Records excluded after screening

644	Llop, E., P. E. Guerrero, A. Duran, S. Barrabés, A. Massaguer, M. J. Ferri, M. Albiol-Quer, R. De Llorens and R. Peracaula	2018	Glycoprotein biomarkers for the detection of pancreatic ductal adenocarcinoma	World Journal of Gastroenterology	Records excluded after screening
645	Loc, W. S., J. P. Smith, G. Matters, M. Kester and J. H. Adair	2014	Novel strategies for managing pancreatic cancer	World J Gastroenterol	Records excluded after screening
646	Loft, M., B. Lee, J. Tie and P. Gibbs	2019	Clinical applications of circulating tumour DNA in pancreatic adenocarcinoma	Journal of Personalized Medicine	Records excluded after screening
647	Long, N. P., S. J. Yoon, N. H. Anh, T. D. Nghi, D. K. Lim, Y. J. Hong, S. S. Hong and S. W. Kwon	2018	A systematic review on metabolomics-based diagnostic biomarker discovery and validation in pancreatic cancer	Metabolomics	Records excluded after screening
648	Long, X. R., Y. J. Zhang, M. Y. Zhang, K. Chen, X. F. S. Zheng and H. Y. Wang	2017	Identification of an 88-microRNA signature in whole blood for diagnosis of hepatocellular carcinoma and other chronic liver diseases	Aging-Us	Records excluded after screening
649	Loosen, S. H., M. Luedde, G. Lurje, M. Spehlmann, P. Paffenholz, T. F. Ulmer, F. Tacke, M. Vucur, C. Trautwein, U. P. Neumann, T. Luedde and C. Roderburg	2019	Serum Levels of Kisspeptin Are Elevated in Patients with Pancreatic Cancer	Disease Markers	Not early-stage
650	Loosen, S. H., U. P. Neumann, C. Trautwein, C. Roderburg and T. Luedde	2017	Current and future biomarkers for pancreatic adenocarcinoma	Tumor Biology	Records excluded after screening
651	López-López, Á., Á. López-González, T. C. Barker-Tejeda and C. Barbas	2018	A review of validated biomarkers obtained through metabolomics	Expert Review of Molecular Diagnostics	Records excluded after screening
652	Lowy, A. M.	2019	Exosomes from Pancreatic Juice: A Step Closer to the Holy Grail?	Annals of Surgical Oncology	Records excluded after screening
653	Lozanovski, V. J., P. Houben, U. Hinz, T. Hackert, I. Herr and P. Schemmer	2014	Pilot study evaluating broccoli sprouts in advanced pancreatic cancer (POUDER trial) - study protocol for a randomized controlled trial	Trials	Records excluded after screening
654	Lu, C., C. F. Xu, X. Y. Wan, H. T. Zhu, C. H. Yu and Y. M. Li	2015	Screening for pancreatic cancer in familial high-risk individuals: A systematic review	World J Gastroenterol	Records excluded after screening
655	Lu, L. and H. A. Risch	2016	Exosomes: Potential for early detection in pancreatic cancer	Future Oncology	Records excluded after screening
656	Lu, Y. K., X. Chu, S. Wang, Y. Sun, J. Zhang, J. Dong and Y. X. Yan	2021	Identification of Circulating hsa_circ_0063425 and hsa_circ_0056891 as Novel Biomarkers for Detection of Type 2 Diabetes	Journal of Clinical Endocrinology & Metabolism	Records excluded after screening

657	Luan, Y., G. Zhong, S. Li, W. Wu, X. Liu, D. Zhu, Y. Feng, Y. Zhang, C. Duan and M. Mao	2023	A panel of seven protein tumour markers for effective and affordable multi-cancer early detection by artificial intelligence: a large-scale and multicentre case–control study	eClinicalMedicine	Records excluded after screening
658	Lucien, F., V. Lac, D. D. Billadeau, A. Borgida, S. Gallinger and H. S. Leong	2019	Glypican-1 and glycoprotein 2 bearing extracellular vesicles do not discern pancreatic cancer from benign pancreatic diseases	Oncotarget	Records excluded after screening
659	Lucien, F., V. Lac and H. S. Leong	2017	Presence of glypican-1 on extracellular vesicles fails to discern pancreatic cancer from benign pancreatic diseases	Journal of Extracellular Vesicles	Records excluded after screening
660	Lumibao, J., S. Okhovat, K. Peck and D. Engle	2022	Utility of patient-derived organoids for precision pharmacotyping, phenotypic screens, and modeling of pancreatic disease	Cancer Research	Records excluded after screening
661	Luo, G., M. Guo, K. Jin, Z. Liu, C. Liu, H. Cheng, Y. Lu, J. Long, L. Liu, J. Xu, Q. Ni and X. Yu	2016	Optimize CA19-9 in detecting pancreatic cancer by Lewis and Secretor genotyping	Pancreatology	Not early-stage
662	Luo, G., K. Jin, S. Deng, H. Cheng, Z. Fan, Y. Gong, Y. Qian, Q. Huang, Q. Ni, C. Liu and X. Yu	2021	Roles of CA19-9 in pancreatic cancer: Biomarker, predictor and promoter	Biochimica et Biophysica Acta - Reviews on Cancer	Records excluded after screening
663	Luo, X., J. Liu, H. Wang and H. Lu	2020	Metabolomics identified new biomarkers for the precise diagnosis of pancreatic cancer and associated tissue metastasis	Pharmacological Research	No sample information provided
664	Luo, Y., H. Zhu, T. Tan and J. He	2018	Current standards and recent advances in biomarkers of major endocrine tumors	Frontiers in Pharmacology	Records excluded after screening
665	Luu, T. T.	2021	Review of Immunohistochemistry Biomarkers in Pancreatic Cancer Diagnosis	Frontiers in Oncology	Records excluded after screening
666	Lv, Y., Z. Zhou, Y. Shen, Q. Zhou, J. Ji, S. Liu and Y. Zhang	2018	Coupled Fluorometer-Potentiostat System and Metal-Free Monochromatic Luminophores for High-Resolution Wavelength-Resolved Electrochemiluminescent Multiplex Bioassay	ACS Sens	Records excluded after screening
667	Lyu, S. C., J. Wang, M. Huang, H. X. Wang, L. Zhou, Q. He and R. Lang	2021	Ca19-9 level to serum γ-glutamyltransferase as a potential prognostic biomarker in patients with pancreatic head carcinoma	Cancer Management and Research	No sample information provided
668	Ma, C., F. Jiang, Y. Ma, J. Wang, H. Li and J. Zhang	2019	Isolation and Detection Technologies of Extracellular Vesicles and Application on Cancer Diagnostic	Dose-Response	Records excluded after screening
669	Ma, J., X. Huang, J. Zhao, J. Lu, W. Lu, Y. Bao, J. Zhou and J. Han	2021	CGM for insulinoma screening: A prospective and observational case-control study	Endocrine-Related Cancer	Records excluded after screening
670	Madhavan, B., S. Yue, U. Galli, S. Rana, W. Gross, M. Müller, N. A. Giese, H. Kalthoff, T. Becker, M. W. Büchler and M. Zöller	2015	Combined evaluation of a panel of protein and miRNA serum-exosome biomarkers for pancreatic cancer diagnosis increases sensitivity and specificity	International Journal of Cancer	Not early-stage

671	Mahon, N., E. Bichenkova, O. Tsigkou and A. Saiani	2023	DESIGNING A PEPTIDE HYDROGEL FOR EARLY DETECTION OF CANCER	Tissue Engineering - Part A	Records excluded after screening
672	Mai, D., Y. Zheng, H. Guo, P. Ding, R. Bai, M. Li, Y. Ye, J. Zhang, X. Huang, D. Liu, Q. Sui, L. Pan, J. Su, J. Deng, G. Wu, R. Li, S. Deng, Y. Bai, Y. Ligu, W. Tan, C. Wu, T. Wu, J. Zheng and D. Lin	2020	Serum piRNA-54265 is a new biomarker for early detection and clinical surveillance of Human Colorectal Cancer	Theranostics	Records excluded after screening
673	Maitra, A., A. Sharma, R. E. Brand, S. K. Van Den Eeden, W. E. Fisher, P. A. Hart, S. J. Hughes, K. J. Mather, S. J. Pandol, W. G. Park, Z. Feng, J. Serrano, J. A. S. Rinaudo, S. Srivastava and S. T. Chari	2018	A prospective study to establish a new-onset diabetes cohort: From the consortium for the study of chronic pancreatitis, diabetes, and pancreatic cancer	Pancreas	Non-diagnostic studies
674	Majumder, S., S. T. Chari and D. A. Ahlquist	2015	Molecular detection of pancreatic neoplasia: Current status and future promise	World Journal of Gastroenterology	Records excluded after screening
675	Majumder, S., A. Chatterjee, W. R. Taylor, P. H. Foote, S. A. A. Mohiuddin, C. K. Berger, R. Graham, K. Doering, K. N. Burger, D. W. Mahoney, M. Giakoumopoulos, A. M. Solsrud, H. T. Allawi, G. M. Petersen and J. B. Kisiel	2021	FEASIBILITY OF DETECTING CANCER IN INTRADUCTAL PAPILLARY MUCINOUS NEOPLASMS USING PLASMA METHYLATED DNA MARKERS	Gastroenterology	Records excluded after screening
676	Majumder, S., M. Raimondo, W. R. Taylor, T. C. Yab, C. K. Berger, B. A. Dukek, X. Cao, P. H. Foote, C. W. Wu, M. E. Devens, D. W. Mahoney, T. C. Smyrk, R. Pannala, S. T. Chari, S. S. Vege, M. D. Topazian, B. T. Petersen, M. J. Levy, E. Rajan, F. C. Gleeson, B. Abu Dayyeh, C. C. Nguyen, D. O. Faigel, T. A. Woodward, M. B. Wallace, G. Petersen, H. T. Allawi, G. P. Lidgard, J. B. Kisiel and D. A. Ahlquist	2020	Methylated DNA in Pancreatic Juice Distinguishes Patients With Pancreatic Cancer From Controls	Clinical Gastroenterology and Hepatology	Studies included in review

677	Majumder, S., W. R. Taylor, P. H. Foote, C. K. Berger, C. W. Wu, T. C. Yab, D. W. Mahoney, W. R. Bamlet, K. N. Burger, N. Postier, K. Doering, G. P. Lidgard, H. T. Allawi, J. B. Kisiel, G. M. Petersen, S. T. Chari and D. A. Ahlquist	2019	PANCREATIC CANCER DETECTION BY PLASMA ASSAY OF NOVEL METHYLATED DNA MARKERS: A CASE-CONTROL STUDY	Gastroenterology	Incomplete diagnostic performance data provided
678	Makler, A. and W. Asghar	2023	Exosomal miRNA Biomarker Panel for Pancreatic Ductal Adenocarcinoma Detection in Patient Plasma: A Pilot Study	International Journal of Molecular Sciences	Records excluded after screening
679	Malhotra, A., B. Rachet, A. Bonaventure, S. P. Pereira and L. M. Woods	2021	Can we screen for pancreatic cancer? Identifying a sub-population of patients at high risk of subsequent diagnosis using machine learning techniques applied to primary care data	Plos One	Non-diagnostic studies
680	Malhotra, P., I. Casari and M. Falasca	2023	Can the molecules carried by extracellular vesicles help to diagnose pancreatic cancer early?	Biochimica Et Biophysica Acta-General Subjects	Records excluded after screening
681	Malya, F. U., E. Biberci Keskin, H. I. Köker, A. Tüzün Ince and H. Senturk	2018	The importance of biomarkers in early diagnosis of pancreatic cancer: Glypican 1	United European Gastroenterology Journal	Records excluded after screening
682	Mamie Lih, T., L. Cao, P. Minoo, G. S. Omenn, R. H. Hruban, D. W. Chan, O. F. Bathe and H. Zhang	2024	Detection of Pancreatic Ductal Adenocarcinoma-Associated Proteins in Serum	Molecular and Cellular Proteomics	Records excluded after screening
683	Manchado, E., S. Weissmueller, J. P. Morris, C. C. Chen, R. Wullenkord, A. Lujambio, E. De Stanchina, J. T. Poirier, J. F. Gainor, R. B. Corcoran, J. A. Engelman, C. M. Rudin, N. Rosen and S. W. Lowe	2016	A combinatorial strategy for treating KRAS-mutant lung cancer	Nature	Records excluded after screening
684	Mann, M. B., N. A. Jenkins, N. G. Copeland and K. M. Mann	2014	Sleeping Beauty mutagenesis: Exploiting forward genetic screens for cancer gene discovery	Current Opinion in Genetics and Development	Records excluded after screening
685	Mao, M., Y. Luan, G. Zhong, S. Li, W. Wu, X. Liu, D. Zhu, S. Tu, Y. Feng, Y. Zhang and C. Duan	2023	A panel of seven protein tumour markers for effective and affordable multi-cancer early detection by artificial intelligence	JCO Global Oncology	Records excluded after screening
686	Mao, M., B. Wei, Q. Xu, Y. Shen, R. Brando, S. Li, W. Wu, P. Xing, Y. Chang and D. Zhu	2024	Large-scale validaton studies of a blood-based effective and affordable test for multicancer early detection	Cancer Research	Records excluded after screening

687	Maqsood, Q., A. Sumrin, Y. Saleem, A. Wajid and M. Mahnoor	2024	Exosomes in Cancer: Diagnostic and Therapeutic Applications	Clinical Medicine Insights: Oncology	Records excluded after screening
688	Marengo, E. and E. Robotti	2014	Biomarkers for pancreatic cancer: Recent achievements in proteomics and genomics through classical and multivariate statistical methods	World Journal of Gastroenterology	Records excluded after screening
689	Marin, A. M., M. Batista, A. L. K. de Azevedo, T. H. B. Gomig, R. S. C. Brant, R. Chammas, M. Uno, D. D. Araújo, D. L. Zanette and M. N. Aoki	2023	Screening of Exosome-Derived Proteins and Their Potential as Biomarkers in Diagnostic and Prognostic for Pancreatic Cancer	International Journal of Molecular Sciences	Small sample size
690	Marks, D. L., R. L. Olson, R. Urrutia, D. D. Billadeau, N. Roy, G. A. Calin, M. Fabbri, M. Koutsioumpa, D. Iliopoulos, T. Ordog, R. Huebert, O. Sarmento, A. O. Bamidele, W. Faubion, G. L. Lomberk, J. Siveke, N. Ahuja, J. Iovanna, R. A. Hlady, K. Robertson, J. Kisiel, C. L. Pin and M. E. Fernandez-Zapico	2018	Epigenetics of gastrointestinal diseases: notes from a workshop	Epigenetics	Records excluded after screening
691	Martin, K., P. Dunne, A. Connolly, C. Richardson, R. McConnell, J. Lamont and S. Fitzgerald	2019	Biomarker glycosylation evaluation in pancreatic cancer utilising biochip array technology	Clinica Chimica Acta	Records excluded after screening
692	Martínez, A., A. Martín-Carnicero, M. T. Tena and M. P. Martínez-Moral	2023	Highly sensitive serum volatolomic biomarkers for pancreatic cancer diagnosis and prognosis	Annals of Oncology	Non-diagnostic studies
693	Martinez-Bosch, N., L. E. Barranco, C. A. Orozco, M. Moreno, L. Visa, M. Iglesias, L. Oldfield, J. P. Neoptolemos, W. Greenhalf, J. Earl, A. Carrato, E. Costello and P. Navarro	2018	Increased plasma levels of galectin-1 in pancreatic cancer: Potential use as biomarker	Oncotarget	Records excluded after screening
694	Masterson, A. N., N. N. Chowdhury, Y. Fang, M. T. Yip-Schneider, S. Hati, P. Gupta, S. Cao, H. Wu, C. M. Schmidt, M. L. Fishel and R. Sardar	2023	Amplification-Free, High-Throughput Nanoplasmonic Quantification of Circulating MicroRNAs in Unprocessed Plasma Microsamples for Earlier Pancreatic Cancer Detection	ACS sensors	Studies included in review

695	Mateo, L., M. Duran-Frigola, A. Gris-Oliver, M. Palafox, M. Scaltriti, P. Razavi, S. Chandarlapaty, J. Arribas, M. Bellet, V. Serra and P. Aloy	2020	Personalized cancer therapy prioritization based on driver alteration co-occurrence patterns	Genome Medicine	Records excluded after screening
696	Matsunaga, T., T. Ohtsuka, K. Asano, T. Fujimoto, K. Date, H. Kimura, Y. Watanabe, K. Tamura, K. Ohuchida, S. Takahata, K. Mizumoto, S. Guha, M. Raimondo and M. Tanaka	2014	A minimally invasive screening test to detect pancreatic ductal adenocarcinoma using biomarkers in the duodenal gluid: An up-to-date report	Pancreas	Records excluded after screening
697	Matsunaga, T., T. Ohtsuka, K. Asano, H. Kimura, K. Ohuchida, H. Kitada, N. Ideno, Y. Mori, S. Tokunaga, Y. Oda, S. Guha, M. Raimondo, M. Nakamura and M. Tanaka	2017	S100P in Duodenal Fluid Is a Useful Diagnostic Marker for Pancreatic Ductal Adenocarcinoma	Pancreas	Studies included in review
698	Matsuzaki, J., K. Kato, Y. Saito, H. Daiko, H. Katai, Y. Kanemitsu, T. Okusaka, K. Shimada, H. Sakamoto, S. Niida and et al.	2018	Circulating microRNA classifiers to distinguish digestive cancers	Cancer science	Records excluded after screening
699	Mawaribuchi, S., O. Shimomura, T. Oda, K. Hiemori, K. Shimizu, K. Yamase, M. Date and H. Tateno	2023	rBC2LCN-reactive SERPINA3 is a glycobiomarker candidate for pancreatic ductal adenocarcinoma	Glycobiology	Studies included in review
700	Mayerle, J., H. Kalthoff, B. Kamlage, G. Adam, N. Christiansen, P. Schatz, T. Fahlbusch, B. Bethan, S. S. Pellengahr, S. Krusekopf, A. Chromik, F. Klein, M. Bahra, M. Distler, C. Pilarsky, R. Grützmann, W. Uhl and M. M. Lerch	2018	Novel metabolic targeted LC-MS/MS assay to differentiate pancreatic cancer from chronic pancreatitis in plasma	Pancreatology	Records excluded after screening

701	Mayerle, J., H. Kalthoff, B. Kamlage, N. Christiansen, P. Schatz, T. Fahlbusch, B. Bethan, S. S. Pellengahr, S. Krusekopf, A. Chromik, F. Klein, M. Bahra, M. Distler, C. Pilarsky, R. Grü tzmann, W. Uhl and M. Lerch	2018	Differentiation of pancreatic cancer from chronic pancreatitis with a novel plasma-based targeted LC-MS/MS assay	Oncology Research and Treatment	Records excluded after screening
702	Mayerle, J., H. Kalthoff, R. Reszka, B. Kamlage, E. Peter, B. Schniewind, S. Gonzá lez Maldonado, C. Pilarsky, C. D. Heidecke, P. Schatz, M. Distler, J. A. Scheiber, U. M. Mahajan, F. U. Weiss, R. Grützmann and M. M. Lerch	2018	Metabolic biomarker signature to differentiate pancreatic ductal adenocarcinoma from chronic pancreatitis	Gut	Studies included in review
703	Mazer, B. L., J. W. Lee, N. J. Roberts, L. C. Chu, A. M. Lennon, A. P. Klein, J. R. Eshleman, E. K. Fishman, M. I. Canto, M. G. Goggins and R. H. Hruban	2023	Screening for pancreatic cancer has the potential to save lives, but is it practical?	Expert Review of Gastroenterology and Hepatology	Records excluded after screening
704	Mazza, T., D. Gioffreda, A. Fontana, T. Biagini, M. Carella, O. Palumbo, E. Maiello, F. Bazzocchi, A. Andriulli and F. Tavano	2020	Clinical Significance of Circulating miR-1273g-3p and miR-122-5p in Pancreatic Cancer	Frontiers in Oncology	Records excluded after screening
705	McCarthy, A. J., D. M. Karamchandani and R. Chetty	2018	Neural and neurogenic tumours of the gastroenteropancreaticobiliary tract	J Clin Pathol	Records excluded after screening
706	McDonald, H., M. Gao, M. Barry-Hundeyin, T. Fan and J. Kim	2023	Exosomal Isolation of Immune Checkpoints From Pancreatic Ductal Adenocarcinoma Patient-Derived Organoids	Annals of Surgical Oncology	Records excluded after screening
707	Mead, E. A., N. Boulghassoul-Pietrzykowska, Y. Wang, O. Anees, N. S. Kinstlinger, M. Lee, S. Hamza, Y. Feng and A. Z. Pietrzykowski	2022	Non-Invasive microRNA Profiling in Saliva can Serve as a Biomarker of Alcohol Exposure and Its Effects in Humans	Frontiers in Genetics	Records excluded after screening
708	Medina, J. E., N. C. Dracopoli, P. B. Bach, A. Lau, R. B. Scharpf, G. A. Meijer, C. L. Andersen and V. E. Velculescu	2023	Cell-free DNA approaches for cancer early detection and interception	Journal for ImmunoTherapy of Cancer	Records excluded after screening

709	Mehta, K. Y., H. J. Wu, S. S. Menon, Y. Fallah, X. Zhong, N. Rizk, K. Unger, M. Mapstone, M. S. Fiandaca, H. J. Federoff and A. K. Cheema	2017	Metabolomic biomarkers of pancreatic cancer: a meta-analysis study	Oncotarget	Records excluded after screening
710	Meidhof, S., S. Brabletz, W. Lehmann, B. T. Preca, K. Mock, M. Ruh, J. Schüler, M. Berthold, A. Weber, U. Burk, M. Lübbert, M. Puhr, Z. Culig, U. Wellner, T. Keck, P. Bronsert, S. K üsters, U. T. Hopt, M. P. Stemmler and T. Brabletz	2015	ZEB1-associated drug resistance in cancer cells is reversed by the class I HDAC inhibitor mocetinostat	Embo Molecular Medicine	Records excluded after screening
711	Meleady, P., R. Abdul Rahman, M. Henry, M. Moriarty and M. Clynes	2020	Proteomic analysis of pancreatic ductal adenocarcinoma	Expert Review of Proteomics	Records excluded after screening
712	Melo, S. A., L. B. Luecke, C. Kahlert, A. F. Fernandez, S. T. Gammon, J. Kaye, V. S. LeBleu, E. A. Mittendorf, J. Weitz, N. Rahbari, C. Reissfelder, C. Pilarsky, M. F. Fraga, D. Piwnica-Worms and R. Kalluri	2015	Glypican-1 identifies cancer exosomes and detects early pancreatic cancer	Nature	Records excluded after screening
713	Melson, J., Y. Li, E. Cassinotti, A. Melnikov, L. Boni, J. Ai, M. Greenspan, S. Mobarhan, V. Levenson and Y. Deng	2014	Commonality and differences of methylation signatures in the plasma of patients with pancreatic cancer and colorectal cancer	International Journal of Cancer	Records excluded after screening
714	Mendonça, J. B., P. V. Fernandes, D. C. Fernandes, F. R. Rodrigues, M. C. Waghabi and T. M. Tilli	2024	Unlocking Overexpressed Membrane Proteins to Guide Breast Cancer Precision Medicine	Cancers	Records excluded after screening
715	Metzenmacher, M., R. Váraljai, B. Hegedüs, I. Cima, J. Forster, A. Schramm, B. Scheffler, P. A. Horn, C. A. Klein, T. Szarvas, H. Reis, N. Bielefeld, A. Roesch, C. Aigner, V. Kunzmann, M. Wiesweg, J. T. Siveke, M. Schuler and S. S. Lueong	2020	Plasma Next Generation Sequencing and Droplet Digital-qPCR-Based Quantification of Circulating Cell-Free RNA for Noninvasive Early Detection of Cancer	Cancers	Records excluded after screening

716	Micallef, J. V.	2016	Epigenetically altered circulating nucleosomes as blood biomarkers for early detection of cancer: Clinical studies in NSCLC, CRC, PCA and PC	Cancer Research	Records excluded after screening
717	Michael Traeger, M., J. Rehkaemper, H. Ullerich, K. Steinestel, E. Wardelmann, N. Senninger and S. Abdallah Dhayat	2018	The ambiguous role of microRNA205 and its clinical potential in pancreatic ductal adenocarcinoma	Journal of Cancer Research and Clinical Oncology	Not early-stage
718	Michálková, L., Š. Horník, J. Sýkora, L. Habartová and V. Setnička	2018	Diagnosis of pancreatic cancer via1H NMR metabolomics of human plasma	The Analyst	Records excluded after screening
719	Middleton, G., D. H. Palmer, W. Greenhalf, P. Ghaneh, R. Jackson, T. Cox, A. Evans, V. E. Shaw, J. Wadsley, J. W. Valle and et al.	2017	Vandetanib plus gemcitabine versus placebo plus gemcitabine in locally advanced or metastatic pancreatic carcinoma (ViP): a prospective, randomised, double-blind, multicentre phase 2 trial	The lancet. Oncology	Records excluded after screening
720	Miki, M., T. Ito, M. Hijioka, K. Kawabe and R. T. Jensen	2017	Utility of serum chromogranin B compared with chromogranin a as a biomarker in japanese patients with pancreatic neuroendocrine tumors	Neuroendocrinology	Records excluded after screening
721	Miles, H. N., D. G. Delafield and L. Li	2021	Recent developments and applications of quantitative proteomics strategies for high-throughput biomolecular analyses in cancer research	RSC Chemical Biology	Records excluded after screening
722	Min, L., D. Ziyu, Z. Xiaofei, X. Shunhe and W. Bolin	2022	Analysis of levels and Clinical value of CA19-9, NLR and SIRI in patients with Pancreatic Cancer with different Clinical Features	Cell Mol Biol (Noisy-le-grand)	Not early-stage
723	Mirus, J. E., Y. Zhang, C. I. Li, A. E. Lokshin, R. L. Prentice, S. R. Hingorani and P. D. Lampe	2015	Cross-species antibody microarray interrogation identifies a 3-protein panel of plasma biomarkers for early diagnosis of pancreas cancer	Clin Cancer Res	Records excluded after screening
724	Mishra, P. J.	2014	MicroRNAs as promising biomarkers in cancer diagnostics	Biomarker Research	Records excluded after screening
725	Mitachi, K., K. Ariake, H. Shima, S. Sato, T. Miura, S. Maeda, M. Ishida, M. Mizuma, H. Ohtsuka, T. Kamei, K. Igarashi and M. Unno	2021	Novel candidate factors predicting the effect of S-1 adjuvant chemotherapy of pancreatic cancer	Scientific reports	Records excluded after screening
726	Miura, Y., E. Brown, J. Davydova and M. Yamamoto	2014	Pancreatic cancer-selective adenovirus with redesigned ab-loop made via adenovirus library shows specificity mesothelin expressing cells	Journal of Gene Medicine	Non-diagnostic studies
727	Mizukami, Y., Y. Ono, H. Karasaki, K. Koizumi, S. Asahara, K. Takahashi and H. Maguchi	2016	Plasma DNA genotyping using digital PCR: Novel diagnostic tool for early detection of pancreatic cancer	Pancreatology	Records excluded after screening

728	Mizukami, Y., Y. Ono, H. Karasaki, M. Ogata, A. Sugitani, K. Koizumi, S. Asahara, K. Kawakubo, K. Takahashi, H. Maguchi and K. Nagashima	2016	Liquid biopsy for early detection of pancreatic cancer	Pancreas	Records excluded after screening
729	Mizukami, Y., Y. Ono, H. Karasaki, T. Okada and J. Sasajima	2018	Digital PCR-based liquid biopsy for early detection of pancreatic cancer	Cancer Science	Records excluded after screening
730	Modlin, I. M., M. Kidd, K. Oberg, M. Falconi, P. L. Filosso, A. Frilling, A. Malczewska, R. Salem, C. Toumpanakis, F. M. Laskaratos, S. Partelli, M. Roffinella, C. von Arx, B. K. Kudla, L. Bodei, I. A. Drozdov and A. Kitz	2021	Early Identification of Residual Disease After Neuroendocrine Tumor Resection Using a Liquid Biopsy Multigenomic mRNA Signature (NETest)	Annals of Surgical Oncology	Records excluded after screening
731	Mohamed, A. A., H. Soliman, M. Ismail, D. Ziada, T. M. Farid, A. M. Aref, M. E. Al Daly and Z. Y. Abd Elmageed	2015	Evaluation of circulating ADH and MIC-1 as diagnostic markers in Egyptian patients with pancreatic cancer	Pancreatology	Studies included in review
732	Mohammadi, M., F. Mianabadi and H. Mehrad-Majd	2019	Circulating visfatin levels and cancers risk: A systematic review and meta-analysis	Journal of Cellular Physiology	Records excluded after screening
733	Molnár, B., K. Tóth, B. K. Barták and Z. Tulassay	2015	Plasma methylated septin 9: A colorectal cancer screening marker	Expert Review of Molecular Diagnostics	Records excluded after screening
734	Moon, M. A.	2014	MicroRNA screening might reveal pancreatic cancer	Oncology Report	Records excluded after screening
735	Morizane, C.	2019	Genome-based chemotherapy for familial pancreatic cancer	Pancreas	Records excluded after screening
736	Mortimer, S., K. Dilger, D. Abdueva, D. Chudova, A. Sarin, C. E. Atreya, J. Leng, J. Lee, H. Eltoukhy, P. N. Munster and A. A. Talasaz	2016	Early, molecular detection of cancer utilizing circulating cell-free DNA assay with ultra high accuracy and sensitivity	Journal of Clinical Oncology	Records excluded after screening
737	Mortoglou, M., Z. K. Tabin, E. D. Arisan, H. M. Kocher and P. Uysal-Onganer	2021	Non-coding RNAs in pancreatic ductal adenocarcinoma: New approaches for better diagnosis and therapy	Translational Oncology	Records excluded after screening
738	Mosier, B. R. and L. E. Bantis	2021	Estimation and construction of confidence intervals for biomarker cutoff-points under the shortest Euclidean distance from the ROC surface to the perfection corner	Statistics in Medicine	Non-diagnostic studies

739	Moutinho-Ribeiro, P., I. A. Batista, S. T. Quintas, B. Adem, M. Silva, R. Morais, A. Peixoto, R. Coelho, P. Costa-Moreira, R. Medas, S. Lopes, F. Vilas-Boas, M. Baptista, D. Dias-Silva, A. L. Esteves, F. Martins, J. Lopes, H. Barroca, F. Carneiro, G. Macedo and S. A. Melo	2022	Exosomal glypican-1 is elevated in pancreatic cancer precursors and can signal genetic predisposition in the absence of endoscopic ultrasound abnormalities	World Journal of Gastroenterology	Non-diagnostic studies
740	Mulder, F. I., N. Kraaijpoel, M. Carrier, N. A. Guman, L. Jara-Palomares, M. Di Nisio, W. Ageno, J. Beyer-Westendorf, F. A. Klok, T. Vanassche, H. M. B. Otten, B. Cosmi, M. T. Wolde, S. G. J. G. In 't Veld, E. Post, J. Ramaker, K. Zwaan, M. Peters, A. Delluc, P. W. Kamphuisen, V. Sanchez-Lopez, E. Porreca, P. M. M. Bossuyt, H. R. Büller, T. Wurdinger, M. G. Best and N. van Es	2023	Platelet RNA sequencing for cancer screening in patients with unprovoked venous thromboembolism: a prospective cohort study	Journal of Thrombosis and Haemostasis	Records excluded after screening
741	Mulvihill, S.	2014	Screening for pancreatic cancer: Gold or dross?	HPB	Records excluded after screening
742	Munugala, N., H. Hammad, L. Vrba, M. Oshiro, B. Wertheim, D. Roe, H. Gavini, L. Latura, D. Pennington, S. Dalgai, H. M. Babiker, E. Elquza, A. J. Scott, M. Nelson, B. W. Futscher and R. T. Shroff	2020	Detection of pancreatic cancer using a novel blood-based DNA methylation signature	Journal of Clinical Oncology	Records excluded after screening
743	Murakami, M., Y. Nagai, A. Tenjin and Y. Tanaka	2018	Proposed cut-off value of CA19-9 for detecting pancreatic cancer in patients with diabetes: a case-control study	Endocr J	No specific biomarkers provided
744	Mussá, N. Y., V. Muzyca, J. Sampaio Matias and J. Melo Cristino	2021	Tumor markers in abdominal and pelvic tumours- myths and facts unfolded	Clinical Chemistry and Laboratory Medicine	Records excluded after screening

745	Myint, N. N. M., A. M. Verma, D. Fernandez-Garcia, P. Sarmah, P. S. Tarpey, S. S. Al-Aqbi, H. Cai, R. Trigg, K. West, L. M. Howells, A. Thomas, K. Brown, D. S. Guttery, B. Singh, H. J. Pringle, U. McDermott, J. A. Shaw and A. Rufini	2018	Circulating tumor DNA in patients with colorectal adenomas: assessment of detectability and genetic heterogeneity	Cell Death Dis	Records excluded after screening
746	Nagai, K., M. Kuwatani, K. Hirata, G. Suda, H. Hirata, Y. Takishin, R. Furukawa, K. Kishi, H. Yonemura, S. Nozawa, R. Sugiura, K. Kawakubo and N. Sakamoto	2022	Genetic Analyses of Cell-Free DNA in Pancreatic Juice or Bile for Diagnosing Pancreatic Duct and Biliary Tract Strictures	Diagnostics	Records excluded after screening
747	Nagayoshi, Y., M. Nakamura, K. Matsuoka, T. Ohtsuka, Y. Mori, H. Kono, T. Aso, N. Ideno, S. Takahata, A. Ryo, H. Takeda, T. Ito, Y. Oda, Y. Endo, T. Sawasaki and M. Tanaka	2014	Profiling of Autoantibodies in Sera of Pancreatic Cancer Patients	Annals of Surgical Oncology	Not early-stage
748	Nair, G. K. K., J. W. G. Yoo, Y. Gao, G. Desir, F. S. Gorelick, J. J. Farrell, G. G. Foster and N. Joshi	2020	ORGANOID-BASED PRECLINICAL MODELS RECAPITULATE RENALASE SIGNALING IN PANCREATIC DUCTAL ADENOCARCINOMA	Gastroenterology	Records excluded after screening
749	Nakamura, K., G. Hernández, G. G. Sharma, Y. Wada, J. K. Banwait, N. González, J. Perea, F. Balaguer, H. Takamaru, Y. Saito, Y. Toiyama, Y. Kodera, C. R. Boland, L. Bujanda, E. Quintero and A. Goel	2022	A Liquid Biopsy Signature for the Detection of Patients With Early-Onset Colorectal Cancer	Gastroenterology	Records excluded after screening

750	Nakamura, K., S. Roy, Z. Zhu, E. Jun, H. Han, R. M. Munoz, S. Nishiwada, G. Sharma, D. Cridebring, F. Zenhausern, S. Kim, D. Roe, S. Darabi, I. W. Han, D. Evans, S. Yamada, M. Demure, S. A. Celinski, E. Borazanci, S. Tsai, J. Bolton, Y. Kodera, J. O. Park, S. C. Kim, X. Wang, D. Von Hoff and A. Goel	2022	An exosomal miRNA-based liquid biopsy signature for the noninvasive early detection of pancreatic ductal adenocarcinoma	Cancer Research	Records excluded after screening
751	Nakamura, K., Z. Zhu, S. Roy, E. Jun, H. Han, R. M. Munoz, S. Nishiwada, G. Sharma, D. Cridebring, F. Zenhausern, S. Kim, D. J. Roe, S. Darabi, I. W. Han, D. B. Evans, S. Yamada, M. J. Demeure, C. Becerra, S. A. Celinski, E. Borazanci, S. Tsai, Y. Kodera, J. O. Park, J. S. Bolton, X. Wang, S. C. Kim, D. Von Hoff and A. Goel	2022	An Exosome-based Transcriptomic Signature for Noninvasive, Early Detection of Patients With Pancreatic Ductal Adenocarcinoma: A Multicenter Cohort Study	Gastroenterology	Studies included in review
752	Nakamura, S., Y. Sadakari, T. Ohtsuka, T. Okayama, Y. Nakashima, Y. Gotoh, K. Saeki, Y. Mori, K. Nakata, Y. Miyasaka, H. Onishi, Y. Oda, M. Goggins and M. Nakamura	2019	Pancreatic Juice Exosomal MicroRNAs as Biomarkers for Detection of Pancreatic Ductal Adenocarcinoma	Annals of Surgical Oncology	Non-diagnostic studies
753	Nakano, R., S. Nishiumi, T. Kobayashi, T. Ikegawa, Y. Kodama and M. Yoshida	2020	Possibility of detecting intraductal papillary mucinous neoplasms using metabolite biomarkers for pancreatic cancer	Biomarkers in Medicine	Not early-stage
754	Nakano, Y., G. Shimane, K. Nakamura, R. Takamatsu, E. Aimono, H. Yagi, A. B. E. Yuta, Y. Hasegawa, S. Hori, M. Tanaka, Y. Masugi, M. Kitago, H. Nishihara and Y. Kitagawa	2024	Next generation sequencing to identify genetic mutations in pancreatic cancer using intraoperative peritoneal washing fluid	Oncology Letters	Records excluded after screening

755	Nakaoka, K., E. Ohno, N. Kawabe, T. Kuzuya, K. Funasaka, Y. Nakagawa, M. Nagasaka, T. Ishikawa, A. Watanabe, T. Tochio, R. Miyahara, T. Shibata, H. Kawashima, S. Hashimoto and Y. Hirooka	2023	Current Status of the Diagnosis of Early-Stage Pancreatic Ductal Adenocarcinoma	Diagnostics	Records excluded after screening
756	Nakatsubo, R., Y. Harada, K. Yamamoto, T. Horibe, T. Hirotsu, T. Sugimoto, Y. Imaizumi, Y. Shibata and M. Tatsuzawa	2020	Usefulness of c.elegans for gastrointestinal cancer screening test based on smell of urine sample	United European Gastroenterology Journal	Records excluded after screening
757	Nam, H., S. S. Hong, K. H. Jung, S. Kang, M. S. Park, S. Kang, H. S. Kim, V. H. Mai, J. Kim, H. Lee, W. Lee, Y. J. Suh, J. H. Lim, S. Y. Kim, S. C. Kim, S. H. Kim and S. Park	2022	A Serum Marker for Early Pancreatic Cancer With a Possible Link to Diabetes	Journal of the National Cancer Institute	Studies included in review
758	Namkung, J., Y. Choi, W. J. Lee, J. S. Heo, J. K. Park, D. Y. Oh, S. C. Kim, C. M. Kang, T. Park, Y. Kim and J. Y. Jang	2015	Novel biomarker panel for the early detection of pancreatic cancer in the blood	United European Gastroenterology Journal	Records excluded after screening
759	Nannini, G., G. Meoni, A. Amedei and L. Tenori	2020	Metabolomics profile in gastrointestinal cancers: Update and future perspectives	World J Gastroenterol	Records excluded after screening
760	Nct	2022	Targeted Therapy Directed by Genetic Testing in Treating Patients With Locally Advanced or Advanced Solid Tumors, The ComboMATCH Screening Trial	https://clinicaltrials.gov/ct2/show/NCT05564377	Records excluded after screening
761	Nct	2023	Circulating Tumor DNA Methylation Guided Postoperative Adjuvant Chemotherapy for High-risk Stage II/III Colorectal Cancer	https://clinicaltrials.gov/ct2/show/NCT05954078	Records excluded after screening
762	Ndow, G., E. Vo-Quang, Y. Shimakawa, A. Ceesay, S. Tamba, H. F. Njai, L. Bojang, C. Hateley, Y. Takao, E. Opoku, Z. Warsop, P. Ingiliz, U. D'Alessandro, I. Chemin, M. Mendy, M. Thursz, R. Njie and M. Lemoine	2023	Clinical characteristics and outcomes of patients with cirrhosis and hepatocellular carcinoma in The Gambia, west Africa: a prospective cohort study	The Lancet Global Health	Records excluded after screening

763	Neggers, J. E., B. Paolella, A. Asfaw, M. V. Rothberg, T. A. Skipper, R. Kalekar, M. Burger, N. Dharia, G. Kugener, J. Kalfon, N. Dumont, Y. Li, L. Spurr, A. Yang, W. Wu, A. Durbin, B. M. Wolpin, D. E. Root, J. Boehm, A. D. Cherniack, A. Tsherniak, A. L. Hong, W. C. Hahn, K. Stegmaier, T. Golub, F. Vazquez and A. J. Aguirre	2021	Synthetic lethal interaction between the ESCRT paralog enzymes VPS4A and VPS4B in cancers harboring loss of chromosome 18q or 16q	Cancer Research	Records excluded after screening
764	Nené, N. R., A. Ney, T. Nazarenko, O. Blyuss, H. E. Johnston, H. J. Whitwell, E. Sedlak, A. Gentry-Maharaj, S. Apostolidou, E. Costello, W. Greenhalf, I. Jacobs, U. Menon, J. S. Hsuan, S. P. Pereira, A. Zaikin and J. F. Timms	2023	Serum biomarker-based early detection of pancreatic ductal adenocarcinomas with ensemble learning	Communications Medicine	Studies excluded after qualitative synthesis
765	Nené, N. R., A. Ney, T. Nazarenko, O. Blyuss, H. E. Johnston, H. J. Whitwell, E. Sedlak, A. Gentry-Maharaj, E. Costello, W. Greenhalf, I. Jacobs, U. Menon, J. Hsuan, S. P. Pereira, A. Zaikin and J. F. Timms .	2021	Early detection of pancreatic ductal adenocarcinomas with an ensemble learning model based on a panel of protein serum biomarkers.		Records excluded after screening
766	Nesteruk, K., I. J. M. Levink, E. de Vries, I. J. Visser, M. P. Peppelenbosch, D. L. Cahen, G. M. Fuhler and M. J. Bruno	2022	Extracellular vesicle-derived microRNAs in pancreatic juice as biomarkers for detection of pancreatic ductal adenocarcinoma	Pancreatology	Not early-stage
767	Ney, A., A. Garcia-Sampedro, G. Goodchild, P. Acedo, G. Fusai and S. P. Pereira	2021	Biliary Strictures and Cholangiocarcinoma – Untangling a Diagnostic Conundrum	Frontiers in Oncology	Records excluded after screening
768	Ney, A., N. R. Nené, E. Sedlak, P. Acedo, O. Blyuss, H. J. Whitwell, E. Costello, A. Gentry-Maharaj, N. R. Williams, U. Menon, G. K. Fusai, A. Zaikin and S. P. Pereira	2023	Identification of a serum proteomic biomarker panel using diagnosis specific ensemble learning and symptoms for early pancreatic cancer detection.		Records excluded after screening

769	Nicholson, J. A., W. Greenhalf, R. Jackson, T. F. Cox, J. V. Butler, T. Hanna, S. Harrison, C. J. Grocock, C. M. Halloran, N. R. Howes, M. G. Raraty, P. Ghaneh, M. Johnstone, S. Sarkar, H. L. Smart, J. C. Evans, G. P. Aithal, R. Sutton, J. P. Neoptolemos and M. G. Lombard	2015	Incidence of post-ERCP pancreatitis from direct pancreatic juice collection in hereditary pancreatitis and familial pancreatic cancer before and after the introduction of prophylactic pancreatic stents and rectal diclofenac	Pancreas	Records excluded after screening
770	Nicoletti, A., M. Negri, M. Paratore, F. Vitale, M. E. Ainora, E. C. Nista, A. Gasbarrini, M. A. Zocco and L. Zileri Dal Verme	2023	Diagnostic and Prognostic Role of Extracellular Vesicles in Pancreatic Cancer: Current Evidence and Future Perspectives	International Journal of Molecular Sciences	Records excluded after screening
771	Nie, R. C., X. B. Zou, S. Q. Yuan, Y. B. Chen, S. Chen, Y. M. Chen, G. M. Chen, X. J. Chen, T. Q. Luo, S. M. Li, J. L. Duan, Y. Wang and Y. F. Li	2020	Disease-free survival as a surrogate endpoint for overall survival in adjuvant trials of pancreatic cancer: a meta-analysis of 20 randomized controlled trials	BMC Cancer	Records excluded after screening
772	Nie, S., A. Lo, J. Wu, J. Zhu, Z. Tan, D. M. Simeone, M. A. Anderson, K. A. Shedden, M. T. Ruffin and D. M. Lubman	2014	Glycoprotein biomarker panel for pancreatic cancer discovered by quantitative proteomics analysis	J Proteome Res	Incomplete diagnostic performance data provided
773	Nikolic, A.	2014	Noninvasive Early Markers in Pancreatic Cancer.		Records excluded after screening
774	Ning, L., H. Cheng, F. Yu, Y. Zhou and Y. Xie	2022	Construction of simple and sensitive pancreatitis related microRNA detection strategy via self-priming triggered cascade signal amplification	Anal Bioanal Chem	Records excluded after screening
775	Nofal, Y. H., Y. Abu Dail, Y. Assaf, H. Abo Samra, F. Abbas, A. Hamzeh and N. Alhaj Hasan	2018	Pancreatic enzyme replacement therapy for steatorrhoea in pancreatic cancer	Cochrane Database of Systematic Reviews	Records excluded after screening
776	Nogueira, L. M., C. C. Newton, M. Pollak, D. T. Silverman, D. Albanes, S. Mannisto, S. J. Weinstein, E. J. Jacobs and R. Z. Stolzenberg-Solomon	2017	Serum C-peptide, total and high molecular weight adiponectin, and pancreatic cancer: Do associations differ by smoking?	Cancer Epidemiology Biomarkers and Prevention	Records excluded after screening

777	Nolen, B. M., R. E. Brand, D. Prosser, L. Velikokhatnaya, P. J. Allen, H. J. Zeh, W. E. Grizzle, Y. Huang, A. Lomakin and A. E. Lokshin	2014	Prediagnostic serum biomarkers as early detection tools for pancreatic cancer in a large prospective cohort study	PLoS One	Records excluded after screening
778	Nolen, B. M., W. Grizzle, R. Brand and A. E. Lokshin	2014	Urine and serum biomarkers of pancreatic cancer	Cancer Research	Records excluded after screening
779	O'Brien, D. P., N. S. Sandanayake, C. Jenkinson, A. Gentry-Maharaj, S. Apostolidou, E. O. Fourkala, S. Camuzeaux, O. Blyuss, R. Gunu, A. Dawnay, A. Zaikin, R. C. Smith, I. J. Jacobs, U. Menon, E. Costello, S. P. Pereira and J. F. Timms	2015	Serum CA19-9 Is Significantly Upregulated up to 2 Years before Diagnosis with Pancreatic Cancer: Implications for Early Disease Detection	Clinical Cancer Research	Not early-stage
780	Oikonomou, E., M. Anastasiou, G. Siasos, E. Androulakis, A. Psyrri, K. Toutouzas and D. Tousoulis	2018	Cancer therapeutics-related cardiovascular complications. Mechanisms, diagnosis and treatment	Current Pharmaceutical Design	Records excluded after screening
781	Okada, T., Y. Mizukami, Y. Ono, H. Sato, A. Hayashi, H. Kawabata, K. Koizumi, S. Masuda, S. Teshima, K. Takahashi, A. Katanuma, Y. Omori, H. Iwano, M. Yamada, T. Yokochi, S. Asahara, K. Kawakubo, M. Kuwatani, N. Sakamoto, K. Enomoto, T. Goto, J. Sasajima, M. Fujiya, J. Ueda, S. Matsumoto, K. Taniue, A. Sugitani, H. Karasaki and T. Okumura	2020	Digital PCR-based plasma cell-free DNA mutation analysis for early-stage pancreatic tumor diagnosis and surveillance	Journal of Gastroenterology	Not early-stage
782	Okano, K. and Y. Suzuki	2014	Strategies for early detection of resectable pancreatic cancer	World Journal of Gastroenterology	Records excluded after screening
783	Okuno, K., S. Watanabe, J. Bolton, M. Tokunaga, Y. Kinugasa and A. Goel	2024	DNA METHYLATION-BASED LIQUID BIOPSY SIGNATURE FOR THE EARLY DETECTION OF PATIENTS WITH PANCREATIC DUCTAL ADENOCARCINOMA	Gastroenterology	Records excluded after screening

784	Oldfield, L., R. Hanson, W. Greenhalf, V. D. Meer R, L. Alison, P. Christiansen, P. Ghaneh, T. Purewal, P. Dan, E. Psarelli, C. Halloran and E. Costello	2020	UK Early Detection Initiative (UK-EDI) for Pancreatic Cancer	Pancreatology	Records excluded after screening
785	Oldfield, L. E., L. Penney, M. Basik, W. Foulkes, C. Elser, L. Murphy, I. Schrader, A. Karsan, A. Pollett, Y. Bombard, R. H. Kim and T. J. Pugh	2022	CHARM Consortium: Early cancer detection in BRCA1 and BRCA2 carriers using cell-free DNA sequencing	Familial Cancer	Records excluded after screening
786	Oliveira, B. B., B. Costa, B. Morao, S. Faias, B. Veigas, L. P. Pereira, C. Albuquerque, R. Maio, M. Cravo, A. R. Fernandes and P. V. Baptista	2023	Combining the amplification refractory mutation system and high-resolution melting analysis for <i>KRAS</i> mutation detection in clinical samples	Analytical and Bioanalytical Chemistry	Records excluded after screening
787	Oluyemi, O. I., S. Luu, H. R. De La Baume and F. Goldwire	2021	Smoke but no fire: A case of massive CA 19-9 elevation from nsaid use	American Journal of Gastroenterology	Records excluded after screening
788	O'Neill, R. S., S. Emmanuel, D. Williams and A. Stoita	2020	Macrophage inhibitory cytokine-1/growth differentiation factor-15 in premalignant and neoplastic tumours in a high-risk pancreatic cancer cohort	World J Gastroenterol	Not early-stage
789	O'Neill, R. S. and A. Stoita	2021	Biomarkers in the diagnosis of pancreatic cancer: Are we closer to finding the golden ticket?	World Journal of Gastroenterology	Records excluded after screening
790	Oni, T. E., C. Bautista, B. Nelson and D. A. Tuveson	2016	Identification of tumor-specific antibodies using phage display selections on pancreatic ductal organoids	Cancer Research	Records excluded after screening
791	Ono, Y., A. Sugitani, H. Karasaki, M. Ogata, R. Nozaki, J. Sasajima and Y. Mizukami	2017	An improved digital polymerase chain reaction protocol to capture low-copy KRAS mutations in plasma cell-free DNA by resolving " subsampling" issues	United European Gastroenterology Journal	Not early-stage
792	Orlowski, M. A., W. R. Bamlet, A. L. Oberg, G. M. Petersen, S. T. Chari, A. Topilow, J. Davis and C. Gilvarg	2014	Serum procarboxypeptidase combined with CA19-9 and accuracy of detection of pancreatic cancer	Journal of Clinical Oncology	Records excluded after screening
793	Osei, E., C. Oghinan, A. Asare, H. Ho and S. Manful	2022	Review of clinical and emerging biomarkers for early diagnosis and treatment management of pancreatic cancer: Towards personalised medicine	Journal of Radiotherapy in Practice	Records excluded after screening

794	Oto, J., S. Navarro, A. C. Larsen, M. J. Solmoirago, E. Plana, D. Hervás, Á. Fernández-Pardo, F. España, S. R. Kristensen, O. Thorlacius-Ussing and P. Medina	2020	MicroRNAs and neutrophil activation markers predict venous thrombosis in pancreatic ductal adenocarcinoma and distal extrahepatic cholangiocarcinoma	International Journal of Molecular Sciences	Records excluded after screening
795	Overbeek, K. A., I. J. M. Levink, B. D. M. Koopmann, F. Harinck, I. C. A. W. Konings, M. G. E. M. Ausems, A. Wagner, P. Fockens, C. H. Van Eijck, B. Groot Koerkamp, O. R. C. Busch, M. G. Besselink, B. A. J. Bastiaansen, L. M. J. W. Van Driel, N. S. Erler, F. P. Vleggaar, J. W. Poley, D. L. Cahen, J. E. Van Hooft and M. J. Bruno	2022	Long-term yield of pancreatic cancer surveillance in high-risk individuals	Gut	Non-diagnostic studies
796	Ozaki, T., M. Yu, D. Yin, D. Sun, Y. Zhu, Y. Bu and M. Sang	2018	Impact of RUNX2 on drug-resistant human pancreatic cancer cells with p53 mutations	BMC Cancer	Records excluded after screening
797	Pal, A., A. Ojha and J. Ju	2023	Functional and Potential Therapeutic Implication of MicroRNAs in Pancreatic Cancer	International Journal of Molecular Sciences	Records excluded after screening
798	Pan, C. H., Y. Otsuka, B. Sridharan, M. Woo, C. V. Leiton, S. Babu, M. Torrente Gonçalves, R. R. Kawalerski, K. B. JD, D. K. Chang, A. V. Biankin, L. Scampavia, T. Spicer, L. F. Escobar-Hoyos and K. R. Shroyer	2020	An unbiased high-throughput drug screen reveals a potential therapeutic vulnerability in the most lethal molecular subtype of pancreatic cancer	Mol Oncol	Records excluded after screening
799	Pan, H., L. Sun, W. Wang, Z. Qin, L. Tang, X. Gu, J. Zhang and B. He	2020	Serum long non-coding RNA LOC553103 as non-specific diagnostic and prognostic biomarker for common types of human cancer	Clinica Chimica Acta	Records excluded after screening
800	Pang, Y., M. V. Holmes, Z. Chen and C. Kartsonaki	2019	A review of lifestyle, metabolic risk factors, and blood-based biomarkers for early diagnosis of pancreatic ductal adenocarcinoma	Journal of Gastroenterology and Hepatology (Australia)	Records excluded after screening
801	Pang, Y., C. Wang, L. Lu, C. Wang, Z. Sun and R. Xiao	2019	Dual-SERS biosensor for one-step detection of microRNAs in exosome and residual plasma of blood samples for diagnosing pancreatic cancer	Biosens Bioelectron	Records excluded after screening

802	Papadopoulos, N., Y. Wang, J. Cohen, R. Hruban, P. Gibbs, J. Tie, L. Diaz, K. Kinzler and B. Vogelstein	2017	Non-invasive detection of somatic mutations in the management of CRC	Cancer Research	Records excluded after screening
803	Papapanagiotou, A., G. Sgourakis, K. Karkoulias, D. Raptis, E. Parkin, P. Brotzakis, S. Panchal and A. G. Papavassiliou	2018	Osteonectin as a screening marker for pancreatic cancer: A prospective study	Journal of International Medical Research	Records excluded after screening
804	Papi, M., V. Palmieri, L. Digiacomo, F. Giulimondi, S. Palchetti, G. Ciasca, G. Perini, D. Caputo, M. C. Cartillone, C. Cascone, R. Coppola, A. L. Capriotti, A. Laganà, D. Pozzi and G. Caracciolo	2019	Converting the personalized biomolecular corona of graphene oxide nanoflakes into a high-throughput diagnostic test for early cancer detection	Nanoscale	No sample information provided
805	Park, J., Y. Choi, J. Namkung, S. G. Yi, H. Kim, J. Yu, Y. Kim, M. S. Kwon, W. Kwon, D. Y. Oh, S. W. Kim, S. Y. Jeong, W. Han, K. E. Lee, J. S. Heo, J. O. Park, J. K. Park, S. C. Kim, C. M. Kang, W. J. Lee, S. Lee, S. Han, T. Park, J. Y. Jang and Y. Kim	2017	Diagnostic performance enhancement of pancreatic cancer using proteomic multimarker panel	Oncotarget	Incomplete diagnostic performance data provided
806	Park, M. S., K. H. Jung, S. E. Kim, Y. J. Lee, S. Park and S. S. Hong	2023	A novel biomarker and therapeutic target for early pancreatic cancer	Cancer Research	Records excluded after screening
807	Patel, H. Y. and I. Mukherjee	2022	A Novel Neural Network to Predict Locally Advanced Pancreatic Cancer Using 4 Urinary Biomarkers: REG1A/1B, LYVE1, and TFF1	Journal of the American College of Surgeons	Records excluded after screening
808	Pelling, M., S. Chandrapalan, E. West and R. P. Arasaradnam	2023	A Systematic Review and Meta-Analysis: Volatile Organic Compound Analysis in the Detection of Hepatobiliary and Pancreatic Cancers	Cancers	Records excluded after screening
809	Peng, H. Y., M. C. Chang, C. M. Hu, H. I. Yang, W. H. Lee and Y. T. Chang	2019	Thrombospondin-2 is a Highly Specific Diagnostic Marker and is Associated with Prognosis in Pancreatic Cancer	Annals of Surgical Oncology	Not early-stage
810	Peng, L., M. Lin, T. C. Chu, M. Sun and X. Gao	2019	Cancer screening using 18F-FDG PET/MR in asymptomatic subjects: A Retrospective Study	Journal of Nuclear Medicine	Records excluded after screening
811	Penitenti, F., L. Landoni, M. Scardoni, M. L. Piredda, S. Cingarlini, A. Scarpa, M. D'Onofrio, D. Girelli and M. V. Davi	2021	Clinical presentation, genotype–phenotype correlations, and outcome of pancreatic neuroendocrine tumors in Von Hippel–Lindau syndrome	Endocrine	Non-diagnostic studies

812	Perazzoli, G., O. M. García-Valdeavero, M. Peña, J. Prados, C. Melguizo and C. Jiménez-Luna	2023	Evaluating Metabolite-Based Biomarkers for Early Diagnosis of Pancreatic Cancer: A Systematic Review	Metabolites	Records excluded after screening
813	Perera, C. J., M. Falasca, S. T. Chari, J. R. Greenfield, Z. Xu, R. C. Pirola, J. S. Wilson and M. V. Apte	2021	Role of pancreatic stellate cell-derived exosomes in pancreatic cancer-related diabetes: A novel hypothesis	Cancers	Records excluded after screening
814	Permuth, J. B.	2021	Using an integrative molecular epidemiology approach to battle pancreatic cancer in the era of precision medicine: Hope is on the horizon	Cancer Research	Records excluded after screening
815	Permuth, J. B., C. Georgeades and M. Malafa	2015	MiRNAs as biomarkers of high-risk pancreatic cysts: A possible holy grail for the early detection of pancreatic cancer	Future Oncology	Records excluded after screening
816	Peschke, K., H. Jakubowsky, A. Schäfer, C. Maurer, S. Lange, F. Orben, R. Bernad, F. N. Harder, M. Eiber, R. Öllinger, K. Steiger, M. Schlitter, W. Weichert, U. Mayr, V. Phillip, C. Schlag, R. M. Schmid, R. F. Braren, B. Kong, I. E. Demir, H. Friess, R. Rad, D. Saur, G. Schneider and M. Reichert	2022	Identification of treatment-induced vulnerabilities in pancreatic cancer patients using functional model systems	Embo Molecular Medicine	Records excluded after screening
817	Peters, M. L. B., A. Eckel, C. L. Seguin, B. Davidi, D. H. Howard, A. B. Knudsen and P. V. Pandharipande	2024	Cost-Effectiveness Analysis of Screening for Pancreatic Cancer Among High-Risk Populations	JCO Oncology Practice	Records excluded after screening
818	Petra, E., K. Katarina, J. Anna, P. Michael, B. Vladimir and G. Robert	2016	Circulating tumor cells in tumors of gastrointestinal tract - Personalized therapy	European Surgery - Acta Chirurgica Austriaca	Records excluded after screening
819	Petre, I., M. Ilie, C. Bleotu, V. Şandru, O. Plotogea and G. Constantinescu	2018	Early diagnosis of pancreatic cancer by determining genetic and serological tumoral markers	Archives of the Balkan Medical Union	Records excluded after screening
820	Petrone, M. C. and P. G. Arcidiacono	2016	New strategies for the early detection of pancreatic cancer	Expert Rev Gastroenterol Hepatol	Records excluded after screening
821	Pezzilli, R., L. Calculli, G. Melzi d'Eril and A. Barassi	2016	Serum tumor markers not useful in screening patients with pancreatic mucinous cystic lesions associated with malignant changes	Hepatobiliary Pancreat Dis Int	Non-diagnostic studies
822	Phan, V. A., P. Saxena, A. Stoita and N. Nguyen	2016	Pancreatic cancer diagnosis and screening	Cancer Forum	Records excluded after screening

823	Pietrasz, D., E. Sereni, F. Lancelotti, A. Pea, C. Luchini, G. Innamorati, R. Salvia and C. Bassi	2022	Circulating tumour DNA: a challenging innovation to develop " precision onco-surgery" in pancreatic adenocarcinoma	British Journal of Cancer	Records excluded after screening
824	Pina, F., F. Botelho, T. Lopes, I. Lopes, G. Figueiredo, R. Portugal, A. Ferro, F. Cruz, H. Barros and N. Lunet	2014	Can serum angiogenin be used to improve the diagnostic performance in prostate cancer screening?	European Journal of Cancer Prevention	Records excluded after screening
825	Ping, C. and Y. WenYuan	2019	Application of combined detection of CEA, AFP, CA199 and CA50 in screening for gastrointestinal tumours in healthy individuals	Acta Medica Mediterranea	Records excluded after screening
826	Pogacar, Z., K. Groot, F. Jochems, M. Dos Santos Dias, B. Morris, M. Roosen, L. Wardak, G. De Conti, A. Velds, C. Lieftink, R. L. Beijersbergen, R. Bernards and R. L. De Oliveira	2021	Genetic and compound screens uncover factors modulating cancer cell response to indisulam.		Records excluded after screening
827	Pollini, T., L. Kone, W. Brugge, C. Fernandez-del Castillo, J. Winter, C. Yeo, T. Hackert, M. Buechler, K. Roggin, C. M. Schmidt, M. Yip-Schneider, R. Salvia, C. Bassi, G. Chlipala and A. Maker	2022	Development of a Microfluidic Cyst Fluid Assay to Identify Pancreatic Cystic Neoplasms that Require Surgical Resection	HPB	Records excluded after screening
828	Pollini, T., P. Wong and A. V. Maker	2023	The Landmark Series: Intraductal Papillary Mucinous Neoplasms of the Pancreas—From Prevalence to Early Cancer Detection	Annals of Surgical Oncology	Records excluded after screening
829	Pons-Belda, O. D., A. Fernandez-Uriarte and E. P. Diamandis	2022	Multi Cancer Early Detection by Using Circulating Tumor DNA—The Galleri Test. Reply to Klein et al. The Promise of Multicancer Early Detection. Comment on "Pons-Belda et al. Can Circulating Tumor DNA Support a Successful Screening Test for Early Cancer Detection? The Grail Paradigm. Diagnostics 2021, 11, 2171"	Diagnostics	Records excluded after screening
830	Postel, M., A. Roosen, P. Laurent-Puig, V. Taly and S. F. Wang-Renault	2018	Droplet-based digital PCR and next generation sequencing for monitoring circulating tumor DNA: a cancer diagnostic perspective	Expert Review of Molecular Diagnostics	Records excluded after screening
831	Poteet, E., D. Liu, Z. Liang, G. Van Buren, C. Chen and Q. Yao	2019	Mesothelin and TGF-α predict pancreatic cancer cell sensitivity to EGFR inhibitors and effective combination treatment with trametinib	PLoS ONE	Records excluded after screening
832	Prasad, K. S., X. Y. Cao, N. Gao, Q. J. Jin, S. T. Sanjay, G. Henao-Pabon and X. J. Li	2020	A low-cost nanomaterial-based electrochemical immunosensor on paper for high-sensitivity early detection of pancreatic cancer	Sensors and Actuators B-Chemical	Records excluded after screening

833	Prassas, I., D. Brinc, S. Farkona, F. Leung, A. Dimitromanolakis, C. C. Chrystoja, R. Brand, V. Kulasingam, I. M. Blasutig and E. P. Diamandis	2014	False biomarker discovery due to reactivity of a commercial ELISA for CUZD1 with cancer antigen CA125	Clinical Chemistry	Non-diagnostic studies
834	Pratt, E. D., R. W. Cowan, S. L. Manning, E. Qiao, H. Cameron, K. Schradle, D. M. Simeone and D. B. Zhen	2019	Multiplex Enrichment and Detection of Rare KRAS Mutations in Liquid Biopsy Samples using Digital Droplet Pre-Amplification	Analytical chemistry	Records excluded after screening
835	Prieto-Fernández, L., S. T. Menéndez, M. Otero-Rosales, I. Montoro-Jiménez, F. Hermida-Prado, J. M. García-Pedrero and S. Álvarez-Teijeiro	2022	Pathobiological functions and clinical implications of annexin dysregulation in human cancers	Frontiers in Cell and Developmental Biology	Records excluded after screening
836	Prieur, A., T. Mazard, E. Assenat, M. Ychou, S. Gourgou, O. Mariani, T. Bronzini, A. Vincent-Salomon and D. Joubert	2017	Progastrin: A new specific early cancer screening biomarker	Journal of Clinical Oncology	Records excluded after screening
837	Pu, X., G. Ding, M. Wu, S. Zhou, S. Jia and L. Cao	2020	Elevated expression of exosomal microRNA–21 as a potential biomarker for the early diagnosis of pancreatic cancer using a tethered cationic lipoplex nanoparticle biochip	Oncology Letters	Records excluded after screening
838	Qiu, M., Y. Chen and C. Zeng	2024	Biological functions of circRNA in regulating the hallmarks of gastrointestinal cancer (Review)	International Journal of Oncology	Records excluded after screening
839	Qiu, S., Y. Cai, H. Yao, C. Lin, Y. Xie, S. Tang and A. Zhang	2023	Small molecule metabolites: discovery of biomarkers and therapeutic targets	Signal Transduction and Targeted Therapy	Records excluded after screening
840	Quirico, L. and F. Orso	2020	The power of microRNAs as diagnostic and prognostic biomarkers in liquid biopsies	Cancer Drug Resistance	Records excluded after screening
841	Radon, T. P., N. J. Massat, R. Jones, W. Alrawashdeh, L. Dumartin, D. Ennis, S. W. Duffy, H. M. Kocher, S. P. Pereira, L. Guarner, C. Murta-Nascimento, F. X. Real, N. Malats, J. Neoptolemos, E. Costello, W. Greenhalf, N. R. Lemoine and T. Crnogorac-Jurcevic	2015	Identification of a three-biomarker panel in urine for early detection of pancreatic adenocarcinoma	Clinical Cancer Research	Studies included in review

842	Rahmani, H., S. Mansouri Majd, A. Salimi and F. Ghasemi	2023	Ultrasensitive immunosensor for monitoring of CA 19-9 pancreatic cancer marker using electrolyte-gated TiS(3) nanoribbons field-effect transistor	Talanta	Records excluded after screening
843	Rahmanuddin, S., R. Korn, D. Cridebring, E. Borazanci, J. Brase, W. Boswell, A. Jamil, W. Cai, A. Sabir, P. Motarjem, E. Koay, A. Mitra, A. Goel, J. Ho, V. Chung and D. D. Von Hoff	2021	Role of 3D Volumetric and Perfusion Imaging for Detecting Early Changes in Pancreatic Adenocarcinoma	Frontiers in Oncology	Small sample size
844	Ramseier, J. Y. and S. H. Perkins	2022	Genodermatoses associated with melanocytic nevi	Clinics in Dermatology	Records excluded after screening
845	Ramshani, Z., C. G. Zhang, K. Richards, L. L. Chen, G. Y. Xu, B. L. Stiles, R. Hill, S. Senapati, D. B. Go and H. C. Chang	2019	Extracellular vesicle microRNA quantification from plasma using an integrated microfluidic device	Communications Biology	Records excluded after screening
846	Randazzo, O., S. M. Cascioferro, C. Pecoraro, W. A. Iddouch, A. Avan, B. Parrino, D. Carbone, U. Perricone, G. J. Peters, P. Diana and E. Giovannetti	2021	SF3B1 modulators affect key genes in metastasis and drug influx: A new approach to fight pancreatic cancer chemoresistance	Cancer Drug Resistance	Records excluded after screening
847	Randeu, H., A. J. Bronkhorst, Z. Mayer, A. Oberhofer, E. Polatoglou, V. Heinemann, M. Haas, S. Boeck and S. Holdenrieder	2022	Preanalytical Variables in the Analysis of Mitochondrial DNA in Whole Blood and Plasma from Pancreatic Cancer Patients	Diagnostics	Records excluded after screening
848	Ranković, B. and N. Hauptman	2023	Circulating microRNA Panels for Detection of Liver Cancers and Liver-Metastasizing Primary Cancers	International Journal of Molecular Sciences	Records excluded after screening
849	Rashid, S., S. Rashid, P. Das, N. Singh, N. R. Dash, B. Nayak, H. C. Sati, S. S. Chauhan, S. Gupta and A. Saraya	2023	Clinical significance of Notch pathway-associated microRNA-107 in pancreatic ductal adenocarcinoma	Future Oncology	Records excluded after screening
850	Raufi, A. G., M. S. May, M. J. Hadfield, A. A. Seyhan and W. S. El-Deiry	2023	Advances in Liquid Biopsy Technology and Implications for Pancreatic Cancer	International Journal of Molecular Sciences	Records excluded after screening
851	Rawashdeh, I., M. G. Al-Fandi, Y. Makableh and T. Harahsha	2021	Developing a nano-biosensor for early detection of pancreatic cancer	Sensor Review	Records excluded after screening
852	Real, M., W. M. Chalhoub and N. G. Haddad	2017	It's in her DNA: A case highlighting the role of dna molecular analysis in intraductal papillary mucinous neoplasm	American Journal of Gastroenterology	Records excluded after screening

853	Reddy, A., M. Pinzon-Ortiz, A. Derti, J. Korn, D. Ruddy, G. Yang, J. Green, H. Gao, J. Lehar, G. Caponigro and Z. A. Cao	2015	High allele frequency of KRAS functional mutations predicts resistance to MEK inhibitors: Evidence from cell lines and human tumor xenograft models	Cancer Research	Records excluded after screening
854	Reddy, P. S. and M. C. Sekhar	2022	AN EFFICIENT NOVEL APPROACH WITH MULTI CLASS LABEL CLASSIFICATION THROUGH MACHINE LEARNING MODELS FOR PANCREATIC CANCER	Scalable Computing-Practice and Experience	Records excluded after screening
855	Resovi, A., G. Taraboletti, A. Scarpa, R. T. Lawlor, R. Giavazzi and D. Belotti	2014	Identification of potential biomarkers in pancreatic ductal adenocarcinoma associated to tumor-stroma interaction	European Journal of Cancer	Records excluded after screening
856	Rezaei, S., E. Gharapapagh, S. Dabiri, P. Heidari and A. Aghanejad	2023	Theranostics in targeting fibroblast activation protein bearing cells: Progress and challenges	Life Sciences	Records excluded after screening
857	Rift, C. V., B. Kovacevic, A. Toxværd, P. Klausen, C. P. Hansen, P. Vilmann and J. P. Hasselby	2020	EUS-guided through-the-needle biopsy sampling of pancreatic cystic lesions: a pathologist's guide for the endoscopist	Gastrointestinal Endoscopy	Records excluded after screening
858	Ritchie, S., A. Hirofumi, I. Takemasa, F. Nomura, D. Jayasinghe, B. Chitou, Y. Yamazaki and D. Goodenowe	2014	Depleted levels of long-chain fatty acids and glycerophosphocholines in pancreatic cancer patient serum: Biomarkers of disease and increased risk	Gastroenterology	Records excluded after screening
859	Ritchie, S. A., B. Chitou, Q. Zheng, D. Jayasinghe, W. Jin, A. Mochizuki and D. B. Goodenowe	2015	Pancreatic cancer serum biomarker PC-594: Diagnostic performance and comparison to CA19-9	World Journal of Gastroenterology	Not early-stage
860	Riva, F., O. I. Dronov, D. I. Khomenko, F. Huguet, C. Louvet, P. Mariani, M. H. Stern, O. Lantz, C. Proudhon, J. Y. Pierga and F. C. Bidard	2016	Clinical applications of circulating tumor DNA and circulating tumor cells in pancreatic cancer	Molecular Oncology	Records excluded after screening
861	Roesli, C.	2015	Recent advances in proteomically subtyping pancreatic ductal adenocarcinomas and their potential clinical impact	Expert Review of Proteomics	Records excluded after screening
862	Roli, L., V. Pecoraro and T. Trenti	2017	Can NGAL be employed as prognostic and diagnostic biomarker in human cancers? A systematic review of current evidence	International Journal of Biological Markers	Records excluded after screening
863	Root, A., P. Allen, P. Tempst and K. Yu	2018	Protein biomarkers for early detection of pancreatic ductal adenocarcinoma: Progress and challenges	Cancers	Records excluded after screening

864	Rosenbaum, M. W., M. Jones, J. C. Dudley, L. P. Le, A. J. Iafrate and M. B. Pitman	2017	Next-generation sequencing adds value to the preoperative diagnosis of pancreatic cysts	Cancer Cytopathology	Not early-stage
865	Rosenthal, M. H. and K. Schawkat	2023	Beyond the AJR: CT Radiomic Features of the Pancreas Predict Development of Pancreatic Cancer	American Journal of Roentgenology	Records excluded after screening
866	Rosiek, V., K. Janas, M. Witkowska and B. Kos-Kudła	2023	Role of Selected Circulating Tumor Biomarkers in Patients with Skeletal Metastatic Pancreatic Neuroendocrine Neoplasms	Journal of Clinical Medicine	Not early-stage
867	Ross, S. B., I. Sucandy, T. Lippert, V. Przetocki, K. Crespo, T. J. Bourdeau and A. S. Rosemurgy	2020	Genetic Profiling of Pancreatic Ductal Adenocarcinomas: Predicts Survival or Just Alphabet Soup?	Journal of the American College of Surgeons	Records excluded after screening
868	Rossez, Y., C. Burtea, S. Laurent, P. Gosset, R. Léonard, W. Gonzalez, S. Ballet, I. Raynal, O. Rousseaux, T. Dugué, L. Vander Elst, J. C. Michalski, R. N. Muller and C. Robbe-Masselot	2016	Early detection of colonic dysplasia by magnetic resonance molecular imaging with a contrast agent raised against the colon cancer marker MUC5AC	Contrast Media & Molecular Imaging	Records excluded after screening
869	Rossi, M. L., A. A. Rehman and C. S. Gondi	2014	Therapeutic options for the management of pancreatic cancer	World J Gastroenterol	Records excluded after screening
870	Rossi, R. E., C. Ciafardini, D. Conte, V. Sciola and S. Massironi	2018	Chromogranin a as a marker in the follow up of gastro-entero-pancreatic neuroendocrine neoplasms (GEP-NENS). A systematic review	Digestive and Liver Disease	Records excluded after screening
871	Rossi, R. E., C. Ciafardini, V. Sciola, D. Conte and S. Massironi	2018	Chromogranin A in the Follow-up of Gastroenteropancreatic Neuroendocrine Neoplasms: Is It Really Game Over? A Systematic Review and Meta-analysis	Pancreas	Records excluded after screening
872	Roy, G. K.	2023	Plasma microrna-based highly predictive model for colorectal neoplasms diagnosis: Improving sensitivity and specificity	Journal of Cardiovascular Disease Research	Records excluded after screening
873	Roy, S., S. Rana, M. Kang, V. Gupta, H. Singh, R. Nada and R. Gupta	2022	CORRELATION OF IMAGING AND BIOCHEMICAL MODALITIES IN IDENTIFYING THE EXACT ANATOMICAL SITE OF ORIGIN OF TUMOUR IN PERIAMPULLARY CARCINOMA – A PROSPECTIVE OBSERVATIONAL STUDY	Gastroenterology	Records excluded after screening
874	Ruan, Z., Y. Zhang, Q. Quan, J. Jiang, Q. Wang, Y. Zhang and R. Peng	2022	Pan-cancer analysis identifies DDX56 as a prognostic biomarker associated with immune infiltration and drug sensitivity	Frontiers in Genetics	Records excluded after screening
875	Ruiz, C., J. Huang, S. F. Giardina, P. B. Feinberg, A. H. Mirza, M. D. Bacolod, S. A. Soper and F. Barany	2020	Single-molecule detection of cancer mutations using a novel PCR-LDR-qPCR assay	Human Mutation	Records excluded after screening

876	Ruiz-Barrios, L. D., T. D. Pineda-Razo, G. Hernández-Flores, P. C. Ortiz-Lazareno, A. Bravo-Cuellar, A. M. Macias-Lamas, K. J. Parra-Saavedra, L. A. Palafox-Mariscal, A. Aguilar-Lemarroy, L. F. Jave-Suárez and M. M. Villasenor-García	2024	Expression of ornithine decarboxylase in peripheral blood mononuclear cells from patients with pancreatic adenocarcinoma: A preliminary report	Biomedical Reports	Records excluded after screening
877	Rüstem, D. G., S. Atay, H. H. Aydin and H. Ak	2021	Synergistic Interactions between GW8510 and Gemcitabine in an In Vitro Model of Pancreatic Cancer	Anticancer Agents Med Chem	Records excluded after screening
878	Sadighbayan, D., K. Sadighbayan, A. Y. Khosroushahi and M. Hasanzadeh	2019	Recent advances on the DNA-based electrochemical biosensing of cancer biomarkers: Analytical approach	TrAC - Trends in Analytical Chemistry	Records excluded after screening
879	Sagami, R., K. Hayasaka, T. Ujihara, T. Iwaki, Y. Katsuyama, H. Harada, Y. Ome, G. Honda, S. I. Horiguchi, K. Murakami and Y. Amano	2023	Role of EUS combined with a newly modified scoring system to detect pancreatic high-grade precancerous lesions	Endoscopic Ultrasound	Records excluded after screening
880	Sagami, R., H. Nishikiori, K. Anami, S. Fujiwara, K. Honda, S. Ikuyama, M. Kitano and K. Murakami	2018	Utility of Endoscopic Ultrasonography Screening for Small Pancreatic Cancer and Proposal for a New Scoring System for Screening	Pancreas	Records excluded after screening
881	Sagami, R., T. Sato, K. Mizukami, M. Motomura, K. Okamoto, S. Fukuchi, Y. Otsuka, T. Abe, H. Ono, K. Mori, K. Wada, T. Iwaki, H. Nishikiori, K. Honda, Y. Amano and K. Murakami	2022	Diagnostic Strategy of Early Stage Pancreatic Cancer via Clinical Predictor Assessment: Clinical Indicators, Risk Factors and Imaging Findings	Diagnostics	No sample information provided
882	Sagredo, A. I., S. A. Sepulveda, J. C. Roa and L. Oróstica	2017	Exosomes in bile as potential pancreatobiliary tumor biomarkers	Translational Cancer Research	Records excluded after screening
883	Saito, R., K. Yoshimura, K. Shoda, S. Furuya, H. Akaike, Y. Kawaguchi, T. Murata, K. Ogata, T. Iwano, S. Takeda and D. Ichikawa	2021	Diagnostic significance of plasma lipid markers and machine learning-based algorithm for gastric cancer	Oncology Letters	Records excluded after screening
884	Sakai, A., M. Suzuki, T. Kobayashi, S. Nishiumi, T. Azuma and M. Yoshida	2016	Pancreatic cancer screening using a multiplatform human serum metabolomics system	Pancreatology	Records excluded after screening

885	Sakai, Y., M. Honda, S. Matsui, O. Komori, T. Murayama, T. Fujiwara, M. Mizuno, Y. Imai, K. Yoshimura, A. Nasti, T. Wada, N. Iida, M. Kitahara, R. Horii, T. Toshikatsu, M. Nishikawa, H. Okafuji, E. Mizukoshi, T. Yamashita, T. Yamashita, K. Arai, K. Kitamura, K. Kawaguchi, H. Takatori, T. Shimakami, T. Terashima, T. Hayashi, K. Nio and S. Kaneko	2019	Development of novel diagnostic system for pancreatic cancer, including early stages, measuring mRNA of whole blood cells	Cancer Science	Not early-stage
886	Sakhdari, A., E. F. Cosar and L. Hutchinson	2017	Sequential analysis of pancreatic cystic lesions generates a significantly higher diagnostic accuracy at molecular than cytologic levels	Laboratory Investigation	Records excluded after screening
887	Sammallahti, H., V. K. Sarhadi, A. Kokkola, R. Ghanbari, S. Rezasoltani, H. A. Aghdaei, P. Puolakkainen and S. Knuutila	2022	Oncogenomic Changes in Pancreatic Cancer and Their Detection in Stool	Biomolecules	Records excluded after screening
888	Sandanayake, N. S., F. Andreola, S. Camuzeaux, J. Sinclair, M. H. Chapman, G. J. Webster, A. Gentry-Maharaj, U. Menon, I. Jacobs, R. C. Smith and et al.	2010	Serum ceacam1 in the preclinical diagnosis of pancreatic adenocarcinoma	Gut	Records excluded after screening
889	Sankarasubramanian, S., G. Erdmann, P. Dudys, C. Pilarsky, C. Regenbrecht, C. Reinhard and L. Wedeken	2024	Development of a High-Throughput Assay for Functionally Profiling Individual Tumors to Direct Clinical Decision Making	Oncology Research and Treatment	Records excluded after screening
890	Sankarasubramanian, S., M. Niethard, C. Pilarsky, C. Regenbrecht and L. Wedeken	2022	Meeting Medical Need - Development of a High-Throughput Assay Using Pancreatic Cancer Patient Derived Organoids as a Tool for Personalized Medicine	Oncology Research and Treatment	Records excluded after screening
891	Sanna, V., S. Nurra, N. Pala, S. Marceddu, D. Pathania, N. Neamati and M. Sechi	2016	Targeted Nanoparticles for the Delivery of Novel Bioactive Molecules to Pancreatic Cancer Cells	J Med Chem	Records excluded after screening

892	Sansone, A., R. Lauretta, S. Vottari, A. Chiefari, A. Barnabei, F. Romanelli and M. Appetecchia	2019	Specific and non-specific biomarkers in neuroendocrine gastroenteropancreatic tumors	Cancers	Records excluded after screening
893	Santini, A. C., G. Giovane, A. Auletta, A. Di Carlo, A. Fiorelli, L. Cito, C. Astarita, A. Giordano, R. Alfano, A. Feola and M. Di Domenico	2016	Translational Research and Plasma Proteomic in Cancer	Journal of Cellular Biochemistry	Records excluded after screening
894	Sanz Pinazo, M. B., S. Martinez Martin, L. Martinez Carreras, L. D. Andres Garrido, M. P. Benayas Bellido and C. Avivar Oyonarte	2023	IMPORTANCE OF THE LABORATORY IN THE EARLY DIAGNOSIS OF PANCREATIC CYST PUNCTURE FLUID ANALYSIS	Clinical Chemistry and Laboratory Medicine	Records excluded after screening
895	Sarcina, L., F. Viola, F. Modena, R. A. Picca, P. Bollella, C. Di Franco, N. Cioffi, M. Caironi, R. Ö sterbacka, I. Esposito, G. Scamarcio, L. Torsi, F. Torricelli and E. Macchia	2022	A large-area organic transistor with 3D-printed sensing gate for noninvasive single-molecule detection of pancreatic mucinous cyst markers	Analytical and Bioanalytical Chemistry	Records excluded after screening
896	Sarr, A., J. Bré, I. H. Um, T. H. Chan, P. Mullen, D. J. Harrison and P. A. Reynolds	2019	Genome-scale CRISPR/Cas9 screen determines factors modulating sensitivity to ProTide NUC-1031	Scientific reports	Records excluded after screening
897	Sato, A., T. Masui, A. Yogo, T. Ito, K. Hirakawa, Y. Kanawaku, K. Koike and S. Uemoto	2020	Time-frequency analysis of serum with proton nuclear magnetic resonance for diagnosis of pancreatic cancer	Scientific Reports	Records excluded after screening
898	Sato, Y., T. Kobayashi, S. Nishiumi, A. Okada, T. Fujita, T. Sanuki, M. Kobayashi, M. Asahara, M. Adachi, A. Sakai, H. Shiomi, A. Masuda, M. Yoshida, K. Takeuchi, Y. Kodama, H. Kutsumi, K. Nagashima and K. Honda	2020	Prospective Study Using Plasma Apolipoprotein A2-Isoforms to Screen for High-Risk Status of Pancreatic Cancer	Cancers	Studies included in review
899	Sato, Y., R. Suzuki, T. Takagi, M. Sugimoto and H. Ohira	2020	- Circulating extracellular vesicle encapsulated microRNA as screening biomarkers for intraductal papillary mucinous neoplasm	Oncology Letters	Not early-stage

900	Sato, Y., H. Ueno, T. Ioka, S. Ohkawa, M. Ikeda, T. Shimamura, A. Tsuji, Y. Tsuchiya, J. Furuse, H. Ishii and et al.	2018	SLCO1B1 Polymorphism Is a Drug Response Predictive Marker for Advanced Pancreatic Cancer Patients Treated with Gemcitabine, S-1, or Gemcitabine plus S-1	Pancreas	Records excluded after screening
901	Savareh, B. A., H. A. Aghdaie, A. Behmanesh, A. Bashiri, A. Sadeghi, M. Zali and R. Shams	2020	A machine learning approach identified a diagnostic model for pancreatic cancer through using circulating microRNA signatures	Pancreatology	Records excluded after screening
902	Scarà, S., P. Bottoni and R. Scatena	2015	CA 19-9: Biochemical and Clinical Aspects. Advances in Cancer Biomarkers: From Biochemistry to Clinic for a Critical Revision. R. Scatena.		Records excluded after screening
903	Schilling, K., F. Larner, A. Saad, R. Roberts, H. M. Kocher, O. Blyuss, A. N. Halliday and T. Crnogorac-Jurcevic	2020	Urine metallomics signature as an indicator of pancreatic cancer	Metallomics	Records excluded after screening
904	Schmitz, D., D. Kazdal, M. Allgäuer, M. Trunk, S. Vornhusen, A. M. Nahm, M. Doll, S. Weingärtner, V. Endris, R. Penzel, M. Kirchner, R. Brandt, O. Neumann, H. Sü ltmann, J. Budczies, P. Kienle, R. Magdeburg, S. Hetjens, P. Schirmacher, F. Bergmann, J. Rudi, A. Stenzinger and A. L. Volckmar	2021	KRAS/GNAS-testing by highly sensitive deep targeted next generation sequencing improves the endoscopic ultrasound-guided workup of suspected mucinous neoplasms of the pancreas	Genes Chromosomes and Cancer	Small sample size
905	Schönemeier, B., J. Metzger, J. Klein, H. Husi, B. Bremer, N. Armbrecht, M. Dakna, J. P. Schanstra, J. Rosendahl, J. Wiegand, M. Jäger, W. Mullen, B. Breuil, R. R. Plentz, R. Lichtinghagen, K. Brand, F. Kühnel, H. Mischak, M. P. Manns and T. O. Lankisch	2016	Urinary Peptide Analysis Differentiates Pancreatic Cancer From Chronic Pancreatitis	Pancreas	Not early-stage

906	Schultz, N. A., C. Dehlendorff, B. V. Jensen, J. K. Bjerregaard, K. R. Nielsen, S. E. Bojesen, D. Calatayud, S. E. Nielsen, M. Yilmaz, N. H. Holländer, K. K. Andersen and J. S. Johansen	2014	MicroRNA Biomarkers in Whole Blood for Detection of Pancreatic Cancer	Jama-Journal of the American Medical Association	Studies included in review
907	Schwartz, A., H. Rincon, N. Hansen, R. Lawrence, S. Anderson, N. Blesie, K. Klussman, A. Epp, S. Gardai and W. Arthur	2021	Targeting Sialyl-Thomsen nouveau (STn) antigen with the SGN-STNV antibody-drug conjugate is effective in preclinical studies	Cancer Research	Records excluded after screening
908	Sculco, M., M. La Vecchia, A. Aspesi, M. G. Clavenna, M. Salvo, G. Borgonovi, A. Pittaro, G. Witel, F. Napoli, A. Listì, F. Grosso, R. Libener, A. Maconi, O. Rena, R. Boldorini, D. Giachino, P. Bironzo, A. Maffè, G. Alì, L. Elefanti, C. Menin, L. Righi, C. Tampieri, G. V. Scagliotti, C. Dianzani, D. Ferrante, E. Migliore, C. Magnani, D. Mirabelli, G. Matullo and I. Dianzani	2022	Diagnostics of BAP1-Tumor Predisposition Syndrome by a Multitesting Approach: A Ten-Year-Long Experience	Diagnostics	Records excluded after screening
909	Sears, R., J. Hinestrosa, G. Schroeder, J. Lewis, H. Balcer, R. Kurzrock, S. Lippman, A. Lowy and P. Billings	2022	1306P Early-stage pancreatic cancer detection using extracellular vesicles	Annals of Oncology	Records excluded after screening
910	Sefrioui, D., F. Blanchard, P. Basile, E. Toure, C. Dolfus, L. Beaussire, N. Vasseur, A. Perdrix, A. Gangloff, L. Schwarz, F. Clatot, J. J. Tuech, J. C. Sabourin, T. Frebourg, P. Michel and F. Di Fiore	2016	Diagnostic performance of liquid biopsy for pancreatic solid lesion as alternative to endoscopic ultrasound-guided fine needle aspiration (EUS-FNA)	Annals of Oncology	Records excluded after screening
911	Sellahewa, R., J. Lundy, D. Croagh and B. Jenkins	2021	High circulating tumour DNA is a strong negative prognostic factor in operable pancreatic cancer	HPB	Records excluded after screening
912	Seo, Y., S. S. Kim, N. Kim, S. Cho, J. B. Park and J. H. Kim	2020	Development of a miRNA-controlled dual-sensing system and its application for targeting miR-21 signaling in tumorigenesis	Experimental and Molecular Medicine	Records excluded after screening

913	Seppälä, T. T., J. W. Zimmerman, E. Sereni, D. Plenker, R. Suri, N. Rozich, A. Blair, D. L. Thomas, J. Teinor, A. Javed, H. Patel, J. L. Cameron, W. R. Burns, J. He, D. A. Tuveson, E. M. Jaffee, J. Eshleman, A. Szabolcs, D. P. Ryan, D. T. Ting, C. L. Wolfgang and R. A. Burkhart	2020	Patient-derived Organoid Pharmacotyping is a Clinically Tractable Strategy for Precision Medicine in Pancreatic Cancer	Annals of Surgery	Records excluded after screening
914	Seppala, T. T., J. W. Zimmerman, R. Suri, H. Zlomke, G. D. Ivey, A. Szabolcs, C. R. Shubert, J. L. Cameron, W. R. Burns, K. J. Lafaro and et al.	2022	Precision Medicine in Pancreatic Cancer: patient-Derived Organoid Pharmacotyping Is a Predictive Biomarker of Clinical Treatment Response	Clinical cancer research	Records excluded after screening
915	Serrano, M. J., M. C. Garrido-Navas, J. J. D. Mochon, M. Cristofanilli, I. Gil-Bazo, P. Pauwels, U. Malapelle, A. Russo, J. A. Lorente, A. J. Ruiz-Rodriguez, L. G. Paz-Ares, E. Vilar, L. E. Raez, A. F. Cardona and C. Rolfo	2020	Precision prevention and cancer interception: The new challenges of liquid biopsy	Cancer Discovery	Records excluded after screening
916	Seth, S., C. Y. Li, I. L. Ho, D. Corti, S. Loponte, L. Sapio, E. Del Poggetto, E. Y. Yen, F. S. Robinson, M. Peoples, T. Karpinets, A. K. Deem, T. Kumar, X. Song, S. Jiang, Y. Kang, J. Fleming, M. Kim, J. Zhang, A. Maitra, T. P. Heffernan, V. Giuliani, G. Genovese, A. Futreal, G. F. Draetta, A. Carugo and A. Viale	2019	Pre-existing Functional Heterogeneity of Tumorigenic Compartment as the Origin of Chemoresistance in Pancreatic Tumors	Cell Reports	Records excluded after screening
917	Seyed Salehi, A., N. Parsa-Nikoo, F. Roshan-Farzad, R. Shams, M. Fathi, H. Asaszadeh Aghdaei and A. Behmanesh	2022	MicroRNA-125a-3p, -4530, and -92a as a Potential Circulating MicroRNA Panel for Noninvasive Pancreatic Cancer Diagnosis	Disease Markers	No sample information provided
918	Seyhan, A. A.	2023	Circulating microRNAs as Potential Biomarkers in Pancreatic Cancer—Advances and Challenges	International Journal of Molecular Sciences	Records excluded after screening

919	Sha, M., B. Kunduzi and S. Froghi	2021	P-25 A review of circulating exosomal biomarkers in the diagnosis of pancreatic cancer	Annals of Oncology	Records excluded after screening
920	Sha, M., B. Kunduzi, S. Froghi, A. Quaglia, B. Davidson and G. K. Fusai	2023	Role of circulating exosomal biomarkers and their diagnostic accuracy in pancreatic cancer	Jgh Open	No sample information provided
921	Shah, I., V. Wadhwa, M. Bilal, K. A. Germansky and M. S. Sawhney	2021	Prospective Assessment for Prediabetes and New-Onset Diabetes in High-Risk Individuals Undergoing Pancreatic Cancer Screening	Gastroenterology	Records excluded after screening
922	Shah, N., R. Podury, E. Kikano, D. A. Smith, N. H. Ramaiya and S. H. Tirumani	2020	Utilization of imaging in pancreatic adenocarcinoma patients status post Whipple procedure	Journal of Clinical Oncology	Records excluded after screening
923	Shams, R., S. Saberi, M. Zali, A. Sadeghi, S. Ghafouri-Fard and H. A. Aghdaei	2020	Identification of potential microRNA panels for pancreatic cancer diagnosis using microarray datasets and bioinformatics methods	Scientific Reports	Records excluded after screening
924	Shang, M., M. Ma, G. Su and L. Xiao	2023	Application value of miRNA-182 as a biomarker for cancer diagnosis: a systematic review with meta-analysis	Biomarkers in Medicine	Records excluded after screening
925	Sharma, A., H. Kandlakunta, S. J. S. Nagpal, Z. Feng, W. Hoos, G. M. Petersen and S. T. Chari	2018	Model to Determine Risk of Pancreatic Cancer in Patients With New-Onset Diabetes	Gastroenterology	Records excluded after screening
926	Sharma, A. K., S. Singh, H. Singh, D. Mahajan, P. Kolli, G. Mandadapu, B. Kumar, D. Kumar, S. Kumar and M. K. Jena	2022	Deep Insight of the Pathophysiology of Gestational Diabetes Mellitus	Cells	Records excluded after screening
927	Sharma, N., A. Perelman, A. Sharma, C. Linke, C. Zelt and S. Gupta	2018	Nonfunctional pancreatic neuroendocrine tumors: A retrospective review and early detection facilitated by EUS and novel core biopsy techniques	Pancreas	Records excluded after screening
928	Shen, C. Q., Y. Li, Y. M. Li, S. J. Wang, Y. Y. Li, F. Tang, P. Wang, H. Liu, Y. Y. Li and Q. Liu	2022	A double reaction system induced electrochemiluminescence enhancement based on SnS2 QDs@MIL-101 for ultrasensitive detection of CA242	Talanta	Records excluded after screening

929	Shen, S. Y., R. Singhania, G. Fehringer, A. Chakravarthy, M. H. A. Roehrl, D. Chadwick, P. C. Zuzarte, A. Borgida, T. T. Wang, T. Li, O. Kis, Z. Zhao, A. Spreafico, T. D. S. Medina, Y. Wang, D. Roulois, I. Ettayebi, Z. Chen, S. Chow, T. Murphy, A. Arruda, G. M. O'Kane, J. Liu, M. Mansour, J. D. McPherson, C. O'Brien, N. Leighl, P. L. Bedard, N. Fleshner, G. Liu, M. D. Minden, S. Gallinger, A. Goldenberg, T. J. Pugh, M. M. Hoffman, S. V. Bratman, R. J. Hung and D. D. De Carvalho	2018	Sensitive tumour detection and classification using plasma cell-free DNA methylomes	Nature	Records excluded after screening
930	Shen, Y., Y. Pan, L. Xu, L. Chen, L. Liu, H. Chen, Z. Chen and Z. Meng	2015	Identifying microRNA-mRNA regulatory network in gemcitabine-resistant cells derived from human pancreatic cancer cells	Tumor Biology	Records excluded after screening
931	Shen, Y., X. Zhu, L. Xu, Y. Pan, Z. Meng, Z. Chen and L. Liu	2014	Identification of microRNA-mRNA regulatory network in gemcitabine resistant cells derived from human pancreatic cancer cells	Asia-Pacific Journal of Clinical Oncology	Records excluded after screening
932	Shi, W., T. Wartmann, S. Accuffi, S. Al-Madhi, A. Perrakis, C. Kahlert, A. Link, M. Venerito, V. Keitel-Anselmino, C. Bruns, R. S. Croner, Y. Zhao and U. D. Kahlert	2024	Integrating a microRNA signature as a liquid biopsy-based tool for the early diagnosis and prediction of potential therapeutic targets in pancreatic cancer	British Journal of Cancer	Non-diagnostic studies
933	Shi, X., S. Guo, Q. Duan, W. Zhang, S. Gao, W. Jing, G. Jiang, X. Kong, P. Li, Y. Li, C. Teng, X. Xu, S. Chen, B. Nian, Z. Li, C. Zhong, X. Yang, G. Zhu, Y. Du, D. Zhang and G. Jin	2024	Detection and characterization of pancreatic and biliary tract cancers using cell-free DNA fragmentomics	Journal of Experimental and Clinical Cancer Research	No sample information provided
934	Shimizu, T., M. Asakuma, A. Tomioka, Y. Inoue, F. Hirokawa, M. Hayashi and K. Uchiyama	2018	Span-1 and CA19-9 as Predictors of Early Recurrence and Lymph Node Metastasis for Patients with Invasive Pancreatic Cancer after Pancreatectomy	Am Surg	Records excluded after screening

935	Shinjo, K., K. Hara, G. Nagae, T. Umeda, K. Katsushima, M. Suzuki, Y. Murofushi, Y. Umezu, I. Takeuchi, S. Takahashi, Y. Okuno, K. Matsuo, H. Ito, S. Tajima, H. Aburatani, K. Yamao and Y. Kondo	2020	A novel sensitive detection method for DNA methylation in circulating free DNA of pancreatic cancer	PLoS One	Records excluded after screening
936	Shinjo, K. and Y. Kondo	2016	Use of DNA methylation in circulating free DNA as a potent biomarker for pancreatic cancer detection	Cancer Research	Records excluded after screening
937	Shinjo, K., F. Ohka, K. Katsushima, A. Hatanaka, N. Ichimura, Z. Juan, K. Yamao and Y. Kondo	2015	DNA methylation in circulating free DNA as a new biomarker for pancreatic cancer	Cancer Research	Records excluded after screening
938	Shinozaki, E., K. Tanabe, T. Akiyoshi, T. Tsuchida, Y. Miyazaki, N. Kojima, M. Igarashi, M. Ueno, M. Suenaga, N. Mizunuma, K. Yamaguchi, K. Nakayama, S. Iijima and T. Yamaguchi	2018	Serum leucine-rich alpha-2-glycoprotein-1 with fucosylated triantennary N-glycan: a novel colorectal cancer marker	Bmc Cancer	Records excluded after screening
939	Shoji, H., N. Miura, H. Ueno and K. Honda	2018	Measurement of copy number of ACTN4 to optimize the therapeutic strategy for locally advanced pancreatic cancer	Pancreatology	Records excluded after screening
940	Siddappa, P. K., S. T. Chari, F. C. Gleeson, T. Malikowski, B. K. A. Dayyeh, V. Chandrasekhara, P. G. Iyer, S. Majumder, R. K. Pearson, B. T. Petersen, E. Rajan, A. C. Storm, M. Topazian, S. S. Vege, K. K. Wang and M. J. Levy	2019	THE UTILITY OF CA 19-9 IN PATIENTS UNDERGOING EUS FOR PANCREATIC DUCTAL ADENOCARCINOMA: SINGLE CENTER EXPERIENCE OVER 2 DECADES	Gastroenterology	Records excluded after screening
941	Sijithra, P. C., N. Santhi and N. Ramasamy	2023	A review study on early detection of pancreatic ductal adenocarcinoma using artificial intelligence assisted diagnostic methods	European Journal of Radiology	Records excluded after screening
942	Sikora, K., C. Bedin, C. Vicentini, G. Malpeli, E. D'Angelo, N. Sperandio, R. T. Lawlor, C. Bassi, G. Tortora, D. Nitti, M. Agostini, M. Fassan and A. Scarpa	2015	Evaluation of cell-free DNA as a biomarker for pancreatic malignancies	Int J Biol Markers	Not early-stage

943	Simpson, R. E., N. J. Cockerill, M. T. Yip-Schneider, E. P. Ceppa, M. G. House, N. J. Zyromski, A. Nakeeb, M. A. Al-Haddad and C. M. Schmidt	2019	Clinical criteria for integrated molecular pathology in intraductal papillary mucinous neoplasm: less is more	HPB	Records excluded after screening
944	Simpson, R. E., M. T. Yip-Schneider, K. F. Flick, H. Wu, C. L. Colgate and C. M. Schmidt	2019	Pancreatic fluid interleukin-1β complements prostaglandin E2 and serum carbohydrate antigen 19-9 in prediction of intraductal papillary mucinous neoplasm dysplasia	Pancreas	Records excluded after screening
945	Simpson, R. E., M. T. Yip-Schneider, H. Wu, H. Fan, Z. Liu, M. Korc, J. Zhang and C. M. Schmidt	2019	Circulating Thrombospondin-2 enhances prediction of malignant intraductal papillary mucinous neoplasm	Am J Surg	Records excluded after screening
946	Singhi, A. D., E. J. Koay, S. T. Chari and A. Maitra	2019	Early Detection of Pancreatic Cancer: Opportunities and Challenges	Gastroenterology	Records excluded after screening
947	Singhi, A. D., M. N. Nikiforova, J. Chennat, G. I. Papachristou, A. Khalid, M. Rabinovitz, R. Das, S. Sarkaria, M. S. Ayasso, A. I. Wald, S. E. Monaco, M. Nalesnik, N. P. Ohori, D. Geller, A. Tsung, A. H. Zureikat, H. Zeh, J. W. Marsh, M. Hogg, K. Lee, D. L. Bartlett, J. F. Pingpank, A. Humar, N. Bahary, A. K. Dasyam, R. Brand, K. E. Fasanella, K. McGrath and A. Slivka	2020	Integrating next-generation sequencing to endoscopic retrograde cholangiopancreatography (ERCP)-obtained biliary specimens improves the detection and management of patients with malignant bile duct strictures	Gut	Non-diagnostic studies
948	Singhi, A. D. and L. D. Wood	2021	Early detection of pancreatic cancer using DNA-based molecular approaches	Nature Reviews Gastroenterology and Hepatology	Non-diagnostic studies
949	Škrha, P., A. Hořínek, M. Anděl, P. Frič and J. Škrha	2016	New-onset diabetes as a target for pancreatic cancer screening	Diabetologia	Records excluded after screening
950	Škrha, P., A. Hořínek, M. Anděl, P. Frič and J. Škrha	2020	CA 19-9, miR200 and GIP in patients with diabetes and pancreatic cancer	Diabetologia	Records excluded after screening
951	Skrha, P., A. Horinek and J. Skrha	2020	CA19-9, MIR200, and GIP in patients with diabetes mellitus and pancreatic cancer	Diabetes	Records excluded after screening
952	Skripova, V., R. Vlasenkova, I. Astsaturov and R. Kiyamova	2020	61P CRISPR/Cas9 and bioinformatics approaches revealed new regulators of pancreatic cancer cells platinum sensitivity	Annals of Oncology	Records excluded after screening

953	Skripova, V., R. Vlasenkova, Y. Zhou, I. Astsaturov and R. Kiyamova	2022	Identification of New Regulators of Pancreatic Cancer Cell Sensitivity to Oxaliplatin and Cisplatin	Molecules	Records excluded after screening
954	Skulimowski, A., A. Durczyński, J. Strzelczyk and P. Hogendorf	2021	Comparison of clinical usefulness of serum Ca125 and CA19-9 in pancreatic adenocarcinoma diagnosis: meta-analysis and systematic review of literature	Biomarkers	Records excluded after screening
955	Slater, E. P., K. Strauch, S. Rospleszcz, A. Ramaswamy, I. Esposito, G. Klöppel, E. Matthäi, K. Heeger, V. Fendrich, P. Langer and D. K. Bartsch	2014	MicroRNA-196a and-196b as Potential Biomarkers for the Early Detection of Familial Pancreatic Cancer	Translational Oncology	Records excluded after screening
956	Slotwinski, R. and S. M. Slotwinska	2016	Diagnostic value of selected markers and apoptotic pathways for pancreatic cancer	Central European Journal of Immunology	Records excluded after screening
957	Smith, L. A. and C. J. McKay	2016	The role of endoscopic ultrasound in the management of intraductal papillary mucinous neoplasms: A systematic update	Minerva Medica	Records excluded after screening
958	Snyder, N. W., C. Mesaros and I. A. Blair	2015	Translational metabolomics in cancer research	Biomarkers in Medicine	Records excluded after screening
959	Sogawa, K., S. Takano, F. Iida, M. Satoh, S. Tsuchida, Y. Kawashima, H. Yoshitomi, A. Sanda, Y. Kodera, H. Takizawa, R. Mikata, M. Ohtsuka, H. Shimizu, M. Miyazaki, O. Yokosuka and F. Nomura	2016	Identification of a novel serum biomarker for pancreatic cancer, C4b-binding protein α-chain (C4BPA) by quantitative proteomic analysis using tandem mass tags	British Journal of Cancer	Records excluded after screening
960	Sokolenko, A. P. and E. N. Imyanitov	2017	Molecular Tests for the Choice of Cancer Therapy	Current Pharmaceutical Design	Records excluded after screening
961	Solaiyappan, M., S. Kumar Bharti, P. T. Winnard, M. Dbouk, M. G. Goggins and Z. M. Bhujwalla	2020	Detecting pancreatic cancer through neural network analysis of human plasma MR spectra	Cancer Research	Records excluded after screening
962	Solanki, A., D. King, G. Thibault, L. Wang and S. L. Gibbs	2020	Quantification of fluorophore distribution and therapeutic response in matched in vivo and ex vivo pancreatic cancer model systems	PLoS ONE	Records excluded after screening
963	Song, B., D. Huang, Y. Zhang, Z. Wei, J. Su, J. Pedro de Magalhães, D. J. Rigden, J. Meng and K. Chen	2023	m6A-TSHub: Unveiling the Context-specific m6A Methylation and m6A-affecting Mutations in 23 Human Tissues	Genomics, Proteomics and Bioinformatics	Records excluded after screening

964	Song, J., D. M. Bach, J. J. Pasay, A. L. Rubin, L. J. Sokoll, D. W. Chan and Z. Zhang	2015	Development of magnetic bead-based multiplex immunoassays to evaluate serum biomarkers for the early detection of pancreatic cancer	Clinical Chemistry	Records excluded after screening
965	Song, J., L. J. Sokoll, D. W. Chan and Z. Zhang	2021	Validation of Serum Biomarkers That Complement CA19-9 in Detecting Early Pancreatic Cancer Using Electrochemiluminescent-Based Multiplex Immunoassays	Biomedicines	Not early-stage
966	Song, J., L. J. Sokoll, J. J. Pasay, A. L. Rubin, H. Li, D. M. Bach, D. W. Chan and Z. Zhang	2019	Identification of serum biomarker panels for the early detection of pancreatic cancer	Cancer Epidemiology Biomarkers and Prevention	Incomplete diagnostic performance data provided
967	Song, W., L. Wu and H. Zhao	2017	Clinical value of peripheral blood CA19-9, CA242 and CEA levels for the diagnosis and pathologic staging of pancreatic cancer	Journal of Practical Oncology	Records excluded after screening
968	Søreide, K., W. Ismail, M. Roalsø, J. Ghotbi and C. Zaharia	2023	Early Diagnosis of Pancreatic Cancer: Clinical Premonitions, Timely Precursor Detection and Increased Curative-Intent Surgery	Cancer Control	Records excluded after screening
969	Souche, R., G. Tosato, B. Rivière, J. C. Valats, A. Debourdeau, N. Flori, D. Pourquier, J. M. Fabre, E. Assenat, J. Colinge and A. Turtoi	2022	Detection of soluble biomarkers of pancreatic cancer in endoscopic ultrasound-guided fine-needle aspiration samples	Endoscopy	Records excluded after screening
970	Spadaccini, M., G. Koleth, J. Emmanuel, K. Khalaf, A. Facciorusso, F. Grizzi, C. Hassan, M. Colombo, B. Mangiavillano, A. Fugazza, A. Anderloni, S. Carrara and A. Repici	2022	Enhanced endoscopic ultrasound imaging for pancreatic lesions: The road to artificial intelligence	World Journal of Gastroenterology	Records excluded after screening
971	Srivastava, S. and S. Hanash	2020	Pan-Cancer Early Detection: Hype or Hope?	Cancer Cell	Records excluded after screening
972	Stahel, P. F.	2014	Commentary on "investigational biomarkers for pancreatic adenocarcinoma: Where do we stand?"	Southern Medical Journal	Records excluded after screening
973	Stasiak, M., B. Wlodarczyk, P. Wlodarczyk, A. Borkowska and E. I. Malecka-Wojciesko	2024	THE DIAGNOSTIC AND PROGNOSTIC VALUE OF APELIN AND SERUM AMYLOID A3 MEASUREMENT IN PATIENTS WITH PANCREATIC DUCTAL ADENOCARCINOMA (PDAC) ANDCHRONIC PANCREATITIS (CP)	Gastroenterology	Records excluded after screening
974	Stoffel, E. M.	2015	Screening in GI Cancers: The Role of Genetics	J Clin Oncol	Records excluded after screening

975	Stoffel, E. M., S. E. McKernin, R. Brand, M. Canto, M. Goggins, C. Moravek, A. Nagarajan, G. M. Petersen, D. M. Simeone, M. Yurgelun and A. A. Khorana	2019	Evaluating Susceptibility to Pancreatic Cancer: ASCO Provisional Clinical Opinion	J Clin Oncol	Records excluded after screening
976	Stosic, K., O. A. Senar, J. Tarfouss, C. Bouchart, J. Navez, J. L. Van Laethem and T. Arsenijevic	2024	A Comprehensive Review of the Potential Role of Liquid Biopsy as a Diagnostic, Prognostic, and Predictive Biomarker in Pancreatic Ductal Adenocarcinoma	Cells	Records excluded after screening
977	Su, F., Z. Gao, Y. Liu, G. Zhou, W. Gao, C. Deng, Y. Liu, Y. Zhang, X. Ma, Y. Wang, L. Guan, Y. Zhang and B. Liu	2022	Prioritizing key synergistic circulating microRNAs for the early diagnosis of biliary tract cancer	Frontiers in Oncology	Records excluded after screening
978	Suehiro, Y., S. Suenaga, Y. Kunimune, S. Yada, K. Hamamoto, T. Tsuyama, S. Amano, H. Matsui, S. Higaki, I. Fujii, C. Suzuki, T. Hoshida, T. Matsumoto, Y. Fujimoto, S. Kaino, K. Shinjo, Y. Kondo, I. Sakaida, T. Takami, H. Nagano and T. Yamasaki	2022	CA19-9 in Combination with Methylated HOXA1 and SST Is Useful to Diagnose Stage I Pancreatic Cancer	Oncology	Records excluded after screening
979	Sugimori, M., K. Sugimori, C. Jimbo, A. Hirotani, K. Sanga, T. Sato, S. Tezuka, Y. Goda, K. Irie, H. Miwa, W. Shibata, A. Nozaki and S. Maeda	2018	Practice of genome diagnosis in pancreatic tumor	Cancer Science	Records excluded after screening
980	Sugimoto, M.	2020	Salivary metabolomics for cancer detection	Expert Review of Proteomics	Records excluded after screening
981	Suh, H. N., S. Jun, A. Y. Oh, M. Srivastava, S. Lee, C. M. Taniguchi, S. Zhang, W. S. Lee, J. Chen, B. J. Park and J. I. Park	2016	Identification of KIAA1199 as a Biomarker for Pancreatic Intraepithelial Neoplasia	Scientific reports	Records excluded after screening
982	Sun, D., H. Jin, J. Zhang and X. Tan	2018	Integrated whole genome microarray analysis and immunohistochemical assay identifies COL11A1, GJB2 and CTRL as predictive biomarkers for pancreatic cancer	Cancer Cell International	Not early-stage
983	Sun, J., M. Su, J. Ma, M. Xu, C. Ma, W. Li, R. Liu, Q. He and Z. Su	2023	Cross-platform comparisons for targeted bisulfite sequencing of MGISEQ2000 and NovaSeq6000	Clinical Epigenetics	Records excluded after screening

984	Sun, L. Q., L. S. Peng, J. F. Guo, F. Jiang, F. Cui, H. J. Huang and Z. D. Jin	2021	Validation of serum tumor biomarkers in predicting advanced cystic mucinous neoplasm of the pancreas	World Journal of Gastroenterology	Not early-stage
985	Sun, X., X. Zhou, Y. Zhang, X. Zhu and H. Liu	2018	Systematic Review and Meta-Analysis of Diagnostic Accuracy of miRNAs in Patients with Pancreatic Cancer	Dis Markers	Records excluded after screening
986	Sunamura, M., T. Itoi, J. Umeda, J. Itakura, M. Toki, A. Tuchida, M. Shimazu, S. Kawachi and M. Sugimoto	2016	Salivary bio-markers for the early detection of pancreatic cancer	Pancreatology	Records excluded after screening
987	Suo, C., R. Zhao, Y. Jiang, Y. Zhang, Q. He, Z. Su, R. Liu, L. Jin and X. Chen	2023	The FuSion Project of Pan-Cancer Early Screening in Chinese- An integrative study by Fudan University and Singlera	Cancer Research	Records excluded after screening
988	Suresh, V., K. Byers, U. C. Rajesh, F. Caiazza, G. Zhu, C. S. Craik, K. Kirkwood, V. J. Davisson and D. A. Sheik	2022	Translation of a Protease Turnover Assay for Clinical Discrimination of Mucinous Pancreatic Cysts	Diagnostics	No sample information provided
989	Suzuki, M., S. Nishiumi, T. Kobayashi, A. Sakai, Y. Iwata, T. Uchikata, Y. Izumi, T. Azuma, T. Bamba and M. Yoshida	2017	Use of on-line supercritical fluid extraction-supercritical fluid chromatography/tandem mass spectrometry to analyze disease biomarkers in dried serum spots compared with serum analysis using liquid chromatography/tandem mass spectrometry	Rapid Communications in Mass Spectrometry	Records excluded after screening
990	Suzuki, R., H. Tamura, R. Honma, N. Konno, H. Irie, T. Takagi, M. Sugimot, H. Asama, Y. Sato, O. Yoshinori, J. Nakamura, M. Taicasumi, T. Kato, M. Hashimoto, T. Hikichi, J. I. Imai, S. Watanabe and H. Ohira	2020	A Blood-based Gene-expression Scoring System for Cancer Screening in Patients With Branch-duct Intraductal Papillary Mucinous Neoplasms	Anticancer Research	Non-diagnostic studies
991	Swords, D. S., M. A. Firpo, C. L. Scaife and S. J. Mulvihill	2016	Biomarkers in pancreatic adenocarcinoma: Current perspectives	OncoTargets and Therapy	Records excluded after screening
992	Taguchi, A., M. Capello, Y. Zhao, I. Babel, G. Goodman, M. A. Tempero, M. A. Firpo, M. H. Katz, Z. Feng and S. Hanash	2015	Development and validation of diagnostic biomarker model for detection of early stage pancreatic cancer	Cancer Research	Records excluded after screening
993	Taguchi, M., R. Bouchi, T. Fukuda, N. Ihana-Sugiyama, N. Kodani, M. Ohsugi, A. Tanabe, K. Ueki and H. Kajio	2023	Clinical significance of tumor markers in patients with type 2 diabetes: a retrospective observational study	Diabetology International	Records excluded after screening

994	Takahashi, R., T. Ishizawa, M. Sato, Y. Inagaki, M. Takanka, Y. Kuriki, M. Kamiya, T. Ushiku, Y. Urano and K. Hasegawa	2021	Fluorescence Imaging Using Enzyme-Activatable Probes for Real-Time Identification of Pancreatic Cancer	Frontiers in Oncology	Records excluded after screening
995	Takayanagi, D., H. Cho, E. Machida, A. Kawamura, A. Takashima, S. Wada, T. Tsunoda, T. Kohno and K. Shiraishi	2022	Update on Epidemiology, Diagnosis, and Biomarkers in Gastroenteropancreatic Neuroendocrine Neoplasms	Cancers	Records excluded after screening
996	Tamm, E. P.	2014	Imaging of Pancreatic Neoplasms	Surgical Oncology Clinics of North America	Records excluded after screening
997	Tan, D., Y. S. Ho, L. Y. Yip, K. H. Lim, C. Y. Chan, C. P. Chung, Y. Fui, A. Chung, B. K. Goh, S. Y. Lee, L. L. Ooi, P. K. Chow, D. Tai, C. Y. Ka, S. Mesenas, W. C. Ong, Z. Wang, C. Wu and C. J. L. Khor	2019	TRIACYLGLYCEROLS AS A MARKER FOR CHARACTERIZATION AND EARLY DETECTION OF MALIGNANCY IN PANCREATIC CYSTS	Gastroenterology	Records excluded after screening
998	Tang, C., P. Corredeira, S. Casimiro, W. Sukdao, L. Costa, E. Yates and G. Bernardes	2024	Immune activation characterization via amino acid concentration signatures for multi-cancer early detection and CDKi treatment response prediction	Cancer Research	Records excluded after screening
999	Tang, F., Y. Liu, Y. Sun, Y. Xiong, Y. Gu, J. Zhou, Y. Ouyang and S. Zhang	2023	Construction of a serum diagnostic signature based on m5C-related miRNAs for cancer detection	Frontiers in Endocrinology	Records excluded after screening
1000	Tang, H., P. Hsueh, D. Kletter, M. Bern and B. Haab	2015	The detection and discovery of glycan motifs in biological samples using lectins and antibodies: New methods and opportunities.		Records excluded after screening
1001	Tang, H., S. Singh, K. Partyka, D. Kletter, P. Hsueh, J. Yadav, E. Ensink, M. Bern, G. Hostetter, D. Hartman, Y. Huang, R. E. Brand and B. B. Haab	2015	Glycan motif profiling reveals plasma sialyl-Lewis X elevations in pancreatic cancers that are negative for sialyl-Lewis A	Molecular and Cellular Proteomics	No sample information provided
1002	Tang, Y. T., X. H. Xu, X. D. Yang, J. Hao, H. Cao, W. Zhu, S. Y. Zhang and J. P. Cao	2014	Role of non-coding RNAs in pancreatic cancer: the bane of the microworld	World J Gastroenterol	Records excluded after screening
1003	Taniuchi, K.	2016	A tumor-associated mRNA localizing in circulating exosomes as a novel serological marker for pancreatic cancer: the retrospective clinical study	Cancer Research	Records excluded after screening
1004	Taniuchi, K., M. Tsuboi, T. Kohsaki, S. Iwasaki and T. Saibara	2019	CCDC88A mRNA localizing in circulating tumorderived exosomes as a novel serological marker for pancreatic cancer	Tumor Biology	Records excluded after screening

1005	Tanţău, A., D. C. Leucuţa, M. Tanţău, E. Boţan, R. Zaharie, A. Mândruţiu and I. C. Tomuleasa	2021	Inflammation, Tumoral Markers and Interleukin-17, -10, and -6 Profiles in Pancreatic Adenocarcinoma and Chronic Pancreatitis	Digestive Diseases and Sciences	Not early-stage
1006	Tarasiuk, A., T. Mackiewicz, E. Małecka-Panas and J. Fichna	2021	Biomarkers for early detection of pancreatic cancer–miRNAs as a potential diagnostic and therapeutic tool?	Cancer Biology and Therapy	Records excluded after screening
1007	Tenchov, R., A. K. Sapra, J. Sasso, K. Ralhan, A. Tummala, N. Azoulay and Q. A. Zhou	2024	Biomarkers for Early Cancer Detection: A Landscape View of Recent Advancements, Spotlighting Pancreatic and Liver Cancers	ACS Pharmacology and Translational Science	Records excluded after screening
1008	The Lancet, G. and Hepatology	2019	Pancreatic cancer screening: more harms than benefits?	The Lancet Gastroenterology and Hepatology	Records excluded after screening
1009	Thierry, A. R.	2023	Circulating DNA fragmentomics and cancer screening	Cell Genomics	Records excluded after screening
1010	Thyparambil, S. P., W. L. Liao, R. Heaton, A. Strasbaugh, M. A. Melkie and X. Ling	2023	Proteomic profiling of antibody-drug conjugate (ADC) biomarkers in pancreatic cancer	Journal of Clinical Oncology	Records excluded after screening
1011	Tidd-Johnson, A., S. A. Sebastian, E. L. Co, M. Afaq, H. Kochhar, M. Sheikh, A. Mago, S. Poudel, J. A. Fernandez, I. D. Rodriguez and S. Razdan	2022	Prostate cancer screening: Continued controversies and novel biomarker advancements	Current Urology	Records excluded after screening
1012	Tonini, V. and M. Zanni	2021	Pancreatic cancer in 2021: What you need to know to win	World Journal of Gastroenterology	Records excluded after screening
1013	Torres, J. B., M. Mosley, S. Koustoulidou, S. Hopkins, S. Knapp, A. Chaikuad, M. Kondoh, K. Tachibana, V. Kersemans and B. Cornelissen	2020	Radiolabeled cCPE peptides for SPECT imaging of claudin-4 overexpression in pancreatic cancer	Journal of Nuclear Medicine	Records excluded after screening
1014	Tosato, G., F. R. Souche, B. Riviere, J. M. Fabre, D. Pourqier, E. Assenat, J. Colinge and A. Turtoi	2021	Discovery of Soluble Pancreatic Cancer Biomarkers Using Innovative Clinical Proteomics and Statistical Learning	Pancreas	Records excluded after screening
1015	Traeger, M. M., J. Rehkaemper, H. Ullerich, K. Steinestel, E. Wardelmann, N. Senninger and S. A. Dhayat	2018	The ambiguous role of microRNA205 and its clinical potential in pancreatic ductal adenocarcinoma	Journal of Cancer Research and Clinical Oncology	Records excluded after screening
1016	Trajtenberg, C., D. Roife, Y. Kang, K. Lundberg, E. Yohannes and J. Fleming	2017	Proteomic analysis of saliva in patients with pancreatic adenocarcinoma	Annals of Surgical Oncology	Records excluded after screening

1017	Traub, B., K. H. Link and M. Kornmann	2021	Curing pancreatic cancer	Seminars in Cancer Biology	Records excluded after screening
1018	Truderung, O. A. H., J. C. Sagi, A. F. Semsei and C. Szalai	2021	Melanoma susceptibility: An update on genetic and epigenetic finding	International Journal of Molecular Epidemiology and Genetics	Records excluded after screening
1019	Tung, C. B., C. Y. Li and H. Y. Lin	2021	Multi-omics reveal the immunological role and the theragnostic value of miR-216a/GDF15 axis in human colon adenocarcinoma	International Journal of Molecular Sciences	Records excluded after screening
1020	Turner, R. C., J. T. Melnychuk, W. Chen, D. Jones and S. G. Krishna	2022	Molecular Analysis of Pancreatic Cyst Fluid for the Management of Intraductal Papillary Mucinous Neoplasms	Diagnostics	Records excluded after screening
1021	Udgata, S., N. Takenaka, W. R. Bamlet, A. L. Oberg, S. S. Yee, E. L. Carpenter, D. Herman, J. Kim, G. M. Petersen and K. S. Zaret	2021	THBS2/CA19-9 Detecting Pancreatic Ductal Adenocarcinoma at Diagnosis Underperforms in Prediagnostic Detection: Implications for Biomarker Advancement	Cancer Prev Res (Phila)	Records excluded after screening
1022	Ulutas, K. T. and I. S. Sarici	2016	Could neutrophil/lymphocyte ratio, platelet/lymphocyte ratio, and mean platelet volume serve as potential biomarkers for detection of resectable pancreas ca?	International Journal of Clinical and Experimental Medicine	Records excluded after screening
1023	Umin	2017	The feasibility study for pancreatic cancer screening by plasma biomarker using apoA2-isoforms	https://trialsearch.who.int/Trial2.aspx?TrialID=JPRN-UMIN000028015	Records excluded after screening
1024	Underwood, J. J., R. S. Quadri, S. P. Kalva, H. Shah, A. R. Sanjeeviah, M. S. Beg and P. D. Sutphin	2020	Liquid biopsy for cancer: Review and implications for the radiologist	Radiology	Records excluded after screening
1025	Unger, K., K. Y. Mehta, P. Kaur, Y. Wang, S. S. Menon, S. K. Jain, R. A. Moonjelly, S. Suman, K. Datta, R. Singh, P. Fogel and A. K. Cheema	2018	Metabolomics based predictive classifier for early detection of pancreatic ductal adenocarcinoma	Oncotarget	Records excluded after screening
1026	Urayama, S.	2015	Pancreatic cancer early detection: Expanding higher-risk group with clinical and metabolomics parameters	World Journal of Gastroenterology	Records excluded after screening
1027	Urayama, S., A. Azarm and R. Khan	2015	Endoscopic ultrasound and pancreatic cancer: Advancing the application of technology for early detection	Journal of Interventional Gastroenterology	Records excluded after screening
1028	Uygun, Z. O. and F. G. Sağin	2021	Detection of circulating prostate cancer cells via prostate specific membrane antigen by chronoimpedimetric aptasensor	Turkish Journal of Biochemistry	Records excluded after screening

1029	Vader, W., T. Brouwer, S. Basten, A. Farina Sarasqueta, J. Overkamp, K. Yan, L. Price, H. Morreau, B. Bonsing and A. Vahrmeijer	2018	In vitro clinical trial for pancreatic cancer patients	ESMO Open	Records excluded after screening
1030	Van de Wiele, C., M. Van Vlaenderen, L. D'Hulst, A. Delcourt, D. Copin, B. De Spiegeleer and A. Maes	2017	Metabolic and morphological measurements of subcutaneous and visceral fat and their relationship with disease stage and overall survival in newly diagnosed pancreatic adenocarcinoma : Metabolic and morphological fat measurements in pancreatic adenocarcinoma	Eur J Nucl Med Mol Imaging	Records excluded after screening
1031	Van Scherpenzeel, M., E. Willems and D. J. Lefeber	2016	Clinical diagnostics and therapy monitoring in the congenital disorders of glycosylation	Glycoconjugate Journal	Records excluded after screening
1032	Vandenbrouck, Y., D. Christiany, F. Combes, V. Loux and V. Brun	2019	Bioinformatics Tools and Workflow to Select Blood Biomarkers for Early Cancer Diagnosis: An Application to Pancreatic Cancer	Proteomics	Records excluded after screening
1033	Vanek, P., M. Eid, R. Psar, V. Zoundjiekpon, O. Urban and L. Kunovsky	2022	Current trends in the diagnosis of pancreatic cancer	Vnitrni Lekarstvi	Records excluded after screening
1034	Vellan, C. J., J. J. Jayapalan, B. K. Yoong, A. Abdul-Aziz, S. Mat-Junit and P. Subramanian	2022	Application of Proteomics in Pancreatic Ductal Adenocarcinoma Biomarker Investigations: A Review	International Journal of Molecular Sciences	Records excluded after screening
1035	Velstra, B., M. A. Vonk, B. A. Bonsing, B. J. Mertens, S. Nicolardi, A. Huijbers, H. Vasen, A. M. Deelder, W. E. Mesker, Y. E. van der Burgt and R. A. Tollenaar	2015	Serum peptide signatures for pancreatic cancer based on mass spectrometry: a comparison to CA19-9 levels and routine imaging techniques	J Cancer Res Clin Oncol	Incomplete diagnostic performance data provided
1036	Verel-Yilmaz, Y., J. P. Fernández, A. Schä fer, S. Nevermann, L. Cook, N. Gercke, F. Helmprobst, C. Jaworek, E. Pogge von Strandmann, A. Pagenstecher, D. K. Bartsch, J. W. Bartsch and E. P. Slater	2021	Extracellular Vesicle-Based Detection of Pancreatic Cancer	Frontiers in Cell and Developmental Biology	Non-diagnostic studies
1037	Verhoeven, Y., S. Tilborghs, J. Jacobs, J. De Waele, D. Quatannens, C. Deben, H. Prenen, P. Pauwels, X. B. Trinh, A. Wouters, E. L. J. Smits, F. Lardon and P. A. van Dam	2020	The potential and controversy of targeting STAT family members in cancer	Seminars in Cancer Biology	Records excluded after screening

1038	Vila-Navarro, E., S. Duran-Sanchon, M. Vila-Casadesús, L. Moreira, À. Ginès, M. Cuatrecasas, J. J. Lozano, L. Bujanda, A. Castells and M. Gironella	2019	Novel Circulating miRNA Signatures for Early Detection of Pancreatic Neoplasia	Clin Transl Gastroenterol	Records excluded after screening
1039	Vinik, A. I. and C. Chaya	2016	Clinical Presentation and Diagnosis of Neuroendocrine Tumors	Hematology/Oncology Clinics of North America	Records excluded after screening
1040	Visani, M., G. Acquaviva, S. Fiorino, M. L. Bacchi Reggiani, M. Masetti, E. Franceschi, A. Fornelli, E. Jovine, C. Fabbri, A. A. Brandes, G. Tallini, A. Pession and D. De Biase	2015	Contribution of microRNA analysis to characterisation of pancreatic lesions: A review	Journal of Clinical Pathology	Records excluded after screening
1041	Visser, I. J., I. J. M. Levink, M. P. Peppelenbosch, G. M. Fuhler, M. J. Bruno and D. L. Cahen	2022	Systematic review and meta-analysis: Diagnostic performance of DNA alterations in pancreatic juice for the detection of pancreatic cancer	Pancreatology	Records excluded after screening
1042	Wan, X. X., H. L. Wang, B. T. Shi, Y. Guo, S. Y. Liu and X. W. Wang	2023	An enzyme activated fluorescent probe for LTA₄H activity sensing and its application in cancer screening	Talanta	Records excluded after screening
1043	Wang, H., X. Li, L. A. Lai, T. A. Brentnall, D. W. Dawson, K. A. Kelly, R. Chen and S. Pan	2021	X-aptamers targeting Thy-1 membrane glycoprotein in pancreatic ductal adenocarcinoma	Biochimie	Records excluded after screening
1044	Wang, H. W., Y. Wang, D. J. Zhang and P. F. Li	2024	Circulating nucleosomes as potential biomarkers for cancer diagnosis and treatment monitoring	International Journal of Biological Macromolecules	Records excluded after screening
1045	Wang, H. Y., C. H. Hsieh, C. N. Wen, Y. H. Wen, C. H. Chen and J. J. Lu	2016	Cancers screening in an asymptomatic population by using multiple tumour markers	PLoS ONE	Records excluded after screening
1046	Wang, J., M. Raimondo, S. Guha, J. Chen, L. Diao, X. Dong, M. B. Wallace, A. M. Killary, M. L. Frazier, T. A. Woodward, J. Wang and S. Sen	2014	Circulating microRNAs in pancreatic juice as candidate biomarkers of pancreatic cancer	Journal of Cancer	Not early-stage
1047	Wang, J., Y. Zhu, J. Chen, Y. Yang, L. Zhu, J. Zhao, Y. Yang, X. Cai, C. Hu, R. Rosell, X. Sun and P. Cao	2020	Identification of a novel PAK1 inhibitor to treat pancreatic cancer	Acta Pharmaceutica Sinica B	Records excluded after screening
1048	Wang, L., A. R. Luedtke and Y. Huang	2020	Assessing the incremental value of new biomarkers based on OR rules	Biostatistics	Records excluded after screening

1049	Wang, L., L. Wang, X. Sun, L. Fu, X. Wang, X. Wang, L. Chen and Y. Huang	2024	Detection of uridine diphosphate glucuronosyltransferase 1A1 for pancreatic cancer imaging and treatment via a "turn-on" fluorescent probe	The Analyst	Records excluded after screening
1050	Wang, L., Z. Wu, C. Xu and H. Ye	2023	Ferroptosis-related genes prognostic signature for pancreatic cancer and immune infiltration: potential biomarkers for predicting overall survival	Journal of Cancer Research and Clinical Oncology	Records excluded after screening
1051	Wang, L. X., L. Y. Wang, X. Sun, L. L. Fu, X. L. Wang, X. Y. Wang, L. X. Chen and Y. Huang	2024	Detection of uridine diphosphate glucuronosyltransferase 1A1 for pancreatic cancer imaging and treatment <i>via</i> a "turn-on" fluorescent probe	Analyst	Records excluded after screening
1052	Wang, M., Y. Yang and Z. Liao	2020	Diabetes and cancer: Epidemiological and biological links	World Journal of Diabetes	Records excluded after screening
1053	Wang, R., B. Cheng and J. Wang	2018	K-RAS mutation analysis by digital PCR in eusguided FNA cytology specimens and CTDNA improve pancreatic cancer diagnosis	United European Gastroenterology Journal	Records excluded after screening
1054	Wang, S., X. Chen and M. Tang	2014	Quantitative assessment of the diagnostic role of MUC1 in pancreatic ductal adenocarcinoma	Tumor Biology	Records excluded after screening
1055	Wang, S., S. Wen, P. Guo, H. Liu, J. Feng and H. Huang	2020	Understanding metabolomic characteristics of pancreatic ductal adenocarcinoma by HR-MAS NMR detection of pancreatic tissues	Journal of Pharmaceutical and Biomedical Analysis	Records excluded after screening
1056	Wang, S., K. Zhang, S. Tan, J. Xin, Q. Yuan, H. Xu, X. Xu, Q. Liang, D. C. Christiani, M. Wang, L. Liu and M. Du	2021	Circular RNAs in body fluids as cancer biomarkers: the new frontier of liquid biopsies	Molecular Cancer	Records excluded after screening
1057	Wang, S. L., X. D. Chen and M. Y. Tang	2014	Quantitative assessment of the diagnostic role of human telomerase activity from pancreatic juice in pancreatic cancer	Tumor Biology	Records excluded after screening
1058	Wang, X., Y. Li, H. Tian, J. Qi, M. Li, C. Fu, F. Wu, Y. Wang, D. Cheng, W. Zhao, C. Zhang, T. Wang, J. Rao and W. Zhang	2014	Macrophage inhibitory cytokine 1 (MIC-1/GDF15) as a novel diagnostic serum biomarker in pancreatic ductal adenocarcinoma	BMC Cancer	Studies included in review
1059	Wang, X. H., P. Meng, S. H. Li, J. H. Tan, B. D. Su, Q. Cheng and X. L. Yang	2023	Detection of two markers for pancreatic cancer (CEA, CA199) based on a nano-silicon sphere-cyclodextrin recognition platform	Alexandria Engineering Journal	Records excluded after screening
1060	Wang, X. H., J. Zhang, Z. J. Han, L. H. Ma and Y. M. Li	2022	¹⁸F-labeled Dimer-Sansalvamide A Cyclodecapeptide: A Novel Diagnostic Probe to Discriminate Pancreatic Cancer from Inflammation in a Nude Mice Model	Journal of Cancer	Records excluded after screening
1061	Wang, Y.	2021	Applications of Lipidomics in Tumor Diagnosis and Therapy.		Records excluded after screening
1062	Wang, Y., K. Liu, Q. Ma, Y. Tan, W. Du, Y. Lv, Y. Tian and H. Wang	2019	Pancreatic cancer biomarker detection by two support vector strategies for recursive feature elimination	Biomarkers in Medicine	Records excluded after screening

1063	Wang, Y., J. Zheng, Z. Li, R. Jiang, J. Peng, J. Sun, G. Yang, X. R. Yang, A. Huang, Y. Wang, Y. Jie, X. Liu, F. Gao, X. Wu, D. Wang, W. Wu, W. Lou, J. Zhou and J. Fan	2020	Development of a novel liquid biopsy test to diagnose and locate gastrointestinal cancers	Journal of Clinical Oncology	Records excluded after screening
1064	Wáng, Y. X. J., J. S. Gong and R. Loffroy	2015	On pancreatic cancer screening by magnetic resonance imaging with the recent evidence by Del Chiaro and colleagues	Chinese Journal of Cancer Research	Records excluded after screening
1065	Wang, Z. Y., X. Q. Ding, H. Zhu, R. X. Wang, X. R. Pan and J. H. Tong	2019	KRAS Mutant Allele Fraction in Circulating Cell-Free DNA Correlates With Clinical Stage in Pancreatic Cancer Patients	Frontiers in Oncology	Incomplete diagnostic performance data provided
1066	Watanabe, H., M. Kanematsu, K. Tanaka, S. Osada, H. Tomita, A. Hara, S. Goshima, H. Kondo, H. Kawada, Y. Noda, Y. Tanahashi, N. Kawai, K. Yoshida and N. Moriyama	2014	Fibrosis and postoperative fistula of the pancreas: correlation with MR imaging findings--preliminary results	Radiology	Small sample size
1067	Watcharanurak, P., A. Mutirangura, V. Aksornkitti, N. Bhummaphan and C. Puttipanyalears	2024	The high FKBP1A expression in WBCs as a potential screening biomarker for pancreatic cancer	Scientific reports	Not early-stage
1068	Weber, C.	2015	Biomarkers: The challenge to find biomarkers for the early detection of pancreatic cancer	Nature Reviews Gastroenterology and Hepatology	Records excluded after screening
1069	Wedeken, L., S. Forbrig, K. Herrera-Glomm, J. Loskutov, U. Pfohl, I. Piven, M. Poehle, M. J. Regenbrecht, B. Seller, C. Yapto, S. Finkler, L. Ruhe, Q. Graf Adelmann, C. Reinhard, M. Flechner, K. Uhlig, D. Kaul, S. Roohani, M. Niethard, R. Sauer and C. R. Regenbrecht	2024	PD3D®models as jacks-of-all-trades for cancer research and therapy response prediction	Cancer Research	Records excluded after screening
1070	Wei, J., L. Yang, Y. N. Wu and J. Xu	2020	Serum miR-1290 and miR-1246 as Potential Diagnostic Biomarkers of Human Pancreatic Cancer	Journal of Cancer	Not early-stage
1071	Wei, L., K. Yao, S. Gan and Z. Suo	2018	Clinical utilization of serum- or plasma-based miRNAs as early detection biomarkers for pancreatic cancer: A meta-analysis up to now	Medicine (United States)	Records excluded after screening
1072	Wei, T., P. Ye, X. Peng, L. L. Wu and G. Y. Yu	2016	Circulating adiponectin levels in various malignancies: An updated meta-analysis of 107 studies	Oncotarget	Records excluded after screening

1073	Wen, W., J. Liu and G. G. Xiao	2020	A novel detection tool being used for miRNA detection in human serum	Pancreas	Records excluded after screening
1074	Wen, Y. H., P. Y. Chang, C. M. Hsu, H. Y. Wang, C. T. Chiu and J. J. Lu	2015	Cancer screening through a multi-analyte serum biomarker panel during health check-up examinations: Results from a 12-year experience	Clinica Chimica Acta	Records excluded after screening
1075	Wen, Y. R., X. W. Lin, Y. W. Zhou, L. Xu, J. L. Zhang, C. Y. Chen and J. He	2024	N-glycan biosignatures as a potential diagnostic biomarker for early-stage pancreatic cancer	World Journal of Gastrointestinal Oncology	Studies included in review
1076	Wiewiora, M., J. Jopek, E. Swietochowska, M. Grynkiewicz and J. Piecuch	2024	Evaluations of the combined use of blood- and tissue-based protein biomarkers for pancreatic cancer	Clinical Hemorheology and Microcirculation	Records excluded after screening
1077	Wilcox, B., J. Blume, K. Swaminathan, P. Williams, M. Khadka, J. Deyarmin, S. Ramaswamy, Y. Kodama, B. Young, C. Belthangady, M. Liu, M. Yang and P. Ma	2022	Deep, unbiased multi-omics approach for identification of pancreatic cancer biomarkers from blood	Cancer Research	Records excluded after screening
1078	Willms, A., C. Müller, H. Julich, N. Klein, R. Schwab, C. Güsgen, I. Richardsen, S. Schaaf, M. Krawczyk, M. Krawczyk, F. Lammert, D. Schuppan, V. Lukacs-Kornek and M. Kornek	2016	Tumour-associated circulating microparticles: A novel liquid biopsy tool for screening and therapy monitoring of colorectal carcinoma and other epithelial neoplasia	Oncotarget	Records excluded after screening
1079	Wlodarczyk, B., A. Borkowska, P. Wlodarczyk, E. Malecka-Panas and A. Gasiorowska	2020	Early PDAC detection: IGF axis protein levels in patients with new onset diabetes as a useful indicator	United European Gastroenterology Journal	Non-diagnostic studies
1080	Wolrab, D., R. Jirásko, E. Cífková, M. Höring, D. Mei, M. Chocholousková, O. Peterka, J. Idkowiak, T. Hrnciarová, L. Kuchar, R. Ahrends, R. Brumarová, D. Friedecky, G. Vivo-Truyols, P. Skrha, J. Skrha, R. Kucera, B. Melichar, G. Liebisch, R. Burkhardt, M. R. Wenk, A. Cazenave-Gassiot, P. Karásek, I. Novotny, K. Greplová, R. Hrstka and M. Holcapek	2022	Lipidomic profiling of human serum enables detection of pancreatic cancer	Nature Communications	Studies included in review

1081	Wong, H. L., K. Bushell, J. Karasinska, S. Arthur, R. Morin, D. F. Schaeffer and D. J. Renouf	2016	Clinical utility of circulating tumor DNA (ctDNA) in resectable pancreatic ductal adenocarcinoma (PDAC)	Journal of Clinical Oncology	Records excluded after screening
1082	Wong, M. H., A. Xue, S. M. Julovi, N. Pavlakis, J. S. Samra, T. J. Hugh, A. J. Gill, L. Peters, R. C. Baxter and R. C. Smith	2014	Cotargeting of epidermal growth factor receptor and PI3K overcomes PI3K-Akt oncogenic dependence in pancreatic ductal adenocarcinoma	Clinical Cancer Research	Records excluded after screening
1083	Wood, L. D., M. B. Yurgelun and M. G. Goggins	2019	Genetics of Familial and Sporadic Pancreatic Cancer	Gastroenterology	Records excluded after screening
1084	Wu, D. M., J. Shi, T. Liu, S. H. Deng, R. Han and Y. Xu	2018	Integrated analysis reveals down-regulation of <i>SPARCL1</i> is correlated with cervical cancer development and progression	Cancer Biomarkers	Records excluded after screening
1085	Wu, D. N., J. Jen, C. P. Hsu, Y. T. Chang and C. M. Hu	2024	1253 NMR-BASED METABOLOMICS-AI (SVM-SVC-RBF) APPROACH FOR EARLY DIAGNOSIS IN PANCREATIC DUCTAL ADENOCARCINOMA PATIENTS	Gastroenterology	Records excluded after screening
1086	Wu, G. F., J. Zhu, G. J. Weng, H. Y. Cai, J. J. Li and J. W. Zhao	2024	Morphology and optical properties of Au-Ag hybrid nanoparticles regulation and its ultra-sensitive SERS immunoassay detection in carbohydrate antigen 19-9	Talanta	Records excluded after screening
1087	Wu, H., S. Guo, X. Liu, Y. Li, Z. Su, Q. He, X. Liu, Z. Zhang, L. Yu, X. Shi, S. Gao, H. Wang, Y. Pan, C. Ma, R. Liu, M. Dai, G. Jin and Z. Liang	2022	Noninvasive detection of pancreatic ductal adenocarcinoma using the methylation signature of circulating tumour DNA	BMC Medicine	Studies included in review
1088	Wu, H., S. Ou, H. Zhang, R. Huang, S. Yu, M. Zhao and S. Tai	2022	Advances in biomarkers and techniques for pancreatic cancer diagnosis	Cancer Cell International	Records excluded after screening
1089	Wu, H. J., K. Mehta, S. S. Menon, K. Unger, M. S. Fiandaca, Y. Fallah, M. Mapstone, H. J. Federoff and A. K. Cheema	2017	Prognostic biomarkers of PDAC-a cross-validation study	Cancer Research	Records excluded after screening
1090	Wu, P., X. Yang, L. Qiao and Y. Gong	2022	Identification of Six Genes as Diagnostic Markers for Colorectal Cancer Detection by Integrating Multiple Expression Profiles	Journal of Oncology	Records excluded after screening
1091	Wu, R. I., W. J. Yoon, W. R. Brugge, M. Mino-Kenudson and M. B. Pitman	2014	Endoscopic ultrasound-guided fine needle aspiration (EUS-FNA) contributes to a triple-negative test in preoperative screening of pancreatic cysts	Cancer Cytopathol	Records excluded after screening
1092	Wu, W.	2022	Early Detection of Pancreatic Cancer: Are We Ready for Prime Time?	Gastroenterology	Records excluded after screening
1093	Wu, W., X. Xia, C. Cheng, L. Niu, J. Wu and Y. Qian	2021	Serum soluble PD-L1, PD-L2, and B7-H5 as potential diagnostic biomarkers of human pancreatic cancer	Clinical Laboratory	Records excluded after screening

1094	Wu, X., Z. X. Zhang, X. Y. Chen, Y. L. Xu, N. Yin, J. Yang, D. M. Zhu, D. C. Li and J. Zhou	2019	A Panel of Three Biomarkers Identified by iTRAQ for the Early Diagnosis of Pancreatic Cancer	Proteomics - Clinical Applications	Records excluded after screening
1095	Wu, X. R., J. F. Wang, J. J. Liang and H. W. Wang	2014	The d-dimer's clinical value in the diagnosis and treatment of the pancreatic cancer	Journal of Digestive Diseases	Records excluded after screening
1096	Wu, Y., Q. Li, R. Zhang, X. Dai, W. Chen and D. Xing	2021	Circulating microRNAs: Biomarkers of disease	Clinica Chimica Acta	Records excluded after screening
1097	Wu, Y. H., Y. P. Hung, N. C. Chiu, R. C. Lee, C. P. Li, Y. Chao, Y. M. Shyr, S. E. Wang, S. C. Chen, S. H. Lin, Y. H. Chen, Y. M. Kang, S. M. Hsu, S. H. Yen, J. Y. Wu, K. D. Lee, H. E. Tseng, J. R. Tsai, J. H. Tang, J. F. Chiou, T. Burnouf, Y. J. Chen, P. Y. Wang and L. S. Lu	2022	Correlation between drug sensitivity profiles of circulating tumour cell-derived organoids and clinical treatment response in patients with pancreatic ductal adenocarcinoma	European Journal of Cancer	Small sample size
1098	Xi, S. J., W. Q. Cai, Q. Q. Wang and X. C. Peng	2021	Role of circular RNAs in gastrointestinal tumors and drug resistance	World Journal of Clinical Cases	Records excluded after screening
1099	Xiao, D., Z. Dong, L. Zhen, G. Xia, X. Huang, T. Wang, H. Guo, B. Yang, C. Xu, W. Wu, X. Zhao and H. Xu	2020	Combined exosomal GPC1, CD82, and serum CA19-9 as multiplex targets: A specific, sensitive, and reproducible detection panel for the diagnosis of pancreatic cancer	Molecular Cancer Research	Records excluded after screening
1100	Xiao, G., H. Z. Ge, Q. C. Yang, Z. Zhang, L. Y. Cheng, S. L. Cao, J. Ji, J. Zhang and Z. Yue	2022	Light-addressable photoelectrochemical sensors for multichannel detections of GPC1, CEA and GSH and its applications in early diagnosis of pancreatic cancer	Sensors and Actuators B-Chemical	Records excluded after screening
1101	Xiao, G., J. Guo and X. Guo	2024	Expert consensus on the molecular diagnosis of early-stage pancreatic cancer(2023 edition)	Journal of Clinical Hepatology	Records excluded after screening
1102	Xiao, H., Y. Zhang, Y. Kim, S. Kim, J. J. Kim, K. M. Kim, J. Yoshizawa, L. Y. Fan, C. X. Cao and D. T. W. Wong	2016	Differential Proteomic Analysis of Human Saliva using Tandem Mass Tags Quantification for Gastric Cancer Detection	Scientific Reports	Records excluded after screening
1103	Xie, G., L. Lu, Y. Qiu, Q. Ni, W. Zhang, Y. T. Gao, H. A. Risch, H. Yu and W. Jia	2015	Plasma metabolite biomarkers for the detection of pancreatic cancer	Journal of Proteome Research	Studies included in review
1104	Xie, I. Y., Z. M. Liu, K. Ng, E. Flores-Figueroa, G. H. Jang, A. X. Zhang, S. Ramotar, A. Dodd, J. Wilson, G. M. O'Kane, J. J. Knox, S. Gallinger and F. Notta	2022	High-throughput organoid drug screening to identify molecular vulnerabilities in pancreatic cancer	Cancer Research	Records excluded after screening

1105	Xie, J., X. Zhou, R. Wang, J. Zhao, J. Tang, Q. Zhang, Y. Du and Y. Pang	2021	Identification of potential diagnostic biomarkers in MMPs for pancreatic carcinoma	Medicine (United States)	Records excluded after screening
1106	Xie, W., M. Chu, G. Song, Z. Zuo, Z. Han, C. Chen, Y. Li and Z. W. Wang	2022	Emerging roles of long noncoding RNAs in chemoresistance of pancreatic cancer	Seminars in Cancer Biology	Records excluded after screening
1107	Xie, Z., X. Chen, J. Li, Y. Guo, H. Li, X. Pan, J. Jiang, H. Liu and B. Wu	2016	Salivary HOTAIR and PVT1 as novel biomarkers for early pancreatic cancer	Oncotarget	Records excluded after screening
1108	Xie, Z., X. Yin, B. Gong, W. Nie, B. Wu, X. Zhang, J. Huang, P. Zhang, Z. Zhou and Z. Li	2015	Salivary microRNAs show potential as a noninvasive biomarker for detecting resectable pancreatic cancer	Cancer Prevention Research	Not early-stage
1109	Xiong, G., M. Feng, G. Yang, S. Zheng, X. Song, Z. Cao, L. You, L. Zheng, Y. Hu, T. Zhang and Y. Zhao	2017	The underlying mechanisms of non-coding RNAs in the chemoresistance of pancreatic cancer	Cancer Letters	Records excluded after screening
1110	Xiong, H., G. Huang, Y. Zhu, R. Chen, L. Zuo and H. Liu	2023	Circ-SHPRH in human cancers: a systematic review and meta-analysis	Frontiers in Cell and Developmental Biology	Records excluded after screening
1111	Xu, C., E. Jun, Y. Toiyama, J. Bolton, A. Taketomi, S. C. Kim and A. Goel	2023	A CIRCRNA-BASED LIQUID BIOPSY ASSAY FOR THE NONINVASIVE AND EARLY DETECTION OF PANCREATIC DUCTAL ADENOCARCINOMA: A MULTICENTER COHORT STUDY	Gastroenterology	Records excluded after screening
1112	Xu, H., L. Zhang, H. Kang, J. Liu, J. Zhang, J. Zhao and S. Liu	2021	Metabolomics Identifies Biomarker Signatures to Differentiate Pancreatic Cancer from Type 2 Diabetes Mellitus in Early Diagnosis	International Journal of Endocrinology	Non-diagnostic studies
1113	Xu, K., H. Jiang, J. Shi, J. Huang, N. Liu, X. Gong, X. Bo, Q. Liu, N. Chen, Y. Yang and C. Wang	2020	Systematic evaluation of the development of diagnosis and screening methods for pancreatic cancer	Chinese Journal of Evidence-Based Medicine	Records excluded after screening
1114	Xu, K., Z. Qiu, L. Xu, X. Qiu, L. Hong and J. Wang	2021	Increased levels of circulating circular RNA (hsa_circ_0013587) may serve as a novel biomarker for pancreatic cancer	Biomark Med	Records excluded after screening
1115	Xu, W., M. Zhang, L. Liu, M. Yin, C. Xu and Z. Weng	2022	Association of mucin family members with prognostic significance in pancreatic cancer patients: A meta-analysis	PLoS ONE	Records excluded after screening
1116	Xu, X., Y. Xiao, B. Hong, B. Hao and Y. Qian	2019	Combined detection of CA19-9 and B7-H4 in the diagnosis and prognosis of pancreatic cancer	Cancer Biomarkers	Not early-stage
1117	Xu, Y., T. Qin, J. Li, X. Wang, C. Gao, C. Xu, J. Hao, J. Liu, S. Gao and H. Ren	2017	Detection of circulating tumor cells using negative enrichment immunofluorescence and an in situ hybridization system in pancreatic cancer	International Journal of Molecular Sciences	Records excluded after screening
1118	Xu, Y., D. Wang, T. Kuang, W. Wu, X. Xu, D. Jin, H. Zhang, S. Zhong, Y. Wang and W. Lou	2020	Nanomaterials augmented LDITOF-MS for pancreatic ductal adenocarcinoma diagnosis and classification	Journal of Clinical Oncology	Records excluded after screening

1119	Xu, Y. F., B. N. Hannafon, Y. D. Zhao, R. G. Postier and W. Q. Ding	2017	Plasma exosome miR-196a and miR-1246 are potential indicators of localized pancreatic cancer	Oncotarget	Small sample size
1120	Xue, J., E. Jia, N. Ren, A. Lindsay and H. Yu	2019	Circulating microRNAs as promising diagnostic biomarkers for pancreatic cancer: A systematic review	OncoTargets and Therapy	Records excluded after screening
1121	Xue, M., M. Shi, J. Xie, J. Zhang, L. Jiang, X. Deng, C. Peng, B. Shen, H. Xu and H. Chen	2021	Serum tRNA-derived small RNAs as potential novel diagnostic biomarkers for pancreatic ductal adenocarcinoma	American Journal of Cancer Research	Not early-stage
1122	Yako, Y. Y., M. Brand, M. Smith and D. Kruger	2017	Inflammatory cytokines and angiogenic factors as potential biomarkers in South African pancreatic ductal adenocarcinoma patients: A preliminary report	Pancreatology	Not early-stage
1123	Yamada, K., K. Higashi, H. Nagahori and K. Saito	2019	Circulating natural antibodies against 3'-sialyllactose complement the diagnostic performance of CA19-9 for the early detection of pancreatic ductal adenocarcinoma	Cancer Biomarkers	Studies included in review
1124	Yamaguchi, K., J. Koya, K. Mizuno, K. Yoshifuji, Y. Ito, M. Yuasa, Y. Saito, M. Tabata, S. Shingaki, Y. Kogure, K. Nakashima, K. Ohshima and K. Kataoka	2022	High-Throughput Screening to Elucidate In Vivo Oncogenicity of Loss-of-Function Alterations in Lymphoid Malignancies	Blood	Records excluded after screening
1125	Yamamoto, K., A. Opina, D. Sail, B. Blackman, K. Saito, J. R. Brender, R. M. Malinowski, T. Seki, N. Oshima, D. R. Crooks, S. Kishimoto, Y. Saida, Y. Otowa, P. L. Choyke, J. H. Ardenkjær-Larsen, J. B. Mitchell, W. M. Linehan, R. E. Swenson and M. C. Krishna	2021	Real-Time insight into in vivo redox status utilizing hyperpolarized [1-13C] N-acetyl cysteine	Scientific reports	Records excluded after screening
1126	Yamusah, N.	2021	Elusive early diagnosis of insulinomas	Journal of Clinical and Translational Endocrinology: Case Reports	Records excluded after screening
1127	Yan, L. N., X. Zhang, F. Xu, Y. Y. Fan, B. Ge, H. Guo and Z. L. Li	2020	Four-microRNA signature for detection of type 2 diabetes	World Journal of Clinical Cases	Records excluded after screening
1128	Yang, B., Y. Gai, X. Song, X. Lv, D. Zeng, R. An and X. Lan	2023	Design, screening, and evaluation of albumin binder-conjugated heterodimer tracers for cancer diagnosis and treatment	Journal of Nuclear Medicine	Records excluded after screening

1129	Yang, C., H. Yan, N. Tang, S. Chakravarty, Y. Zou, X. Xu and R. T. Chen	2019	Silicon photonics chip based biosensors for biomarker detection of pancreatic cancer biomarkers	Basic and Clinical Pharmacology and Toxicology	Records excluded after screening
1130	Yang, G., W. Guan, Z. Cao, W. Guo, G. Xiong, F. Zhao, M. Feng, J. Qiu, Y. Liu, M. Q. Zhang, L. You, T. Zhang, Y. Zhao and J. Gu	2021	Integrative genomic analysis of gemcitabine resistance in pancreatic cancer by patient-derived xenograft models	Clinical Cancer Research	Records excluded after screening
1131	Yang, G. B.	2015	Clinical value of serum cancer antigen 19-9 as a tumor screening marker among healthy individuals	Journal of B.U.ON.	Non-diagnostic studies
1132	Yang, H., W. Li, L. Ren, Y. Yang, Y. Zhang, B. Ge, S. Li, X. Zheng, J. Liu, S. Zhang, G. Du, B. Tang, H. Wang and J. Wang	2023	Progress on diagnostic and prognostic markers of pancreatic cancer	Oncology Research	Records excluded after screening
1133	Yang, J., Z. Gui, S. Tan, J. Qiao, D. Li, O. Y. Ibhagui, H. Yang, K. Hekmatyar, Y. Meng, N. Wang, Y. Xie, P. Z. Sun, E. Seki, D. Li and H. Grossniklaus	2022	Non-invasive Mapping of molecular determinants for multiple cancer and metastatic aggressiveness by Precision MRI (pMRI)	Molecular Imaging and Biology	Records excluded after screening
1134	Yang, J., J. Li, R. Zhu, H. Zhang, Y. Zheng, W. Dai, F. Wang, M. Shen, K. Chen, P. Cheng, Y. Zhang, C. Wang, J. Wang, Y. Xia, J. Lu, Y. Zhou and C. Guo	2014	K-ras mutational status in cytohistological tissue as a molecular marker for the diagnosis of pancreatic cancer: A systematic review and meta-analysis	Disease Markers	Records excluded after screening
1135	Yang, J., R. Xu, C. Wang, J. Qiu, B. Ren and L. You	2021	Early screening and diagnosis strategies of pancreatic cancer: a comprehensive review	Cancer Communications	Records excluded after screening
1136	Yang, J., Y. Zhang, X. Gao, Y. Yuan, J. Zhao, S. Zhou, H. Wang, L. Wang, G. Xu, X. Li, P. Wang, X. Zou, D. Zhu, Y. Lv and S. Zhang	2021	Plasma-Derived Exosomal ALIX as a Novel Biomarker for Diagnosis and Classification of Pancreatic Cancer	Frontiers in Oncology	Records excluded after screening
1137	Yang, J. Y. and B. S. Qiu	2021	The Advance of Magnetic Resonance Elastography in Tumor Diagnosis	Frontiers in Oncology	Records excluded after screening
1138	Yang, J. Y., Y. W. Sun, D. J. Liu, J. F. Zhang, J. Li and R. Hua	2014	MicroRNAs in stool samples as potential screening biomarkers for pancreatic ductal adenocarcinoma cancer	American Journal of Cancer Research	Not early-stage
1139	Yang, K. S., D. Ciprani, A. O'Shea, A. S. Liss, R. Yang, S. Fletcher-Mercaldo, M. Mino-Kenudson, C. Fernández-del Castillo and R. Weissleder	2021	Extracellular Vesicle Analysis Allows for Identification of Invasive IPMN	Gastroenterology	Records excluded after screening

1140	Yang, K. S., H. Im, S. Hong, I. Pergolini, A. F. del Castillo, R. Wang, S. Clardy, C. H. Huang, C. Pille, S. Ferrone, R. Yang, C. M. Castro, H. Lee, C. F. del Castillo and R. Weissleder	2017	Multiparametric plasma EV profiling facilitates diagnosis of pancreatic malignancy	Science Translational Medicine	Records excluded after screening
1141	Yang, L.	2021	Folate-Receptor Positive Circulating Tumor Cell Is a Potential Diagnostic Marker of Prostate Cancer	Frontiers in Oncology	Records excluded after screening
1142	Yang, L., R. Cui, Y. Li, K. Liang, M. Ni and Y. Gu	2020	Hypoxia-induced TGFBI as a serum biomarker for laboratory diagnosis and prognosis in patients with pancreatic ductal adenocarcinoma	Lab Medicine	Records excluded after screening
1143	Yang, L., W. C. Wei, X. N. Meng, J. Gao, N. Guo, F. T. Wu and W. W. Zeng	2019	Significance of IL28RA in diagnosis of early pancreatic cancer and its regulation to pancreatic cancer cells by JAK/STAT signaling pathway - effects of IL28RA on pancreatic cancer	Eur Rev Med Pharmacol Sci	Records excluded after screening
1144	Yang, M. and C. Y. Zhang	2021	Diagnostic biomarkers for pancreatic cancer: An update	World Journal of Gastroenterology	Records excluded after screening
1145	Yang, M. X., R. F. Coates, A. Ambaye, V. Cortright, J. M. Mitchell, A. M. Buskey, R. Zubarik, J. G. Liu, S. Ades and M. M. Barry	2018	NKX2.2, PDX-1 and CDX-2 as potential biomarkers to differentiate well-differentiated neuroendocrine tumors	Biomarker Research	Records excluded after screening
1146	Yang, Y., S. Yan, H. Tian and Y. Bao	2018	Macrophage inhibitory cytokine-1 versus carbohydrate antigen 19-9 as a biomarker for diagnosis of pancreatic cancer	Medicine (United States)	Records excluded after screening
1147	Yang, Z., J. Huang, X. Wu, Y. Zhou, Y. Tang, Y. Zhu, B. Li, X. Chen and W. Yao	2024	Contribution of a Circulating 2'-O-methylated MicroRNA Panel to the Diagnosis of Pancreatic Ductal Adenocarcinoma	Journal of Cancer	Not early-stage
1148	Yao, W., X. Chen, B. Fan, L. Zeng, Z. Zhou, Z. Mao and Q. Shen	2023	Multidisciplinary team diagnosis and treatment of pancreatic cancer: Current landscape and future prospects	Frontiers in Oncology	Records excluded after screening
1149	Yates, C. J., P. J. Newey and R. V. Thakker	2015	Challenges and controversies in management of pancreatic neuroendocrine tumours in patients with MEN1	The Lancet Diabetes and Endocrinology	Records excluded after screening
1150	Ye, D., Z. Shen and S. Zhou	2019	Function of microRNA-145 and mechanisms underlying its role in malignant tumor diagnosis and treatment	Cancer Management and Research	Records excluded after screening
1151	Ye, H., H. Wang, P. Wang, C. H. Song, K. J. Wang, L. P. Dai, J. X. Shi, X. X. Liu, C. Q. Sun, X. Wang, Y. Peng, X. B. Chen and J. Y. Zhang	2019	Systematic review: Exosomal microRNAs associated with pancreatic cancer for early detection and prognosis	European Review for Medical and Pharmacological Sciences	Records excluded after screening

1152	Ye, X. K., J. Jiang, Z. Luo, J. Wei, J. He, G. Zhuang, Y. Yan, T. Yu, W. Du, J. Gu, J. Liu and Z. Gu	2016	Prediction of cancer patient responses to different treatments using personalized tumor models or models with matching genomic profiles	Journal of Clinical Oncology	Records excluded after screening
1153	Yeo, D., A. Bastian, H. Strauss, P. Saxena, P. Grimison and J. E. J. Rasko	2022	Exploring the Clinical Utility of Pancreatic Cancer Circulating Tumor Cells	International Journal of Molecular Sciences	Records excluded after screening
1154	Yi, N., X. Zhao, J. Ji, M. Xu, Y. Jiao, T. Qian, S. Zhu, F. Jiang, J. Chen and M. Xiao	2020	Serum galectin-3 as a biomarker for screening, early diagnosis, prognosis and therapeutic effect evaluation of pancreatic cancer	Journal of Cellular and Molecular Medicine	Records excluded after screening
1155	Yin, H. D., Z. J. Tan, J. Wu, J. H. Zhu, K. A. Shedden, J. Marrero and D. M. Lubman	2015	Mass-Selected Site-Specific Core-Fucosylation of Serum Proteins in Hepatocellular Carcinoma	Journal of Proteome Research	Records excluded after screening
1156	Yin, L., C. Cao, K. Zhang, F. Guo, J. Chen, M. Tu, J. Wei, J. Wu, W. Gao, Y. Miao, Z. Lu and K. Jiang	2024	Fragmentomics of cell-free DNA as a sensitive biomarker for early detection of pancreatic cancer	HPB	Records excluded after screening
1157	Ying, L., A. Sharma, A. Chhoda, N. Ruzgar, N. Hasan, R. Kwak, C. L. Wolfgang, T. H. Wang, J. W. Kunstman, R. R. Salem, L. D. Wood, C. Iacobuzio-Donahue, E. B. Schneider, J. J. Farrell and N. Ahuja	2021	Methylation-based Cell-free DNA Signature for Early Detection of Pancreatic Cancer	Pancreas	Not early-stage
1158	Yip-Schneider, M. T., R. A. Carr, H. Wu and C. M. Schmidt	2017	Prostaglandin E2: A Pancreatic Fluid Biomarker of Intraductal Papillary Mucinous Neoplasm Dysplasia	Journal of the American College of Surgeons	Non-diagnostic studies
1159	Yip-Schneider, M. T., R. Muraru, R. C. Kim, H. H. Wu, S. Sherman, A. Gutta, M. A. Al-Haddad, J. M. Dewitt and C. M. Schmidt	2023	EUS-guided fine needle aspiration-based clues to mistaken or uncertain identity: serous pancreatic cysts	HPB	Records excluded after screening
1160	Yip-Schneider, M. T., H. Wu, H. R. Allison, J. J. Easler, S. Sherman, M. A. Al-Haddad, J. M. Dewitt and C. M. Schmidt	2021	Biomarker Risk Score Algorithm and Preoperative Stratification of Patients with Pancreatic Cystic Lesions	Journal of the American College of Surgeons	Non-diagnostic studies
1161	Yip-Schneider, M. T., H. Wu, R. P. Dumas, B. A. Hancock, N. Agaram, M. Radovich and C. M. Schmidt	2014	Vascular endothelial growth factor, a novel and highly accurate pancreatic fluid biomarker for serous pancreatic cysts	Journal of the American College of Surgeons	No sample information provided

1162	Yokose, T., Y. Kabe, A. Matsuda, M. Kitago, S. Matsuda, M. Hirai, T. Nakagawa, Y. Masugi, T. Hishiki, Y. Nakamura, M. Shinoda, H. Yagi, Y. Abe, G. Oshima, S. Hori, Y. Nakano, K. Honda, A. Kashiro, C. Morizane, S. Nara, S. Kikuchi, T. Shibahara, M. Itonaga, M. Ono, N. Minegishi, S. Koshiba, M. Yamamoto, A. Kuno, H. Handa, M. Sakamoto, M. Suematsu and Y. Kitagawa	2020	O-glycan-altered extracellular vesicles: A specific serum marker elevated in pancreatic cancer	Cancers	No sample information provided
1163	Yong, B. J. C. and M. W. Diyana	2022	Low Carbohydrate Antigen 19-9 (CA 19-9) Levels in a Patient Highly Suspected of Having Caput Pancreas Tumor	Cureus Journal of Medical Science	Records excluded after screening
1164	Yoon, J. H., Y. G. Park, S. W. Nam and W. S. Park	2019	The diagnostic value of serum gastrokine 1 (GKN1) protein in gastric cancer	Cancer Medicine	Records excluded after screening
1165	Yoshinaga, T., T. Niou, T. Niihara, Y. Kajiya, E. Hori, A. Tomiyoshi, E. Tokudome, H. Nishimata, T. Takei and M. Yoshida	2018	Angiopoietin-like protein 2 is a useful biomarker for pancreatic cancer that is associated with type 2 diabetes mellitus and inflammation	Journal of Cancer	Records excluded after screening
1166	Yoshioka, Y., T. Katsuda and T. Ochiya	2018	Extracellular vesicles and encapusulated miRNAs as emerging cancer biomarkers for novel liquid biopsy	Japanese Journal of Clinical Oncology	Records excluded after screening
1167	Yoshioka, Y., M. Shimomura, K. Saito, H. Ishii, Y. Doki, H. Eguchi, T. Nakatsura, T. Itoi, M. Kuroda, M. Mori and T. Ochiya	2022	Circulating cancer-associated extracellular vesicles as early detection and recurrence biomarkers for pancreatic cancer	Cancer Science	Non-diagnostic studies
1168	Young, M. R., N. Abrams, S. Ghosh, J. A. S. Rinaudo, G. Marquez and S. Srivastava	2020	Prediagnostic Image Data, Artificial Intelligence, and Pancreatic Cancer: A Tell-Tale Sign to Early Detection	Pancreas	Records excluded after screening
1169	Young, M. R., P. D. Wagner, S. Ghosh, J. A. Rinaudo, S. G. Baker, K. S. Zaret, M. Goggins and S. Srivastava	2018	Validation of Biomarkers for Early Detection of Pancreatic Cancer <i>Summary of The Alliance of Pancreatic Cancer Consortia for Biomarkers for Early Detection Workshop</i>	Pancreas	Records excluded after screening
1170	Yu, H. J., E. Jang, A. Woo, I. W. Han, H. G. Jeon, V. T. N. Linh, S. G. Park, H. S. Jung and M. Y. Lee	2024	Cancer screening through surface-enhanced Raman spectroscopy fingerprinting analysis of urinary metabolites using surface-carbonized silver nanowires on a filter membrane	Analytica Chimica Acta	Not early-stage

1171	Yu, I. S. and W. Y. Cheung	2018	A Contemporary Review of the Treatment Landscape and the Role of Predictive and Prognostic Biomarkers in Pancreatic Adenocarcinoma	Canadian Journal of Gastroenterology and Hepatology	Records excluded after screening
1172	Yu, J., A. Ploner, M. Kordes, M. Löhr, M. Nilsson, M. E. L. de Maturana, L. Estudillo, H. Renz, A. Carrato, X. Molero, F. X. Real, N. Malats and W. Ye	2021	Plasma protein biomarkers for early detection of pancreatic ductal adenocarcinoma	International Journal of Cancer	Studies included in review
1173	Yu, J., X. Yang, H. Wu and J. Li	2021	Clinical Significance of Color Ultrasound, MRI, miR-21, and CA199 in the Diagnosis of Pancreatic Cancer	Journal of Oncology	Not early-stage
1174	Yu, P., S. Luo, J. Cai, J. Li and C. Peng	2022	ERAP2 as a potential biomarker for predicting gemcitabine response in patients with pancreatic cancer	Aging	Records excluded after screening
1175	Yu, S., P. Wang and Z. Chen	2019	Blood exosomal long RNA profiling identifies diagnostic and prognostic markers in pancreatic ductal adenocarcinoma	JNCCN Journal of the National Comprehensive Cancer Network	Records excluded after screening
1176	Yu, Y., C. Liang, Q. Q. Wan, D. Jin, X. Liu, Z. Zhang, Z. Y. Sun and G. J. Zhang	2023	Integrated FET sensing microsystem for specific detection of pancreatic cancer exosomal miRNA10b	Analytica Chimica Acta	Records excluded after screening
1177	Yu, Y., R. Lu and L. Guo	2022	Identification of serum microrna-25 as a novel biomarker for pancreatic cancer	Clinica Chimica Acta	Not early-stage
1178	Yu, Y., Y. Tong, A. Zhong, Y. Wang, R. Lu and L. Guo	2020	Identification of Serum microRNA-25 as a novel biomarker for pancreatic cancer	Medicine (United States)	Studies included in review
1179	Yu, Z., Y. Yang, W. Fang, P. Hu, Y. Liu and J. Shi	2023	Dual Tumor Exosome Biomarker Co-recognitions Based Nanoliquid Biopsy for the Accurate Early Diagnosis of Pancreatic Cancer	ACS nano	Records excluded after screening
1180	Yuan, C., J. Fang, X. Luo, Y. Zhang, G. Huang, X. Zeng, K. Xia, M. Li, X. Chen, X. Yang, M. L. de la Chapelle and W. Fu	2022	One-step isothermal amplification strategy for microRNA specific and ultrasensitive detection based on nicking-assisted entropy-driven DNA circuit triggered exponential amplification reaction	Analytica Chimica Acta	Records excluded after screening
1181	Yuan, F., Z. Li, L. Chen, T. Zeng, Y. H. Zhang, S. Ding, T. Huang and Y. D. Cai	2021	Identifying the Signatures and Rules of Circulating Extracellular MicroRNA for Distinguishing Cancer Subtypes	Frontiers in Genetics	Records excluded after screening
1182	Yuan, T. L., R. Bagni, M. Yi, A. Amzallag, S. Afghani, K. Beam, W. Burgan, N. Fer, L. Garvey, B. Smith, A. Waters, R. Stephens, C. Benes and F. McCormick	2015	Next-generation screen for integrative subtyping and target discovery for KRAS-mutant cancer	Cancer Research	Records excluded after screening
1183	Yue, Z., L. Pei, G. Meng, A. Zhang, M. Li, M. Jia, H. Wang and L. Cao	2023	Simultaneous Quantification of Serum Lipids and Their Association with Type 2 Diabetes Mellitus-Positive Hepatocellular Cancer	Metabolites	Records excluded after screening

1184	Zaidi, S. Y. and S. Froghi	2020	Novel Circulating Exosome Biomarkers Diagnostic of Pancreatic Cancer – A Systematic Review	European Journal of Surgical Oncology	Records excluded after screening
1185	Zardab, M., V. Balarajah, A. Banerjee, K. Stasinos, A. Saad, A. Imrali, C. Hughes, R. Roberts, A. Vajrala, C. Chelala, A. Z. M. Dayem Ullah and H. Kocher	2022	Development of a Model for Differentiating PDAC from Benign Pancreatic Conditions: A Prospective Case-control Study.		Records excluded after screening
1186	Zeichner, S. B., C. Stanislaw and J. L. Meisel	2016	Prevention and Screening in Hereditary Breast and Ovarian Cancer	Oncology (Williston Park)	Records excluded after screening
1187	Zeng, D., Z. Wang, Z. Meng, P. Wang, L. San, W. Wang, A. Aldalbahi, L. Li, J. Shen and X. Mi	2017	DNA Tetrahedral Nanostructure-Based Electrochemical miRNA Biosensor for Simultaneous Detection of Multiple miRNAs in Pancreatic Carcinoma	ACS applied materials & interfaces	Records excluded after screening
1188	Zeng, M. S.	2016	Noncoding RNAs in cancer diagnosis		Records excluded after screening
1189	Zeng, X., D. Ren, R. Liu, Q. Zhang, X. Yan and X. Yuan	2023	Oral microbiome-driven virulence factors: A novel approach to pancreatic cancer diagnosis	Biomolecules & biomedicine	Records excluded after screening
1190	Zhang, A. and H. Hu	2022	Development and validation of a novel circulating cell-free microRNA diagnostic model with high accuracy for multi-cancer early detection	Cancer Research	Records excluded after screening
1191	Zhang, A. and H. Hu	2022	A Novel Blood-Based microRNA Diagnostic Model with High Accuracy for Multi-Cancer Early Detection	Cancers	Records excluded after screening
1192	Zhang, B., Z. Chen, B. Tao, C. Yi, Z. Lin, Y. Li, W. Shao, J. Lin and J. Chen	2021	m6A target microRNAs in serum for cancer detection	Molecular Cancer	Records excluded after screening
1193	Zhang, C., M. Alhamdani, A. Bauer, L. Peng and J. Hoheisel	2020	The identification of biomarker for the earlydiagnosis of IPMN associated PDAC based on serumprotein profiling	Cancer Research	Records excluded after screening
1194	Zhang, H., H. Li, Q. Ma, F. Y. Yang and T. Y. Diao	2016	Predicting malignant transformation of esophageal squamous cell lesions by combined biomarkers in an endoscopic screening program	World Journal of Gastroenterology	Records excluded after screening
1195	Zhang, H. and J. Liu	2024	NF2 is a candidate diagnosis, prognostic, and immunotherapeutic biomarker: a systematic pan-cancer analysis	Translational Cancer Research	Records excluded after screening

1196	Zhang, H., L. Zhao, J. Jiang, J. Zheng, L. Yang, Y. Li, J. Zhou, T. Liu, J. Xu, W. Lou, W. Yang, L. Tan, W. Liu, Y. Yu, M. Ji, Y. Xu, Y. Lu, X. Li, Z. Liu, R. Tian, C. Hu, S. Zhang, Q. Hu, Y. Deng, H. Ying, S. Zhong, X. Zhang, Y. Wang, H. Wang, J. Bai, X. Li and X. Duan	2022	Multiplexed nanomaterial-assisted laser desorption/ionization for pan-cancer diagnosis and classification	Nature Communications	Non-diagnostic studies
1197	Zhang, J., Y. Zhang, Y. Li, S. Guo and G. Yang	2020	Identification of cancer biomarkers in human body fluids by using enhanced physicochemical-incorporated evolutionary conservation scheme	Current Topics in Medicinal Chemistry	Records excluded after screening
1198	Zhang, L., S. Sanagapalli and A. Stoita	2018	Challenges in diagnosis of pancreatic cancer	World Journal of Gastroenterology	Records excluded after screening
1199	Zhang, M., Y. Zhang, J. Fu and L. Zhang.	2019	Serum CA125 levels are decreased in rectal cancer but increased in fibrosis-associated diseases and in most types of cancers.		Records excluded after screening
1200	Zhang, P., M. Zou, X. Wen, F. Gu, J. Li, G. Liu, J. Dong, X. Deng, J. Gao, X. Li, X. Jia, Z. Dong, L. Chen, Y. Wang and Y. Tian	2014	Development of serum parameters panels for the early detection of pancreatic cancer	International Journal of Cancer	Studies included in review
1201	Zhang, Q., S. Chen, L. Zeng, Y. Chen, G. Lian, C. Qian, J. Li, R. Xie and K. H. Huang	2017	New developments in the early diagnosis of pancreatic cancer	Expert Review of Gastroenterology and Hepatology	Records excluded after screening
1202	Zhang, Q., M. Ye, C. Lin, M. Hu, Y. Wang, Y. Lou, Q. Kong, J. Zhang, J. Li, Y. Zhang, T. Yang, X. Sun, W. Yao, Y. Hua, H. Huang, M. Xu, X. Wang, X. Yu, W. Tao, R. Liu, Y. Gao, T. Wang, J. Wang, X. Wei, J. Wu, Z. Yu, C. Zhang, C. Yu, X. Bai and T. Liang	2023	Mass cytometry-based peripheral blood analysis as a novel tool for early detection of solid tumours: A multicentre study	Gut	No specific biomarkers provided
1203	Zhang, S., X. Wan, M. Lv, C. Li, Q. Chu and G. Wang	2022	TMEM92 acts as an immune-resistance and prognostic marker in pancreatic cancer from the perspective of predictive, preventive, and personalized medicine	EPMA Journal	Non-diagnostic studies
1204	Zhang, W. H., W. Q. Wang, X. Han, H. L. Gao, T. J. Li, S. S. Xu, S. Li, H. X. Xu, H. Li, L. Y. Ye, X. Lin, C. T. Wu, J. Long, X. J. Yu and L. Liu	2020	Advances on diagnostic biomarkers of pancreatic ductal adenocarcinoma: A systems biology perspective	Computational and Structural Biotechnology Journal	Records excluded after screening

1205	Zhang, X., G. Soori, T. J. Dobleman and G. G. Xiao	2014	The application of monoclonal antibodies in cancer diagnosis	Expert Review of Molecular Diagnostics	Records excluded after screening
1206	Zhang, Y., V. Chandra, E. Riquelme Sanchez, P. Dutta, P. R. Quesada, A. Rakoski, M. Zoltan, N. Arora, S. Baydogan, W. Horne, J. Burks, H. Xu, P. Hussain, H. Wang, S. Gupta, A. Maitra, J. M. Bailey, S. J. Moghaddam, S. Banerjee, I. Sahin, P. Bhattacharya and F. McAllister	2020	Interleukin-17-induced neutrophil extracellular traps mediate resistance to checkpoint blockade in pancreatic cancer	J Exp Med	Records excluded after screening
1207	Zhang, Y., L. Qiu, Y. Wang, X. Qin and Z. Li	2014	High-throughput and high-sensitivity quantitative analysis of serum unsaturated fatty acids by chip-based nanoelectrospray ionization-Fourier transform ion cyclotron resonance mass spectrometry: Early stage diagnostic biomarkers of pancreatic cancer	Analyst	Studies included in review
1208	Zhang, Y., Y. Wang, X. Su, P. Wang and W. Lin	2021	The Value of Circulating Circular RNA in Cancer Diagnosis, Monitoring, Prognosis, and Guiding Treatment	Frontiers in Oncology	Records excluded after screening
1209	Zhang, Y., J. Yang, H. Li, Y. Wu, H. Zhang and W. Chen	2015	Tumor markers CA19-9, CA242 and CEA in the diagnosis of pancreatic cancer: A meta-analysis	International Journal of Clinical and Experimental Medicine	Records excluded after screening
1210	Zhang, Z., W. Qin and Y. Sun	2018	Contribution of biomarkers for pancreatic cancer-associated new-onset diabetes to pancreatic cancer screening	Pathology Research and Practice	Records excluded after screening
1211	Zhang, Z., H. Wang, Q. Yan, J. Cui, Y. Chen, S. Ruan, J. Yang, Z. Wu, M. Han, S. Huang, Q. Zhou, C. Zhang and B. Hou	2023	Genome-wide CRISPR/Cas9 screening for drug resistance in tumors	Frontiers in Pharmacology	Records excluded after screening
1212	Zhang, Z. M., J. S. Wang, H. Zulfiqar, H. Lv, F. Y. Dao and H. Lin	2020	Early Diagnosis of Pancreatic Ductal Adenocarcinoma by Combining Relative Expression Orderings With Machine-Learning Method	Frontiers in Cell and Developmental Biology	No sample information provided
1213	Zhao, B., B. Zhao and F. Chen	2022	Diagnostic value of serum carbohydrate antigen 19-9 in pancreatic cancer: A systematic review and meta-analysis	European Journal of Gastroenterology and Hepatology	Records excluded after screening

1214	Zhao, G., R. Jiang, Y. Shi, S. Gao, D. Wang, Z. Li, Y. Zhou, J. Sun, W. Wu, J. Peng, T. Kuang, Y. Rong, J. Yuan, S. Zhu, G. Jin, Y. Wang and W. Lou	2024	Circulating cell-free DNA methylation-based multi-omics analysis allows early diagnosis of pancreatic ductal adenocarcinoma	Molecular Oncology	Records excluded after screening
1215	Zhao, J., Y. Liang, Q. Yin, S. Liu, Q. Wang, Y. Tang and C. Cao	2016	Clinical and prognostic significance of serum transforming growth factor-beta1 levels in patients with pancreatic ductal adenocarcinoma	Brazilian Journal of Medical and Biological Research	Non-diagnostic studies
1216	Zhao, Q. W., B. Situ and L. Zheng	2017	Current progress in research of circulating tumor cells	Nan fang yi ke da xue xue bao = Journal of Southern Medical University	Records excluded after screening
1217	Zhao, R., Z. Han, H. Zhou, Y. Xue, X. Chen and X. Cao	2023	Diagnostic and prognostic role of circRNAs in pancreatic cancer: a meta-analysis	Frontiers in Oncology	Records excluded after screening
1218	Zhao, S., Y. Li, J. Xu and L. Shen	2024	APOBEC3C is a novel target for the immune treatment of lower-grade gliomas	Neurol Res	Records excluded after screening
1219	Zhao, W., S. Yang, C. Li, F. Li, H. Pang, G. Xu, Y. Wang and M. Cong	2022	Amphiphilic Dendritic Nanomicelle-Mediated Delivery of Gemcitabine for Enhancing the Specificity and Effectiveness	International Journal of Nanomedicine	Records excluded after screening
1220	Zhao, X., M. Lu, Z. Liu, M. Zhang, H. Yuan, Z. Dan, D. Wang, B. Ma, Y. Yang, F. Yang, R. Sun, L. Li and C. Dang	2023	Comprehensive analysis of alfa defensin expression and prognosis in human colorectal cancer	Frontiers in Oncology	Records excluded after screening
1221	Zhao, Y., L. Zhao, H. Jin, Y. Xie, L. Chen, W. Zhang, L. Dong, L. Zhang, Y. Huang, K. Wan, Q. Yang and S. Wang	2024	Plasma methylated GNB4 and Riplet as a novel dual-marker panel for the detection of hepatocellular carcinoma	Epigenetics	Records excluded after screening
1222	Zheng, M., W. Wang, Y. Bu, J. Liu, J. Ma, R. Wang, X. Ren, Z. Lu, J. Li and J. Cai	2023	Pan-Cancer Analysis of the Roles and Driving Forces of RAB42	Biomolecules	Records excluded after screening
1223	Zheng, Y. S., M. L. Chen, W. D. Lei, S. L. Zhu, X. Q. You and Y. Liu	2020	NUDT21 knockdown inhibits proliferation and promotes apoptosis of pancreatic ductal adenocarcinoma through EIF2 signaling	Exp Cell Res	Records excluded after screening
1224	Zhong, N., Y. Z. Cui, X. Y. Zhou, T. L. Li and J. X. Han	2015	Identification of prohibitin 1 as a potential prognostic biomarker in human pancreatic carcinoma using modified aqueous two-phase partition system combined with 2D-MALDI-TOF-TOF-MS/MS	Tumor Biology	Records excluded after screening
1225	Zhou, B., J. W. Xu, Y. G. Cheng, J. Y. Gao, S. Y. Hu, L. Wang and H. X. Zhan	2017	Early detection of pancreatic cancer: Where are we now and where are we going?	International Journal of Cancer	Records excluded after screening

1226	Zhou, C. Y., Y. P. Dong, X. Sun, X. Sui, H. Zhu, Y. Q. Zhao, Y. Y. Zhang, C. Mason, Q. Zhu and S. X. Han	2018	High levels of serum glypican-1 indicate poor prognosis in pancreatic ductal adenocarcinoma	Cancer Med	Studies included in review
1227	Zhou, H., X. C. Wang, B. B. Yuan and B. B. Lu	2022	Clinical value of combining serum tumor marker detection with fecal occult blood testing in diagnosing colorectal cancer	J Physiol Pharmacol	Records excluded after screening
1228	Zhou, Q., R. Andersson, D. Hu, M. Bauden, A. Sasor, T. Bygott, K. PawŁowski, I. Pla, G. Marko-Varga and D. Ansari	2019	Alpha-1-acid glycoprotein 1 is upregulated in pancreatic ductal adenocarcinoma and confers a poor prognosis	Translational Research	Records excluded after screening
1229	Zhou, X., Z. Lu, T. Wang, Z. Huang, W. Zhu and Y. Miao	2018	Plasma miRNAs in diagnosis and prognosis of pancreatic cancer: A miRNA expression analysis	Gene	Records excluded after screening
1230	Zhou, Y., J. Cui and H. Du	2019	Autoantibody-targeted TAAs in pancreatic cancer: A comprehensive analysis	Pancreatology	Records excluded after screening
1231	Zhou, Y. F., L. X. Xu, L. T. Huang, F. Guo, F. Zhang, X. Y. He, W. Y. Yao and Y. Z. Yuan	2014	Combined detection of serum ULBP2 and MIC-1 improves early diagnosis and prognostic prediction of pancreatic cancer	Journal of Digestive Diseases	Not early-stage
1232	Zhou, Y. F., L. X. Xu, L. Y. Huang, F. Guo, F. Zhang, X. Y. He, Y. Z. Yuan and W. Y. Yao	2014	Combined detection of serum UL16-binding protein 2 and macrophage inhibitory cytokine-1 improves early diagnosis and prognostic prediction of pancreatic cancer	Oncology Letters	Records excluded after screening
1233	Zhu, J. and J. H. Strickler	2016	Clinical applications of liquid biopsies in gastrointestinal oncology	Journal of Gastrointestinal Oncology	Records excluded after screening
1234	Zhu, L., L. Zhao, Q. Wang, S. Zhong, X. Guo, Y. Zhu, J. Bao, K. Xu and S. Liu	2022	Circulating exosomal miRNAs and cancer early diagnosis	Clinical and Translational Oncology	Records excluded after screening
1235	Zhu, X. Y., Q. X. Li, Y. Kong, K. K. Huang, G. Wang, Y. J. Wang, J. Lu, G. Q. Hua, Y. L. Wu and T. L. Ying	2024	A novel human single-domain antibody-drug conjugate targeting CEACAM5 exhibits potent in vitro and in vivo antitumor activity	Acta Pharmacologica Sinica	Records excluded after screening
1236	Zhu, Y. X., C. H. Li, G. Li, H. Feng, T. Xia, C. H. Wong, F. K. C. Fung, J. H. Tong, K. F. To, R. Chen and Y. Chen	2020	LLGL1 Regulates Gemcitabine Resistance by Modulating the ERK-SP1-OSMR Pathway in Pancreatic Ductal Adenocarcinoma	Cell Mol Gastroenterol Hepatol	Records excluded after screening
1237	Zhuang, L., C. Huang, Z. Ning, L. Yang, W. Zou, P. Wang, C. S. Cheng and Z. Meng	2023	Circulating tumor-associated autoantibodies as novel diagnostic biomarkers in pancreatic adenocarcinoma	International Journal of Cancer	Not early-stage
1238	Ziogas, D. E., I. D. Kyrochristos, E. G. Lykoudis and D. H. Roukos	2018	Early solid tumor diagnosis through next-generation sequencing of cell-free DNA	Biomarkers in Medicine	Records excluded after screening

1239	Zöller, M., S. Yue, B. Madhavan, U. Galli, W. Gross, N. A. Giese, H. Kalthoff and M. W. Büchler	2014	Highly sensitive pancreatic cancer diagnosis by serum exosome stem cell and miRNA markers	European Journal of Cancer	Records excluded after screening
1240	Zou, X., J. Wei, Z. Huang, X. Zhou, Z. Lu, W. Zhu and Y. Miao	2019	Identification of a six-miRNA panel in serum benefiting pancreatic cancer diagnosis	Cancer Med	Not early-stage
1241		2017	New Biomarker Identified for PDAC	Cancer Discov	Duplicate records removed
1242	Aalami, A. H., H. Abdeahad, M. Mesgari and A. Sahebkar	2021	MicroRNA-223 in gastrointestinal cancers: A systematic review and diagnostic meta-analysis	Eur J Clin Invest	Duplicate records removed
1243	Abdallah, R., V. Taly, S. Zhao, D. Pietrasz, J. B. Bachet, D. Basile, L. Mas, A. Zaanan, P. Laurent-Puig and J. Taieb	2020	Plasma circulating tumor DNA in pancreatic adenocarcinoma for screening, diagnosis, prognosis, treatment and follow-up: A systematic review	Cancer Treat Rev	Duplicate records removed
1244	Abe, N., K. Matsuo, T. Kumasaka, K. Naka, S. Hashimoto, T. Takemura, M. Fujiwara, Y. Ito, R. Nakata, T. Hashimoto, M. Makuuchi, Y. Soejima and M. Sawabe	2016	Systematic cytological evaluation and immunocytochemistry of minichromosome maintenance protein 2 and p53 significantly improve cytological diagnosis of pancreaticobiliary adenocarcinoma	J Med Dent Sci	Duplicate records removed
1245	Abe, T., C. Koi, S. Kohi, K. B. Song, K. Tamura, A. Macgregor-Das, N. Kitaoka, M. Chuidian, M. Ford, M. Dbouk, M. Borges, J. He, R. Burkhart, C. L. Wolfgang, A. P. Klein, J. R. Eshleman, R. H. Hruban, M. I. Canto and M. Goggins	2020	Gene Variants That Affect Levels of Circulating Tumor Markers Increase Identification of Patients With Pancreatic Cancer	Clin Gastroenterol Hepatol	Duplicate records removed
1246	Ahmadipour, M., A. Bhattacharya, M. Sarafbidabad, E. Syuhada Sazali, S. Krishna Ghoshal, M. Satgunam, R. Singh, M. Rezaei Ardani, N. Missaoui, H. Kahri, U. Pal and A. Ling Pang	2024	CA19-9 and CEA biosensors in pancreatic cancer	Clin Chim Acta	Duplicate records removed
1247	Ali, S., M. Coory, P. Donovan, R. H. Na, N. Pandeya, S. A. Pearson, K. Spilsbury, K. Tuesley, S. J. Jordan and R. E. Neale	2024	Predicting the risk of pancreatic cancer in women with new-onset diabetes mellitus	Journal of Gastroenterology and Hepatology	Duplicate records removed

1248	Alizadeh Savareh, B., H. Asadzadeh Aghdaie, A. Behmanesh, A. Bashiri, A. Sadeghi, M. Zali and R. Shams	2020	A machine learning approach identified a diagnostic model for pancreatic cancer through using circulating microRNA signatures	Pancreatology	Duplicate records removed
1249	Ansari, D., A. Gustafsson and R. Andersson	2015	Update on the management of pancreatic cancer: surgery is not enough	World J Gastroenterol	Duplicate records removed
1250	Arasawa, T., T. Hiwasa, A. Kagaya, T. Maruyama, M. Uesato, M. Kano, S. Kobayashi, H. Takizawa, K. Iwase, F. Nomura, K. Matsushita and H. Matsubara	2023	Analysis of patients with colorectal cancer shows a specific increase in serum anti-ING1 autoantibody levels	BMC Cancer	Duplicate records removed
1251	Arasawa, T., T. Hiwasa, A. Kagaya, T. Maruyama, M. Uesato, M. Kano, S. Kobayashi, H. Takizawa, K. Iwase, F. Nomura, K. Matsushita and H. Matsubara	2023	Analysis of patients with colorectal cancer shows a specific increase in serum anti-ING1 autoantibody levels	BMC Cancer	Duplicate records removed
1252	Aronsson, L., R. Andersson, M. Bauden, B. Andersson, T. Bygott and D. Ansari	2018	High-density and targeted glycoproteomic profiling of serum proteins in pancreatic cancer and intraductal papillary mucinous neoplasm	Scandinavian Journal of Gastroenterology	Duplicate records removed
1253	Aronsson, L., R. Andersson, M. Bauden, B. Andersson, T. Bygott and D. Ansari	2018	High-density and targeted glycoproteomic profiling of serum proteins in pancreatic cancer and intraductal papillary mucinous neoplasm	Scand J Gastroenterol	Duplicate records removed
1254	Asada, T., S. Nakahata, Y. R. Fauzi, T. Ichikawa, K. Inoue, N. Shibata, Y. Fujii, N. Imamura, M. Hiyoshi, A. Nanashima and K. Morishita	2022	Integrin α6A (ITGA6A)-type Splice Variant in Extracellular Vesicles Has a Potential as a Novel Marker of the Early Recurrence of Pancreatic Cancer	Anticancer Research	Duplicate records removed
1255	Asada, T., S. Nakahata, Y. R. Fauzi, T. Ichikawa, K. Inoue, N. Shibata, Y. Fujii, N. Imamura, M. Hiyoshi, A. Nanashima and K. Morishita	2022	Integrin α6A (ITGA6A)-type Splice Variant in Extracellular Vesicles Has a Potential as a Novel Marker of the Early Recurrence of Pancreatic Cancer	Anticancer Res	Duplicate records removed
1256	Bahado-Singh, R. O., O. Turkoglu, B. Aydas and S. Vishweswaraiah	2023	Precision oncology: Artificial intelligence, circulating cell-free DNA, and the minimally invasive detection of pancreatic cancer-A pilot study	Cancer Medicine	Duplicate records removed

1257	Balasenthil, S., Y. Huang, S. Liu, T. Marsh, J. Chen, S. A. Stass, D. KuKuruga, R. Brand, N. Chen, M. L. Frazier, J. J. Lee, S. Srivastava, S. Sen and A. McNeill Killary	2017	A Plasma Biomarker Panel to Identify Surgically Resectable Early-Stage Pancreatic Cancer	Journal of the National Cancer Institute	Duplicate records removed
1258	Balasenthil, S., Y. Huang, S. Y. Liu, T. Marsh, J. Y. Chen, S. A. Stass, D. KuKuruga, R. Brand, N. Y. Chen, M. L. Frazier, J. J. Lee, S. Srivastava, S. Sen and A. M. Killary	2017	A Plasma Biomarker Panel to Identify Surgically Resectable Early-Stage Pancreatic Cancer	Jnci-Journal of the National Cancer Institute	Duplicate records removed
1259	Banaei, N., A. Foley, J. M. Houghton, Y. Sun and B. Kim	2017	Multiplex detection of pancreatic cancer biomarkers using a SERS-based immunoassay	Nanotechnology	Duplicate records removed
1260	Banaei, N., A. Foley, J. M. Houghton, Y. B. Sun and B. Kim	2017	Multiplex detection of pancreatic cancer biomarkers using a SERS-based immunoassay	Nanotechnology	Duplicate records removed
1261	Bantis, L. E. and J. V. Tsimikas	2022	On optimal biomarker cutoffs accounting for misclassification costs in diagnostic trilemmas with applications to pancreatic cancer	Stat Med	Duplicate records removed
1262	Bantis, L. E., Q. Yan, J. V. Tsimikas and Z. Feng	2017	Estimation of smooth ROC curves for biomarkers with limits of detection	Statistics in Medicine	Duplicate records removed
1263	Bantis, L. E., Q. Yan, J. V. Tsimikas and Z. Feng	2017	Estimation of smooth ROC curves for biomarkers with limits of detection	Stat Med	Duplicate records removed
1264	Bartsch, D. K., N. Gercke, K. Strauch, R. Wieboldt, E. Matthäi, V. Wagner, S. Rospleszcz, A. Schäfer, F. S. Franke, I. Mintziras, C. Bauer, T. Grote, J. Figiel, P. Di Fazio, A. Burchert, S. Reinartz, E. P. von Strandmann, G. Klöppel and E. P. Slater	2018	The combination of miRNA-196b, LCN2, and TIMP1 is a potential set of circulating biomarkers for screening individuals at risk for familial pancreatic cancer	Journal of Clinical Medicine	Duplicate records removed
1265	Bauden, M., D. Pamart, D. Ansari, M. Herzog, M. Eccleston, J. Micallef, B. Andersson and R. Andersson	2015	Circulating nucleosomes as epigenetic biomarkers in pancreatic cancer	Clinical Epigenetics	Duplicate records removed
1266	Benati, M., M. Montagnana, E. Danese, E. Paviati, S. Giudici, O. Ruzzenente, M. Franchi and G. Lippi	2018	The clinical significance of DJ-1 and HE4 in patients with endometrial cancer	Journal of Clinical Laboratory Analysis	Duplicate records removed

1267	Benati, M., M. Montagnana, E. Danese, E. Paviati, S. Giudici, O. Ruzzenente, M. Franchi and G. Lippi	2018	The clinical significance of DJ-1 and HE4 in patients with endometrial cancer	J Clin Lab Anal	Duplicate records removed
1268	Bhasin, M., A. Bullock, X. Gu, O. Bucur, R. Najarian, J. C. Haines, K. Ruping, R. Miksad, T. Libermann and R. Khosravi-Far	2014	Early detection of pancreatic cancer using a new 5-gene classifier	Cancer Research	Duplicate records removed
1269	Bhasin, M. K., K. Ndebele, O. Bucur, E. U. Yee, H. H. Otu, J. Plati, A. Bullock, X. Gu, E. Castan, P. Zhang, R. Najarian, M. S. Muraru, R. Miksad, R. Khosravi-Far and T. A. Libermann	2016	Meta-analysis of transcriptome data identifies a novel 5-gene pancreatic adenocarcinoma classifier	Oncotarget	Duplicate records removed
1270	Bhasin, M. K., K. Ndebele, O. Bucur, E. U. Yee, H. H. Otu, J. Plati, A. Bullock, X. S. Gu, E. Castan, P. Zhang, R. Najarian, M. S. Muraru, R. Miksad, R. Khosravi-Far and T. A. Libermann	2016	Meta-analysis of transcriptome data identifies a novel 5-gene pancreatic adenocarcinoma classifier	Oncotarget	Duplicate records removed
1271	Bian, F., L. Sun, L. Cai, Y. Wang, Y. Zhao, S. Wang and M. Zhou	2019	Molybdenum disulfide-integrated photonic barcodes for tumor markers screening	Biosens Bioelectron	Duplicate records removed
1272	Bian, F. J., L. Y. Sun, L. J. Cai, Y. Wang, Y. J. Zhao, S. Q. Wang and M. T. Zhou	2019	Molybdenum disulfide-integrated photonic barcodes for tumor markers screening	Biosensors & Bioelectronics	Duplicate records removed
1273	Blyuss, O., A. Zaikin, V. Cherepanova, D. Munblit, E. M. Kiseleva, O. M. Prytomanova, S. W. Duffy and T. Crnogorac-Jurcevic	2020	Development of PancRISK, a urine biomarker-based risk score for stratified screening of pancreatic cancer patients	British Journal of Cancer	Duplicate records removed
1274	Blyuss, O., A. Zaikin, V. Cherepanova, D. Munblit, E. M. Kiseleva, O. M. Prytomanova, S. W. Duffy and T. Crnogorac-Jurcevic	2020	Development of PancRISK, a urine biomarker-based risk score for stratified screening of pancreatic cancer patients	Br J Cancer	Duplicate records removed
1275	Brychta, N., T. Krahn and O. von Ahsen	2016	Detection of KRAS Mutations in Circulating Tumor DNA by Digital PCR in Early Stages of Pancreatic Cancer	Clin Chem	Duplicate records removed
1276	Brychta, N., T. Krahn and O. von Ahsenh	2016	Detection of <i>KRAS</i> Mutations in Circulating Tumor DNA by Digital PCR in Early Stages of Pancreatic Cancer	Clinical Chemistry	Duplicate records removed

1277	Bunganič, B., L. š t'ovičková, M. Tatarkovič, L. Kocourková, S. Šuchá nek, P. Frič, V. Setničska and M. Zavoral	2015	Molecular spectroscopy of blood plasma-towards the diagnostics of pancreatic cancer?	Gastroenterologi a y Hepatologia	Duplicate records removed
1278	Cao, F., A. Wei, X. Hu, Y. He, J. Zhang, L. Xia, K. Tu, J. Yuan, Z. Guo, H. Liu, D. Xie and A. Li	2020	Integrated epigenetic biomarkers in circulating cell-free DNA as a robust classifier for pancreatic cancer	Clin Epigenetics	Duplicate records removed
1279	Cao, F., A. L. Wei, X. L. Hu, Y. J. He, J. Zhang, L. Xia, K. L. Tu, J. Yuan, Z. H. Guo, H. Y. Liu, D. Xie and A. Li	2020	Integrated epigenetic biomarkers in circulating cell-free DNA as a robust classifier for pancreatic cancer	Clinical Epigenetics	Duplicate records removed
1280	Caputo, D. and G. Caracciolo	2020	Nanoparticle-enabled blood tests for early detection of pancreatic ductal adenocarcinoma	Cancer Lett	Duplicate records removed
1281	Caputo, D., E. Quagliarini, A. Coppola, V. La Vaccara, B. Marmiroli, B. Sartori, G. Caracciolo and D. Pozzi	2023	Inflammatory biomarkers and nanotechnology: new insights in pancreatic cancer early detection	International Journal of Surgery	Duplicate records removed
1282	Caputo, D., E. Quagliarini, A. Coppola, V. La Vaccara, B. Marmiroli, B. Sartori, G. Caracciolo and D. Pozzi	2023	Inflammatory biomarkers and nanotechnology: new insights in pancreatic cancer early detection	Int J Surg	Duplicate records removed
1283	Carr, R. A., M. T. Yip-Schneider, S. Dolejs, B. A. Hancock, H. B. Wu, M. Radovich and C. M. Schmidt	2017	Pancreatic Cyst Fluid Vascular Endothelial Growth Factor A and Carcinoembryonic Antigen: A Highly Accurate Test for the Diagnosis of Serous Cystic Neoplasm	Journal of the American College of Surgeons	Duplicate records removed
1284	Carr, R. A., M. T. Yip-Schneider, R. E. Simpson, S. Dolejs, J. G. Schneider, H. Wu, E. P. Ceppa, W. Park and C. M. Schmidt	2018	Pancreatic cyst fluid glucose: rapid, inexpensive, and accurate diagnosis of mucinous pancreatic cysts	Surgery	Duplicate records removed
1285	Carter, J. V., H. L. Roberts, J. Pan, J. D. Rice, J. F. Burton, N. J. Galbraith, M. R. Eichenberger, J. Jorden, P. Deveaux, R. Farmer, A. Williford, Z. Kanaan, S. N. Rai and S. Galandiuk	2016	A Highly Predictive Model for Diagnosis of Colorectal Neoplasms Using Plasma MicroRNA: Improving Specificity and Sensitivity	Ann Surg	Duplicate records removed

1286	Carter, J. V., H. L. Roberts, J. M. Pan, J. D. Rice, J. F. Burton, N. J. Galbraith, M. R. Eichenberger, J. Jorden, P. Deveaux, R. Farmer, A. Williford, Z. Kanaan, S. N. Rai and S. Galandiuk	2016	A Highly Predictive Model for Diagnosis of Colorectal Neoplasms Using Plasma MicroRNA: Improving Specificity and Sensitivity	Annals of Surgery	Duplicate records removed
1287	Champanhac, C., I. T. Teng, S. Cansiz, L. Zhang, X. Wu, Z. Zhoa, T. Fu and W. Tan	2015	Development of a panel of DNA Aptamers with High Affinity for Pancreatic Ductal Adenocarcinoma	Sci Rep	Duplicate records removed
1288	Champanhac, C., I. T. Teng, S. Cansiz, L. Q. Zhang, X. Q. Wu, Z. L. Zhoa, T. Fu and W. H. Tan	2015	Development of a panel of DNA Aptamers with High Affinity for Pancreatic Ductal Adenocarcinoma	Scientific Reports	Duplicate records removed
1289	Chang, J. C. and M. Kundranda	2017	Novel Diagnostic and Predictive Biomarkers in Pancreatic Adenocarcinoma	Int J Mol Sci	Duplicate records removed
1290	Chen, J., H. Li, W. Xu and X. Guo	2021	Evaluation of serum ATX and LPA as potential diagnostic biomarkers in patients with pancreatic cancer	BMC Gastroenterol	Duplicate records removed
1291	Chen, J., H. Y. Li, W. D. Xu and X. Z. Guo	2021	Evaluation of serum ATX and LPA as potential diagnostic biomarkers in patients with pancreatic cancer	Bmc Gastroenterology	Duplicate records removed
1292	Chen, J., H. Wang, L. Zhou, Z. Liu and X. Tan	2022	A combination of circulating tumor cells and CA199 improves the diagnosis of pancreatic cancer	J Clin Lab Anal	Duplicate records removed
1293	Chen, J. L., H. T. Wang, L. Zhou, Z. H. Liu and X. D. Tan	2022	A combination of circulating tumor cells and CA199 improves the diagnosis of pancreatic cancer	Journal of Clinical Laboratory Analysis	Duplicate records removed
1294	Chen, Q., D. R. Cherry, V. Nalawade, E. M. Qiao, A. Kumar, A. M. Lowy, D. R. Simpson and J. D. Murphy	2021	Clinical Data Prediction Model to Identify Patients With Early-Stage Pancreatic Cancer	JCO clinical cancer informatics	Duplicate records removed
1295	Chen, Q., D. R. Cherry, V. Nalawade, E. M. Qiao, A. Kumar, A. M. Lowy, D. R. Simpson and J. D. Murphy	2021	Clinical Data Prediction Model to Identify Patients With Early-Stage Pancreatic Cancer	JCO Clin Cancer Inform	Duplicate records removed
1296	Chen, Q., D. Yu, Y. Zhao, J. Qiu, Y. Xie and M. Tao	2019	Screening and identification of hub genes in pancreatic cancer by integrated bioinformatics analysis	J Cell Biochem	Duplicate records removed
1297	Chen, Q., D. M. Yu, Y. Y. Zhao, J. J. Qiu, Y. F. Xie and M. Tao	2019	Screening and identification of hub genes in pancreatic cancer by integrated bioinformatics analysis	Journal of Cellular Biochemistry	Duplicate records removed

1298	Chen, W., Y. Zhou, V. Asadpour, R. A. Parker, E. Lustigova, E. J. Puttock and B. U. Wu (2022). Quantitative Radiomic Features from Computed Tomography Can Predict Pancreatic Cancer up to 36 Months Before Diagnosis.	2022			Duplicate records removed
1299	Chen, W., Y. Zhou, V. Asadpour, R. A. Parker, E. J. Puttock, E. Lustigova and B. U. Wu	2023	Quantitative Radiomic Features From Computed Tomography Can Predict Pancreatic Cancer up to 36 Months Before Diagnosis	Clinical and translational gastroenterology	Duplicate records removed
1300	Chen, W., Y. Zhou, V. Asadpour, R. A. Parker, E. J. Puttock, E. Lustigova and B. U. Wu	2023	Quantitative Radiomic Features From Computed Tomography Can Predict Pancreatic Cancer up to 36 Months Before Diagnosis	Clin Transl Gastroenterol	Duplicate records removed
1301	Chen, W. C., B. Boursi, R. Mamtani and Y. X. Yang	2019	Total Serum Cholesterol and Pancreatic Cancer: A Nested Case-Control Study	Cancer Epidemiol Biomarkers Prev	Duplicate records removed
1302	Chen, W. C., B. Boursi and Y. X. Yang	2017	Total serum cholesterol and pancreatic cancer: A nested case-control study	Gastroenterology	Duplicate records removed
1303	Chen, W. C. Y., B. Boursi, R. Mamtani and Y. X. Yang	2019	Total serum cholesterol and pancreatic cancer: A nested case-control study	Cancer Epidemiology Biomarkers and Prevention	Duplicate records removed
1304	Chen, Y. Z., D. Liu, Y. X. Zhao, H. T. Wang, Y. Gao and Y. Chen	2014	Diagnostic performance of serum macrophage inhibitory cytokine-1 in pancreatic cancer: a meta-analysis and meta-regression analysis	DNA Cell Biol	Duplicate records removed
1305	Chew, C., E. Cheow, C. Shing Leng, E. Chow, A. Kow, S. Iyer, K. Madhavan, C. Cheng Ean, L. Ho and G. Bonney	2022	A Precision Cilinical-omics approach in predicting therapeutic response in pancreatic cancer	Journal of Hepato-Biliary-Pancreatic Sciences	Duplicate records removed
1306	Chhoda, A., A. Sharma, B. Sailo, H. Tang, N. Ruzgar, W. Y. Tan, L. Ying, R. Khatri, A. Narayanan, S. Mane, B. De Kumar, L. D. Wood, C. Iacobuzio-Donahue, C. L. Wolfgang, J. W. Kunstman, R. R. Salem, J. J. Farrell and N. Ahuja	2023	Utility of promoter hypermethylation in malignant risk stratification of intraductal papillary mucinous neoplasms	Clin Epigenetics	Duplicate records removed

1307	Chhoda, A., A. Sharma, B. Sailo, H. Y. Tang, N. Ruzgar, W. Y. Tan, L. Ying, R. Khatri, A. Narayanan, S. Mane, B. De Kumar, L. D. Wood, C. Iacobuzio-Donahue, C. L. Wolfgang, J. W. Kunstman, R. R. Salem, J. J. Farrell and N. Ahuja	2023	Utility of promoter hypermethylation in malignant risk stratification of intraductal papillary mucinous neoplasms	Clinical Epigenetics	Duplicate records removed
1308	Choi, M., M. Park, S. H. Lee, M. J. Lee, Y. K. Paik, S. I. Jang, D. K. Lee, S. G. Lee and C. M. Kang	2023	Development of a metabolite calculator for diagnosis of pancreatic cancer	Cancer Medicine	Duplicate records removed
1309	Choi, M., M. Park, S. H. Lee, M. J. Lee, Y. K. Paik, S. I. Jang, D. K. Lee, S. G. Lee and C. M. Kang	2023	Development of a metabolite calculator for diagnosis of pancreatic cancer	Cancer Med	Duplicate records removed
1310	Chung, K. H., J. C. Lee, J. Lee, I. K. Cho, J. Kim, W. Jang, B. C. Yoo and J. H. Hwang	2020	Serum fibrinogen as a diagnostic and prognostic biomarker for pancreatic ductal adenocarcinoma	Pancreatology	Duplicate records removed
1311	Cohen, J. D., L. Li, Y. Wang, C. Thoburn, B. Afsari, L. Danilova, C. Douville, A. A. Javed, F. Wong, A. Mattox, R. H. Hruban, C. L. Wolfgang, M. G. Goggins, M. D. Molin, T. L. Wang, R. Roden, A. P. Klein, J. Ptak, L. Dobbyn, J. Schaefer, N. Silliman, M. Popoli, J. T. Vogelstein, J. D. Browne, R. E. Schoen, R. E. Brand, J. Tie, P. Gibbs, H. L. Wong, A. S. Mansfield, J. Jen, S. M. Hanash, M. Falconi, P. J. Allen, S. Zhou, C. Bettegowda, L. A. Diaz, C. Tomasetti, K. W. Kinzler, B. Vogelstein, A. M. Lennon and N. Papadopoulos	2018	Detection and localization of surgically resectable cancers with a multi-analyte blood test	Science	Duplicate records removed

1312	Cruz-Monserrate, Z., K. Gumpper, V. Pita, P. A. Hart, C. Forsmark, D. C. Whitcomb, D. Yadav, R. T. Waldron, S. Pandol, H. Steen, V. Anani, N. Kanwar, S. S. Vege, S. Appana, L. Li, J. Serrano, J. A. S. Rinaudo, M. Topazian and D. L. Conwell	2021	Biomarkers of Chronic Pancreatitis: A systematic literature review	Pancreatology	Duplicate records removed
1313	Dbouk, M., T. Abe, C. Koi, Y. Ando, H. Saba, E. A. Diwan, A. MacGregor-Das, A. L. Blackford, E. Mocci, K. Beierl, A. Dbouk, J. He, R. Burkhart, A. M. Lennon, L. Sokoll, M. I. Canto, J. R. Eshleman and M. Goggins	2023	Diagnostic Performance of a Tumor Marker Gene Test to Personalize Serum CA19–9 Reference Ranges	Clinical Cancer Research	Duplicate records removed
1314	Deben, C., L. F. Boullosa, F. R. Fortes, E. C. De La Hoz, M. Le Compte, S. Seghers, M. Peeters, S. Vanlanduit, A. Lin, K. K. Dijkstra, P. Van Schil, J. M. H. Hendriks, H. Prenen, G. Roeyen, F. Lardon and E. Smits	2024	Auranofin repurposing for lung and pancreatic cancer: low CA12 expression as a marker of sensitivity in patient-derived organoids, with potentiated efficacy by AKT inhibition	J Exp Clin Cancer Res	Duplicate records removed
1315	Deben, C., L. F. Boullosa, F. R. Fortes, E. C. de la Hoz, M. Le Compte, S. Seghers, M. Peeters, S. Vanlanduit, A. B. H. Lin, K. K. Dijkstra, P. Van Schil, J. M. H. Hendriks, H. Prenen, G. Roeyen, F. Lardon and E. Smits	2024	Auranofin repurposing for lung and pancreatic cancer: low CA12 expression as a marker of sensitivity in patient-derived organoids, with potentiated efficacy by AKT inhibition	Journal of Experimental & Clinical Cancer Research	Duplicate records removed
1316	Debernardi, S., O. Blyuss, D. Rycyk, K. Srivastava, C. Y. Jeon, H. Cai, Q. Cai, X. O. Shu and T. Crnogorac-Jurcevic	2023	Urine biomarkers enable pancreatic cancer detection up to 2 years before diagnosis	Int J Cancer	Duplicate records removed
1317	Debernardi, S., O. Blyuss, D. Rycyk, K. Srivastava, C. Y. Jeon, H. Cai, Q. Y. Cai, X. O. Shu and T. Crnogorac-Jurcevic	2023	Urine biomarkers enable pancreatic cancer detection up to 2 years before diagnosis	International Journal of Cancer	Duplicate records removed

1318	Debernardi, S., H. O'Brien, A. S. Algahmdi, N. Malats, G. D. Stewart, M. Pljesa-Ercegovac, E. Costello, W. Greenhalf, A. Saad, R. Roberts, A. Ney, S. P. Pereira, H. M. Kocher, S. Duffy, O. Blyuss and T. Crnogorac-Jurcevic	2020	A combination of urinary biomarker panel and PancRISK score for earlier detection of pancreatic cancer: A case-control study	Plos Medicine	Duplicate records removed
1319	Debernardi, S., H. O'Brien, A. S. Algahmdi, N. Malats, G. D. Stewart, M. Plješa-Ercegovac, E. Costello, W. Greenhalf, A. Saad, R. Roberts, A. Ney, S. P. Pereira, H. M. Kocher, S. Duffy, O. Blyuss and T. Crnogorac-Jurcevic	2020	A combination of urinary biomarker panel and PancRISK score for earlier detection of pancreatic cancer: A case-control study	PLoS Med	Duplicate records removed
1320	Demyan, L., A. N. Habowski, D. Plenker, D. A. King, O. J. Standring, C. Tsang, L. St Surin, A. Rishi, J. M. Crawford, J. Boyd, S. A. Pasha, H. Patel, Z. Galluzzo, C. Metz, P. K. Gregersen, S. Fox, C. Valente, S. Abadali, S. Matadial-Ragoo, D. K. DePeralta, G. B. Deutsch, J. M. Herman, M. A. Talamini, D. A. Tuveson and M. J. Weiss	2022	Pancreatic Cancer Patient-derived Organoids Can Predict Response to Neoadjuvant Chemotherapy	Ann Surg	Duplicate records removed
1321	Demyan, L., A. N. Habowski, D. Plenker, D. A. King, O. J. Standring, C. Tsang, L. St. Surin, A. Rishi, J. M. Crawford, J. Boyd, S. A. Pasha, H. Patel, Z. Galluzzo, C. Metz, P. K. Gregersen, S. Fox, C. Valente, S. Abadali, S. Matadial-Ragoo, D. K. Deperalta, G. B. Deutsch, J. M. Herman, M. A. Talamini, D. A. Tuveson and M. J. Weiss	2022	Pancreatic Cancer Patient-derived Organoids Can Predict Response to Neoadjuvant Chemotherapy	Annals of Surgery	Duplicate records removed

1322	Deng, T., Y. Yuan, C. Zhang, C. Zhang, W. Yao, C. Wang, R. Liu and Y. Ba	2016	Identification of Circulating MiR-25 as a Potential Biomarker for Pancreatic Cancer Diagnosis	Cell Physiol Biochem	Duplicate records removed
1323	Deutsch, O., Y. Haviv, G. Krief, N. Keshet, R. Westreich, S. M. Stemmer, B. Zaks, S. P. Navat, R. Yanko, O. Lahav, D. J. Aframian and A. Palmon	2020	Possible proteomic biomarkers for the detection of pancreatic cancer in oral fluids	Scientific reports	Duplicate records removed
1324	Deutsch, O., Y. Haviv, G. Krief, N. Keshet, R. Westreich, S. M. Stemmer, B. Zaks, S. P. Navat, R. Yanko, O. Lahav, D. J. Aframian and A. Palmon	2020	Possible proteomic biomarkers for the detection of pancreatic cancer in oral fluids	Sci Rep	Duplicate records removed
1325	Dhasmana, A., S. Dhasmana, S. Kotnala, P. Laskar, S. Khan, S. Haque, M. Jaggi, M. M. Yallapu and S. C. Chauhan	2024	CEACAM7 expression contributes to early events of pancreatic cancer	Journal of advanced research	Duplicate records removed
1326	Dhasmana, A., S. Dhasmana, S. Kotnala, P. Laskar, S. Khan, S. Haque, M. Jaggi, M. M. Yallapu and S. C. Chauhan	2024	CEACAM7 expression contributes to early events of pancreatic cancer	J Adv Res	Duplicate records removed
1327	Dhayat, S. A. and Z. Yang	2020	Impact of circulating tumor DNA in hepatocellular and pancreatic carcinomas	J Cancer Res Clin Oncol	Duplicate records removed
1328	Digiacomo, L., E. Quagliarini, D. Pozzi, R. Coppola, G. Caracciolo and D. Caputo	2023	Stratifying Risk for Pancreatic Cancer by Multiplexed Blood Test	Cancers	Duplicate records removed
1329	Dittmar, R. L., S. Liu, M. C. Tai, K. Rajapakshe, Y. Huang, G. Longton, C. DeCapite, M. W. Hurd, P. L. Paris, K. S. Kirkwood, C. Coarfa, A. Maitra, R. E. Brand, A. M. Killary and S. Sen	2021	Plasma miRNA Biomarkers in Limited Volume Samples for Detection of Early-stage Pancreatic Cancer	Cancer Prev Res (Phila)	Duplicate records removed
1330	Dittmar, R. L., S. Y. Liu, M. C. Tai, K. Rajapakshe, Y. Huang, G. Longton, C. DeCapite, M. W. Hurd, P. L. Paris, K. S. Kirkwood, C. Coarfa, A. Maitra, R. E. Brand, A. M. Killary and S. Sen	2021	Plasma miRNA Biomarkers in Limited Volume Samples for Detection of Early-stage Pancreatic Cancer	Cancer Prevention Research	Duplicate records removed

1331	Douville, C., K. Lahouel, A. Kuo, H. Grant, B. E. Avigdor, S. D. Curtis, M. Summers, J. D. Cohen, Y. Wang, A. Mattox, J. Dudley, L. Dobbyn, M. Popoli, J. Ptak, N. Nehme, N. Silliman, C. Blair, K. Romans, C. Thoburn, J. Gizzi, R. E. Schoen, J. Tie, P. Gibbs, L. T. Ho-Pham, B. N. H. Tran, T. S. Tran, T. V. Nguyen, M. Goggins, C. L. Wolfgang, T. L. Wang, I. M. Shih, A. M. Lennon, R. H. Hruban, C. Bettegowda, K. W. Kinzler, N. Papadopoulos, B. Vogelstein and C. Tomasetti	2024	Machine learning to detect the SINEs of cancer	Sci Transl Med	Duplicate records removed
1332	Draus, T., D. Ansari and R. Andersson	2023	Model-based screening for pancreatic cancer in Sweden	Scandinavian Journal of Gastroenterology	Duplicate records removed
1333	Draus, T., D. Ansari and R. Andersson	2023	Model-based screening for pancreatic cancer in Sweden	Scand J Gastroenterol	Duplicate records removed
1334	Duan, B., X. Hu, M. Fan, X. Xiong, L. Han, Z. Wang, D. Tong, L. Liu, X. Wang, W. Li, J. Yang and C. Huang	2019	RNA-Binding Motif Protein 6 is a Candidate Serum Biomarker for Pancreatic Cancer	Proteomics Clin Appl	Duplicate records removed
1335	Duan, B. J., X. Y. Hu, M. Y. Fan, X. F. Xiong, L. Han, Z. Wang, D. D. Tong, L. Y. Liu, X. F. Wang, W. S. Li, J. Yang and C. Huang	2019	RNA-Binding Motif Protein 6 is a Candidate Serum Biomarker for Pancreatic Cancer	Proteomics Clinical Applications	Duplicate records removed
1336	Duarte-Medrano, G., I. Lopez-Méndez, M. Ramírez-Luna, F. Valdovinos-Andraca, R. Cruz-Martínez, I. Medina-Vera, C. Pérez-Monter and F. I. Téllez-Ávila	2019	Analysis of circulating blood and tissue biopsy PDX1 and MSX2 gene expression in patients with pancreatic cancer: A case-control experimental study	Medicine (Baltimore)	Duplicate records removed
1337	Duarte-Medrano, G., I. Lopez-Méndez, M. A. Ramírez-Luna, F. Valdovinos-Andraca, R. Cruz-Martínez, I. Medina-Vera, C. Pérez-Monter and F. I. Téllez-Avila	2019	Analysis of circulating blood and tissue biopsy <i>PDX1</i> and <i>MSX2</i> gene expression in patients with pancreatic cancer A case-control experimental study	Medicine	Duplicate records removed

1338	Dumitrescu, R. G. (2018). Early epigenetic markers for precision medicine. 1856: 3-17.	2018			Duplicate records removed
1339	Dumstrei, K., H. Chen and H. Brenner	2016	A systematic review of serum autoantibodies as biomarkers for pancreatic cancer detection	Oncotarget	Duplicate records removed
1340	Dumstrei, K., H. D. Chen and H. Brenner	2016	A systematic review of serum autoantibodies as biomarkers for pancreatic cancer detection	Oncotarget	Duplicate records removed
1341	Eissa, M. A. L., L. Lerner, E. Abdelfatah, N. Shankar, J. K. Canner, N. M. Hasan, V. Yaghoobi, B. Huang, Z. Kerner, F. Takaesu, C. Wolfgang, R. Kwak, M. Ruiz, M. Tam, T. R. Pisanic, C. A. Iacobuzio-Donahue, R. H. Hruban, J. He, T. H. Wang, L. D. Wood, A. Sharma and N. Ahuja	2019	Promoter methylation of ADAMTS1 and BNC1 as potential biomarkers for early detection of pancreatic cancer in blood	Clinical Epigenetics	Duplicate records removed
1342	Eissa, M. A. L., L. Lerner, E. Abdelfatah, N. Shankar, J. K. Canner, N. M. Hasan, V. Yaghoobi, B. Huang, Z. Kerner, F. Takaesu, C. Wolfgang, R. Kwak, M. Ruiz, M. Tam, T. R. Pisanic, 2nd, C. A. Iacobuzio-Donahue, R. H. Hruban, J. He, T. H. Wang, L. D. Wood, A. Sharma and N. Ahuja	2019	Promoter methylation of ADAMTS1 and BNC1 as potential biomarkers for early detection of pancreatic cancer in blood	Clin Epigenetics	Duplicate records removed
1343	El-Jurdi, N. H. and M. W. Saif	2014	Pancreatic cancer: new hopes for early detection and a future screening tool?	Jop	Duplicate records removed
1344	Esposito, I., B. Konukiewitz, A. M. Schlitter and G. Klöppel	2014	Pathology of pancreatic ductal adenocarcinoma: facts, challenges and future developments	World J Gastroenterol	Duplicate records removed

1345	Fahrmann, J. F., C. M. Schmidt, X. Mao, E. Irajizad, M. Loftus, J. Zhang, N. Patel, J. Vykoukal, J. B. Dennison, J. P. Long, K. A. Do, J. Zhang, J. A. Chabot, M. D. Kluger, F. Kastrinos, L. Brais, A. Babic, K. Jajoo, L. S. Lee, T. E. Clancy, K. Ng, A. Bullock, J. Genkinger, M. T. Yip-Schneider, A. Maitra, B. M. Wolpin and S. Hanash	2021	Lead-Time Trajectory of CA19-9 as an Anchor Marker for Pancreatic Cancer Early Detection	Gastroenterology	Duplicate records removed
1346	Fahrmann, J. F., C. M. Schmidt, X. Mao, E. Irajizad, M. Loftus, J. Zhang, N. Patel, J. Vykoukal, J. B. Dennison, J. P. Long and et al.	2021	Lead-Time Trajectory of CA19-9 as an Anchor Marker for Pancreatic Cancer Early Detection	Gastroenterology	Duplicate records removed
1347	Fahrmann, J. F., C. M. Schmidt, X. Y. Mao, E. Irajizad, M. Loftus, J. M. Zhang, N. Patel, J. Vykoukal, J. B. Dennison, J. P. Long, K. A. Do, J. J. Zhang, J. A. Chabot, M. D. Kluger, F. Kastrinos, L. Brais, A. Babic, K. Jajoo, L. S. Lee, T. E. Clancy, K. Ng, A. Bullock, J. Genkinger, M. T. Yip-Schneider, A. Maitra, B. M. Wolpin and S. Hanash	2021	Lead-Time Trajectory of CA19-9 as an Anchor Marker for Pancreatic Cancer Early Detection	Gastroenterology	Duplicate records removed
1348	Faleiro, I., V. P. Roberto, S. D. Canli, N. A. Fraunhoffer, J. Iovanna, A. O. Gure, W. Link and P. Castelo-Branco	2021	Dna methylation of pi3k/akt pathway-related genes predicts outcome in patients with pancreatic cancer: A comprehensive bioinformatics-based study	Cancers	Duplicate records removed
1349	Farina, A., J. M. Dumonceau, P. Antinori, I. Annessi-Ramseyer, J. L. Frossard, D. F. Hochstrasser, M. Delhaye and P. Lescuyer	2014	Bile carcinoembryonic cell adhesion molecule 6 (CEAM6) as a biomarker of malignant biliary stenoses	Biochimica et Biophysica Acta - Proteins and Proteomics	Duplicate records removed
1350	Farina, A., J. M. Dumonceau, P. Antinori, I. Annessi-Ramseyer, J. L. Frossard, D. F. Hochstrasser, M. Delhaye and P. Lescuyer	2014	Bile carcinoembryonic cell adhesion molecule 6 (CEAM6) as a biomarker of malignant biliary stenoses	Biochim Biophys Acta	Duplicate records removed

1351	Felix, K., K. Honda, K. Nagashima, A. Kashiro, K. Takeuchi, T. Kobayashi, S. Hinterkopf, M. M. Gaida, H. Dang, N. Brindl, J. Kaiser, M. W. Büchler and O. Strobel	2022	Noninvasive risk stratification of intraductal papillary mucinous neoplasia with malignant potential by serum apolipoprotein-A2-isoforms	Int J Cancer	Duplicate records removed
1352	Fiala, C. and E. P. Diamandis	2020	Can a Broad Molecular Screen Based on Circulating Tumor DNA Aid in Early Cancer Detection?	The journal of applied laboratory medicine	Duplicate records removed
1353	Fiala, C. and E. P. Diamandis	2020	Can a Broad Molecular Screen Based on Circulating Tumor DNA Aid in Early Cancer Detection?	J Appl Lab Med	Duplicate records removed
1354	Filiputti, V. B., M. L. A. Cirino, F. S. L. Neto, P. C. Novais, L. P. Turra, M. F. G. S. Tazima, F. M. Peria, D. P. C. Tirapelli, R. Kemp, J. S. Dos Santos and A. K. Sankarankutty	2020	Combination of microrna-21 expression with the serum marker ca19-9 increases the accuracy of diagnosis of pancreatic adenocarcinoma	Genetics and Molecular Research	Duplicate records removed
1355	Firpo, M. A., K. M. Boucher and S. J. Mulvihill	2014	Prospects for developing an accurate diagnostic biomarker panel for low prevalence cancers	Theoretical biology & medical modelling	Duplicate records removed
1356	Firpo, M. A., K. M. Boucher and S. J. Mulvihill	2014	Prospects for developing an accurate diagnostic biomarker panel for low prevalence cancers	Theor Biol Med Model	Duplicate records removed
1357	Franses, J. W., O. Basar, A. Kadayifci, O. Yuksel, M. Choz, A. S. Kulkarni, E. Tai, K. D. Vo, K. S. Arora, N. Desai, J. A. Licausi, M. Toner, S. Maheswaran, D. A. Haber, D. P. Ryan, W. R. Brugge and D. T. Ting	2018	Improved Detection of Circulating Epithelial Cells in Patients with Intraductal Papillary Mucinous Neoplasms	Oncologist	Duplicate records removed
1358	Franses, J. W., O. Basar, A. Kadayifci, O. Yuksel, M. Choz, A. S. Kulkarni, E. Tai, K. D. Vo, K. S. Arora, N. Desai, J. A. Licausi, M. Toner, S. Maheswaran, D. A. Haber, D. P. Ryan, W. R. Brugge and D. T. Ting	2018	Improved Detection of Circulating Epithelial Cells in Patients with Intraductal Papillary Mucinous Neoplasms	Oncologist	Duplicate records removed

1359	Fujimoto, Y., Y. Suehiro, S. Kaino, S. Suenaga, T. Tsuyama, H. Matsui, S. Higaki, I. Fujii, C. Suzuki, T. Hoshida, T. Matsumoto, T. Takami, H. Nagano, I. Sakaida and T. Yamasaki	2021	Combination of CA19-9 and Blood Free-Circulating Methylated <i>RUNX3</i> May Be Useful to Diagnose Stage I Pancreatic Cancer	Oncology	Duplicate records removed
1360	Fujimoto, Y., Y. Suehiro, S. Kaino, S. Suenaga, T. Tsuyama, H. Matsui, S. Higaki, I. Fujii, C. Suzuki, T. Hoshida, T. Matsumoto, T. Takami, H. Nagano, I. Sakaida and T. Yamasaki	2021	Combination of CA19-9 and Blood Free-Circulating Methylated RUNX3 May Be Useful to Diagnose Stage i Pancreatic Cancer	Oncology (Switzerland)	Duplicate records removed
1361	Gao, G., C. Liu, S. Jain, D. Li, H. Wang, Y. Zhao and J. Liu	2019	Potential use of aptamers for diagnosis and treatment of pancreatic cancer	J Drug Target	Duplicate records removed
1362	Ge, L., B. Pan, F. Song, J. Ma, D. Zeraatkar, J. Zhou and J. Tian	2017	Comparing the diagnostic accuracy of five common tumour biomarkers and CA19-9 for pancreatic cancer: a protocol for a network meta-analysis of diagnostic test accuracy	BMJ Open	Duplicate records removed
1363	Ge, L., B. Pan, F. J. Song, J. C. Ma, D. Zeraatkar, J. G. Zhou and J. H. Tian	2017	Comparing the diagnostic accuracy of five common tumour biomarkers and CA19-9 for pancreatic cancer: a protocol for a network meta-analysis of diagnostic test accuracy	Bmj Open	Duplicate records removed
1364	Ge, W., Y. Wang, M. Quan, T. Mao, E. Y. Bischof, H. Xu, X. Zhang, S. Li, M. Yue, J. Ma, H. Yang, L. Wang, Z. Yu, L. Wang and J. Cui	2024	Activation of the PI3K/AKT signaling pathway by ARNTL2 enhances cellular glycolysis and sensitizes pancreatic adenocarcinoma to erlotinib	Mol Cancer	Duplicate records removed
1365	Ge, W. Y., Y. L. Wang, M. Quan, T. B. Mao, E. Y. Bischof, H. Y. Xu, X. F. Zhang, S. M. Li, M. Yue, J. Y. Ma, H. Y. Yang, L. Wang, Z. Y. Yu, L. W. Wang and J. J. Cui	2024	Activation of the PI3K/AKT signaling pathway by ARNTL2 enhances cellular glycolysis and sensitizes pancreatic adenocarcinoma to erlotinib	Molecular Cancer	Duplicate records removed

1366	Genco, E., F. Modena, L. Sarcina, K. Björkström, C. Brunetti, M. Caironi, M. Caputo, V. M. Demartis, C. Di Franco, G. Frusconi, L. Haeberle, P. Larizza, M. T. Mancini, R. Österbacka, W. Reeves, G. Scamarcio, C. Scandurra, M. Wheeler, E. Cantatore, I. Esposito, E. Macchia, F. Torricelli, F. A. Viola and L. Torsi	2023	A Single-Molecule Bioelectronic Portable Array for Early Diagnosis of Pancreatic Cancer Precursors	Advanced materials (Deerfield Beach, Fla.)	Duplicate records removed
1367	Genco, E., F. Modena, L. Sarcina, K. Björkström, C. Brunetti, M. Caironi, M. Caputo, V. M. Demartis, C. Di Franco, G. Frusconi, L. Haeberle, P. Larizza, M. T. Mancini, R. Österbacka, W. Reeves, G. Scamarcio, C. Scandurra, M. Wheeler, E. Cantatore, I. Esposito, E. Macchia, F. Torricelli, F. A. Viola and L. Torsi	2023	A Single-Molecule Bioelectronic Portable Array for Early Diagnosis of Pancreatic Cancer Precursors	Adv Mater	Duplicate records removed
1368	Goel, S.	2021	PanCan Diagnosed (a miRNA Approach): Using Feature Selection, Ensemble Algorithms, and Interpretability for the Early Diagnosis and Personalized Treatment of Pancreatic Cancer	Pancreas	Duplicate records removed
1369	Gong, J., Q. H. Zhang, Q. M. Peng and D. L. Shi	2024	Identification of Chronic Pancreatitis Associated microRNAs and Genes for the Diagnosis of Pancreatic Cancer	American Surgeon	Duplicate records removed
1370	Gong, Y., L. Song, L. Ou, Y. Y. Lu, X. Huang and Q. Zeng	2023	Diagnostic and Prognostic Performance of MicroRNA-25, Carbohydrate Antigen 19-9, Carcinoembryonic Antigen, and Carbohydrate Antigen 125 in Pancreatic Ductal Adenocarcinoma	Iran J Med Sci	Duplicate records removed
1371	Gong, Y., L. L. Song, L. Ou, Y. Y. Lu, X. Y. Huang and Q. Zeng	2023	Diagnostic and Prognostic Performance of MicroRNA-25, Carbohydrate Antigen 19- 9, Carcinoembryonic Antigen, and Carbohydrate Antigen 125 in Pancreatic Ductal Adenocarcinoma	Iranian Journal of Medical Sciences	Duplicate records removed

1372	González-Moreno, J., Á. Gragera-Martínez, A. Rodríguez, C. Borrachero-Garro, S. García-Garrido, C. Barceló, A. Manovel-Sánchez, M. A. Ribot-Sansó, L. Ibargüen-González, R. Gomila, F. Muñoz-Beamud, I. Losada-López and E. Cisneros-Barroso	2024	Biomarkers of axonal damage to favor early diagnosis in variant transthyretin amyloidosis (A-ATTRv)	Sci Rep	Duplicate records removed
1373	Goonesekere, N. C. W., X. S. Wang, L. Ludwig and C. Guda	2014	A Meta Analysis of Pancreatic Microarray Datasets Yields New Targets as Cancer Genes and Biomarkers	Plos One	Duplicate records removed
1374	Gress, T. M., L. Lausser, L. R. Schirra, L. Ortmüller, R. Diels, B. Kong, C. W. Michalski, T. Hackert, O. Strobel, N. A. Giese, M. Schenk, R. T. Lawlor, A. Scarpa, H. A. Kestler and M. Buchholz	2017	Combined microRNA and mRNA microfluidic TaqMan array cards for the diagnosis of malignancy of multiple types of pancreaticobiliary tumors in fine-needle aspiration material	Oncotarget	Duplicate records removed
1375	Gu, M., J. Sun, S. Zhang, J. Chen, G. Wang, S. Ju and X. Wang	2021	A novel methylation signature predicts inferior outcome of patients with PDAC	Aging (Albany NY)	Duplicate records removed
1376	Gu, M. Q., J. Sun, S. H. Zhang, J. Chen, G. H. Wang, S. Q. Ju and X. D. Wang	2021	A novel methylation signature predicts inferior outcome of patients with PDAC	Aging-Us	Duplicate records removed
1377	Gu, W. and Z. Tong	2020	Clinical Application of Metabolomics in Pancreatic Diseases: A Mini-Review	Lab Med	Duplicate records removed
1378	Gu, Y. L., C. Lan, H. Pei, S. N. Yang, Y. F. Liu and L. L. Xiao	2015	Applicative Value of Serum CA19-9, CEA, CA125 and CA242 in Diagnosis and Prognosis for Patients with Pancreatic Cancer Treated by Concurrent Chemoradiotherapy	Asian Pac J Cancer Prev	Duplicate records removed
1379	Guerrero, P. E., A. Duran, M. R. Ortiz, E. Castro, A. Garcia-Velasco, E. Llop and R. Peracaula	2021	Microfibril associated protein 4 (MFAP4) is a carrier of the tumor associated carbohydrate sialyl-Lewis x (sLe(x)) in pancreatic adenocarcinoma	J Proteomics	Duplicate records removed
1380	Guo, Y. M., J. L. Ren, X. O. Li, X. F. Liu, N. Liu, Y. M. Wang and Z. L. Li	2017	Simultaneous Quantification of Serum Multi-Phospholipids as Potential Biomarkers for Differentiating Different Pathophysiological states of lung, stomach, intestine, and pancreas	Journal of Cancer	Duplicate records removed
1381	Habartová, L., B. Bunganič, M. Tatarkovič, M. Zavoral, J. Vondroušová, K. Syslová and V. Setnička	2018	Chiroptical spectroscopy and metabolomics for blood-based sensing of pancreatic cancer	Chirality	Duplicate records removed

1382	Habartová, L., B. Bunganič, M. Tatarkovič, M. Zavoral, J. Vondroušová, K. Syslová and V. Setnička	2018	Chiroptical spectroscopy and metabolomics for blood-based sensing of pancreatic cancer	Chirality	Duplicate records removed
1383	Han, S. X., X. Zhou, X. Sui, C. C. He, M. J. Cai, J. L. Ma, Y. Y. Zhang, C. Y. Zhou, C. X. Ma, A. Varela-Ramirez and Q. Zhu	2015	Serum dickkopf-1 is a novel serological biomarker for the diagnosis and prognosis of pancreatic cancer	Oncotarget	Duplicate records removed
1384	Han, S. X., X. Zhou, X. Sui, C. C. He, M. J. Cai, J. L. Ma, Y. Y. Zhang, C. Y. Zhou, C. X. Ma, A. Varela-Ramirez and Q. Zhu	2015	Serum dickkopf-1 is a novel serological biomarker for the diagnosis and prognosis of pancreatic cancer	Oncotarget	Duplicate records removed
1385	Hanada, K., A. Shimizu, K. Kurihara, M. Ikeda, T. Yamamoto, Y. Okuda and S. Tazuma	2022	Endoscopic approach in the diagnosis of high-grade pancreatic intraepithelial neoplasia	Dig Endosc	Duplicate records removed
1386	Hata, T., M. Mizuma, T. Kusakabe, H. Amano, T. Furukawa, T. Iwao and M. Unno	2023	Simultaneous and sequential combination of genetic and epigenetic biomarkers for the presence of high-grade dysplasia in patients with pancreatic cyst: Discovery in cyst fluid and test in pancreatic juice	Pancreatology	Duplicate records removed
1387	He, J., J. Long, C. H. Zhai, J. S. Xu, K. W. Bao, W. Q. Su, L. Jiang, G. X. Shen and X. T. Ding	2024	Codetection of Proteins and RNAs on Extracellular Vesicles for Pancreatic Cancer Early Diagnosis	Analytical Chemistry	Duplicate records removed
1388	He, X. Y., J. Zhong, S. W. Wang, Y. F. Zhou, L. Wang, Y. P. Zhang and Y. Z. Yuan	2017	Serum metabolomics differentiating pancreatic cancer from new-onset diabetes	Oncotarget	Duplicate records removed
1389	Henriksen, S. D., P. H. Madsen, A. C. Larsen, M. B. Johansen, A. M. Drewes, I. S. Pedersen, H. Krarup and O. Thorlacius-Ussing	2016	Cell-free DNA promoter hypermethylation in plasma as a diagnostic marker for pancreatic adenocarcinoma	Clinical Epigenetics	Duplicate records removed
1390	Henriksen, S. D., P. H. Madsen, A. C. Larsen, M. B. Johansen, A. M. Drewes, I. S. Pedersen, H. Krarup and O. Thorlacius-Ussing	2016	Cell-free DNA promoter hypermethylation in plasma as a diagnostic marker for pancreatic adenocarcinoma	Clinical epigenetics	Duplicate records removed

1391	Henriksen, S. D., P. H. Madsen, A. C. Larsen, M. B. Johansen, A. M. Drewes, I. S. Pedersen, H. Krarup and O. Thorlacius-Ussing	2016	Cell-free DNA promoter hypermethylation in plasma as a diagnostic marker for pancreatic adenocarcinoma	Clin Epigenetics	Duplicate records removed
1392	Hiramoto, H., T. Muramatsu, D. Ichikawa, K. Tanimoto, S. Yasukawa, E. Otsuji and J. Inazawa	2017	<i>miR</i>-<i>509</i>-<i>5p</i> and <i>miR-1243</i> increase the sensitivity to gemcitabine by inhibiting epithelial-mesenchymal transition in pancreatic cancer	Scientific Reports	Duplicate records removed
1393	Hiramoto, H., T. Muramatsu, D. Ichikawa, K. Tanimoto, S. Yasukawa, E. Otsuji and J. Inazawa	2017	miR-509-5p and miR-1243 increase the sensitivity to gemcitabine by inhibiting epithelial-mesenchymal transition in pancreatic cancer	Sci Rep	Duplicate records removed
1394	Hirata, Y., T. Kobayashi, S. Nishiumi, K. Yamanaka, T. Nakagawa, S. Fujigaki, T. Iemoto, M. Kobayashi, T. Okusaka, S. Nakamori, M. Shimahara, T. Ueno, A. Tsuchida, N. Sata, T. Ioka, Y. Yasunami, T. Kosuge, T. Kaneda, T. Kato, K. Yagihara, S. Fujita, T. Yamada, K. Honda, T. Azuma and M. Yoshida	2017	Identification of highly sensitive biomarkers that can aid the early detection of pancreatic cancer using GC/MS/MS-based targeted metabolomics	Clinica Chimica Acta	Duplicate records removed
1395	Hirata, Y., T. Kobayashi, S. Nishiumi, K. Yamanaka, T. Nakagawa, S. Fujigaki, T. Iemoto, M. Kobayashi, T. Okusaka, S. Nakamori, M. Shimahara, T. Ueno, A. Tsuchida, N. Sata, T. Ioka, Y. Yasunami, T. Kosuge, T. Kaneda, T. Kato, K. Yagihara, S. Fujita, T. Yamada, K. Honda, T. Azuma and M. Yoshida	2017	Identification of highly sensitive biomarkers that can aid the early detection of pancreatic cancer using GC/MS/MS-based targeted metabolomics	Clin Chim Acta	Duplicate records removed
1396	Hogendorf, P., A. Durczynski, A. Skulimowski, A. Kumor, G. Poznanska and J. Strzelczyk	2018	Growth differentiation factor (GDF-15) concentration combined with Ca125 levels in serum is superior to commonly used cancer biomarkers in differentiation of pancreatic mass	Cancer Biomarkers	Duplicate records removed

1397	Hogendorf, P., A. Durczyński, A. Skulimowski, A. Kumor, G. Poznańska and J. Strzelczyk	2018	Growth differentiation factor (GDF-15) concentration combined with Ca125 levels in serum is superior to commonly used cancer biomarkers in differentiation of pancreatic mass	Cancer Biomark	Duplicate records removed
1398	Honda, K.	2022	Risk stratification of pancreatic cancer by a blood test for apolipoprotein A2-isoforms	Cancer Biomarkers	Duplicate records removed
1399	Honda, K.	2022	Risk stratification of pancreatic cancer by a blood test for apolipoprotein A2-isoforms	Cancer Biomark	Duplicate records removed
1400	Honda, K., V. A. Katzke, A. Hüsing, S. Okaya, H. Shoji, K. Onidani, A. Olsen, A. Tjønneland, K. Overvad, E. Weiderpass, P. Vineis, D. Muller, K. Tsilidis, D. Palli, V. Pala, R. Tumino, A. Naccarati, S. Panico, K. Aleksandrova, H. Boeing, H. B. Bueno-de-Mesquita, P. H. Peeters, A. Trichopoulou, P. Lagiou, K. T. Khaw, N. Wareham, R. C. Travis, S. Merino, E. J. Duell, M. Rodríguez-Barranco, M. D. Chirlaque, A. Barricarte, V. Rebours, M. C. Boutron-Ruault, F. Romana Mancini, P. Brennan, G. Scelo, J. Manjer, M. Sund, D. Öhlund, F. Canzian and R. Kaaks	2019	CA19-9 and apolipoprotein-A2 isoforms as detection markers for pancreatic cancer: a prospective evaluation	International Journal of Cancer	Duplicate records removed

1401	Honda, K., V. A. Katzke, A. Hüsing, S. Okaya, H. Shoji, K. Onidani, A. Olsen, A. Tjønneland, K. Overvad, E. Weiderpass, P. Vineis, D. Muller, K. Tsilidis, D. Palli, V. Pala, R. Tumino, A. Naccarati, S. Panico, K. Aleksandrova, H. Boeing, H. B. Bueno-de-Mesquita, P. H. Peeters, A. Trichopoulou, P. Lagiou, K. T. Khaw, N. Wareham, R. C. Travis, S. Merino, E. J. Duell, M. Rodríguez-Barranco, M. D. Chirlaque, A. Barricarte, V. Rebours, M. C. Boutron-Ruault, F. Romana Mancini, P. Brennan, G. Scelo, J. Manjer, M. Sund, D. Öhlund, F. Canzian and R. Kaaks	2019	CA19-9 and apolipoprotein-A2 isoforms as detection markers for pancreatic cancer: a prospective evaluation	Int J Cancer	Duplicate records removed
1402	Honda, K. and S. Srivastava	2016	Potential usefulness of apolipoprotein A2 isoforms for screening and risk stratification of pancreatic cancer	Biomark Med	Duplicate records removed
1403	Huang, J., G. Gao, Y. Ge, J. Liu, H. Cui, R. Zheng, J. Wang, S. Wang, V. L. Go, S. Hu, Y. Liu, M. Yang, Y. Sun, D. Shang, Y. Tian, Z. Zhang, Z. Xiang, H. Wang, J. Guo and G. G. Xiao	2024	Development of a Serum-Based MicroRNA Signature for Early Detection of Pancreatic Cancer: A Multicenter Cohort Study	Dig Dis Sci	Duplicate records removed
1404	Huang, J., G. Gao, Y. Ge, J. Z. Liu, H. T. Cui, R. Zheng, J. L. Wang, S. Wang, V. L. Go, S. Hu, Y. F. Liu, M. W. Yang, Y. W. Sun, D. Shang, Y. T. Tian, Z. G. Zhang, Z. Y. Xiang, H. Y. Wang, J. C. Guo and G. G. Xiao	2024	Development of a Serum-Based MicroRNA Signature for Early Detection of Pancreatic Cancer: A Multicenter Cohort Study	Digestive Diseases and Sciences	Duplicate records removed
1405	Huang, P., W. Gao, C. Fu and R. Tian	2023	Functional and Clinical Proteomic Exploration of Pancreatic Cancer	Mol Cell Proteomics	Duplicate records removed
1406	Huang, W., L. Xue, H. Xu, Z. Kong, J. Xu, H. Zhao and Y. Nie	2021	Diagnostic value of neuronal pentraxin II methylation in patients with pancreatic cancer: Meta-analysis	Int J Clin Pract	Duplicate records removed

1407	Huang, W. Q., L. F. Xue, H. M. Xu, Z. Q. Kong, J. Xu, H. L. Zhao and Y. Q. Nie	2021	Diagnostic value of neuronal pentraxin II methylation in patients with pancreatic cancer: Meta-analysis	International Journal of Clinical Practice	Duplicate records removed
1408	Huang, X. D., F. J. Xiao, Y. T. Guo, Y. Sun, Y. K. Zhang and X. J. Shi	2022	Protein tyrosine phosphatase 1 protects human pancreatic cancer from erastin-induced ferroptosis	Asian journal of surgery	Duplicate records removed
1409	Huang, X. D., F. J. Xiao, Y. T. Guo, Y. Sun, Y. K. Zhang and X. J. Shi	2022	Protein tyrosine phosphatase 1 protects human pancreatic cancer from erastin-induced ferroptosis	Asian J Surg	Duplicate records removed
1410	Huang, Y., F. Chen, L. Zhang, Q. Lv, J. Yan and W. Cui	2021	MALDI-TOF-MS Analysis in the Discovery and Identification of the Serum Peptide Pattern of Pancreatic Ductal Adenocarcinoma	Lab Med	Duplicate records removed
1411	Huang, Y., W. Zhang, Q. Li, Z. Wang and X. Yang	2023	Identification of m6A/m5C/m1A-associated LncRNAs for prognostic assessment and immunotherapy in pancreatic cancer	Sci Rep	Duplicate records removed
1412	Huang, Y. Q., W. Zhang, Q. X. Li, Z. Wang and X. H. Yang	2023	Identification of m6A/m5C/m1A-associated LncRNAs for prognostic assessment and immunotherapy in pancreatic cancer	Scientific Reports	Duplicate records removed
1413	Huang, Z., Z. Jiang, C. Zhao, W. Han, L. Lin, A. Liu, S. Weng and X. Lin	2017	Simple and effective label-free electrochemical immunoassay for carbohydrate antigen 19-9 based on polythionine-Au composites as enhanced sensing signals for detecting different clinical samples	Int J Nanomedicine	Duplicate records removed
1414	Huang, Z. J., Z. Q. Jiang, C. F. Zhao, W. D. Han, L. Q. Lin, A. L. Liu, S. H. Weng and X. H. Lin	2017	Simple and effective label-free electrochemical immunoassay for carbohydrate antigen 19-9 based on polythionine-Au composites as enhanced sensing signals for detecting different clinical samples	International Journal of Nanomedicine	Duplicate records removed
1415	Humeau, M., A. Vignolle-Vidoni, F. Sicard, F. Martins, B. Bournet, L. Buscail, J. Torrisani and P. Cordelier	2015	Salivary microRNA in pancreatic cancer patients	PLoS ONE	Duplicate records removed
1416	Humeau, M., A. Vignolle-Vidoni, F. Sicard, F. Martins, B. Bournet, L. Buscail, J. Torrisani and P. Cordelier	2015	Salivary MicroRNA in Pancreatic Cancer Patients	PLoS One	Duplicate records removed
1417	Husi, H., R. J. E. Skipworth, A. Cronshaw, N. A. Stephens, H. Wackerhage, C. Greig, K. C. H. Fearon and J. A. Ross	2015	Programmed cell death 6 interacting protein (PDCD6IP) and Rabenosyn-5 (ZFYVE20) are potential urinary biomarkers for upper gastrointestinal cancer	Proteomics - Clinical Applications	Duplicate records removed
1418	Hussein, N. A. E. M., Z. A. E. Kholy, M. M. Anwar, M. A. Ahmad and S. M. Ahmad	2017	Plasma miR-22-3p, miR-642b-3p and miR-885-5p as diagnostic biomarkers for pancreatic cancer	Journal of Cancer Research and Clinical Oncology	Duplicate records removed

1419	Huynh, K. Q., A. T. Le, T. T. Phan, T. T. Ho, S. P. Pho, H. T. Nguyen, B. T. Le, T. T. Nguyen and S. T. Nguyen	2023	The Diagnostic Power of Circulating miR-1246 in Screening Cancer: An Updated Meta-analysis	Oxid Med Cell Longev	Duplicate records removed
1420	Ikeura, T., Y. Hori, T. Mitsuyama, H. Miyoshi, M. Shimatani, K. Uchida, M. Takaoka, U. Ota, A. Kamiya, K. Takahashi, M. Ishizuka, M. Kaibori and K. Okazaki	2020	Effectiveness of Photodynamic Screening Using 5-Aminolevulinic Acid for the Diagnosis of Pancreatic Cancer	Anticancer Res	Duplicate records removed
1421	Ishige, F., I. Hoshino, Y. Iwatate, S. Chiba, H. Arimitsu, H. Yanagibashi, H. Nagase and W. Takayama	2020	MIR1246 in body fluids as a biomarker for pancreatic cancer	Scientific reports	Duplicate records removed
1422	Ishige, F., I. Hoshino, Y. Iwatate, S. Chiba, H. Arimitsu, H. Yanagibashi, H. Nagase and W. Takayama	2020	MIR1246 in body fluids as a biomarker for pancreatic cancer	Sci Rep	Duplicate records removed
1423	Iwano, T., K. Yoshimura, G. Watanabe, R. Saito, S. Kiritani, H. Kawaida, T. Moriguchi, T. Murata, K. Ogata, D. Ichikawa, J. Arita, K. Hasegawa and S. Takeda	2021	High-performance collective biomarker from liquid biopsy for diagnosis of pancreatic cancer based on mass spectrometry and machine learning	Journal of Cancer	Duplicate records removed
1424	Jahan, R., K. Ganguly, L. M. Smith, P. Atri, J. Carmicheal, Y. Sheinin, S. Rachagani, G. Natarajan, R. E. Brand, M. A. Macha, P. M. Grandgenett, S. Kaur and S. K. Batra	2019	Trefoil factor(s) and CA19.9: A promising panel for early detection of pancreatic cancer	EBioMedicine	Duplicate records removed
1425	Jahan, R., K. Ganguly, L. M. Smith, P. Atri, J. Carmicheal, Y. Sheinin, S. Rachagani, G. Natarajan, R. E. Brand, M. A. Macha, P. M. Grandgenett, S. Kaur and S. K. Batra	2019	Trefoil factor(s) and CA19.9: A promising panel for early detection of pancreatic cancer	EBioMedicine	Duplicate records removed

1426	Jang, S. I., H. K. Lee, E. J. Chang, S. Kim, S. Y. Kim, I. Y. Hong, J. K. Kim, H. S. Lee, J. Yang, J. H. Cho and D. K. Lee	2023	Improved predictability of pancreatic ductal adenocarcinoma diagnosis using a blood immune cell biomarker panel developed from bulk mRNA sequencing and single-cell RNA-sequencing	Cancer Immunol Immunother	Duplicate records removed
1427	Javed, S., T. A. Qureshi, S. Gaddam, L. X. Wang, L. Azab, A. M. Wachsman, W. S. Chen, V. Asadpour, C. Y. Jeon, B. C. Wu, Y. B. Xie, S. J. Pandol and D. B. Li	2022	Risk prediction of pancreatic cancer using AI analysis of pancreatic subregions in computed tomography images	Frontiers in Oncology	Duplicate records removed
1428	Jelski, W. and B. Mroczko	2019	Biochemical diagnostics of pancreatic cancer - Present and future	Clin Chim Acta	Duplicate records removed
1429	Jia, E., N. Ren, X. Shi, R. Zhang, H. Yu, F. Yu, S. Qin and J. Xue	2022	Extracellular vesicle biomarkers for pancreatic cancer diagnosis: a systematic review and meta-analysis	BMC Cancer	Duplicate records removed
1430	Jiao, B., R. Gulati, H. A. Katki, P. E. Castle and R. Etzioni	2022	A Quantitative Framework to Study Potential Benefits and Harms of Multi-Cancer Early Detection Testing	Cancer Epidemiol Biomarkers Prev	Duplicate records removed
1431	Johansen, J. S., D. Calatayud, V. Albieri, N. A. Schultz, C. Dehlendorff, J. Werner, B. V. Jensen, P. Pfeiffer, S. E. Bojesen, N. Giese, K. R. Nielsen, S. E. Nielsen, M. Yilmaz, N. H. Holländer and K. K. Andersen	2016	The potential diagnostic value of serum microRNA signature in patients with pancreatic cancer	International Journal of Cancer	Duplicate records removed
1432	Johansen, J. S., D. Calatayud, V. Albieri, N. A. Schultz, C. Dehlendorff, J. Werner, B. V. Jensen, P. Pfeiffer, S. E. Bojesen, N. Giese, K. R. Nielsen, S. E. Nielsen, M. Yilmaz, N. H. Holländer and K. K. Andersen	2016	The potential diagnostic value of serum microRNA signature in patients with pancreatic cancer	Int J Cancer	Duplicate records removed
1433	Kamal, M. A., I. Siddiqui, C. Belgiovine, M. Barbagallo, V. Paleari, D. Pistillo, C. Chiabrando, S. Schiarea, B. Bottazzi, R. Leone, R. Avigni, R. Migliore, P. Spaggiari, F. Gavazzi, G. Capretti, F. Marchesi, A. Mantovani, A. Zerbi and P. Allavena	2022	Oncogenic KRAS-Induced Protein Signature in the Tumor Secretome Identifies Laminin-C2 and Pentraxin-3 as Useful Biomarkers for the Early Diagnosis of Pancreatic Cancer	Cancers	Duplicate records removed

1434	Kannan, S., P. Shaik Syed Ali and A. Sheeza	2022	Short report - Lethal and aggressive pancreatic cancer: Molecular pathogenesis, cellular heterogeneity, and biomarkers of pancreatic ductal adenocarcinoma	European Review for Medical and Pharmacological Sciences	Duplicate records removed
1435	Kannan, S., P. Shaik Syed Ali and A. Sheeza	2022	Short report - Lethal and aggressive pancreatic cancer: molecular pathogenesis, cellular heterogeneity, and biomarkers of pancreatic ductal adenocarcinoma	Eur Rev Med Pharmacol Sci	Duplicate records removed
1436	Kartal, E., T. S. B. Schmidt, E. Molina-Montes, S. Rodríguez-Perales, J. Wirbel, O. M. Maistrenko, W. A. Akanni, B. A. Alhamwe, R. J. Alves, A. Carrato, H. P. Erasmus, L. Estudillo, F. Finkelmeier, A. Fullam, A. M. Glazek, P. Gómez-Rubio, R. Hercog, F. Jung, S. Kandels, S. Kersting, M. Langheinrich, M. Márquez, X. Molero, A. Orakov, T. Van Rossum, R. Torres-Ruiz, A. Telzerow, K. Zych, V. Benes, G. Zeller, J. Trebicka, F. X. Real, N. Malats, P. Bork, M. S. Investigators and E. U. S. I. PanGen	2022	A faecal microbiota signature with high specificity for pancreatic cancer	Gut	Duplicate records removed
1437	Kartal, E., T. S. B. Schmidt, E. Molina-Montes, S. Rodríguez-Perales, J. Wirbel, O. M. Maistrenko, W. A. Akanni, B. A. Alhamwe, R. J. Alves, A. Carrato, H. P. Erasmus, L. Estudillo, F. Finkelmeier, A. Fullam, A. M. Glazek, P. Gómez-Rubio, R. Hercog, F. Jung, S. Kandels, S. Kersting, M. Langheinrich, M. Márquez, X. Molero, A. Orakov, T. Van Rossum, R. Torres-Ruiz, A. Telzerow, K. Zych, V. Benes, G. Zeller, J. Trebicka, F. X. Real, N. Malats and P. Bork	2022	A faecal microbiota signature with high specificity for pancreatic cancer	Gut	Duplicate records removed

1438	Kashiro, A., M. Kobayashi, T. Oh, M. Miyamoto, J. Atsumi, K. Nagashima, K. Takeuchi, S. Nara, S. Hijioka, C. Morizane, S. Kikuchi, S. Kato, K. Kato, H. Ochiai, D. Obata, Y. Shizume, H. Konishi, Y. Nomura, K. Matsuyama, C. Xie, C. Wong, Y. Huang, G. Jung, S. Srivastava, H. Kutsumi and K. Honda	2024	Clinical development of a blood biomarker using apolipoprotein-A2 isoforms for early detection of pancreatic cancer	J Gastroenterol	Duplicate records removed
1439	Kashiro, A., M. Kobayashi, T. Oh, M. Miyamoto, J. Atsumi, K. Nagashima, K. Takeuchi, S. Nara, S. Hijioka, C. Morizane, S. Kikuchi, S. Kato, K. Kato, H. Ochiai, D. Obata, Y. Shizume, H. Konishi, Y. Nomura, K. Matsuyama, C. S. Xie, C. S. Wong, Y. Huang, G. M. Jung, S. Srivastava, H. Kutsumi and K. Honda	2024	Clinical development of a blood biomarker using apolipoprotein-A2 isoforms for early detection of pancreatic cancer	Journal of Gastroenterology	Duplicate records removed
1440	Katchman, B. A., R. Barderas, R. Alam, D. Chowell, M. S. Field, L. J. Esserman, G. Wallstrom, J. LaBaer, D. W. Cramer, M. A. Hollingsworth and K. S. Anderson	2016	Proteomic mapping of p53 immunogenicity in pancreatic, ovarian, and breast cancers	Proteomics Clin Appl	Duplicate records removed
1441	Khatri, I. and M. K. Bhasin	2020	A Transcriptomics-Based Meta-Analysis Combined With Machine Learning Identifies a Secretory Biomarker Panel for Diagnosis of Pancreatic Adenocarcinoma	Frontiers in Genetics	Duplicate records removed
1442	Kiio, L. K., J. O. Onyatta, P. M. Ndangili, F. Oloo, C. Santamaria, L. M. Montuenga and D. N. Mbui	2024	Ultrasensitive immunosensor for multiplex detection of cancer biomarkers carcinoembryonic antigen (CEA) and yamaguchi sarcoma viral oncogene homolog 1 (YES1) based on eco-friendly synthesized gold nanoparticles	Talanta	Duplicate records removed
1443	Kiio, L. K., J. O. Onyatta, P. M. Ndangili, F. Oloo, C. Santamaria, L. M. Montuenga and D. N. Mbui	2024	Ultrasensitive immunosensor for multiplex detection of cancer biomarkers carcinoembryonic antigen (CEA) and yamaguchi sarcoma viral oncogene homolog 1 (YES1) based on eco-friendly synthesized gold nanoparticles	Talanta	Duplicate records removed

1444	Kim, A. K., J. P. Hamilton, S. Y. Lin, T. T. Chang, H. W. Hann, C. T. Hu, Y. Lou, Y. J. Lin, T. P. Gade, G. Park, H. Luu, T. J. Lee, J. Wang, D. Chen, M. G. Goggins, S. Jain, W. Song and Y. H. Su	2022	Urine DNA biomarkers for hepatocellular carcinoma screening	Br J Cancer	Duplicate records removed
1445	Kim, H., K. N. Kang, Y. S. Shin, Y. Byun, Y. Han, W. Kwon, C. W. Kim and J. Y. Jang	2020	Biomarker panel for the diagnosis of pancreatic ductal adenocarcinoma	Cancers	Duplicate records removed
1446	Kim, J., W. R. Bamlet, A. L. Oberg, K. G. Chaffee, G. Donahue, X. J. Cao, S. Chari, B. A. Garcia, G. M. Petersen and K. S. Zaret	2017	Detection of early pancreatic ductal adenocarcinoma with thrombospondin-2 and CA19-9 blood markers	Sci Transl Med	Duplicate records removed
1447	Kim, M. W., H. Koh, J. Y. Kim, S. Lee, H. Lee, Y. Kim, H. K. Hwang and S. I. Kim	2021	Tumor-Specific miRNA Signatures in Combination with CA19-9 for Liquid Biopsy-Based Detection of PDAC	Int J Mol Sci	Duplicate records removed
1448	Kim, M. W., H. Koh, J. Y. Kim, S. Lee, H. Lee, Y. Kim, H. K. Hwang and S. I. Kim	2021	Tumor-specific mirna signatures in combination with ca19 9 for liquid biopsy-based detection of pdac	International Journal of Molecular Sciences	Duplicate records removed
1449	Kim, Y. J., H. S. Lee, D. E. Jung, J. M. Kim and S. Y. Song	2017	The DNA aptamer binds stemness-enriched cancer cells in pancreatic cancer	J Mol Recognit	Duplicate records removed
1450	Kobayashi, M., A. Fujita, T. Ogawa, Y. Tanisaka, M. Mizuide, N. Kondo, Y. Imaizumi, T. Hirotsu and S. Ryozawa	2021	Caenorhabditis elegans as a Diagnostic Aid for Pancreatic Cancer	Pancreas	Duplicate records removed
1451	Konno, N., R. Suzuki, T. Takagi, M. Sugimoto, H. Asama, Y. Sato, H. Irie, T. Hikichi and H. Ohira	2021	Clinical utility of a newly developed microfluidic device for detecting circulating tumor cells in the blood of patients with pancreatico-biliary malignancies	J Hepatobiliary Pancreat Sci	Duplicate records removed
1452	Konno, N., R. Suzuki, T. Takagi, M. Sugimoto, Y. Sato, H. Irie, T. Hikichi, K. Watanabe, J. Nakamura, M. Takasumi, M. Hashimoto and H. Ohira	2018	Clinical utility of newly developed microfluidic device for detecting circulating tumor cells in the blood of panceatico-biliary malignancies	United European Gastroenterology Journal	Duplicate records removed

1453	Korfiatis, P., G. Suman, N. G. Patnam, K. H. Trivedi, A. Karbhari, S. Mukherjee, C. Cook, J. R. Klug, A. Patra, H. Khasawneh, N. Rajamohan, J. G. Fletcher, M. J. Truty, S. Majumder, C. W. Bolan, K. Sandrasegaran, S. T. Chari and A. H. Goenka	2023	Automated Artificial Intelligence Model Trained on a Large Data Set Can Detect Pancreas Cancer on Diagnostic Computed Tomography Scans As Well As Visually Occult Preinvasive Cancer on Prediagnostic Computed Tomography Scans	Gastroenterology	Duplicate records removed
1454	Korfiatis, P., G. Suman, N. G. Patnam, K. H. Trivedi, A. Karbhari, S. Mukherjee, C. Cook, J. R. Klug, A. Patra, H. Khasawneh, N. Rajamohan, J. G. Fletcher, M. J. Truty, S. Majumder, C. W. Bolan, K. Sandrasegaran, S. T. Chari and A. H. Goenka	2023	Automated Artificial Intelligence Model Trained on a Large Data Set Can Detect Pancreas Cancer on Diagnostic Computed Tomography Scans As Well As Visually Occult Preinvasive Cancer on Prediagnostic Computed Tomography Scans	Gastroenterology	Duplicate records removed
1455	Kowada, A.	2022	Cost-effectiveness of MicroRNA for Pancreatic Cancer Screening in Patients With Diabetes	Pancreas	Duplicate records removed
1456	Kowada, A.	2022	Cost-effectiveness of MicroRNA for Pancreatic Cancer Screening in Patients with Diabetes	Pancreas	Duplicate records removed
1457	Kowada, A.	2022	Cost-effectiveness of MicroRNA for Pancreatic Cancer Screening in Patients With Diabetes	Pancreas	Duplicate records removed
1458	Krasnoslobodtsev, A. V., M. P. Torres, S. Kaur, I. V. Vlassiouk, R. J. Lipert, M. Jain, S. K. Batra and Y. L. Lyubchenko	2015	Nano-immunoassay with improved performance for detection of cancer biomarkers	Nanomedicine: Nanotechnology, Biology, and Medicine	Duplicate records removed
1459	Krasnoslobodtsev, A. V., M. P. Torres, S. Kaur, I. V. Vlassiouk, R. J. Lipert, M. Jain, S. K. Batra and Y. L. Lyubchenko	2015	Nano-immunoassay with improved performance for detection of cancer biomarkers	Nanomedicine	Duplicate records removed
1460	Kriz, D., D. Ansari and R. Andersson	2020	Potential biomarkers for early detection of pancreatic ductal adenocarcinoma	Clin Transl Oncol	Duplicate records removed
1461	Kruger, D., Y. Y. Yako, J. Devar, N. Lahoud and M. Smith	2019	Inflammatory cytokines and combined biomarker panels in pancreatic ductal adenocarcinoma: Enhancing diagnostic accuracy	PLoS One	Duplicate records removed
1462	Kumar, V., A. K. Chaudhary, Y. Dong, H. A. Zhong, G. Mondal, F. Lin, V. Kumar and R. I. Mahato	2017	Design, Synthesis and Biological Evaluation of novel Hedgehog Inhibitors for treating Pancreatic Cancer	Sci Rep	Duplicate records removed

1463	Kunovsky, L., P. Tesarikova, Z. Kala, R. Kroupa, P. Kysela, J. Dolina and J. Trna	2018	The Use of Biomarkers in Early Diagnostics of Pancreatic Cancer	Can J Gastroenterol Hepatol	Duplicate records removed
1464	Laeseke, P. F., R. Chen, R. B. Jeffrey, T. A. Brentnall and J. K. Willmann	2015	Combining in Vitro Diagnostics with in Vivo Imaging for Earlier Detection of Pancreatic Ductal Adenocarcinoma: Challenges and Solutions	Radiology	Duplicate records removed
1465	Lane, J. S., D. Von Hoff, D. Cridebring and A. Goel	2020	Extracellular vesicles in diagnosis and treatment of pancreatic cancer: Current state and future perspectives	Cancers	Duplicate records removed
1466	Lee, D. H., W. Yoon, A. Lee, Y. Han, Y. Byun, J. S. Kang, H. Kim, W. Kwon, Y. A. Suh, Y. Choi, J. Namkung, S. Han, S. G. Yi, J. S. Heo, I. W. Han, J. O. Park, J. K. Park, S. C. Kim, E. Jun, C. M. Kang, W. J. Lee, H. K. Lee, H. Lee, S. Lee, S. Y. Jeong, K. E. Lee, W. Han, T. Park and J. Y. Jang	2023	Multi-biomarker panel prediction model for diagnosis of pancreatic cancer	Journal of Hepato-Biliary-Pancreatic Sciences	Duplicate records removed
1467	Lee, D. H., W. Yoon, A. Lee, Y. Han, Y. Byun, J. S. Kang, H. Kim, W. Kwon, Y. A. Suh, Y. Choi, J. Namkung, S. Han, S. G. Yi, J. S. Heo, I. W. Han, J. O. Park, J. K. Park, S. C. Kim, E. Jun, C. M. Kang, W. J. Lee, H. K. Lee, H. Lee, S. Lee, S. Y. Jeong, K. E. Lee, W. Han, T. Park and J. Y. Jang	2023	Multi-biomarker panel prediction model for diagnosis of pancreatic cancer	J Hepatobiliary Pancreat Sci	Duplicate records removed
1468	Lee, J. H., L. S. Cassani, P. Bhosale and W. A. Ross	2016	The endoscopist's role in the diagnosis and management of pancreatic cancer	Expert Rev Gastroenterol Hepatol	Duplicate records removed
1469	Lee, J. H., Y. H. Kim, K. H. Kim, J. Y. Cho, S. M. Woo, B. C. Yoo and S. C. Kim	2018	Profiling of Serum Metabolites Using MALDI-TOF and Triple-TOF Mass Spectrometry to Develop a Screen for Ovarian Cancer	Cancer Res Treat	Duplicate records removed
1470	Lee, J. H., Y. H. Kim, K. H. Kim, J. Y. Cho, S. M. Woo, B. Y. Yoo and S. C. Kim	2018	Profiling of serum metabolites using maldi-tof and triple-tof mass spectrometry to develop a screen for ovarian cancer	Cancer Research and Treatment	Duplicate records removed
1471	Lee, M. J., K. Na, S. K. Jeong, J. S. Lim, S. A. Kim, M. J. Lee, S. Y. Song, H. Kim, W. S. Hancock and Y. K. Paik	2014	Identification of human complement factor B as a novel biomarker candidate for pancreatic ductal adenocarcinoma	Journal of Proteome Research	Duplicate records removed

1472	Lee, M. J., K. Na, S. K. Jeong, J. S. Lim, S. A. Kim, M. J. Lee, S. Y. Song, H. Kim, W. S. Hancock and Y. K. Paik	2014	Identification of human complement factor B as a novel biomarker candidate for pancreatic ductal adenocarcinoma	J Proteome Res	Duplicate records removed
1473	Lei, X. F., S. Z. Jia, J. Ye, Y. L. Qiao, G. M. Zhao, X. H. Li and H. Chang	2017	Application values of detection of serum CA199, CA242 and CA50 in the diagnosis of pancreatic cancer	J Biol Regul Homeost Agents	Duplicate records removed
1474	Lenggenhager, D., S. Bengs, R. Fritsch, S. Hussung, P. Busenhart, K. Endhardt, A. Töpfer, F. O. The, S. Bütikofer, C. Gubler, M. Scharl and B. Morell	2021	β6-Integrin Serves as a Potential Serum Marker for Diagnosis and Prognosis of Pancreatic Adenocarcinoma	Clin Transl Gastroenterol	Duplicate records removed
1475	Levink, I. J. M., D. C. F. Klatte, R. G. Hanna-Sawires, G. C. M. Vreeker, I. S. Ibrahim, Y. E. M. van der Burgt, K. A. Overbeek, B. D. M. Koopmann, D. L. Cahen, G. M. Fuhler, M. Wuhrer, B. A. Bonsing, R. Tollenaar, F. P. Vleggaar, H. F. A. Vasen, M. E. van Leerdam, M. J. Bruno and W. E. Mesker	2022	Longitudinal changes of serum protein N-Glycan levels for earlier detection of pancreatic cancer in high-risk individuals	Pancreatology	Duplicate records removed
1476	Levink, I. J. M., D. C. F. Klatte, R. G. Hanna-Sawires, G. C. M. Vreeker, I. S. Ibrahim, Y. E. M. van der Burgt, K. A. Overbeek, B. D. M. Koopmann, D. L. Cahen, G. M. Fuhler, M. Wuhrer, B. A. Bonsing, R. A. E. M. Tollenaar, F. P. Vleggaar, H. F. A. Vasen, M. E. van Leerdam, M. J. Bruno and W. E. Mesker	2022	Longitudinal changes of serum protein N-Glycan levels for earlier detection of pancreatic cancer in high-risk individuals	Pancreatology	Duplicate records removed
1477	Lewis, A. R., J. W. Valle and M. G. McNamara	2016	Pancreatic cancer: Are "liquid biopsies" ready for prime-time?	World J Gastroenterol	Duplicate records removed
1478	Lewis, J. M., A. D. Vyas, Y. Qiu, K. S. Messer, R. White and M. J. Heller	2018	Integrated Analysis of Exosomal Protein Biomarkers on Alternating Current Electrokinetic Chips Enables Rapid Detection of Pancreatic Cancer in Patient Blood	ACS Nano	Duplicate records removed
1479	Lewis, J. M., A. D. Vyas, Y. Q. Qiu, K. S. Messer, R. White and M. J. Heller	2018	Integrated Analysis of Exosomal Protein Biomarkers on Alternating Current Electrokinetic Chips Enables Rapid Detection of Pancreatic Cancer in Patient Blood	Acs Nano	Duplicate records removed

1480	Li, H., A. R. Warden, W. Su, J. He, X. Zhi, K. Wang, L. Zhu, G. Shen and X. Ding	2021	Highly sensitive and portable mRNA detection platform for early cancer detection	J Nanobiotechnology	Duplicate records removed
1481	Li, J., Y. R. Li, S. Chen, W. L. Duan, X. Kong, Y. S. Wang, L. Q. Zhou, P. L. Li, C. P. Zhang, L. T. Du and C. X. Wang	2022	Highly Sensitive Exosome Detection for Early Diagnosis of Pancreatic Cancer Using Immunoassay Based on Hierarchical Surface-Enhanced Raman Scattering Substrate	Small Methods	Duplicate records removed
1482	Li, L., M. Liu, J. B. Lin, X. B. Hong, W. X. Chen, H. Guo, L. Y. Xu, Y. W. Xu, E. M. Li and Y. H. Peng	2017	Diagnostic Value of Autoantibodies against Ezrin in Esophageal Squamous Cell Carcinoma	Dis Markers	Duplicate records removed
1483	Li, L. X., B. Zhang, R. Z. Gong and P. Severino	2020	Insights into the role of tumor abnormal protein in early diagnosis of cancer: A prospective cohort study	Medicine (United States)	Duplicate records removed
1484	Li, Q., S. H. Maier, P. Li, J. Peterhansl, C. Belka, J. Mayerle and U. M. Mahajan	2020	Aptamers: a novel targeted theranostic platform for pancreatic ductal adenocarcinoma	Radiat Oncol	Duplicate records removed
1485	Li, T. H., N. Gan, D. Z. Wu, H. J. Jin, Y. T. Cao and Q. L. Jiang	2014	An Ultrasensitive Simultaneous Multianalyte Immunoassay Based on Arsenic and Mercury Ions Labeled SiO₂@Au Nanoparticle Probes	Chinese Journal of Analytical Chemistry	Duplicate records removed
1486	Li, W. Y., M. Gonzalez-Gonzalez, L. Sanz-Criado, N. Garcia-Carbonero, A. Celdran, P. Villarejo-Campos, P. Minguez, R. Pazo-Cid, C. Garcia-Jimenez, A. Orta-Ruiz, J. Garcia-Foncillas and J. Martinez-Useros	2022	A Novel PiRNA Enhances CA19-9 Sensitivity for Pancreatic Cancer Identification by Liquid Biopsy	Journal of Clinical Medicine	Duplicate records removed
1487	Li, X., X. Guo, H. Li, H. Lin and Y. Sun	2014	Serum carbohydrate antigen 242 expression exerts crucial function in the diagnosis of pancreatic cancer	Tumour Biol	Duplicate records removed
1488	Li, Y., K. Unger, C. Hinzman, M. Jayatilake, A. Iliuk, S. Bansal, M. Girgis, P. Banerjee, J. B. Tyburski, T. Bauer and A. K. Cheema	2021	Extracellular vesicle based multi-omics prediction model for the early detection of pancreatic cancer	Cancer Research	Duplicate records removed
1489	Li, Y., K. Unger, C. Hinzman, M. Jayatilake, A. Iliuk, S. Bansal, M. Girgis, P. Banerjee, J. B. Tyburski, T. Bauer and et al.	2021	Extracellular vesicle based multi-omics prediction model for the early detection of pancreatic cancer	Cancer research	Duplicate records removed
1490	Li, Y., L. Wu, W. Tao, D. Wu, F. Ma and N. Li	2020	Expression Atlas of FGF and FGFR Genes in Pancancer Uncovered Predictive Biomarkers for Clinical Trials of Selective FGFR Inhibitors	Biomed Res Int	Duplicate records removed

1491	Liang, B., L. S. Zhong, Q. He, S. C. Wang, Z. C. Pan, T. J. Wang and Y. J. Zhao	2015	Serum dickkopf-1 as a biomarker in screening gastrointestinal cancers: a systematic review and meta-analysis	Oncotargets and Therapy	Duplicate records removed
1492	Lin, M., M. Alnaggar, S. Liang, J. Chen, K. Xu, S. Dong, D. Du and L. Niu	2018	Circulating Tumor DNA as a Sensitive Marker in Patients Undergoing Irreversible Electroporation for Pancreatic Cancer	Cell Physiol Biochem	Duplicate records removed
1493	Lin, M., M. Alnaggar, S. Z. Liang, J. B. Chen, K. C. Xu, S. H. Dong, D. M. Du and L. Z. Niu	2018	Circulating Tumor DNA as a Sensitive Marker in Patients Undergoing Irreversible Electroporation for Pancreatic Cancer	Cellular Physiology and Biochemistry	Duplicate records removed
1494	Lin, M. S., W. C. Chen, J. X. Huang, H. J. Gao and H. H. Sheng	2014	Aberrant expression of microRNAs in serum may identify individuals with pancreatic cancer	International Journal of Clinical and Experimental Medicine	Duplicate records removed
1495	Linh, V. T. N., M. Y. Lee, J. Mun, Y. Kim, H. Kim, I. W. Han, S. G. Park, S. Choi, D. H. Kim, J. Rho and H. S. Jung	2023	3D plasmonic coral nanoarchitecture paper for label-free human urine sensing and deep learning-assisted cancer screening	Biosens Bioelectron	Duplicate records removed
1496	Linh, V. T. N., J. Mun, Y. Kim, I. W. Han, H. Kim, S. G. Park, S. Choi, D. H. Kim, J. Rho and H. S. Jung	2023	3D plasmonic coral nanoarchitecture paper for label-free human urine sensing and deep learning-assisted cancer screening	Biosensors & Bioelectronics	Duplicate records removed
1497	Liu, J. X., A. Li, L. Y. Zhou, X. F. Liu, Z. H. Wei, X. Z. Wang and H. Q. Ying	2018	Significance of combined preoperative serum Alb and dNLR for diagnosis of pancreatic cancer	Future Oncol	Duplicate records removed
1498	Liu, W., W. Liu, K. Lin, Y. Liu, H. Hu and L. Yang	2022	A clinical study of serum human epididymis protein 4 (HE4) in the diagnosis of pancreatic cancer	Indian J Cancer	Duplicate records removed
1499	Liu, W. Q., W. L. Liu, K. Lin, Y. H. Liu, H. Hu and L. N. Yang	2022	A clinical study of serum human epididymis protein 4 (HE4) in the diagnosis of pancreatic cancer	Indian Journal of Cancer	Duplicate records removed
1500	Liu, X., W. Zheng, W. Wang, H. Shen, L. Liu, W. Lou, X. Wang and P. Yang	2017	A new panel of pancreatic cancer biomarkers discovered using a mass spectrometry-based pipeline	Br J Cancer	Duplicate records removed
1501	Liu, X. D., H. Wu, Y. Li, X. Liu, Z. Zhang, L. Yu, Z. Qin, Z. Su, R. Liu, Q. He, M. Dai and Z. Liang	2019	Early detection of pancreatic ductal adenocarcinoma using methylation signatures in circulating tumour DNA	Annals of Oncology	Duplicate records removed
1502	Llop, E., E. G. P, A. Duran, S. Barrabés, A. Massaguer, M. José Ferri, M. Albiol-Quer, R. de Llorens and R. Peracaula	2018	Glycoprotein biomarkers for the detection of pancreatic ductal adenocarcinoma	World J Gastroenterol	Duplicate records removed
1503	Long, N. P., S. J. Yoon, N. H. Anh, T. D. Nghi, D. K. Lim, Y. J. Hong, S. S. Hong and S. W. Kwon	2018	A systematic review on metabolomics-based diagnostic biomarker discovery and validation in pancreatic cancer	Metabolomics	Duplicate records removed

1504	Loosen, S. H., U. P. Neumann, C. Trautwein, C. Roderburg and T. Luedde	2017	Current and future biomarkers for pancreatic adenocarcinoma	Tumour Biol	Duplicate records removed
1505	Lozanovski, V. J., P. Houben, U. Hinz, T. Hackert, I. Herr and P. Schemmer	2014	Pilot study evaluating broccoli sprouts in advanced pancreatic cancer (POUDER trial) - study protocol for a randomized controlled trial	Trials	Duplicate records removed
1506	Luo, G., M. Guo, K. Jin, Z. Liu, C. Liu, H. Cheng, Y. Lu, J. Long, L. Liu, J. Xu, Q. Ni and X. Yu	2016	Optimize CA19-9 in detecting pancreatic cancer by Lewis and Secretor genotyping	Pancreatology	Duplicate records removed
1507	Luo, G., K. Jin, S. Deng, H. Cheng, Z. Fan, Y. Gong, Y. Qian, Q. Huang, Q. Ni, C. Liu and X. Yu	2021	Roles of CA19-9 in pancreatic cancer: Biomarker, predictor and promoter	Biochim Biophys Acta Rev Cancer	Duplicate records removed
1508	Luo, G. P., M. Guo, K. Z. Jin, Z. L. Liu, C. Liu, H. Cheng, Y. Lu, J. Long, L. Liu, J. Xu, Q. X. Ni and X. J. Yu	2016	Optimize CA19-9 in detecting pancreatic cancer by Lewis and Secretor genotyping	Pancreatology	Duplicate records removed
1509	Luo, X., J. Liu, H. Wang and H. Lu	2020	Metabolomics identified new biomarkers for the precise diagnosis of pancreatic cancer and associated tissue metastasis	Pharmacol Res	Duplicate records removed
1510	Luo, X. L., J. J. Liu, H. Z. Wang and H. T. Lu	2020	Metabolomics identified new biomarkers for the precise diagnosis of pancreatic cancer and associated tissue metastasis	Pharmacological Research	Duplicate records removed
1511	Madhavan, B., S. Yue, U. Galli, S. Rana, W. Gross, M. Müller, N. A. Giese, H. Kalthoff, T. Becker, M. W. Bü chler and M. Zöller	2015	Combined evaluation of a panel of protein and miRNA serum-exosome biomarkers for pancreatic cancer diagnosis increases sensitivity and specificity	Int J Cancer	Duplicate records removed
1512	Madhavan, B., S. J. Yue, U. Galli, S. Rana, W. Gross, M. Müller, N. A. Giese, H. Kalthoff, T. Becker, M. W. Büchler and M. Zö ller	2015	Combined evaluation of a panel of protein and miRNA serum-exosome biomarkers for pancreatic cancer diagnosis increases sensitivity and specificity	International Journal of Cancer	Duplicate records removed
1513	Majumder, S., S. T. Chari and D. A. Ahlquist	2015	Molecular detection of pancreatic neoplasia: Current status and future promise	World Journal of Gastroenterology	Duplicate records removed
1514	Majumder, S., S. T. Chari and D. A. Ahlquist	2015	Molecular detection of pancreatic neoplasia: Current status and future promise	World J Gastroenterol	Duplicate records removed
1515	Makler, A. and W. Asghar	2023	Exosomal miRNA Biomarker Panel for Pancreatic Ductal Adenocarcinoma Detection in Patient Plasma: A Pilot Study	Int J Mol Sci	Duplicate records removed
1516	Malhotra, A., B. Rachet, A. Bonaventure, S. P. Pereira and L. M. Woods	2021	Can we screen for pancreatic cancer? Identifying a sub-population of patients at high risk of subsequent diagnosis using machine learning techniques applied to primary care data	PLoS ONE	Duplicate records removed

1517	Malhotra, A., B. Rachet, A. Bonaventure, S. P. Pereira and L. M. Woods	2021	Can we screen for pancreatic cancer? Identifying a sub-population of patients at high risk of subsequent diagnosis using machine learning techniques applied to primary care data	PLoS One	Duplicate records removed
1518	Malhotra, P., I. Casari and M. Falasca	2023	Can the molecules carried by extracellular vesicles help to diagnose pancreatic cancer early?	Biochimica et Biophysica Acta - General Subjects	Duplicate records removed
1519	Malhotra, P., I. Casari and M. Falasca	2023	Can the molecules carried by extracellular vesicles help to diagnose pancreatic cancer early?	Biochim Biophys Acta Gen Subj	Duplicate records removed
1520	Manchado, E., S. Weissmueller, J. P. t. Morris, C. C. Chen, R. Wullenkord, A. Lujambio, E. de Stanchina, J. T. Poirier, J. F. Gainor, R. B. Corcoran, J. A. Engelman, C. M. Rudin, N. Rosen and S. W. Lowe	2016	A combinatorial strategy for treating KRAS-mutant lung cancer	Nature	Duplicate records removed
1521	Marengo, E. and E. Robotti	2014	Biomarkers for pancreatic cancer: Recent achievements in proteomics and genomics through classical and multivariate statistical methods	World Journal of Gastroenterology	Duplicate records removed
1522	Marengo, E. and E. Robotti	2014	Biomarkers for pancreatic cancer: recent achievements in proteomics and genomics through classical and multivariate statistical methods	World J Gastroenterol	Duplicate records removed
1523	Marin, A. M., M. Batista, A. L. Korte de Azevedo, T. H. Bombardelli Gomig, R. Soares Caldeira Brant, R. Chammas, M. Uno, D. Dias Araú jo, D. L. Zanette and M. Nóbrega Aoki	2023	Screening of Exosome-Derived Proteins and Their Potential as Biomarkers in Diagnostic and Prognostic for Pancreatic Cancer	International Journal of Molecular Sciences	Duplicate records removed
1524	Marin, A. M., M. Batista, A. L. Korte de Azevedo, T. H. Bombardelli Gomig, R. Soares Caldeira Brant, R. Chammas, M. Uno, D. Dias Araú jo, D. L. Zanette and M. Nóbrega Aoki	2023	Screening of Exosome-Derived Proteins and Their Potential as Biomarkers in Diagnostic and Prognostic for Pancreatic Cancer	Int J Mol Sci	Duplicate records removed
1525	Masterson, A. N., N. N. Chowdhury, Y. Fang, M. T. Yip-Schneider, S. Hati, P. Gupta, S. Cao, H. Wu, C. M. Schmidt, M. L. Fishel and R. Sardar	2023	Amplification-Free, High-Throughput Nanoplasmonic Quantification of Circulating MicroRNAs in Unprocessed Plasma Microsamples for Earlier Pancreatic Cancer Detection	ACS Sens	Duplicate records removed

1526	Masterson, A. N., N. N. Chowdhury, Y. Fang, M. T. Yip-Schneider, S. Hati, P. Gupta, S. Cao, H. B. Wu, C. M. Schmidt, M. L. Fishel and R. Sardar	2023	Amplification-Free, High-Throughput Nanoplasmonic Quantification of Circulating MicroRNAs in Unprocessed Plasma Microsamples for Earlier Pancreatic Cancer Detection	Acs Sensors	Duplicate records removed
1527	Mateo, L., M. Duran-Frigola, A. Gris-Oliver, M. Palafox, M. Scaltriti, P. Razavi, S. Chandarlapaty, J. Arribas, M. Bellet, V. Serra and P. Aloy	2020	Personalized cancer therapy prioritization based on driver alteration co-occurrence patterns	Genome Medicine	Duplicate records removed
1528	Matsunaga, T., T. Ohtsuka, K. Asano, H. Kimura, K. Ohuchida, H. Kitada, N. Ideno, Y. Mori, S. Tokunaga, Y. Oda, S. Guha, M. Raimondo, M. Nakamura and M. Tanaka	2017	S100P in Duodenal Fluid Is a Useful Diagnostic Marker for Pancreatic Ductal Adenocarcinoma	Pancreas	Duplicate records removed
1529	Mazer, B. L., J. W. Lee, N. J. Roberts, L. C. Chu, A. M. Lennon, A. P. Klein, J. R. Eshleman, E. K. Fishman, M. I. Canto, M. G. Goggins and R. H. Hruban	2023	Screening for pancreatic cancer has the potential to save lives, but is it practical?	Expert Rev Gastroenterol Hepatol	Duplicate records removed
1530	Mehta, K. Y., H. J. Wu, S. S. Menon, Y. Fallah, X. G. Zhong, N. Rizk, K. Unger, M. Mapstone, M. S. Fiandaca, H. J. Federoff and A. K. Cheema	2017	Metabolomic biomarkers of pancreatic cancer - a meta-analysis study	Oncotarget	Duplicate records removed
1531	Meidhof, S., S. Brabletz, W. Lehmann, B. T. Preca, K. Mock, M. Ruh, J. Schüler, M. Berthold, A. Weber, U. Burk, M. Lübbert, M. Puhr, Z. Culig, U. Wellner, T. Keck, P. Bronsert, S. Küsters, U. T. Hopt, M. P. Stemmler and T. Brabletz	2015	ZEB1-associated drug resistance in cancer cells is reversed by the class I HDAC inhibitor mocetinostat	EMBO Molecular Medicine	Duplicate records removed

1532	Meidhof, S., S. Brabletz, W. Lehmann, B. T. Preca, K. Mock, M. Ruh, J. Schüler, M. Berthold, A. Weber, U. Burk, M. Lübbert, M. Puhr, Z. Culig, U. Wellner, T. Keck, P. Bronsert, S. Küsters, U. T. Hopt, M. P. Stemmler and T. Brabletz	2015	ZEB1-associated drug resistance in cancer cells is reversed by the class I HDAC inhibitor mocetinostat	EMBO Mol Med	Duplicate records removed
1533	Melson, J., Y. Li, E. Cassinotti, A. Melnikov, L. Boni, J. Ai, M. Greenspan, S. Mobarhan, V. Levenson and Y. Deng	2014	Commonality and differences of methylation signatures in the plasma of patients with pancreatic cancer and colorectal cancer	Int J Cancer	Duplicate records removed
1534	Melson, J., Y. Li, E. Cassinotti, A. Melnikov, L. Boni, J. M. Ai, M. Greenspan, S. Mobarhan, V. Levenson and Y. P. Deng	2014	Commonality and differences of methylation signatures in the plasma of patients with pancreatic cancer and colorectal cancer	International Journal of Cancer	Duplicate records removed
1535	Metzenmacher, M., R. Váraljai, B. Hegedüs, I. Cima, J. Forster, A. Schramm, B. Scheffler, P. A. Horn, C. A. Klein, T. Szarvas, H. Reis, N. Bielefeld, A. Roesch, C. Aigner, V. Kunzmann, M. Wiesweg, J. T. Siveke, M. Schuler and S. S. Lueong	2020	Plasma next generation sequencing and droplet digital-qpcr-based quantification of circulating cell-free rna for noninvasive early detection of cancer	Cancers	Duplicate records removed
1536	Michael Traeger, M., J. Rehkaemper, H. Ullerich, K. Steinestel, E. Wardelmann, N. Senninger and S. Abdallah Dhayat	2018	The ambiguous role of microRNA205 and its clinical potential in pancreatic ductal adenocarcinoma	J Cancer Res Clin Oncol	Duplicate records removed
1537	Michálková, L., Š. Horník, J. Sýkora, L. Habartová and V. Setnička	2018	Diagnosis of pancreatic cancer via(1)H NMR metabolomics of human plasma	Analyst	Duplicate records removed
1538	Middleton, G., D. H. Palmer, W. Greenhalf, P. Ghaneh, R. Jackson, T. Cox, A. Evans, V. E. Shaw, J. Wadsley, J. W. Valle and et al.	2017	Vandetanib plus gemcitabine versus placebo plus gemcitabine in locally advanced or metastatic pancreatic carcinoma (ViP): a prospective, randomised, double-blind, multicentre phase 2 trial	The lancet. Oncology	Duplicate records removed

1539	Mitachi, K., K. Ariake, H. Shima, S. Sato, T. Miura, S. Maeda, M. Ishida, M. Mizuma, H. Ohtsuka, T. Kamei, K. Igarashi and M. Unno	2021	Novel candidate factors predicting the effect of S-1 adjuvant chemotherapy of pancreatic cancer	Sci Rep	Duplicate records removed
1540	Modlin, I. M., M. Kidd, K. Oberg, M. Falconi, P. L. Filosso, A. Frilling, A. Malczewska, R. Salem, C. Toumpanakis, F. M. Laskaratos, S. Partelli, M. Roffinella, C. von Arx, B. K. Kudla, L. Bodei, I. A. Drozdov and A. Kitz	2021	Early Identification of Residual Disease After Neuroendocrine Tumor Resection Using a Liquid Biopsy Multigenomic mRNA Signature (NETest)	Annals of Surgical Oncology	Duplicate records removed
1541	Modlin, I. M., M. Kidd, K. Oberg, M. Falconi, P. L. Filosso, A. Frilling, A. Malczewska, R. Salem, C. Toumpanakis, F. M. Laskaratos, S. Partelli, M. Roffinella, C. von Arx, B. K. Kudla, L. Bodei, I. A. Drozdov and A. Kitz	2021	Early Identification of Residual Disease After Neuroendocrine Tumor Resection Using a Liquid Biopsy Multigenomic mRNA Signature (NETest)	Ann Surg Oncol	Duplicate records removed
1542	Mohamed, A. A., H. Soliman, M. Ismail, D. Ziada, T. M. Farid, A. M. Aref, M. E. Al Daly and Z. Y. Abd Elmageed	2015	Evaluation of circulating ADH and MIC-1 as diagnostic markers in Egyptian patients with pancreatic cancer	Pancreatology	Duplicate records removed
1543	Mosier, B. R. and L. E. Bantis	2021	Estimation and construction of confidence intervals for biomarker cutoff-points under the shortest Euclidean distance from the ROC surface to the perfection corner	Stat Med	Duplicate records removed
1544	Moutinho-Ribeiro, P., I. A. Batista, S. T. Quintas, B. Adem, M. Silva, R. Morais, A. Peixoto, R. Coelho, P. Costa-Moreira, R. Medas, S. Lopes, F. Vilas-Boas, M. Baptista, D. Dias-Silva, A. L. Esteves, F. Martins, J. Lopes, H. Barroca, F. Carneiro, G. MacEdo and S. A. Melo	2022	Exosomal glypican-1 is elevated in pancreatic cancer precursors and can signal genetic predisposition in the absence of endoscopic ultrasound abnormalities	World Journal of Gastroenterology	Duplicate records removed

1545	Moutinho-Ribeiro, P., I. A. Batista, S. T. Quintas, B. Adem, M. Silva, R. Morais, A. Peixoto, R. Coelho, P. Costa-Moreira, R. Medas, S. Lopes, F. Vilas-Boas, M. Baptista, D. Dias-Silva, A. L. Esteves, F. Martins, J. Lopes, H. Barroca, F. Carneiro, G. Macedo and S. A. Melo	2022	Exosomal glypican-1 is elevated in pancreatic cancer precursors and can signal genetic predisposition in the absence of endoscopic ultrasound abnormalities	World J Gastroenterol	Duplicate records removed
1546	Nakamura, K., Z. Zhu, S. Roy, E. Jun, H. Han, R. M. Munoz, S. Nishiwada, G. Sharma, D. Cridebring, F. Zenhausern, S. Kim, D. J. Roe, S. Darabi, I. W. Han, D. B. Evans, S. Yamada, M. J. Demeure, C. Becerra, S. A. Celinski, E. Borazanci, S. Tsai, Y. Kodera, J. O. Park, J. S. Bolton, X. Wang, S. C. Kim, D. Von Hoff and A. Goel	2022	An Exosome-based Transcriptomic Signature for Noninvasive, Early Detection of Patients With Pancreatic Ductal Adenocarcinoma: A Multicenter Cohort Study	Gastroenterology	Duplicate records removed
1547	Nakamura, K., Z. X. Zhu, S. Roy, E. Jun, H. Y. Han, R. M. Munoz, S. Nishiwada, G. Sharma, D. Cridebring, F. Zenhausern, S. Kim, D. J. Roe, S. Darabi, I. W. Han, D. B. Evans, S. Yamada, M. J. Demeure, C. Becerra, S. A. Celinski, E. Borazanci, S. Tsai, Y. Kodera, J. O. Park, J. S. Bolton, X. Wang, S. C. Kim, D. Von Hoff and A. Goel	2022	An Exosome-based Transcriptomic Signature for Noninvasive, Early Detection of Patients With Pancreatic Ductal Adenocarcinoma: A Multicenter Cohort Study	Gastroenterology	Duplicate records removed
1548	Nakano, R., S. Nishiumi, T. Kobayashi, T. Ikegawa, Y. Kodama and M. Yoshida	2020	Possibility of detecting intraductal papillary mucinous neoplasms using metabolite biomarkers for pancreatic cancer	Biomarkers in Medicine	Duplicate records removed
1549	Nakano, R., S. Nishiumi, T. Kobayashi, T. Ikegawa, Y. Kodama and M. Yoshida	2020	Possibility of detecting intraductal papillary mucinous neoplasms using metabolite biomarkers for pancreatic cancer	Biomark Med	Duplicate records removed

1550	Nam, H., S. S. Hong, K. H. Jung, S. Kang, M. S. Park, S. Kang, H. S. Kim, V. H. Mai, J. Kim, H. Lee, W. Lee, Y. J. Suh, J. H. Lim, S. Y. Kim, S. C. Kim, S. H. Kim and S. Park	2022	A Serum Marker for Early Pancreatic Cancer With a Possible Link to Diabetes	J Natl Cancer Inst	Duplicate records removed
1551	Nam, H., S. S. Hong, K. H. Jung, S. M. Kang, M. S. Park, S. Kang, H. S. Kim, V. H. Mai, J. Kim, H. Lee, W. Lee, Y. J. Suh, J. H. Lim, S. Y. Kim, S. C. Kim, S. H. Kim and S. Park	2022	A Serum Marker for Early Pancreatic Cancer With a Possible Link to Diabetes	Jnci-Journal of the National Cancer Institute	Duplicate records removed
1552	Nannini, G., G. Meoni, A. Amedei and L. Tenori	2020	Metabolomics profile in gastrointestinal cancers: Update and future perspectives	World Journal of Gastroenterology	Duplicate records removed
1553	Nesteruk, K., I. J. M. Levink, E. de Vries, I. J. Visser, M. P. Peppelenbosch, D. L. Cahen, G. M. Fuhler and M. J. Bruno	2022	Extracellular vesicle-derived microRNAs in pancreatic juice as biomarkers for detection of pancreatic ductal adenocarcinoma	Pancreatology	Duplicate records removed
1554	Nesteruk, K., I. J. M. Levink, E. de Vries, I. J. Visser, M. P. Peppelenbosch, D. L. Cahen, G. M. Fuhler and M. J. Bruno	2022	Extracellular vesicle-derived microRNAs in pancreatic juice as biomarkers for detection of pancreatic ductal adenocarcinoma	Pancreatology	Duplicate records removed
1555	Nicoletti, A., M. Negri, M. Paratore, F. Vitale, M. E. Ainora, E. C. Nista, A. Gasbarrini, M. A. Zocco and L. Zileri Dal Verme	2023	Diagnostic and Prognostic Role of Extracellular Vesicles in Pancreatic Cancer: Current Evidence and Future Perspectives	Int J Mol Sci	Duplicate records removed
1556	Nolen, B. M., R. E. Brand, D. Prosser, L. Velikokhatnaya, P. J. Allen, H. J. Zeh, W. E. Grizzle, A. Lomakin and A. E. Lokshin	2014	Prediagnostic Serum Biomarkers as Early Detection Tools for Pancreatic Cancer in a Large Prospective Cohort Study	Plos One	Duplicate records removed
1557	Nolen, B. M., R. E. Brand, D. Prosser, L. Velikokhatnaya, P. J. Allen, H. J. Zeh, W. E. Grizzle, A. Lomakin and A. E. Lokshin	2014	Prediagnostic serum biomarkers as early detection tools for pancreatic cancer in a large prospective cohort study	PLoS ONE	Duplicate records removed

1558	O'Brien, D. P., N. S. Sandanayake, C. Jenkinson, A. Gentry-Maharaj, S. Apostolidou, E. O. Fourkala, S. Camuzeaux, O. Blyuss, R. Gunu, A. Dawnay, A. Zaikin, R. C. Smith, I. J. Jacobs, U. Menon, E. Costello, S. P. Pereira and J. F. Timms	2015	Serum CA19-9 is significantly upregulated up to 2 years before diagnosis with pancreatic cancer: Implications for early disease detection	Clinical Cancer Research	Duplicate records removed
1559	O'Brien, D. P., N. S. Sandanayake, C. Jenkinson, A. Gentry-Maharaj, S. Apostolidou, E. O. Fourkala, S. Camuzeaux, O. Blyuss, R. Gunu, A. Dawnay, A. Zaikin, R. C. Smith, I. J. Jacobs, U. Menon, E. Costello, S. P. Pereira and J. F. Timms	2015	Serum CA19-9 is significantly upregulated up to 2 years before diagnosis with pancreatic cancer: implications for early disease detection	Clin Cancer Res	Duplicate records removed
1560	Okano, K. and Y. Suzuki	2014	Strategies for early detection of resectable pancreatic cancer	World J Gastroenterol	Duplicate records removed
1561	Oliveira, B. B., B. Costa, B. Morão, S. Faias, B. Veigas, L. P. Pereira, C. Albuquerque, R. Maio, M. Cravo, A. R. Fernandes and P. V. Baptista	2023	Combining the amplification refractory mutation system and high-resolution melting analysis for KRAS mutation detection in clinical samples	Analytical and bioanalytical chemistry	Duplicate records removed
1562	Oliveira, B. B., B. Costa, B. Morão, S. Faias, B. Veigas, L. P. Pereira, C. Albuquerque, R. Maio, M. Cravo, A. R. Fernandes and P. V. Baptista	2023	Combining the amplification refractory mutation system and high-resolution melting analysis for KRAS mutation detection in clinical samples	Anal Bioanal Chem	Duplicate records removed
1563	O'Neill, R. S. and A. Stoita	2021	Biomarkers in the diagnosis of pancreatic cancer: Are we closer to finding the golden ticket?	World J Gastroenterol	Duplicate records removed
1564	Ono, Y., A. Sugitani, H. Karasaki, M. Ogata, R. Nozaki, J. Sasajima, T. Yokochi, S. Asahara, K. Koizumi, K. Ando, K. Hironaka, T. Daito and Y. Mizukami	2017	An improved digital polymerase chain reaction protocol to capture low-copy <i>KRAS</i> mutations in plasma cell-free DNA by resolving 'subsampling' issues	Molecular Oncology	Duplicate records removed
1565	Ono, Y., A. Sugitani, H. Karasaki, M. Ogata, R. Nozaki, J. Sasajima, T. Yokochi, S. Asahara, K. Koizumi, K. Ando, K. Hironaka, T. Daito and Y. Mizukami	2017	An improved digital polymerase chain reaction protocol to capture low-copy KRAS mutations in plasma cell-free DNA by resolving ‘ subsampling’ issues	Molecular Oncology	Duplicate records removed

1566	Ono, Y., A. Sugitani, H. Karasaki, M. Ogata, R. Nozaki, J. Sasajima, T. Yokochi, S. Asahara, K. Koizumi, K. Ando, K. Hironaka, T. Daito and Y. Mizukami	2017	An improved digital polymerase chain reaction protocol to capture low-copy KRAS mutations in plasma cell-free DNA by resolving 'subsampling' issues	Mol Oncol	Duplicate records removed
1567	Pal, A., A. Ojha and J. Ju	2023	Functional and Potential Therapeutic Implication of MicroRNAs in Pancreatic Cancer	Int J Mol Sci	Duplicate records removed
1568	Pan, C. H., Y. Otsuka, B. Sridharan, M. Woo, C. V. Leiton, S. Babu, M. Torrente Gonç alves, R. R. Kawalerski, J. D. K. Bai, D. K. Chang, A. V. Biankin, L. Scampavia, T. Spicer, L. F. Escobar-Hoyos and K. R. Shroyer	2020	An unbiased high-throughput drug screen reveals a potential therapeutic vulnerability in the most lethal molecular subtype of pancreatic cancer	Molecular Oncology	Duplicate records removed
1569	Pang, Y., M. V. Holmes, Z. Chen and C. Kartsonaki	2019	A review of lifestyle, metabolic risk factors, and blood-based biomarkers for early diagnosis of pancreatic ductal adenocarcinoma	J Gastroenterol Hepatol	Duplicate records removed
1570	Papapanagiotou, A., G. Sgourakis, K. Karkoulias, D. Raptis, E. Parkin, P. Brotzakis, S. Panchal and A. G. Papavassiliou	2018	Osteonectin as a screening marker for pancreatic cancer: A prospective study	J Int Med Res	Duplicate records removed
1571	Papi, M., V. Palmieri, L. Digiacomo, F. Giulimondi, S. Palchetti, G. Ciasca, G. Perini, D. Caputo, M. C. Cartillone, C. Cascone, R. Coppola, A. L. Capriotti, A. Laganà, D. Pozzi and G. Caracciolo	2019	Converting the personalized biomolecular corona of graphene oxide nanoflakes into a high-throughput diagnostic test for early cancer detection	Nanoscale	Duplicate records removed
1572	Park, J., Y. Choi, J. Namkung, S. G. Yi, H. Kim, J. Yu, Y. Kim, M. S. Kwon, W. Kwon, D. Y. Oh, S. W. Kim, S. Y. Jeong, W. Han, K. E. Lee, J. S. Heo, J. O. Park, J. K. Park, S. C. Kim, C. M. Kang, W. J. Lee, S. Lee, S. Han, T. Park, J. Y. Jang and Y. Kim	2017	Diagnostic performance enhancement of pancreatic cancer using proteomic multimarker panel	Oncotarget	Duplicate records removed
1573	Peng, H. Y., M. C. Chang, C. M. Hu, H. I. Yang, W. H. Lee and Y. T. Chang	2019	Thrombospondin-2 is a Highly Specific Diagnostic Marker and is Associated with Prognosis in Pancreatic Cancer	Annals of Surgical Oncology	Duplicate records removed
1574	Peng, H. Y., M. C. Chang, C. M. Hu, H. I. Yang, W. H. Lee and Y. T. Chang	2019	Thrombospondin-2 is a Highly Specific Diagnostic Marker and is Associated with Prognosis in Pancreatic Cancer	Ann Surg Oncol	Duplicate records removed

1575	Pietrasz, D., E. Sereni, F. Lancelotti, A. Pea, C. Luchini, G. Innamorati, R. Salvia and C. Bassi	2022	Circulating tumour DNA: a challenging innovation to develop "precision onco-surgery" in pancreatic adenocarcinoma	Br J Cancer	Duplicate records removed
1576	Prassas, I., D. Brinc, S. Farkona, F. Leung, A. Dimitromanolakis, C. C. Chrystoja, R. Brand, V. Kulasingam, I. M. Blasutig and E. P. Diamandis	2014	False biomarker discovery due to reactivity of a commercial ELISA for CUZD1 with cancer antigen CA125	Clin Chem	Duplicate records removed
1577	Pratt, E. D., R. W. Cowan, S. L. Manning, E. Qiao, H. Cameron, K. Schradle, D. M. Simeone and D. B. Zhen	2019	Multiplex Enrichment and Detection of Rare <i>KRAS</i> Mutations in Liquid Biopsy Samples using Digital Droplet Pre-Amplification	Analytical Chemistry	Duplicate records removed
1578	Pratt, E. D., R. W. Cowan, S. L. Manning, E. Qiao, H. Cameron, K. Schradle, D. M. Simeone and D. B. Zhen	2019	Multiplex Enrichment and Detection of Rare KRAS Mutations in Liquid Biopsy Samples using Digital Droplet Pre-Amplification	Anal Chem	Duplicate records removed
1579	Randeu, H., A. J. Bronkhorst, Z. Mayer, A. Oberhofer, E. Polatoglou, V. Heinemann, M. Haas, S. Boeck and S. Holdenrieder	2022	Preanalytical Variables in the Analysis of Mitochondrial DNA in Whole Blood and Plasma from Pancreatic Cancer Patients	Diagnostics	Duplicate records removed
1580	Raufi, A. G., M. S. May, M. J. Hadfield, A. A. Seyhan and W. S. El-Deiry	2023	Advances in Liquid Biopsy Technology and Implications for Pancreatic Cancer	Int J Mol Sci	Duplicate records removed
1581	Ritchie, S. A., B. Chitou, Q. Zheng, D. Jayasinghe, W. Jin, A. Mochizuki and D. B. Goodenowe	2015	Pancreatic cancer serum biomarker PC-594: Diagnostic performance and comparison to CA19-9	World Journal of Gastroenterology	Duplicate records removed
1582	Ritchie, S. A., B. Chitou, Q. Zheng, D. Jayasinghe, W. Jin, A. Mochizuki and D. B. Goodenowe	2015	Pancreatic cancer serum biomarker PC-594: Diagnostic performance and comparison to CA19-9	World J Gastroenterol	Duplicate records removed
1583	Riva, F., O. I. Dronov, D. I. Khomenko, F. Huguet, C. Louvet, P. Mariani, M. H. Stern, O. Lantz, C. Proudhon, J. Y. Pierga and F. C. Bidard	2016	Clinical applications of circulating tumor DNA and circulating tumor cells in pancreatic cancer	Mol Oncol	Duplicate records removed
1584	Roli, L., V. Pecoraro and T. Trenti	2017	Can NGAL be employed as prognostic and diagnostic biomarker in human cancers? A systematic review of current evidence	Int J Biol Markers	Duplicate records removed

1585	Ross, S. B., I. Sucandy, T. Lippert, V. Przetocki, K. Crespo, T. J. Bourdeau and A. S. Rosemurgy	2020	Genetic Profiling of Pancreatic Ductal Adenocarcinomas: Predicts Survival or Just Alphabet Soup?	Journal of the American College of Surgeons	Duplicate records removed
1586	Rossi, R. E., C. Ciafardini, D. Conte, V. Sciola and S. Massironi	2018	Chromogranin a as a marker in the follow-up of gastro-entero-pancreatic neuroendocrine neoplasms (GEP-NENS). a systematic review	Neuroendocrinology	Duplicate records removed
1587	Ruiz-Barrios, L. D. R., T. D. Pineda-Razo, G. Hernández-Flores, P. C. Ortiz-Lazareno, A. Bravo-Cuéllar, A. M. Macias-Lamas, K. J. Parra-Saavedra, L. A. Palafox-Mariscal, A. Aguilar-Lemarroy, L. F. Jave-Suárez and M. M. Villaseñor-García	2024	Expression of ornithine decarboxylase in peripheral blood mononuclear cells from patients with pancreatic adenocarcinoma: A preliminary report	Biomedical Reports	Duplicate records removed
1588	Sagami, R., T. Sato, K. Mizukami, M. Motomura, K. Okamoto, S. Fukuchi, Y. Otsuka, T. Abe, H. Ono, K. Mori, K. Wada, T. Iwaki, H. Nishikiori, K. Honda, Y. Amano and K. Murakami	2022	Diagnostic Strategy of Early Stage Pancreatic Cancer via Clinical Predictor Assessment: Clinical Indicators, Risk Factors and Imaging Findings	Diagnostics	Duplicate records removed
1589	Sakai, A., M. Suzuki, T. Kobayashi, S. Nishiumi, K. Yamanaka, Y. Hirata, T. Nakagawa, T. Azuma and M. Yoshida	2016	Pancreatic cancer screening using a multiplatform human serum metabolomics system	Biomarkers in Medicine	Duplicate records removed
1590	Sakai, A., M. Suzuki, T. Kobayashi, S. Nishiumi, K. Yamanaka, Y. Hirata, T. Nakagawa, T. Azuma and M. Yoshida	2016	Pancreatic cancer screening using a multiplatform human serum metabolomics system	Biomark Med	Duplicate records removed

1591	Sakai, Y., M. Honda, S. Matsui, O. Komori, T. Murayama, T. Fujiwara, M. Mizuno, Y. Imai, K. Yoshimura, A. Nasti, T. Wada, N. Iida, M. Kitahara, R. Horii, T. Toshikatsu, M. Nishikawa, H. Okafuji, E. Mizukoshi, T. Yamashita, T. Yamashita, K. Arai, K. Kitamura, K. Kawaguchi, H. Takatori, T. Shimakami, T. Terashima, T. Hayashi, K. Nio and S. Kaneko	2019	Development of novel diagnostic system for pancreatic cancer, including early stages, measuring mRNA of whole blood cells	Cancer Sci	Duplicate records removed
1592	Sarcina, L., F. Viola, F. Modena, R. A. Picca, P. Bollella, C. Di Franco, N. Cioffi, M. Caironi, R. Ö sterbacka, I. Esposito, G. Scamarcio, L. Torsi, F. Torricelli and E. Macchia	2022	A large-area organic transistor with 3D-printed sensing gate for noninvasive single-molecule detection of pancreatic mucinous cyst markers	Analytical and bioanalytical chemistry	Duplicate records removed
1593	Sarcina, L., F. Viola, F. Modena, R. A. Picca, P. Bollella, C. Di Franco, N. Cioffi, M. Caironi, R. Ö sterbacka, I. Esposito, G. Scamarcio, L. Torsi, F. Torricelli and E. Macchia	2022	A large-area organic transistor with 3D-printed sensing gate for noninvasive single-molecule detection of pancreatic mucinous cyst markers	Anal Bioanal Chem	Duplicate records removed
1594	Sarr, A., J. Bré, I. H. Um, T. H. Chan, P. Mullen, D. J. Harrison and P. A. Reynolds	2019	Genome-scale CRISPR/Cas9 screen determines factors modulating sensitivity to ProTide NUC-1031	Sci Rep	Duplicate records removed
1595	Sato, A., T. Masui, A. Yogo, T. Ito, K. Hirakawa, Y. Kanawaku, K. Koike and S. Uemoto	2020	Time-frequency analysis of serum with proton nuclear magnetic resonance for diagnosis of pancreatic cancer	Scientific reports	Duplicate records removed
1596	Sato, A., T. Masui, A. Yogo, T. Ito, K. Hirakawa, Y. Kanawaku, K. Koike and S. Uemoto	2020	Time-frequency analysis of serum with proton nuclear magnetic resonance for diagnosis of pancreatic cancer	Sci Rep	Duplicate records removed

1597	Sato, Y., T. Kobayashi, S. Nishiumi, A. Okada, T. Fujita, T. Sanuki, M. Kobayashi, M. Asahara, M. Adachi, A. Sakai, H. Shiomi, A. Masuda, M. Yoshida, K. Takeuchi, Y. Kodama, H. Kutsumi, K. Nagashima and K. Honda	2020	Prospective study using plasma apolipoprotein a2isoforms to screen for high-risk status of pancreatic cancer	Cancers	Duplicate records removed
1598	Scarà, S., P. Bottoni and R. Scatena (2015). CA 19-9: Biochemical and clinical aspects. 867: 247-260.	2015			Duplicate records removed
1599	Scarà, S., P. Bottoni and R. Scatena	2015	CA 19-9: Biochemical and Clinical Aspects	Adv Exp Med Biol	Duplicate records removed
1600	Schilling, K., F. Larner, A. Saad, R. Roberts, H. M. Kocher, O. Blyuss, A. N. Halliday and T. Crnogorac-Jurcevic	2020	Urine metallomics signature as an indicator of pancreatic cancer	Metallomics	Duplicate records removed
1601	Schilling, K., F. Larner, A. Saad, R. Roberts, H. M. Kocher, O. Blyuss, A. N. Halliday and T. Crnogorac-Jurcevic	2020	Urine metallomics signature as an indicator of pancreatic cancer	Metallomics	Duplicate records removed
1602	Schönemeier, B., J. Metzger, J. Klein, H. Husi, B. Bremer, N. Armbrecht, M. Dakna, J. P. Schanstra, J. Rosendahl, J. Wiegand, M. Jäger, W. Mullen, B. Breuil, R. R. Plentz, R. Lichtinghagen, K. Brand, F. Kühnel, H. Mischak, M. P. Manns and T. O. Lankisch	2016	Urinary Peptide Analysis Differentiates Pancreatic Cancer from Chronic Pancreatitis	Pancreas	Duplicate records removed
1603	Schönemeier, B., J. Metzger, J. Klein, H. Husi, B. Bremer, N. Armbrecht, M. Dakna, J. P. Schanstra, J. Rosendahl, J. Wiegand, M. Jäger, W. Mullen, B. Breuil, R. R. Plentz, R. Lichtinghagen, K. Brand, F. Kühnel, H. Mischak, M. P. Manns and T. O. Lankisch	2016	Urinary Peptide Analysis Differentiates Pancreatic Cancer From Chronic Pancreatitis	Pancreas	Duplicate records removed

1604	Schultz, N. A., C. Dehlendorff, B. V. Jensen, J. K. Bjerregaard, K. R. Nielsen, S. E. Bojesen, D. Calatayud, S. E. Nielsen, M. Yilmaz, N. H. Holländer, K. K. Andersen and J. S. Johansen	2014	MicroRNA biomarkers in whole blood for detection of pancreatic cancer	Jama	Duplicate records removed
1605	Schultz, N. A., C. Dehlendorff, B. V. Jensen, J. K. Bjerregaard, K. R. Nielsen, S. E. Bojesen, D. Calatayud, S. E. Nielsen, M. Yilmaz, N. H. Holländer, K. K. Andersen and J. S. Johansen	2014	MicroRNA biomarkers in whole blood for detection of pancreatic cancer	JAMA	Duplicate records removed
1606	Seppala, T. T., J. W. Zimmerman, R. Suri, H. Zlomke, G. D. Ivey, A. Szabolcs, C. R. Shubert, J. L. Cameron, W. R. Burns, K. J. Lafaro, J. He, C. L. Wolfgang, Y. S. Zou, L. Zheng, D. A. Tuveson, J. R. Eshlemann, D. P. Ryan, A. C. Kimmelman, T. S. Hong, D. T. Ting, E. M. Jaffee and R. A. Burkhart	2022	Precision Medicine in Pancreatic Cancer: Patient-Derived Organoid Pharmacotyping Is a Predictive Biomarker of Clinical Treatment Response	Clinical Cancer Research	Duplicate records removed
1607	Seppala, T. T., J. W. Zimmerman, R. Suri, H. Zlomke, G. D. Ivey, A. Szabolcs, C. R. Shubert, J. L. Cameron, W. R. Burns, K. J. Lafaro, J. He, C. L. Wolfgang, Y. S. Zou, L. Zheng, D. A. Tuveson, J. R. Eshlemann, D. P. Ryan, A. C. Kimmelman, T. S. Hong, D. T. Ting, E. M. Jaffee and R. A. Burkhart	2022	Precision Medicine in Pancreatic Cancer: Patient-Derived Organoid Pharmacotyping Is a Predictive Biomarker of Clinical Treatment Response	Clinical Cancer Research	Duplicate records removed

1608	Seppälä, T. T., J. W. Zimmerman, R. Suri, H. Zlomke, G. D. Ivey, A. Szabolcs, C. R. Shubert, J. L. Cameron, W. R. Burns, K. J. Lafaro, J. He, C. L. Wolfgang, Y. S. Zou, L. Zheng, D. A. Tuveson, J. R. Eshlemann, D. P. Ryan, A. C. Kimmelman, T. S. Hong, D. T. Ting, E. M. Jaffee and R. A. Burkhart	2022	Precision Medicine in Pancreatic Cancer: Patient-Derived Organoid Pharmacotyping Is a Predictive Biomarker of Clinical Treatment Response	Clin Cancer Res	Duplicate records removed
1609	Seyhan, A. A.	2023	Circulating microRNAs as Potential Biomarkers in Pancreatic Cancer-Advances and Challenges	Int J Mol Sci	Duplicate records removed
1610	Sha, M., B. Kunduzi, S. Froghi, A. Quaglia, B. Davidson and G. K. Fusai	2023	Role of circulating exosomal biomarkers and their diagnostic accuracy in pancreatic cancer	JGH Open	Duplicate records removed
1611	Shams, R., S. Saberi, M. Zali, A. Sadeghi, S. Ghafouri-Fard and H. A. Aghdaei	2020	Identification of potential microRNA panels for pancreatic cancer diagnosis using microarray datasets and bioinformatics methods	Scientific reports	Duplicate records removed
1612	Shams, R., S. Saberi, M. Zali, A. Sadeghi, S. Ghafouri-Fard and H. A. Aghdaei	2020	Identification of potential microRNA panels for pancreatic cancer diagnosis using microarray datasets and bioinformatics methods	Sci Rep	Duplicate records removed
1613	Sharma, A., H. Kandlakunta, S. J. S. Nagpal, Z. Feng, W. Hoos, G. M. Petersen and S. T. Chari	2018	Model to Determine Risk of Pancreatic Cancer in Patients With New-Onset Diabetes	Gastroenterology	Duplicate records removed
1614	Sharma, N. R., A. Perelman, A. Sharma, C. M. Zelt, C. S. Linke and K. Lowe	2017	Nonfunctional pancreatic neuroendocrine tumors: A retrospective review and early detection facilitated by EUS and novel core biopsy techniques	Gastrointestinal Endoscopy	Duplicate records removed
1615	Shen, Y., Y. Pan, L. Xu, L. Chen, L. Liu, H. Chen, Z. Chen and Z. Meng	2015	Identifying microRNA-mRNA regulatory network in gemcitabine-resistant cells derived from human pancreatic cancer cells	Tumour Biol	Duplicate records removed
1616	Shen, Y. H., Y. Pan, L. T. Xu, L. Y. Chen, L. M. Liu, H. Chen, Z. Chen and Z. Q. Meng	2015	Identifying microRNA-mRNA regulatory network in gemcitabine-resistant cells derived from human pancreatic cancer cells	Tumor Biology	Duplicate records removed
1617	Sijithra, P. C., N. Santhi and N. Ramasamy	2023	A review study on early detection of pancreatic ductal adenocarcinoma using artificial intelligence assisted diagnostic methods	Eur J Radiol	Duplicate records removed

1618	Simpson, R. E., M. T. Yip-Schneider, K. F. Flick, H. Wu, C. L. Colgate and C. M. Schmidt	2019	Pancreatic Fluid Interleukin-1β Complements Prostaglandin E2 and Serum Carbohydrate Antigen 19-9 in Prediction of Intraductal Papillary Mucinous Neoplasm Dysplasia	Pancreas	Duplicate records removed
1619	Skripova, V., R. Vlasenkova, Y. Zhou, I. Astsaturov and R. Kiyamova	2022	Identification of New Regulators of Pancreatic Cancer Cell Sensitivity to Oxaliplatin and Cisplatin	Molecules (Basel, Switzerland)	Duplicate records removed
1620	Skripova, V., R. Vlasenkova, Y. Zhou, I. Astsaturov and R. Kiyamova	2022	Identification of New Regulators of Pancreatic Cancer Cell Sensitivity to Oxaliplatin and Cisplatin	Molecules	Duplicate records removed
1621	Skulimowski, A., A. Durczyński, J. Strzelczyk and P. Hogendorf	2021	Comparison of clinical usefulness of serum Ca125 and CA19-9 in pancreatic adenocarcinoma diagnosis: meta-analysis and systematic review of literature	Biomarkers	Duplicate records removed
1622	Slater, E. P., K. Strauch, S. Rospleszcz, A. Ramaswamy, I. Esposito, G. Klöppel, E. Matthäi, K. Heeger, V. Fendrich, P. Langer and D. K. Bartsch	2014	MicroRNA-196a and -196b as potential biomarkers for the early detection of familial pancreatic cancer	Translational Oncology	Duplicate records removed
1623	Slater, E. P., K. Strauch, S. Rospleszcz, A. Ramaswamy, I. Esposito, G. Klöppel, E. Matthäi, K. Heeger, V. Fendrich, P. Langer and D. K. Bartsch	2014	MicroRNAs 196a and 196b as potential biomarkers for the early detection of familial pancreatic cancer	Langenbeck's Archives of Surgery	Duplicate records removed
1624	Słotwiński, R. and S. M. Słotwińska	2016	Diagnostic value of selected markers and apoptotic pathways for pancreatic cancer	Central European Journal of Immunology	Duplicate records removed
1625	Song, J., L. J. Sokoll, J. J. Pasay, A. L. Rubin, H. Li, D. M. Bach, D. W. Chan and Z. Zhang	2019	Identification of Serum Biomarker Panels for the Early Detection of Pancreatic Cancer	Cancer Epidemiol Biomarkers Prev	Duplicate records removed
1626	Song, J., L. J. Sokoll, J. J. Pasay, A. L. Rubin, H. Y. Li, D. M. Bach, D. W. Chan and Z. Zhang	2019	Identification of Serum Biomarker Panels for the Early Detection of Pancreatic Cancer	Cancer Epidemiology Biomarkers & Prevention	Duplicate records removed
1627	Souche, R., G. Tosato, B. Rivière, J. C. Valats, A. Debourdeau, N. Flori, D. Pourquier, J. M. Fabre, E. Assenat, J. Colinge and A. Turtoi	2022	Detection of soluble biomarkers of pancreatic cancer in endoscopic ultrasound-guided fine-needle aspiration samples	Endoscopy	Duplicate records removed

1628	Souche, R., G. Tosato, B. Rivière, J. C. Valats, A. Debourdeau, N. Flori, D. Pourquier, J. M. Fabre, E. Assenat, J. Colinge and A. Turtoi	2022	Detection of soluble biomarkers of pancreatic cancer in endoscopic ultrasound-guided fine-needle aspiration samples	Endoscopy	Duplicate records removed
1629	Suh, H. N., S. Jun, A. Y. Oh, M. Srivastava, S. Lee, C. M. Taniguchi, S. Zhang, W. S. Lee, J. Chen, B. J. Park and J. I. Park	2016	Identification of KIAA1199 as a Biomarker for Pancreatic Intraepithelial Neoplasia	Sci Rep	Duplicate records removed
1630	Sun, L. Q., L. S. Peng, J. F. Guo, F. Jiang, F. Cui, H. J. Huang and Z. D. Jin	2021	Validation of serum tumor biomarkers in predicting advanced cystic mucinous neoplasm of the pancreas	World J Gastroenterol	Duplicate records removed
1631	Suresh, V., K. Byers, U. C. Rajesh, F. Caiazza, G. A. Zhu, C. S. Craik, K. Kirkwood, V. J. Davisson and D. A. Sheik	2022	Translation of a Protease Turnover Assay for Clinical Discrimination of Mucinous Pancreatic Cysts	Diagnostics	Duplicate records removed
1632	Suzuki, M., S. Nishiumi, T. Kobayashi, A. Sakai, Y. Iwata, T. Uchikata, Y. Izumi, T. Azuma, T. Bamba and M. Yoshida	2017	Use of on-line supercritical fluid extraction-supercritical fluid chromatography/tandem mass spectrometry to analyze disease biomarkers in dried serum spots compared with serum analysis using liquid chromatography/tandem mass spectrometry	Rapid communications in mass spectrometry : RCM	Duplicate records removed
1633	Suzuki, M., S. Nishiumi, T. Kobayashi, A. Sakai, Y. Iwata, T. Uchikata, Y. Izumi, T. Azuma, T. Bamba and M. Yoshida	2017	Use of on-line supercritical fluid extraction-supercritical fluid chromatography/tandem mass spectrometry to analyze disease biomarkers in dried serum spots compared with serum analysis using liquid chromatography/tandem mass spectrometry	Rapid Commun Mass Spectrom	Duplicate records removed
1634	Suzuki, R., H. Tamura, R. Honma, N. Konno, H. Irie, T. Takagi, M. Sugimoto, H. Asama, Y. Sato, O. Yoshinori, J. Nakamura, M. Takasumi, T. Kato, M. Hashimoto, T. Hikichi, J. I. Imai, S. Watanabe and H. Ohira	2020	A blood-based gene-expression scoring system for cancer screening in patients with branch-duct intraductal papillary mucinous neoplasms	Anticancer Research	Duplicate records removed

1635	Suzuki, R., H. Tamura, R. Honma, N. Konno, H. Irie, T. Takagi, M. Sugimoto, H. Asama, Y. Sato, O. Yoshinori, J. Nakamura, M. Takasumi, T. Kato, M. Hashimoto, T. Hikichi, J. I. Imai, S. Watanabe and H. Ohira	2020	A Blood-based Gene-expression Scoring System for Cancer Screening in Patients With Branch-duct Intraductal Papillary Mucinous Neoplasms	Anticancer Res	Duplicate records removed
1636	Tang, H., S. Singh, K. Partyka, D. Kletter, P. Hsueh, J. Yadav, E. Ensink, M. Bern, G. Hostetter, D. Hartman, Y. Huang, R. E. Brand and B. B. Haab	2015	Glycan motif profiling reveals plasma sialyl-lewis x elevations in pancreatic cancers that are negative for sialyl-lewis A	Mol Cell Proteomics	Duplicate records removed
1637	Tang, H. Y., S. Singh, K. Partyka, D. Kletter, P. Hsueh, J. Yadav, E. Ensink, M. Bern, G. Hostetter, D. Hartman, Y. Huang, R. E. Brand and B. B. Haab	2015	Glycan Motif Profiling Reveals Plasma Sialyl-Lewis X Elevations in Pancreatic Cancers That Are Negative for Sialyl-Lewis A	Molecular & Cellular Proteomics	Duplicate records removed
1638	Tanţău, A., D. C. Leucuţa, M. Tanţău, E. Boţan, R. Zaharie, A. Mândruţiu and I. C. Tomuleasa	2021	Inflammation, Tumoral Markers and Interleukin-17, -10, and -6 Profiles in Pancreatic Adenocarcinoma and Chronic Pancreatitis	Dig Dis Sci	Duplicate records removed
1639	Tarasiuk, A., T. Mackiewicz, E. Małecka-Panas and J. Fichna	2021	Biomarkers for early detection of pancreatic cancer - miRNAs as a potential diagnostic and therapeutic tool?	Cancer Biol Ther	Duplicate records removed
1640	Ulutas, K. T. and I. S. Sarici	2016	Could neutrophil/lymphocyte ratio, platelet/lymphocyte ratio, and mean platelet volume serve as potential biomarkers for detection of resectable pancreas ca?	International Journal of Clinical and Experimental Medicine	Duplicate records removed
1641	Urayama, S.	2015	Pancreatic cancer early detection: expanding higher-risk group with clinical and metabolomics parameters	World J Gastroenterol	Duplicate records removed
1642	Vanek, P., M. Eid, R. Psar, V. Zoundjiekpon, O. Urban and L. Kunovský	2022	Current trends in the diagnosis of pancreatic cancer	Vnitr Lek	Duplicate records removed
1643	Vellan, C. J., J. J. Jayapalan, B. K. Yoong, A. Abdul-Aziz, S. Mat-Junit and P. Subramanian	2022	Application of Proteomics in Pancreatic Ductal Adenocarcinoma Biomarker Investigations: A Review	Int J Mol Sci	Duplicate records removed

1644	Velstra, B., M. A. Vonk, B. A. Bonsing, B. J. Mertens, S. Nicolardi, A. Huijbers, H. Vasen, A. M. Deelder, W. E. Mesker, Y. E. M. van der Burgt and R. Tollenaar	2015	Serum peptide signatures for pancreatic cancer based on mass spectrometry: a comparison to CA19-9 levels and routine imaging techniques	Journal of Cancer Research and Clinical Oncology	Duplicate records removed
1645	Velstra, B., M. A. Vonk, B. A. Bonsing, B. J. Mertens, S. Nicolardi, A. Huijbers, H. Vasen, A. M. Deelder, W. E. Mesker, Y. E. M. van der Burgt and R. A. E. M. Tollenaar	2015	Serum peptide signatures for pancreatic cancer based on mass spectrometry: a comparison to CA19-9 levels and routine imaging techniques	Journal of Cancer Research and Clinical Oncology	Duplicate records removed
1646	Verel-Yilmaz, Y., J. P. Fernández, A. Schä fer, S. Nevermann, L. Cook, N. Gercke, F. Helmprobst, C. Jaworek, E. P. von Strandmann, A. Pagenstecher, D. K. Bartsch, J. W. Bartsch and E. P. Slater	2021	Extracellular Vesicle-Based Detection of Pancreatic Cancer	Frontiers in Cell and Developmental Biology	Duplicate records removed
1647	Vila-Navarro, E., S. Duran-Sanchon, M. Vila-Casadesús, L. Moreira, A. Gins, M. Cuatrecasas, J. Josè Lozano, L. Bujanda, A. Castells and M. Gironella	2019	Novel circulating mirna signatures for early detection of pancreatic neoplasia	Clinical and Translational Gastroenterology	Duplicate records removed
1648	Visani, M., G. Acquaviva, S. Fiorino, M. L. Bacchi Reggiani, M. Masetti, E. Franceschi, A. Fornelli, E. Jovine, C. Fabbri, A. A. Brandes, G. Tallini, A. Pession and D. de Biase	2015	Contribution of microRNA analysis to characterisation of pancreatic lesions: a review	J Clin Pathol	Duplicate records removed
1649	Visser, I. J., I. J. M. Levink, M. P. Peppelenbosch, G. M. Fuhler, M. J. Bruno and D. L. Cahen	2022	Systematic review and meta-analysis: Diagnostic performance of DNA alterations in pancreatic juice for the detection of pancreatic cancer	Pancreatology	Duplicate records removed
1650	Wang, H., X. Li, L. A. Lai, T. A. Brentnall, D. W. Dawson, K. A. Kelly, R. Chen and S. Pan	2021	X-aptamers targeting Thy-1 membrane glycoprotein in pancreatic ductal adenocarcinoma	Biochimie	Duplicate records removed
1651	Wang, H. Y., X. Li, L. A. Lai, T. A. Brentnall, D. W. Dawson, K. A. Kelly, R. Chen and S. Pan	2021	X-aptamers targeting Thy-1 membrane glycoprotein in pancreatic ductal adenocarcinoma	Biochimie	Duplicate records removed

1652	Wang, J., M. Raimondo, S. Guha, J. Y. Chen, L. X. Diao, X. Q. Dong, M. B. Wallace, A. M. Killary, M. L. Frazier, T. A. Woodward, J. Wang and S. Sen	2014	Circulating microRNAs in Pancreatic Juice as Candidate Biomarkers of Pancreatic Cancer	Journal of Cancer	Duplicate records removed
1653	Wang, L., A. R. Luedtke and Y. Huang	2020	Assessing the incremental value of new biomarkers based on OR rules	Biostatistics (Oxford, England)	Duplicate records removed
1654	Wang, L., A. R. Luedtke and Y. Huang	2020	Assessing the incremental value of new biomarkers based on OR rules	Biostatistics	Duplicate records removed
1655	Wang, L., L. Wang, X. Sun, L. Fu, X. Wang, X. Wang, L. Chen and Y. Huang	2024	Detection of uridine diphosphate glucuronosyltransferase 1A1 for pancreatic cancer imaging and treatment via a "turn-on" fluorescent probe	Analyst	Duplicate records removed
1656	Wang, L., Z. Wu, C. Xu and H. Ye	2023	Ferroptosis-related genes prognostic signature for pancreatic cancer and immune infiltration: potential biomarkers for predicting overall survival	J Cancer Res Clin Oncol	Duplicate records removed
1657	Wang, L., Z. X. Wu, C. Xu and H. Ye	2023	Ferroptosis-related genes prognostic signature for pancreatic cancer and immune infiltration: potential biomarkers for predicting overall survival	Journal of Cancer Research and Clinical Oncology	Duplicate records removed
1658	Wang, S., X. Chen and M. Tang	2014	Quantitative assessment of the diagnostic role of human telomerase activity from pancreatic juice in pancreatic cancer	Tumor Biology	Duplicate records removed
1659	Wang, S., X. Chen and M. Tang	2014	Quantitative assessment of the diagnostic role of human telomerase activity from pancreatic juice in pancreatic cancer	Tumour Biol	Duplicate records removed
1660	Wang, S., X. Chen and M. Tang	2014	Quantitative assessment of the diagnostic role of MUC1 in pancreatic ductal adenocarcinoma	Tumour Biol	Duplicate records removed
1661	Wang, S., S. Wen, P. Guo, H. Liu, J. Feng and H. Huang	2020	Understanding metabolomic characteristics of pancreatic ductal adenocarcinoma by HR-MAS NMR detection of pancreatic tissues	J Pharm Biomed Anal	Duplicate records removed
1662	Wang, X., Y. Li, H. Tian, J. Qi, M. Li, C. Fu, F. Wu, Y. Wang, D. Cheng, W. Zhao, C. Zhang, T. Wang, J. Rao and W. Zhang	2014	Macrophage inhibitory cytokine 1 (MIC-1/GDF15) as a novel diagnostic serum biomarker in pancreatic ductal adenocarcinoma	BMC Cancer	Duplicate records removed
1663	Wang, X. B., Y. F. Li, H. M. Tian, J. Qi, M. Li, C. Fu, F. Wu, Y. Wang, D. W. Cheng, W. Y. Zhao, C. Zhang, T. Wang, J. Y. Rao and W. Zhang	2014	Macrophage inhibitory cytokine 1 (MIC-1/GDF15) as a novel diagnostic serum biomarker in pancreatic ductal adenocarcinoma	Bmc Cancer	Duplicate records removed
1664	Wang, Z. Y., X. Q. Ding, H. Zhu, R. X. Wang, X. R. Pan and J. H. Tong	2019	<i>KRAS</i> Mutant Allele Fraction in Circulating Cell-Free DNA Correlates With Clinical Stage in Pancreatic Cancer Patients	Frontiers in Oncology	Duplicate records removed

1665	Watcharanurak, P., A. Mutirangura, V. Aksornkitti, N. Bhummaphan and C. Puttipanyalears	2024	The high FKBP1A expression in WBCs as a potential screening biomarker for pancreatic cancer	Sci Rep	Duplicate records removed
1666	Wei, L., K. Yao, S. Gan and Z. Suo	2018	Clinical utilization of serum- or plasma-based miRNAs as early detection biomarkers for pancreatic cancer: A meta-analysis up to now	Medicine (Baltimore)	Duplicate records removed
1667	Wiewiora, M., J. Jopek, E. Świętochowska, M. Grynkiewicz and J. Piecuch	2024	Evaluations of the combined use of blood- and tissue-based protein biomarkers for pancreatic cancer	Clin Hemorheol Microcirc	Duplicate records removed
1668	Wiewiora, M., J. Jopek, E. Świętochowska, M. Grynkiewicz and J. Piecuch	2024	Evaluations of the combined use of blood- and tissue-based protein biomarkers for pancreatic cancer	Clinical Hemorheology and Microcirculation	Duplicate records removed
1669	Willms, A., C. Müller, H. Julich, N. Klein, R. Schwab, C. Güsgen, I. Richardsen, S. Schaaf, M. Krawczyk, M. Krawczyk, F. Lammert, D. Schuppan, V. Lukacs-Kornek and M. Kornek	2016	Tumour-associated circulating microparticles: A novel liquid biopsy tool for screening and therapy monitoring of colorectal carcinoma and other epithelial neoplasia	Oncotarget	Duplicate records removed
1670	Willms, A., C. Müller, H. Julich, N. Klein, R. Schwab, C. Güsgen, I. Richardsen, S. Schaaf, M. Krawczyk, F. Lammert, D. Schuppan, V. Lukacs-Kornek and M. Kornek	2016	Tumour-associated circulating microparticles: A novel liquid biopsy tool for screening and therapy monitoring of colorectal carcinoma and other epithelial neoplasia	Zeitschrift fur Gastroenterologie	Duplicate records removed
1671	Wolrab, D., R. Jirásko, E. Cífková, M. Höring, D. Mei, M. Chocholoušková, O. Peterka, J. Idkowiak, T. Hrnčiarová, L. Kuchař, R. Ahrends, R. Brumarová, D. Friedecký, G. Vivo-Truyols, P. Škrha, J. Škrha, R. Kučera, B. Melichar, G. Liebisch, R. Burkhardt, M. R. Wenk, A. Cazenave-Gassiot, P. Karásek, I. Novotný, K. Greplová, R. Hrstka and M. Holčapek	2022	Lipidomic profiling of human serum enables detection of pancreatic cancer	Nature Communications	Duplicate records removed

1672	Wolrab, D., R. Jirásko, E. Cífková, M. Höring, D. Mei, M. Chocholoušková, O. Peterka, J. Idkowiak, T. Hrnčiarová, L. Kuchař, R. Ahrends, R. Brumarová, D. Friedecký, G. Vivo-Truyols, P. Škrha, J. Škrha, R. Kučera, B. Melichar, G. Liebisch, R. Burkhardt, M. R. Wenk, A. Cazenave-Gassiot, P. Karásek, I. Novotný, K. Greplová, R. Hrstka and M. Holčapek	2022	Lipidomic profiling of human serum enables detection of pancreatic cancer	Nat Commun	Duplicate records removed
1673	Wong, H. L., K. Bushell, J. Karasinska, S. Arthur, R. Morin, D. F. Schaeffer and D. J. Renouf	2016	Clinical utility of circulating tumor DNA (ctDNA) in resectable pancreatic ductal adenocarcinoma (PDAC)	Journal of Clinical Oncology	Duplicate records removed
1674	Wong, H. L., K. Bushell, J. Karasinska, S. Arthur, P. Pararajalingam, R. Morin, D. F. Schaeffer and D. J. Renouf	2016	Clinical utility of circulating tumor DNA (ctDNA) in resectable pancreatic ductal adenocarcinoma (PDAC)	Annals of Oncology	Duplicate records removed
1675	Wu, H., S. Guo, X. Liu, Y. Li, Z. Su, Q. He, X. Liu, Z. Zhang, L. Yu, X. Shi, S. Gao, H. Wang, Y. Pan, C. Ma, R. Liu, M. Dai, G. Jin and Z. Liang	2022	Noninvasive detection of pancreatic ductal adenocarcinoma using the methylation signature of circulating tumour DNA	BMC Med	Duplicate records removed
1676	Wu, W., X. Xia, C. Cheng, L. Niu, J. Wu and Y. Qian	2021	Serum Soluble PD-L1, PD-L2, and B7-H5 as Potential Diagnostic Biomarkers of Human Pancreatic Cancer	Clin Lab	Duplicate records removed
1677	Wu, W. G., X. P. Xia, C. Cheng, L. L. Niu, J. G. Wu and Y. Qian	2021	Serum Soluble PD-L1, PD-L2, and B7-H5 as Potential Diagnostic Biomarkers of Human Pancreatic Cancer	Clinical Laboratory	Duplicate records removed
1678	Wu, Y. H., Y. P. Hung, N. C. Chiu, R. C. Lee, C. P. Li, Y. Chao, Y. M. Shyr, S. E. Wang, S. C. Chen, S. H. Lin, Y. H. Chen, Y. M. Kang, S. M. Hsu, S. H. Yen, J. Y. Wu, K. D. Lee, H. E. Tseng, J. R. Tsai, J. H. Tang, J. F. Chiou, T. Burnouf, Y. J. Chen, P. Y. Wang and L. S. Lu	2022	Correlation between drug sensitivity profiles of circulating tumour cell-derived organoids and clinical treatment response in patients with pancreatic ductal adenocarcinoma	Eur J Cancer	Duplicate records removed
1679	Xie, G., L. Lu, Y. Qiu, Q. Ni, W. Zhang, Y. T. Gao, H. A. Risch, H. Yu and W. Jia	2015	Plasma metabolite biomarkers for the detection of pancreatic cancer	J Proteome Res	Duplicate records removed

1680	Xie, G. X., L. G. Lu, Y. P. Qiu, Q. X. Ni, W. Zhang, Y. T. Gao, H. A. Risch, H. Yu and W. Jia	2015	Plasma Metabolite Biomarkers for the Detection of Pancreatic Cancer	Journal of Proteome Research	Duplicate records removed
1681	Xie, J., X. Zhou, R. Wang, J. Zhao, J. Tang, Q. Zhang, Y. Du and Y. Pang	2021	Identification of potential diagnostic biomarkers in MMPs for pancreatic carcinoma	Medicine (Baltimore)	Duplicate records removed
1682	Xie, J. H., X. Z. Zhou, R. Wang, J. L. Zhao, J. Tang, Q. C. Zhang, Y. Q. Du and Y. N. Pang	2021	Identification of potential diagnostic biomarkers in MMPs for pancreatic carcinoma	Medicine	Duplicate records removed
1683	Xie, W., M. Chu, G. Song, Z. Zuo, Z. Han, C. Chen, Y. Li and Z. W. Wang	2022	Emerging roles of long noncoding RNAs in chemoresistance of pancreatic cancer	Semin Cancer Biol	Duplicate records removed
1684	Xie, W. K., M. Chu, G. D. Song, Z. Y. Zuo, Z. Han, C. B. Chen, Y. Y. Li and Z. W. Wang	2022	Emerging roles of long noncoding RNAs in chemoresistance of pancreatic cancer	Seminars in Cancer Biology	Duplicate records removed
1685	Xie, Z., X. Yin, B. Gong, W. Nie, B. Wu, X. Zhang, J. Huang, P. Zhang, Z. Zhou and Z. Li	2015	Salivary microRNAs show potential as a noninvasive biomarker for detecting resectable pancreatic cancer	Cancer Prev Res (Phila)	Duplicate records removed
1686	Xie, Z. J., X. Y. Yin, B. Gong, W. J. Nie, B. Wu, X. C. Zhang, J. Huang, P. Y. Zhang, Z. W. Zhou and Z. J. Li	2015	Salivary microRNAs Show Potential as a Noninvasive Biomarker for Detecting Resectable Pancreatic Cancer	Cancer Prevention Research	Duplicate records removed
1687	Xiong, G., M. Feng, G. Yang, S. Zheng, X. Song, Z. Cao, L. You, L. Zheng, Y. Hu, T. Zhang and Y. Zhao	2017	The underlying mechanisms of non-coding RNAs in the chemoresistance of pancreatic cancer	Cancer Lett	Duplicate records removed
1688	Xu, W., M. Zhang, L. Liu, M. Yin, C. Xu and Z. Weng	2022	Association of mucin family members with prognostic significance in pancreatic cancer patients: A meta-analysis	PLoS One	Duplicate records removed
1689	Xu, W., M. Zhang, L. Liu, M. Y. Yin, C. F. Xu and Z. Weng	2022	Association of mucin family members with prognostic significance in pancreatic cancer patients: A meta-analysis	Plos One	Duplicate records removed
1690	Xu, X., Y. Xiao, B. Hong, B. Hao and Y. Qian	2019	Combined detection of CA19-9 and B7-H4 in the diagnosis and prognosis of pancreatic cancer	Cancer Biomark	Duplicate records removed
1691	Xu, Y., T. Qin, J. Li, X. Wang, C. Gao, C. Xu, J. Hao, J. Liu, S. Gao and H. Ren	2017	Detection of Circulating Tumor Cells Using Negative Enrichment Immunofluorescence and an In Situ Hybridization System in Pancreatic Cancer	Int J Mol Sci	Duplicate records removed
1692	Xu, Y., T. Qin, J. Li, X. C. Wang, C. T. Gao, C. Xu, J. H. Hao, J. C. Liu, S. Gao and H. Ren	2017	Detection of Circulating Tumor Cells Using Negative Enrichment Immunofluorescence and an In Situ Hybridization System in Pancreatic Cancer	International Journal of Molecular Sciences	Duplicate records removed

1693	Yamada, K., K. Higashi, H. Nagahori and K. Saito	2020	Circulating natural antibodies against 3’-sialyllactose complement the diagnostic performance of CA19-9 for the early detection of pancreatic ductal adenocarcinoma	Cancer Biomarkers	Duplicate records removed
1694	Yamada, K., K. Higashi, H. Nagahori and K. Saito	2020	Circulating natural antibodies against 3'-sialyllactose complement the diagnostic performance of CA19-9 for the early detection of pancreatic ductal adenocarcinoma	Cancer Biomark	Duplicate records removed
1695	Yang, H., W. Li, L. Ren, Y. Yang, Y. Zhang, B. Ge, S. Li, X. Zheng, J. Liu, S. Zhang, G. Du, B. O. Tang, H. Wang and J. Wang	2023	Progress on diagnostic and prognostic markers of pancreatic cancer	Oncol Res	Duplicate records removed
1696	Yang, H., W. Li, L. W. Ren, Y. H. Yang, Y. Z. Zhang, B. B. Ge, S. Li, X. J. Zheng, J. Y. Liu, S. Zhang, G. H. Du, B. Tang, H. Q. Wang and J. H. Wang	2023	Progress on diagnostic and prognostic markers of pancreatic cancer	Oncology Research	Duplicate records removed
1697	Yang, J., R. Xu, C. Wang, J. Qiu, B. Ren and L. You	2021	Early screening and diagnosis strategies of pancreatic cancer: a comprehensive review	Cancer Commun (Lond)	Duplicate records removed
1698	Yang, K. S., D. Ciprani, A. O'Shea, A. S. Liss, R. Yang, S. Fletcher-Mercaldo, M. Mino-Kenudson, C. Fernández-del Castillo and R. Weissleder	2021	Extracellular Vesicle Analysis Allows for Identification of Invasive IPMN	Gastroenterology	Duplicate records removed
1699	Yang, K. S., D. Ciprani, A. O'Shea, A. S. Liss, R. Yang, S. Fletcher-Mercaldo, M. Mino-Kenudson, C. Fernández-Del Castillo and R. Weissleder	2021	Extracellular Vesicle Analysis Allows for Identification of Invasive IPMN	Gastroenterology	Duplicate records removed
1700	Yang, K. S., H. Im, S. Hong, I. Pergolini, A. F. Del Castillo, R. Wang, S. Clardy, C. H. Huang, C. Pille, S. Ferrone, R. Yang, C. M. Castro, H. Lee, C. F. Del Castillo and R. Weissleder	2017	Multiparametric plasma EV profiling facilitates diagnosis of pancreatic malignancy	Science Translational Medicine	Duplicate records removed
1701	Yang, K. S., H. Im, S. Hong, I. Pergolini, A. F. Del Castillo, R. Wang, S. Clardy, C. H. Huang, C. Pille, S. Ferrone, R. Yang, C. M. Castro, H. Lee, C. F. Del Castillo and R. Weissleder	2017	Multiparametric plasma EV profiling facilitates diagnosis of pancreatic malignancy	Sci Transl Med	Duplicate records removed

1702	Yang, M. and C. Y. Zhang	2021	Diagnostic biomarkers for pancreatic cancer: An update	World J Gastroenterol	Duplicate records removed
1703	Yang, Y., S. Yan, H. Tian and Y. Bao	2018	Macrophage inhibitory cytokine-1 versus carbohydrate antigen 19-9 as a biomarker for diagnosis of pancreatic cancer: A PRISMA-compliant meta-analysis of diagnostic accuracy studies	Medicine (Baltimore)	Duplicate records removed
1704	Ye, H., H. Wang, P. Wang, C. H. Song, K. J. Wang, L. P. Dai, J. X. Shi, X. X. Liu, C. Q. Sun, X. Wang, Y. Peng, X. B. Chen and J. Y. Zhang	2019	Systematic review: exosomal microRNAs associated with pancreatic cancer for early detection and prognosis	Eur Rev Med Pharmacol Sci	Duplicate records removed
1705	Yi, N., X. Zhao, J. Ji, M. Xu, Y. Jiao, T. Qian, S. Zhu, F. Jiang, J. Chen and M. Xiao	2020	Serum galectin-3 as a biomarker for screening, early diagnosis, prognosis and therapeutic effect evaluation of pancreatic cancer	J Cell Mol Med	Duplicate records removed
1706	Yi, N., X. Y. Zhao, J. Ji, M. X. Xu, Y. J. Jiao, T. Y. Qian, S. Z. Zhu, F. Jiang, J. H. Chen and M. B. Xiao	2020	Serum galectin-3 as a biomarker for screening, early diagnosis, prognosis and therapeutic effect evaluation of pancreatic cancer	Journal of Cellular and Molecular Medicine	Duplicate records removed
1707	Ying, L., A. Sharma, A. Chhoda, N. Ruzgar, N. Hasan, R. Kwak, C. L. Wolfgang, T. H. Wang, J. W. Kunstman, R. R. Salem, L. D. Wood, C. Iacobuzio-Donahue, E. B. Schneider, J. J. Farrell and N. Ahuja	2021	Methylation-based cell-free DNA signature for early detection of pancreatic cancer	Pancreas	Duplicate records removed
1708	Ying, L., A. Sharma, A. Chhoda, N. Ruzgar, N. Hasan, R. Kwak, C. L. Wolfgang, T. H. Wang, J. W. Kunstman, R. R. Salem, L. D. Wood, C. Iacobuzio-Donahue, E. B. Schneider, J. J. Farrell and N. Ahuja	2021	Methylation-based Cell-free DNA Signature for Early Detection of Pancreatic Cancer	Pancreas	Duplicate records removed
1709	Yip-Schneider, M. T., R. A. Carr, H. Wu and C. M. Schmidt	2017	Prostaglandin E(2): A Pancreatic Fluid Biomarker of Intraductal Papillary Mucinous Neoplasm Dysplasia	J Am Coll Surg	Duplicate records removed
1710	Yip-Schneider, M. T., R. Muraru, R. C. Kim, H. H. Wu, S. Sherman, A. Gutta, M. A. Al-Haddad, J. M. Dewitt and C. M. Schmidt	2023	EUS-guided fine needle aspiration-based clues to mistaken or uncertain identity: serous pancreatic cysts	HPB (Oxford)	Duplicate records removed

1711	Yip-Schneider, M. T., H. Wu, H. R. Allison, J. J. Easler, S. Sherman, M. A. Al-Haddad, J. M. Dewitt and C. M. Schmidt	2021	Biomarker Risk Score Algorithm and Preoperative Stratification of Patients with Pancreatic Cystic Lesions	J Am Coll Surg	Duplicate records removed
1712	Yip-Schneider, M. T., H. Wu, R. P. Dumas, B. A. Hancock, N. Agaram, M. Radovich and C. M. Schmidt	2014	Vascular endothelial growth factor, a novel and highly accurate pancreatic fluid biomarker for serous pancreatic cysts	J Am Coll Surg	Duplicate records removed
1713	Young, M. R., P. D. Wagner, S. Ghosh, J. A. Rinaudo, S. G. Baker, K. S. Zaret, M. Goggins and S. Srivastava	2018	Validation of Biomarkers for Early Detection of Pancreatic Cancer: Summary of the Alliance of Pancreatic Cancer Consortia for Biomarkers for Early Detection Workshop	Pancreas	Duplicate records removed
1714	Young, M. R., P. D. Wagner, S. Ghosh, J. A. Rinaudo, S. G. Baker, K. S. Zaret, M. Goggins and S. Srivastava	2018	Validation of Biomarkers for Early Detection of Pancreatic Cancer: Summary of The Alliance of Pancreatic Cancer Consortia for Biomarkers for Early Detection Workshop	Pancreas	Duplicate records removed
1715	Yu, J., A. Ploner, M. Kordes, M. Löhr, M. Nilsson, M. E. L. de Maturana, L. Estudillo, H. Renz, A. Carrato, X. Molero, F. X. Real, N. Malats and W. Ye	2021	Plasma protein biomarkers for early detection of pancreatic ductal adenocarcinoma	Int J Cancer	Duplicate records removed
1716	Yu, P., S. Luo, J. Cai, J. Li and C. Peng	2022	ERAP2 as a potential biomarker for predicting gemcitabine response in patients with pancreatic cancer	Aging (Albany NY)	Duplicate records removed
1717	Yu, P., S. F. Luo, J. X. Cai, J. Li and C. Peng	2022	ERAP2 as a potential biomarker for predicting gemcitabine response in patients with pancreatic cancer	Aging-Us	Duplicate records removed
1718	Yu, Y., Y. Tong, A. Zhong, Y. Wang, R. Lu and L. Guo	2020	Identification of Serum microRNA-25 as a novel biomarker for pancreatic cancer	Medicine (Baltimore)	Duplicate records removed
1719	Yu, Y. W., Y. Tong, A. L. Zhong, Y. C. Wang, R. Q. Lu and L. Guo	2020	Identification of Serum microRNA-25 as a novel biomarker for pancreatic cancer	Medicine	Duplicate records removed
1720	Yu, Z., Y. Yang, W. Fang, P. Hu, Y. Liu and J. Shi	2023	Dual Tumor Exosome Biomarker Co-recognitions Based Nanoliquid Biopsy for the Accurate Early Diagnosis of Pancreatic Cancer	ACS Nano	Duplicate records removed
1721	Yu, Z. G., Y. Yang, W. M. Fang, P. Hu, Y. B. Liu and J. L. Shi	2023	Dual Tumor Exosome Biomarker Co-recognitions Based Nanoliquid Biopsy for the Accurate Early Diagnosis of Pancreatic Cancer	Acs Nano	Duplicate records removed
1722	Yuan, C., J. Fang, X. Luo, Y. Zhang, G. Huang, X. Zeng, K. Xia, M. Li, X. Chen, X. Yang, M. L. de la Chapelle and W. Fu	2022	One-step isothermal amplification strategy for microRNA specific and ultrasensitive detection based on nicking-assisted entropy-driven DNA circuit triggered exponential amplification reaction	Anal Chim Acta	Duplicate records removed

1723	Yuan, C. J., J. Fang, X. Z. Luo, Y. Zhang, G. R. Huang, X. J. Zeng, K. Xia, M. Y. Li, X. P. Chen, X. Yang, M. L. de la Chapelle and W. L. Fu	2022	One-step isothermal amplification strategy for microRNA specific and ultrasensitive detection based on nicking-assisted entropy-driven DNA circuit triggered exponential amplification reaction	Analytica Chimica Acta	Duplicate records removed
1724	Zeng, D., Z. Wang, Z. Meng, P. Wang, L. San, W. Wang, A. Aldalbahi, L. Li, J. Shen and X. Mi	2017	DNA Tetrahedral Nanostructure-Based Electrochemical miRNA Biosensor for Simultaneous Detection of Multiple miRNAs in Pancreatic Carcinoma	ACS Appl Mater Interfaces	Duplicate records removed
1725	Zeng, D. D., Z. H. Wang, Z. Q. Meng, P. Wang, L. L. San, W. Wang, A. Aldalbahi, L. Li, J. W. Shen and X. Q. Mi	2017	DNA Tetrahedral Nanostructure-Based Electrochemical miRNA Biosensor for Simultaneous Detection of Multiple miRNAs in Pancreatic Carcinoma	Acs Applied Materials & Interfaces	Duplicate records removed
1726	Zhang, C., M. Alhamdani, A. Bauer, L. Peng and J. Hoheisel	2020	The identification of biomarker for the earlydiagnosis of IPMN associated PDAC based on serumprotein profiling	Cancer Research	Duplicate records removed
1727	Zhang, M., Y. Zhang, J. Fu and L. Zhang	2019	Serum CA125 levels are decreased in rectal cancer but increased in fibrosis-associated diseases and in most types of cancers	Prog Mol Biol Transl Sci	Duplicate records removed
1728	Zhang, P., M. Zou, X. Wen, F. Gu, J. Li, G. Liu, J. Dong, X. Deng, J. Gao, X. Li, X. Jia, Z. Dong, L. Chen, Y. Wang and Y. Tian	2014	Development of serum parameters panels for the early detection of pancreatic cancer	Int J Cancer	Duplicate records removed
1729	Zhang, P. J., M. Zou, X. Y. Wen, F. Gu, J. Li, G. X. Liu, J. X. Dong, X. X. Deng, J. Gao, X. L. Li, X. W. Jia, Z. N. Dong, L. N. Chen, Y. Wang and Y. P. Tian	2014	Development of serum parameters panels for the early detection of pancreatic cancer	International Journal of Cancer	Duplicate records removed
1730	Zhang, Q., S. Chen, L. Zeng, Y. Chen, G. Lian, C. Qian, J. Li, R. Xie and K. H. Huang	2017	New developments in the early diagnosis of pancreatic cancer	Expert Rev Gastroenterol Hepatol	Duplicate records removed
1731	Zhang, Q., M. Ye, C. Lin, M. Hu, Y. Wang, Y. Lou, Q. Kong, J. Zhang, J. Li, Y. Zhang, T. Yang, X. Sun, W. Yao, Y. Hua, H. Huang, M. Xu, X. Wang, X. Yu, W. Tao, R. Liu, Y. Gao, T. Wang, J. Wang, X. Wei, J. Wu, Z. Yu, C. Zhang, C. Yu, X. Bai and T. Liang	2023	Mass cytometry-based peripheral blood analysis as a novel tool for early detection of solid tumours: a multicentre study	Gut	Duplicate records removed

1732	Zhang, W. H., W. Q. Wang, X. Han, H. L. Gao, T. J. Li, S. S. Xu, S. Li, H. X. Xu, H. Li, L. Y. Ye, X. Lin, C. T. Wu, J. Long, X. J. Yu and L. Liu	2020	Advances on Diagnostic Biomarkers of Pancreatic Ductal Adenocarcinoma: A Systems Biology Perspective	Pancreas	Duplicate records removed
1733	Zhang, Y., L. Qiu, Y. Wang, X. Qin and Z. Li	2014	High-throughput and high-sensitivity quantitative analysis of serum unsaturated fatty acids by chip-based nanoelectrospray ionization-Fourier transform ion cyclotron resonance mass spectrometry: early stage diagnostic biomarkers of pancreatic cancer	Analyst	Duplicate records removed
1734	Zhao, B., B. Zhao and F. Chen	2022	Diagnostic value of serum carbohydrate antigen 19-9 in pancreatic cancer: a systematic review and meta-analysis	European journal of gastroenterology & hepatology	Duplicate records removed
1735	Zhao, B., B. Zhao and F. Chen	2022	Diagnostic value of serum carbohydrate antigen 19-9 in pancreatic cancer: a systematic review and meta-analysis	Eur J Gastroenterol Hepatol	Duplicate records removed
1736	Zhao, J., Y. Liang, Q. Yin, S. Liu, Q. Wang, Y. Tang and C. Cao	2016	Clinical and prognostic significance of serum transforming growth factor-beta1 levels in patients with pancreatic ductal adenocarcinoma	Braz J Med Biol Res	Duplicate records removed
1737	Zhao, X. L., M. N. Lu, Z. G. Liu, M. M. Zhang, H. M. Yuan, Z. Q. Dan, D. H. Wang, B. B. Ma, Y. Q. Yang, F. N. Yang, R. F. Sun, L. Li and C. X. Dang	2023	Comprehensive analysis of alfa defensin expression and prognosis in human colorectal cancer	Frontiers in Oncology	Duplicate records removed
1738	Zhou, B., J. W. Xu, Y. G. Cheng, J. Y. Gao, S. Y. Hu, L. Wang and H. X. Zhan	2017	Early detection of pancreatic cancer: Where are we now and where are we going?	Int J Cancer	Duplicate records removed
1739	Zhou, Y. F., L. X. Xu, L. Y. Huang, F. Guo, F. Zhang, X. Y. He, Y. Z. Yuan and W. Y. Yao	2014	Combined detection of serum ul16-binding protein 2 and macrophage inhibitory cytokine-1 improves early diagnosis and prognostic prediction of pancreatic cancer	Oncology Letters	Duplicate records removed
1740	Zhu, Y. X., C. H. Li, G. Li, H. Feng, T. Xia, C. H. Wong, F. K. C. Fung, J. H. M. Tong, K. F. To, R. Chen and Y. Chen	2020	LLGL1 Regulates Gemcitabine Resistance by Modulating the ERK-SP1-OSMR Pathway in Pancreatic Ductal Adenocarcinoma	Cellular and Molecular Gastroenterology and Hepatology	Duplicate records removed
1741	Zhu, Y. X., C. H. Li, G. L. Li, H. Y. Feng, T. Xia, C. H. Wong, F. K. C. Fung, J. H. M. Tong, K. F. To, R. F. Chen and Y. C. Chen	2020	LLGL1 Regulates Gemcitabine Resistance by Modulating the ERK-SP1-OSMR Pathway in Pancreatic Ductal Adenocarcinoma	Cellular and Molecular Gastroenterology and Hepatology	Duplicate records removed
1742	Zhuang, L., C. Huang, Z. Ning, L. Yang, W. Zou, P. Wang, C. S. Cheng and Z. Meng	2023	Circulating tumor-associated autoantibodies as novel diagnostic biomarkers in pancreatic adenocarcinoma	Int J Cancer	Duplicate records removed
