# Supplementary material for: The role of novel biomarkers in the early diagnosis of pancreatic cancer: A systematic review and meta-analysis
Source: PLoS One. 2025 May 23;20(5):e0322720. doi: 10.1371/journal.pone.0322720 (PMC12101772; doi:10.1371/journal.pone.0322720)
Supplement: S3 file — (PDF) [file pone.0322720.s003.pdf]

| Study | Year | Data Extractor | Date of Extraction | Meets Inclusion Criteria |
|---|---|---|---|---|
| **Aronsson** | 2018 | Zeyi Zheng and Ziyu Lu | 2024/7/21 | yes |
| **Bauden** | 2015 | Zeyi Zheng and Ziyu Lu | 2024/7/21 | yes |
| **Brand** | 2022 | Zeyi Zheng and Ziyu Lu | 2024/7/21 | yes |
| **Cohen** | 2017 | Zeyi Zheng and Ziyu Lu | 2024/7/22 | yes |
| **Eissa** | 2019 | Zeyi Zheng and Ziyu Lu | 2024/7/22 | yes |
| **Firpo** | 2023 | Zeyi Zheng and Ziyu Lu | 2024/7/22 | yes |
| **Debernardi** | 2015 | Zeyi Zheng and Ziyu Lu | 2024/7/23 | yes |
| **Fujimoto** | 2021 | Zeyi Zheng and Ziyu Lu | 2024/7/23 | yes |
| **Fukutake** | 2015 | Zeyi Zheng and Ziyu Lu | 2024/7/23 | yes |
| **Ganepola** | 2014 | Zeyi Zheng and Ziyu Lu | 2024/7/24 | yes |
| **Haab** | 2024 | Zeyi Zheng and Ziyu Lu | 2024/7/24 | yes |
| **Hirata** | 2017 | Zeyi Zheng and Ziyu Lu | 2024/7/24 | yes |
| **Han** | 2015 | Zeyi Zheng and Ziyu Lu | 2024/7/25 | yes |
| **Henriksen** | 2016 | Zeyi Zheng and Ziyu Lu | 2024/7/25 | yes |
| **Huang** | 2024 | Zeyi Zheng and Ziyu Lu | 2024/7/25 | yes |
| **Kashiro** | 2024 | Zeyi Zheng and Ziyu Lu | 2024/7/26 | yes |
| **Kim** | 2020 | Zeyi Zheng and Ziyu Lu | 2024/7/26 | yes |
| **Lee. D** | 2021 | Zeyi Zheng and Ziyu Lu | 2024/7/26 | yes |
| **Lee. M** | 2021 | Zeyi Zheng and Ziyu Lu | 2024/7/27 | yes |
| **Li** | 2023 | Zeyi Zheng and Ziyu Lu | 2024/7/27 | yes |
| **Majumder** | 2021 | Zeyi Zheng and Ziyu Lu | 2024/7/27 | yes |
| **Masterson** | 2023 | Zeyi Zheng and Ziyu Lu | 2024/7/28 | yes |
| **Matsunaga** | 2017 | Zeyi Zheng and Ziyu Lu | 2024/7/28 | yes |
| **Mayerle** | 2017 | Zeyi Zheng and Ziyu Lu | 2024/7/28 | yes |
| **Mawaribuchi** | 2023 | Zeyi Zheng and Ziyu Lu | 2024/7/29 | yes |
| **Mohamed** | 2015 | Zeyi Zheng and Ziyu Lu | 2024/7/29 | yes |
| **Nakamura** | 2022 | Zeyi Zheng and Ziyu Lu | 2024/7/29 | yes |
| **Nam** | 2022 | Zeyi Zheng and Ziyu Lu | 2024/7/30 | yes |
| **Radon** | 2015 | Zeyi Zheng and Ziyu Lu | 2024/7/30 | yes |
| **Wang** | 2014 | Zeyi Zheng and Ziyu Lu | 2024/7/30 | yes |
| **Sato** | 2020 | Zeyi Zheng and Ziyu Lu | 2024/7/31 | yes |
| **Schultz** | 2014 | Zeyi Zheng and Ziyu Lu | 2024/7/31 | yes |
| **Wen** | 2024 | Zeyi Zheng and Ziyu Lu | 2024/7/31 | yes |
| **Wu** | 2022 | Zeyi Zheng and Ziyu Lu | 2024/8/1 | yes |
| **Xie** | 2015 | Zeyi Zheng and Ziyu Lu | 2024/8/1 | yes |
| **Xie** | 2015 | Zeyi Zheng and Ziyu Lu | 2024/8/1 | yes |
| **Yamada** | 2019 | Zeyi Zheng and Ziyu Lu | 2024/8/1 | yes |
| **Wolrab** | 2021 | Zeyi Zheng and Ziyu Lu | 2024/8/2 | yes |
| **Yu** | 2021 | Zeyi Zheng and Ziyu Lu | 2024/8/2 | yes |
| **Yu** | 2020 | Zeyi Zheng and Ziyu Lu | 2024/8/2 | yes |
| **Zhang** | 2013 | Zeyi Zheng and Ziyu Lu | 2024/8/3 | yes |
| **Zhang** | 2014 | Zeyi Zheng and Ziyu Lu | 2024/8/3 | yes |
| **Zhou** | 2018 | Zeyi Zheng and Ziyu Lu | 2024/8/3 | yes |
