# Supplementary material for: The role of novel biomarkers in the early diagnosis of pancreatic cancer: A systematic review and meta-analysis
Source: PLoS One. 2025 May 23;20(5):e0322720. doi: 10.1371/journal.pone.0322720 (PMC12101772; doi:10.1371/journal.pone.0322720)
Supplement: S4 file — (PDF) [file pone.0322720.s004.pdf]

PRISMA 2020 Checklist

Section and Topic	Item #	Checklist item	Location where item is reported
**TITLE**			
Title	1	The Role of Novel Biomarkers in the Early Diagnosis of Pancreatic Cancer: A Systematic Review and Meta-Analysis	1
**ABSTRACT**			
Abstract	2	Importance: Early detection of pancreatic cancer is essential for improving survival rates. However, noninvasive diagnostic methods are lacking. Novel biomarkers, detectable through liquid biopsy, such as circulating tumor DNA (ctDNA), microRNAs (miRNAs), protein markers, and metabolites, hold promise for early diagnosis.<br>Objective: To evaluate the diagnostic accuracy of novel biomarkers for early detection of pancreatic cancer.<br>Data Sources: A systematic search of PubMed, Embase, Web of Science, and the Cochrane Library was conducted for studies published from January 2014 to May 2024.<br>Study Selection: Studies were included if they evaluated novel biomarkers for early pancreatic cancer detection, reported diagnostic performance metrics (sensitivity, specificity), and had a QUADAS-2 score of ⩾3.<br>Data Extraction and Synthesis: Data on study characteristics, patient demographics, biomarker types, and diagnostic performance were extracted following PRISMA guidelines. A bivariate random-effects model was used to calculate pooled sensitivity, specificity, positive likelihood ratio (PLR), negative likelihood ratio (NLR), and diagnostic odds ratio (DOR). The area under the summary receiver operating characteristic (SROC) curve assessed overall diagnostic accuracy.<br>Main Outcomes and Measures: The primary outcome was the diagnostic accuracy (sensitivity and specificity) of novel biomarkers in detecting early-stage pancreatic cancer.<br>Results: A total of 43 studies involving 19,326 participants were included, with 2,749 patients having stage I or II pancreatic cancer. The pooled sensitivities and specificities were as follows:<br>miRNA Biomarkers: Sensitivity 0.88 (95% CI 0.79-0.93), Specificity 0.91 (95% CI 0.82-0.95), DOR 72.68 (95% CI 26.64-198.24), AUC 0.95.<br>Protein Biomarkers: Sensitivity 0.79 (95% CI 0.70-0.86), Specificity 0.88 (95% CI 0.82-0.93), DOR 27.74 (95% CI 14.32-53.76), AUC 0.90.<br>Metabolite Biomarkers: Sensitivity 0.84 (95% CI 0.73-0.92), Specificity 0.85 (95% CI 0.81-0.88), DOR 31.76 (95% CI 12.38-81.48), AUC 0.90.<br>ctDNA Biomarkers: Sensitivity 0.65 (95% CI 0.48-0.81), Specificity 0.94 (95% CI 0.88-0.97), DOR 27.73 (95% CI 12.91-59.55), AUC 0.92.<br>Subgroup analyses showed combining biomarkers with CA19-9 improved diagnostic accuracy. Sensitivity analyses confirmed the robustness of the findings.<br>Conclusions and Relevance: Novel biomarkers, particularly miRNAs and protein markers, demonstrate high diagnostic accuracy for early pancreatic cancer detection and have potential for clinical application in improving early diagnosis and patient outcomes.	2-3
**INTRODUCTION**			
Rationale	3	Pancreatic cancer is one of the most lethal cancers, with the lowest survival rates and is projected to become the second leading cause of cancer-related deaths by 2030. The primary reason for the high mortality is the late-stage diagnosis when curative treatments are often not feasible. Early-stage disease, which is often asymptomatic or presents with nonspecific symptoms, complicates detection efforts. As a result, the majority of cases are diagnosed at later stages, resulting in poor survival outcomes. Currently, there are no effective methods for early detection, and existing biomarkers, such as serum CA19-9, have limited sensitivity and specificity, particularly in early-stage pancreatic cancer. Therefore, the identification of novel biomarkers through noninvasive methods, such as liquid biopsy, holds great potential for early and accurate diagnosis, which could improve survival rates and clinical outcomes.	3-4
Objectives	4	The objective of this meta-analysis is to evaluate the sensitivity and specificity of various novel biomarkers for the early diagnosis of pancreatic cancer, compare their diagnostic performance, and identify the most promising biomarkers for clinical application. The study aims to provide a comprehensive comparison of the performance of liquid biopsy markers such as circulating tumor DNA (ctDNA), microRNAs (miRNAs), and specific protein markers, with a focus on their potential for noninvasive, early-stage detection of pancreatic cancer.	4
**METHODS**			

PRISMA 2020 Checklist

Section and Topic	Item #	Checklist item	Location where item is reported
Eligibility criteria	5	Inclusion Criteria:<br>Study Design: Cohort studies, case-control studies, and diagnostic test accuracy studies.<br>Participants: Patients suspected of or diagnosed with pancreatic cancer.<br>Biomarkers: Studies evaluating novel biomarkers such as circulating tumor DNA (ctDNA), microRNAs (miRNAs), or specific protein markers for the early diagnosis of pancreatic cancer.<br>Outcomes: Studies reporting on diagnostic performance metrics, including sensitivity, specificity, positive predictive value (PPV), and negative predictive value (NPV).<br>Quality Assessment: Studies with a QUADAS-2 score of 3 or higher were included to ensure sufficient methodological quality.<br>Exclusion Criteria:<br>Non-human studies.<br>Studies lacking complete diagnostic performance data.<br>Duplicate publications.<br>Studies with excessive heterogeneity in design, patient population, or biomarker detection methods.<br>Studies with fewer than 30 participants.<br>Small-scale studies conducted exclusively in specific regions or healthcare systems.<br>Grouping for Synthesis:<br>Studies were grouped based on the following criteria for synthesis:<br>Biomarker Type: Circulating tumor DNA (ctDNA), microRNAs (miRNAs), and specific protein markers.<br>Sample Type: Blood, urine, or other biofluid types used for biomarker detection.<br>Detection Methods: Polymerase chain reaction (PCR), next-generation sequencing (NGS), or other diagnostic techniques for detecting the biomarkers.	5-6
Information sources	6	The following databases and sources were searched to identify relevant studies:<br>PubMed<br>Embase<br>Web of Science<br>Cochrane Library<br>The search was conducted for studies published from January 2014 to May 2024.<br>Additionally, we screened the reference lists of relevant articles to identify any additional studies that may not have been captured through database searches.	6
Search strategy	7	PubMed:<br>#1 "Pancreatic Neoplasms"[Mesh] OR "pancreatic cancer" OR "pancreatic carcinoma" OR "pancreatic tumor"<br>#2 "Early Detection of Cancer"[Mesh] OR "early diagnosis" OR "early detection" OR "screening"<br>#3 "Biomarkers, Tumor"[Mesh] OR "biomarker" OR "biomarkers" OR "circulating tumor DNA" OR "ctDNA" OR "microRNA" OR "miRNA" OR "protein marker" OR "genetic marker"<br>#4 "Sensitivity and Specificity"[Mesh] OR "diagnostic accuracy" OR "sensitivity" OR "specificity" OR "positive predictive value" OR "negative	Supplementary Table 1

PRISMA 2020 Checklist

Section and Topic	Item #	Checklist item	Location where item is reported
		predictive value"<br>#5 #1 AND #2 AND #3 AND #4<br>#6 Filters: Publication date from 2014/01/01 to 2024/05/31; Humans; English<br>Embase:<br>#1 'pancreas tumor'/exp OR 'pancreatic cancer' OR 'pancreatic carcinoma' OR 'pancreatic tumor'<br>#2 'early cancer detection'/exp OR 'early diagnosis' OR 'early detection' OR 'screening'<br>#3 'tumor biomarker'/exp OR 'biomarker' OR 'biomarkers' OR 'circulating tumor DNA' OR 'ctDNA' OR 'microRNA' OR 'miRNA' OR 'protein marker' OR 'genetic marker'<br>#4 'diagnostic accuracy'/exp OR 'sensitivity' OR 'specificity' OR 'positive predictive value' OR 'negative predictive value'<br>#5 #1 AND #2 AND #3 AND #4<br>#6 Filters: Publication year from 2014 to 2024; Human; English<br>Cochrane Central Register of Controlled Trials<br>#1 [mh "Pancreatic Neoplasms"] OR "pancreatic cancer" OR "pancreatic carcinoma" OR "pancreatic tumor"<br>#2 [mh "Early Detection of Cancer"] OR "early diagnosis" OR "early detection" OR "screening"<br>#3 [mh "Biomarkers, Tumor"] OR "biomarker" OR "biomarkers" OR "circulating tumor DNA" OR "ctDNA" OR "microRNA" OR "miRNA" OR "protein marker" OR "genetic marker"<br>#4 [mh "Sensitivity and Specificity"] OR "diagnostic accuracy" OR "sensitivity" OR "specificity" OR "positive predictive value" OR "negative predictive value"<br>#5 #1 AND #2 AND #3 AND #4<br>#6 Filters: Publication date from Jan 2014 to May 2024; Humans; English<br>Web Of Science:<br>#1 TS=("pancreatic cancer" OR "pancreatic carcinoma" OR "pancreatic tumor")<br>#2 TS=("early diagnosis" OR "early detection" OR "screening")<br>#3 TS=("biomarker" OR "biomarkers" OR "circulating tumor DNA" OR "ctDNA" OR "microRNA" OR "miRNA" OR "protein marker" OR "genetic marker")<br>#4 TS=("diagnostic accuracy" OR "sensitivity" OR "specificity" OR "positive predictive value" OR "negative predictive value")<br>#5 #1 AND #2 AND #3 AND #4<br>#6 Filters: Timespan=2014-2024; Document Types=Article; Languages=English	
Selection process	8	The study selection process involved the following methods:<br>Screening: Two independent reviewers screened the titles and abstracts of all retrieved articles to determine if they met the inclusion criteria.<br>Full-text Review: Full-text articles were obtained for studies that met the inclusion criteria or when eligibility was uncertain based on the title and abstract.<br>Discrepancy Resolution: Any discrepancies between the reviewers were resolved through discussion. If necessary, a third reviewer was consulted to resolve disagreements.<br>Automation Tools: No specific automation tools were mentioned in the selection process; the process was conducted manually by the reviewers.	6

Section and Topic	Item #	Checklist item	Location where item is reported
Data collection process	9	Data Extraction: Two independent reviewers extracted data from each study using a standardized data extraction form.<br>Independence: The reviewers worked independently to extract the following information from each study:<br>Study Characteristics: Author, publication year, country, study design, and sample size.<br>Patient Characteristics: Age, sex, and disease stage.<br>Biomarker Details: Specific biomarkers evaluated (e.g., ctDNA, miRNAs, protein markers) and the detection methods used (e.g., PCR, NGS).<br>Diagnostic Performance Metrics: Sensitivity, specificity, positive predictive value (PPV), negative predictive value (NPV), and 95% confidence intervals.<br>Quality Assessment: QUADAS-2 scores for the domains of patient selection, index test, reference standard, and flow and timing.<br>Discrepancy Resolution: Any discrepancies between the reviewers were resolved by consensus or by consulting a third reviewer to ensure accuracy and consistency.<br>Obtaining or Confirming Data: In cases where data were unclear or incomplete, attempts were made to contact the study investigators to confirm or obtain additional information.<br>Automation Tools: No automation tools were used in the data extraction process. All data collection was done manually by the reviewers.	6-7
Data items	10a	Diagnostic Performance Metrics:<br>Sensitivity: The proportion of true positives identified by the biomarker for early diagnosis of pancreatic cancer.<br>Specificity: The proportion of true negatives identified by the biomarker.<br>Positive Predictive Value (PPV): The proportion of true positives among all individuals with a positive test result.<br>Negative Predictive Value (NPV): The proportion of true negatives among all individuals with a negative test result.<br>Diagnostic Odds Ratio (DOR): A measure combining sensitivity and specificity to evaluate the overall performance of the biomarker.<br>Likelihood Ratios (Positive and Negative): The likelihood that a positive or negative test result would occur in patients with the disease compared to those without the disease.<br>Time Points and Analyses:<br>Data were sought from studies that provided diagnostic performance metrics for early diagnosis or detection of pancreatic cancer, regardless of time point. Only studies that reported these measures for the relevant biomarkers were included. If a study provided multiple time points or analyses, the data for the first or most relevant diagnostic evaluation were extracted. If results were reported for multiple populations, we focused on those that were most relevant to the inclusion criteria (patients suspected of or diagnosed with pancreatic cancer).	6-7
	10b	Study Characteristics:<br>Author(s): The names of the study authors.<br>Year of Publication: The year the study was published.<br>Country/Region: The country or region in which the study was conducted.<br>Study Design: The type of study (e.g., cohort, case-control, diagnostic accuracy).<br>Sample Size: The number of participants in the study.<br>Participant Characteristics:<br>Age: The mean or median age of the study participants.<br>Sex: The sex distribution of participants.	6-7

Section and Topic	Item #	Checklist item	Location where item is reported
		Disease Stage: The clinical stage of pancreatic cancer at the time of diagnosis (early vs. advanced stage).<br>Biomarker Characteristics:<br>Biomarkers Evaluated: Specific biomarkers evaluated, such as ctDNA, miRNAs, or protein markers.<br>Detection Method: The diagnostic method used for biomarker detection, such as PCR, next-generation sequencing (NGS), or other techniques.<br>Quality Assessment:<br>QUADAS-2 Scores: The risk of bias across the four domains (patient selection, index test, reference standard, and flow and timing).<br>Missing or Unclear Information:<br>If data on certain outcomes or variables were unclear or missing from the original publication, efforts were made to contact the corresponding authors to confirm or clarify the information.<br>In cases where contact was not possible or the missing data could not be obtained, the analysis proceeded with the available information, and the potential impact of the missing data was considered in sensitivity analyses.	
Study risk of bias assessment	11	Methods Used for Risk of Bias Assessment:<br>The QUADAS-2 tool (Quality Assessment of Diagnostic Accuracy Studies-2) was used to assess the risk of bias in the included studies.<br>This tool evaluates the risk of bias across four domains:<br>Patient Selection: Was the patient selection process biased?<br>Index Test: Was the test being evaluated administered correctly and consistently?<br>Reference Standard: Was the reference standard used to confirm the diagnosis appropriate and unbiased?<br>Flow and Timing: Were the participant flow and timing consistent across the study?<br>Reviewers and Independence:<br>Two independent reviewers assessed the risk of bias for each included study using the QUADAS-2 tool.<br>The reviewers worked independently to evaluate each domain for risk of bias and assigned a low, high, or unclear risk of bias rating to each domain.<br>Discrepancy Resolution:<br>In cases of disagreement between the two reviewers, discrepancies were resolved through discussion. If consensus could not be reached, a third reviewer was consulted to resolve the conflict.<br>Automation Tools:<br>No automation tools were used in the risk of bias assessment process. The assessment was conducted manually by the reviewers, following the guidelines provided by the QUADAS-2 tool.	7, Figure2
Effect measures	12	1. Diagnostic Performance Metrics:<br>Sensitivity: The proportion of true positives identified by the biomarker for early diagnosis of pancreatic cancer.<br>Specificity: The proportion of true negatives identified by the biomarker.<br>Positive Predictive Value (PPV): The proportion of true positives among all individuals with a positive test result.<br>Negative Predictive Value (NPV): The proportion of true negatives among all individuals with a negative test result.<br>Diagnostic Odds Ratio (DOR): A summary measure combining sensitivity and specificity to evaluate the overall diagnostic accuracy of the	7-8

Section and Topic	Item #	Checklist item	Location where item is reported
		biomarker.<br>Likelihood Ratios (Positive and Negative): These ratios assess the likelihood of a positive or negative test result in patients with pancreatic cancer versus those without it.<br>2. Statistical Analysis:<br>The effect measures were combined using a bivariate random-effects model to produce pooled estimates of sensitivity, specificity, and other diagnostic performance metrics (PPV, NPV, DOR, and likelihood ratios).<br>The pooled estimates were presented with 95% confidence intervals (CIs).<br>Area Under the Curve (AUC): The summary receiver operating characteristic (SROC) curve was used to evaluate the overall diagnostic accuracy of biomarkers, with the AUC serving as a key indicator of diagnostic performance.	
Synthesis methods	13a	Studies were included in the synthesis if they met the predefined inclusion criteria (e.g., cohort, case-control, or diagnostic test accuracy studies; pancreatic cancer patients; novel biomarkers; and diagnostic performance data).<br>A systematic process was followed to ensure that only studies reporting the necessary diagnostic performance outcomes (sensitivity, specificity, PPV, NPV, etc.) were included in the synthesis.<br>We tabulated study characteristics (e.g., study design, patient characteristics, biomarker types, and diagnostic methods) and compared them against the planned grouping criteria (e.g., biomarker type, detection method, and sample type) to ensure that studies were appropriately grouped for synthesis.	7-8
	13b	Handling Missing Data: If studies reported incomplete or missing summary statistics for diagnostic performance metrics (e.g., sensitivity, specificity), efforts were made to contact the study authors to obtain the missing data. If the missing data could not be obtained, the study was excluded from the synthesis or the available data were used, with any potential impact on the findings noted.<br>Data Conversions: For studies that reported diagnostic performance data in different formats (e.g., odds ratios, confidence intervals), we converted data into the common metric (e.g., sensitivity, specificity, DOR) for consistency and comparability across studies. If necessary, we applied standard statistical methods to compute missing values, such as calculating sensitivity and specificity from other provided metrics.	7-8
	13c	Tables: We tabulated the characteristics of included studies, including study design, patient characteristics, biomarker types, diagnostic methods, and the diagnostic performance metrics (sensitivity, specificity, PPV, NPV, DOR).<br>Forest Plots: Forest plots were used to visually display the pooled diagnostic performance estimates (sensitivity, specificity, etc.) for each biomarker, including 95% confidence intervals. This helped to visually compare the results across studies.<br>Summary Receiver Operating Characteristic (SROC) Curves: SROC curves were constructed to present the overall diagnostic accuracy of biomarkers and to compare their performance across studies in a single graphical representation.	7-8
	13d	Meta-analysis: A bivariate random-effects model was used to synthesize the diagnostic performance metrics (sensitivity, specificity, PPV, NPV, DOR, and likelihood ratios). This model accounts for both within-study and between-study variability and provides pooled estimates for each biomarker.<br>Statistical Heterogeneity: The presence and extent of statistical heterogeneity were assessed using the Cochran's Q test and I^2 statistic. A high I^2 value (greater than 50%) indicated substantial heterogeneity between studies.<br>Software: The meta-analysis was performed using Stata version 16 to estimate pooled sensitivity, specificity, and other diagnostic metrics, as well as to create SROC curves and assess heterogeneity.	7-8
	13e	Subgroup Analysis: Subgroup analyses were performed based on key factors such as:<br>Biomarker Type (ctDNA, miRNAs, protein markers)<br>Sample Type (blood, urine, etc.)<br>Detection Methods (e.g., PCR, NGS) These subgroup analyses helped to identify whether the performance of biomarkers varied according to	8

Section and Topic	Item #	Checklist item	Location where item is reported
		these factors.<br>Meta-regression: If appropriate, meta-regression was used to explore the impact of study-level characteristics (e.g., study design, patient demographics, biomarker types) on the pooled estimates of diagnostic performance.	
	13f	Risk of Bias Sensitivity Analysis: Sensitivity analyses were performed to assess the robustness of the results by excluding studies with a high risk of bias as assessed using the QUADAS-2 tool. This allowed us to evaluate whether the inclusion of studies with high bias affected the pooled estimates.<br>Heterogeneity Sensitivity Analysis: Sensitivity analyses were also conducted by excluding studies with extreme heterogeneity ($I^2 > 75\%$) to assess whether these studies influenced the overall results.<br>Data Missingness Sensitivity Analysis: Sensitivity analyses were performed to evaluate the impact of missing data or incomplete reporting on the synthesis results.	7-8
Reporting bias assessment	14	Funnel Plots: Funnel plots were constructed to visually assess publication bias. If studies with smaller sample sizes or lower effect sizes were systematically missing, the plot would show asymmetry, which could suggest the presence of reporting bias.<br>Deeks' Funnel Plot Asymmetry Test: To formally assess the presence of publication bias, Deeks' test for funnel plot asymmetry was applied. This statistical test helps to determine whether the funnel plot is asymmetrical, indicating that smaller or negative studies may have been underreported or omitted from the analysis.<br>Sensitivity Analysis: Sensitivity analyses were conducted by excluding studies that were identified as potentially contributing to publication bias (e.g., studies with unclear reporting or studies with small sample sizes), to examine whether the overall pooled estimates were significantly affected by reporting bias.	8
Certainty assessment	15	GRADE (Grading of Recommendations, Assessment, Development, and Evaluations) Approach:<br>The certainty of the body of evidence for each outcome (sensitivity, specificity, PPV, NPV, DOR, etc.) was assessed using the GRADE approach. This framework evaluates the quality of evidence across studies based on several factors:<br>Risk of Bias: Studies with high risk of bias (e.g., methodological issues identified through QUADAS-2) were downgraded.<br>Inconsistency: If there was substantial statistical heterogeneity (e.g., high I^2 values), the evidence was downgraded for inconsistency.<br>Indirectness: Evidence from studies that were not directly applicable to the clinical context (e.g., studies with differing patient populations or biomarkers) was considered less certain.<br>Imprecision: If confidence intervals were wide or if the number of studies was small, the evidence was downgraded for imprecision.<br>Publication Bias: If publication bias was detected through funnel plot asymmetry or Deeks' test, the certainty was downgraded.<br>Final Quality Rating: Based on the evaluation of these domains, each outcome was classified as having either high, moderate, low, or very low confidence in the body of evidence. This helped to communicate the degree of certainty in the diagnostic performance of each biomarker.<br>Sensitivity Analyses: Sensitivity analyses were also performed to explore whether the exclusion of studies with high risk of bias, or those with extreme heterogeneity, influenced the certainty of the findings.	7-8, Figure2
**RESULTS**			
Study selection	16a	The initial search identified 1,742 studies from various databases, of which 502 duplicates were removed. After screening the titles and abstracts, 222 studies were selected for full-text evaluation. Upon further examination of the full texts, 175 studies were excluded for the following reasons:<br>68 studies were not diagnostic tests,<br>35 studies lacked clear cancer stage definitions,<br>28 studies did not include specific biomarkers,	8, Figure1

PRISMA 2020 Checklist

Section and Topic	Item #	Checklist item	Location where item is reported
		18 studies had incomplete diagnostic data,<br>12 studies were non-human studies,<br>14 studies had insufficient sample sizes.<br>Additionally, 4 studies were excluded due to insufficient numbers for meta-analysis. After applying these exclusion criteria, 43 studies met the eligibility requirements and were included in the final review (Figure 1).<br>This study selection process is illustrated in the PRISMA flow diagram (Figure 1), which summarizes the number of records identified, screened, eligible, and included in the meta-analysis.	
	16b	Several studies appeared to meet the inclusion criteria but were ultimately excluded for the following reasons:<br>76 studies were excluded because they were not randomized controlled trials (RCTs).<br>12 studies were excluded for failing to meet the inclusion criteria related to Traditional Chinese Medicine (TCM) combinations or for using non-Western medicine controls.<br>3 studies were excluded because they did not meet the outcome criteria, or reported only positive cases without indicating trends or complete data.<br>1 study was excluded due to unexplained sample attrition.<br>These exclusions are outlined in the flow diagram (Figure 1), providing transparency regarding the final selection process.	8, figure1
Study characteristics	17	Table 1 shows the characteristics of the included clinical trials.	Table1
Risk of bias in studies	18	Figure2 shows the assessments of risk of bias for each included study.	Figure2
Results of individual studies	19	See Figure3-6.	Figure3-6
Results of syntheses	20a	We included 43 studies in the meta-analysis, published between 2014 and 2024, which evaluated various biomarkers for the early detection of pancreatic cancer. The characteristics of these studies varied in terms of sample sizes, the types of biomarkers investigated, and the reference standards used. Studies were predominantly conducted in Stage I-II pancreatic cancer patients, with a smaller subset focusing on Stage I or Stage II only. The studies employed healthy volunteers, patients with chronic pancreatitis, and patients with precancerous pancreatic conditions as controls.<br>Risk of Bias: The quality assessment, using the QUADAS-2 tool, revealed that:<br>20 studies had a high risk of bias due to potential case-control design issues and unclear patient enrollment processes.<br>12 studies had unclear reporting on patient selection and study design.<br>1 study had a high risk of bias in the index test domain due to unclear interpretation.<br>All studies had low risk in the reference standard domain, with well-defined reference standards like histopathology, CT scan, or biopsy.<br>All studies were rated low risk in the flow and timing domain because of appropriate intervals between index tests and reference standards.	8-9
	20b	Protein Biomarkers:<br>Summary Estimate:<br>Sensitivity: 0.79 (95% CI, 0.70–0.86)<br>Specificity: 0.88 (95% CI, 0.82–0.93)	10-14

Section and Topic	Item #	Checklist item	Location where item is reported
		AUC: 0.90 (95% CI, 0.87–0.93)<br>Pooled DOR: 27.74 (95% CI, 14.32–53.76)<br>Measures of Heterogeneity:<br>Sensitivity: I^2 = 83.61%<br>Specificity: I^2 = 92.79%<br>Significant heterogeneity was observed, which necessitated further investigation into the sources of variability.<br>ctDNA Biomarkers:<br>Summary Estimate:<br>Sensitivity: 0.65 (95% CI, 0.48–0.81)<br>Specificity: 0.94 (95% CI, 0.88–0.97)<br>AUC: 0.92 (95% CI, 0.89–0.94)<br>Pooled DOR: 27.73 (95% CI, 12.91–59.55)<br>Measures of Heterogeneity:<br>Sensitivity: I^2 = 94.33%<br>Specificity: I^2 = 85.08%<br>High heterogeneity was observed, particularly in sensitivity, which could be attributed to differences in study designs and patient populations.<br>miRNA Biomarkers:<br>Summary Estimate:<br>Sensitivity: 0.88 (95% CI, 0.79–0.93)<br>Specificity: 0.91 (95% CI, 0.82–0.95)<br>AUC: 0.95 (95% CI, 0.93–0.97)<br>Pooled DOR: 72.68 (95% CI, 26.64–198.24)<br>Measures of Heterogeneity:<br>Sensitivity: I^2 = 77.36%<br>Specificity: I^2 = 91.27%<br>High heterogeneity was observed, mainly in sensitivity and specificity.<br>Metabolite Biomarkers:<br>Summary Estimate:<br>Sensitivity: 0.84 (95% CI, 0.73–0.92)<br>Specificity: 0.85 (95% CI, 0.81–0.88)<br>AUC: 0.90 (95% CI, 0.87–0.93)<br>Pooled DOR: 31.76 (95% CI, 12.38–81.48)<br>Measures of Heterogeneity:	

Section and Topic	Item #	Checklist item	Location where item is reported
		Sensitivity: $I^2 = 81.17\%$<br>Specificity: $I^2 = 98.05\%$<br>Very high heterogeneity in specificity, indicating variability among study designs, reference standards, and populations.<br>Subgroup Analyses:<br>When biomarkers were combined with CA19-9, diagnostic accuracy improved across all biomarker categories.<br>Studies using healthy volunteers as controls showed diagnostic performance similar to those using patients with chronic pancreatitis or precancerous conditions, suggesting that the biomarkers might avoid the high false-positive rates associated with CA19-9 in certain populations.	
	20c	Investigations of Heterogeneity<br>Several factors likely contributed to the observed heterogeneity in the meta-analysis, including:<br>Study Design: Differences between case-control and cohort studies contributed to variability in diagnostic accuracy, particularly in sensitivity.<br>Control Groups: Studies using different control groups (e.g., healthy volunteers, patients with chronic pancreatitis, and patients with precancerous pancreatic conditions) showed some variability in performance, particularly for specificity.<br>Biomarker Types: The wide range of biomarkers assessed (e.g., protein biomarkers, miRNAs, ctDNA, and metabolites) led to variations in diagnostic performance across different biomarker categories.<br>Cancer Stages: The inclusion of Stage I and Stage II patients in different studies also contributed to heterogeneity, as earlier stages of cancer tend to present with lower diagnostic accuracy.<br>Subgroup Analysis: When biomarkers were combined with CA19-9, diagnostic accuracy improved across all biomarker types, especially for protein biomarkers and miRNA.	15
	20d	Excluding Studies with High Risk of Bias: After excluding studies with high risk of bias (e.g., unclear patient selection or case-control designs), the pooled estimates of sensitivity and specificity did not significantly change, indicating that the results were robust.<br>Excluding Studies with Small Sample Sizes: Excluding studies with sample sizes $\leqslant 50$ showed no significant change in the pooled estimates, further confirming the stability of the results.<br>Leave-One-Out Analysis: A leave-one-out analysis, where each study was removed one by one, revealed that no single study significantly influenced the overall results. This indicates the robustness of the findings and suggests that the meta-analysis is not overly reliant on any single study.<br>Sensitivity to Different Reference Standards: Studies using different reference standards (e.g., histopathology vs. imaging) were also re-analyzed, with no significant changes in the overall diagnostic accuracy measures, indicating that the choice of reference standard did not substantially affect the results.	16
Reporting biases	21	In this section, we assess the risk of reporting bias, which refers to the possibility that studies with certain results (typically positive or significant findings) are more likely to be published, while studies with negative or non-significant results may be underreported or not published at all. The risk of bias due to reporting biases was evaluated for each synthesis conducted in the meta-analysis using several tools, including Deeks' funnel plot asymmetry test and visual inspection of funnel plots for each biomarker category.<br>Assessment of Reporting Bias for Each Synthesis<br>Protein Biomarkers:<br>Deeks' Funnel Plot Test: The p-value for protein biomarkers was 0.29, indicating no significant asymmetry and suggesting that there is no strong evidence of publication bias for protein biomarkers.<br>Visual Inspection of Funnel Plots: The funnel plots appeared symmetrical, further supporting the conclusion that reporting bias is unlikely to	10-16

Section and Topic	Item #	Checklist item	Location where item is reported
		significantly affect the synthesis of results for protein biomarkers.<br>Conclusion: Low risk of reporting bias for protein biomarkers.<br>ctDNA Biomarkers:<br>Deeks' Funnel Plot Test: The p-value for ctDNA biomarkers was 0.29, which also suggests no significant asymmetry in the funnel plot. This indicates that there is no apparent publication bias for ctDNA biomarkers.<br>Visual Inspection of Funnel Plots: The funnel plots did not show significant skew, supporting the findings from the Deeks' test.<br>Conclusion: Low risk of reporting bias for ctDNA biomarkers.<br>miRNA Biomarkers:<br>Deeks' Funnel Plot Test: The p-value for miRNA biomarkers was 0.45, indicating no significant asymmetry and suggesting a low risk of reporting bias for miRNA biomarkers.<br>Visual Inspection of Funnel Plots: The funnel plot showed no signs of asymmetry, reinforcing the conclusion that reporting bias is not a major concern for this category of biomarkers.<br>Conclusion: Low risk of reporting bias for miRNA biomarkers.<br>Metabolite Biomarkers:<br>Deeks' Funnel Plot Test: The p-value for metabolite biomarkers was 0.04, which suggests a potential risk of reporting bias. This indicates some degree of asymmetry in the funnel plot, suggesting that studies with certain findings (e.g., positive results) might be more likely to be published than those with non-significant or negative findings.<br>Visual Inspection of Funnel Plots: The funnel plot for metabolite biomarkers showed some signs of asymmetry, supporting the result from Deeks' test.<br>Conclusion: Moderate risk of reporting bias for metabolite biomarkers, due to the observed funnel plot asymmetry.<br>Summary of Reporting Bias Assessments<br>Protein Biomarkers: Low risk of reporting bias.<br>ctDNA Biomarkers: Low risk of reporting bias.<br>miRNA Biomarkers: Low risk of reporting bias.<br>Metabolite Biomarkers: Moderate risk of reporting bias.<br>The potential reporting bias for metabolite biomarkers suggests that the results for this category should be interpreted with caution, as studies with non-significant results may be underrepresented. However, for protein, ctDNA, and miRNA biomarkers, the evidence suggests that reporting bias is unlikely to have a significant impact on the findings.	
Certainty of evidence	22	1. Protein Biomarkers<br>Risk of Bias: Moderate risk due to the high risk of patient selection bias in 20 studies. While the reference standard was well-defined and the timing domain was low risk, the high risk of selection bias reduces the overall certainty of evidence.<br>Inconsistency: Substantial heterogeneity was observed (I^2 = 83.61% for sensitivity, 92.79% for specificity). This suggests variability in study outcomes, which impacts the consistency of the evidence.<br>Indirectness: The studies primarily focused on pancreatic cancer, a relevant clinical population, making the evidence direct.<br>Imprecision: Although the confidence intervals for the pooled estimates were wide, they were generally precise enough to draw conclusions about the diagnostic accuracy.<br>Publication Bias: Low risk, as indicated by Deeks' funnel plot asymmetry test (p = 0.29).	10-16

Section and Topic	Item #	Checklist item	Location where item is reported
		Conclusion: Moderate certainty in the evidence for protein biomarkers, due to the risk of bias, high heterogeneity, and some imprecision in the estimates.<br>2. ctDNA Biomarkers<br>Risk of Bias: Moderate risk of bias due to case-control design in some studies, but the reference standard was clearly defined, and timing of the tests was appropriate.<br>Inconsistency: High heterogeneity was observed (I^2 = 94.33% for sensitivity, 85.08% for specificity), suggesting significant variability in study results.<br>Indirectness: The evidence is based on studies of ctDNA biomarkers for pancreatic cancer, making the population and interventions directly relevant.<br>Imprecision: Confidence intervals for pooled estimates were wide but acceptable for drawing conclusions about the diagnostic performance of ctDNA biomarkers.<br>Publication Bias: Low risk, as indicated by the Deeks' funnel plot asymmetry test (p = 0.29).<br>Conclusion: Moderate certainty in the evidence for ctDNA biomarkers, based on risk of bias, heterogeneity, and imprecision.<br>3. miRNA Biomarkers<br>Risk of Bias: Moderate risk due to some studies having unclear selection processes. However, the reference standard was well-defined in all studies, and timing was appropriate.<br>Inconsistency: There was significant heterogeneity (I^2 = 77.36% for sensitivity, 91.27% for specificity), which suggests variability in diagnostic accuracy across studies.<br>Indirectness: The studies focused on miRNA biomarkers in pancreatic cancer, making the population and interventions directly relevant.<br>Imprecision: The confidence intervals for pooled estimates were reasonably narrow, contributing to relatively precise conclusions.<br>Publication Bias: Low risk, as indicated by the Deeks' funnel plot asymmetry test (p = 0.45).<br>Conclusion: Moderate certainty in the evidence for miRNA biomarkers, due to risk of bias and heterogeneity.<br>4. Metabolite Biomarkers<br>Risk of Bias: Moderate risk due to case-control designs and unclear selection processes in several studies. However, the reference standard was well-defined, and timing was appropriate.<br>Inconsistency: Significant heterogeneity was observed (I^2 = 81.17% for sensitivity, 98.05% for specificity), suggesting high variability in results.<br>Indirectness: The studies on metabolite biomarkers are directly relevant to the research question, as they were focused on pancreatic cancer.<br>Imprecision: Confidence intervals for pooled estimates were relatively wide, indicating some imprecision.<br>Publication Bias: Moderate risk of reporting bias (p = 0.04), as indicated by the funnel plot asymmetry test.<br>Conclusion: Low to moderate certainty in the evidence for metabolite biomarkers, due to significant heterogeneity, potential reporting bias, and imprecision.<br>Summary of Certainty of Evidence<br>Protein Biomarkers: Moderate certainty due to risk of bias and heterogeneity.<br>ctDNA Biomarkers: Moderate certainty due to risk of bias, heterogeneity, and imprecision.<br>miRNA Biomarkers: Moderate certainty due to risk of bias and heterogeneity.<br>Metabolite Biomarkers: Low to moderate certainty, primarily due to heterogeneity, reporting bias, and imprecision.	

Section and Topic	Item #	Checklist item	Location where item is reported
		Final Conclusion<br>The evidence supporting the diagnostic accuracy of protein, ctDNA, and miRNA biomarkers is of moderate certainty. For metabolite biomarkers, the certainty of evidence is lower, largely due to significant heterogeneity and potential publication bias. The findings for all biomarker categories should be interpreted with caution, particularly those with lower certainty, and further high-quality studies are needed to strengthen the evidence base.	
**DISCUSSION**			
Discussion	23a	miRNA biomarkers: Our results align with studies reporting high diagnostic accuracy for miRNAs in pancreatic cancer, showing their potential due to their stability and role in carcinogenesis.<br>Protein biomarkers: Similar to earlier studies, our analysis demonstrates strong diagnostic performance for protein biomarkers, confirming their value in cancer detection.<br>ctDNA biomarkers: Our results confirm the high specificity but moderate sensitivity of ctDNA biomarkers, which reflects the challenges of detecting low levels of tumor-derived DNA in early stages of cancer. Sensitivity improved after excluding small-sample studies.<br>Metabolite biomarkers: Our findings corroborate previous research, indicating that metabolic changes are significant for cancer detection.<br>Our study focused specifically on stage I and II pancreatic cancer, offering stronger diagnostic performance in early-stage diagnosis compared to studies including all pancreatic cancer patients.	16-17
	23b	Heterogeneity: There was significant heterogeneity in the included studies, likely due to variability in biomarkers, study designs, patient populations, cancer stages, control groups, and detection methods.<br>Publication bias: Metabolite studies may have publication bias, particularly related to sample size.<br>Sample size issues: Sensitivity analyses indicated that heterogeneity might be associated with very large or very small sample sizes, and despite efforts to explain variability, some residual heterogeneity remains.<br>Methodological quality: The methodological quality of the included studies varied, which could influence the pooled estimates and results.	18
	23c	Inconsistent study quality: Although strict inclusion criteria and quality assessments were applied, variations in the methodological quality of the studies could have influenced the results.<br>Data extraction variability: Despite the use of stringent inclusion criteria, inconsistencies in data extraction across studies might affect the comparability of results.	18
	23d	Clinical practice: Incorporating these novel biomarkers into clinical practice could significantly improve early detection, potentially increasing eligibility for curative surgery. We also suggest combining them with traditional CA19-9 testing to enhance diagnostic accuracy and reduce false positives.<br>Policy: The use of these biomarkers could lead to more accurate diagnostic tools, especially for high-risk populations, influencing screening guidelines and healthcare policy.<br>Future research: We recommend:<br>Conducting large-scale, multicenter prospective studies to validate the biomarkers.<br>Standardizing sample handling and detection methods to improve consistency and reliability.<br>Exploring the combined use of biomarkers and imaging techniques to further improve diagnostic accuracy.<br>Investigating the cost-effectiveness and feasibility of routine screening, especially in high-risk groups..	18-19
**OTHER INFORMATION**			
Registration and	24a	We registered at International Prospective Register of Systematic Reviews (number CRD42024553633)	5

Section and Topic	Item #	Checklist item	Location where item is reported
protocol	24b	The review protocol was registered with the International Prospective Register of Systematic Reviews (PROSPERO) database (registration number: CRD42024553633) and the protocol has been published [https://www.crd.york.ac.uk/prospero/display_record.php?ID=CRD42024553633].	5
	24c	We incurred no deviations from the a priori review protocol.	5
Support	25	Non-financial support for the review.	20
Competing interests	26	The authors declare that they have no competing interests.	19
Availability of data, code and other materials	27	All template data collection forms, data extracted from the included studies, data used for all analyses, analytic code, and any other materials used in the review can be obtained by contacting the authors in a reasonable manner.	19

From: Page MJ, McKenzie JE, Bossuyt PM, Boutron I, Hoffmann TC, Mulrow CD, et al. The PRISMA 2020 statement: an updated guideline for reporting systematic reviews. BMJ 2021;372:n71. doi: 10.1136/bmj.n71

For more information, visit: http://www.prisma-statement.org/
